# ﻿Redescription of *Papuanatula* Lugo-Ortiz & McCafferty (Ephemeroptera, Baetidae), with description of a new subgenus and 20 new species

**DOI:** 10.3897/zookeys.1227.138100

**Published:** 2025-02-11

**Authors:** Thomas Kaltenbach, Nikita J. Kluge, Jean-Luc Gattolliat

**Affiliations:** 1 Département de zoologie, Muséum cantonal des Sciences Naturelles, Palais de Rumine, Place Riponne 6, CH-1005 Lausanne, Switzerland Département de zoologie, Muséum cantonal des Sciences Naturelles Lausanne Switzerland; 2 Department of Ecology and Evolution, University of Lausanne (UNIL), CH-1015 Lausanne, Switzerland University of Lausanne (UNIL) Lausanne Switzerland; 3 Department of Entomology, Biological Faculty, Saint-Petersburg State University, Universitetskaya nab., 7/9, Saint Petersburg, 199034, Russia Saint-Petersburg State University Saint Petersburg Russia

**Keywords:** COI, imagos, integrated taxonomy, New Guinea, Sulawesi

## Abstract

Material from the type localities of most described species of *Papuanatula* and rich material newly collected across New Guinea and on the island Sulawesi form the bases for this comprehensive study of the genus, including the description of a new subgenus Papuafiliola**subgen. nov.** and 20 new species. Species delimitation is based on morphology of larvae and partly also imagos, supported by mitochondrial DNA data (COI) for some of the species. The total number of species is augmented from six to 26. The genus has a disjunct distribution on the islands New Guinea (incl. New Britain) and Sulawesi. A key to the larvae of all species is provided. Morphological similarities, the relationship of *Papuanatula* to other genera of Baetidae, and possible explanations for the high diversity in New Guinea are discussed.

## ﻿﻿Introduction

The mayfly fauna of New Guinea, the second largest island of the world after Greenland, comprises remarkably few families and genera. Only five of approximately 40 families (Baetidae, Caenidae, Leptophlebiidae, Palingeniidae, Prosopistomatidae) and 11 of approximately 460 genera worldwide are present (Jacobus et al. 2019; Kluge 2024). From Baetidae, the genera *Centroptella* Braasch & Soldán, 1980, *Cloeon* Leach, 1815, *Labiobaetis* Novikova & Kluge, 1987, *Mystaxiops* McCafferty & Sun, 2005, and *Papuanatula* Lugo-Ortiz & McCafferty, 1999 are known from New Guinea. However, some of these few genera were able to develop a remarkable diversity, including the rather well studied genus *Labiobaetis* Novikova & Kluge, 1987 (Baetidae) with 43 species (Lugo-Ortiz et al. 1999; Kaltenbach and Gattolliat 2018, 2021; Kaltenbach et al. 2021a, 2023; Kluge 2021), and *Thraulus* Eaton, 1881 s.l. (Leptophlebiidae) (Sartori and Salles, pers. comm. June 2024).

New Guinea lies at the interface between the Australian plate and the Pacific plate with ongoing tectonic activity, uplift, volcanism, and rifting as accompanying events. Most of the landmass was formed in the past five million years, after an earlier Cenozoic period characterized by an archipelago structure (Davies 2009). Today, there is an impressive central mountain range (fold belt) across New Guinea, with peaks to almost 4900 m a.s.l.

The biogeography of New Guinea is influenced by a faunal exchange with Australia on one side through repeated land connections during late Cenozoic and, on the other side, by strong affinities with Southeast Asia despite an oceanic barrier; plants and invertebrates are largely of this origin (Allison 2009).

Up to now, the genus *Papuanatula* has been comprised of six species, all endemic to New Guinea, including the island of New Britain, described in a single paper (Lugo-Ortiz and McCafferty 1999). So far, it is the only study on *Papuanatula*. However, the species from New Britain (*P.vaisisi* Lugo-Ortiz & McCafferty, 1999) was already informally described and illustrated by Demoulin (1969) without naming it.

Here, we re-describe the genus *Papuanatula* based on material that was collected with the holotypes and paratypes (the ”additional material” of Lugo-Ortiz and McCafferty 1999) of most known species and rich newly collected material of more than 1000 specimens from across New Guinea. Additionally, a new subgenus and 20 new species are described and illustrated, mostly based on larvae. In some species, winged stages and eggs are also described. *Papuanatula* larvae are characterized by a femur with a row of long, dense setae on the outer margin; the presence of a regular row of long, dense setae on tibia and tarsus; cerci long, primary swimming setae strongly reduced or vestigial; the paracercus strongly reduced or vestigial; glossae of labium much shorter than paraglossae; thoracic sternites usually with protuberances in posterolateral corners; femoral patch absent on all segments; sterno-styliger muscle absent.

Considering the high diversity of *Papuanatula* in New Guinea, the limited collection activities in the past with many still unexplored regions, and the fact that we already have identified several additional new species without describing them, a substantial number of new species should be expected in the future.

## ﻿﻿Materials and methods

The larvae were collected by kick-sampling and preserved in 70%–96% ethanol.

Subimagos were reared by one of us (NK) from mature larvae in glasses with stagnant water. Subsequently, imagos were reared from subimagos placed in containers with wet air, but without water. Imagos were associated with their larval and subimaginal exuviae. Indication of reared material according to the system of Kluge (2024) (e.g., L-S-I♂).

The dissection of larvae was done in Cellosolve (2-Ethoxyethanol) with subsequent mounting on slides with Euparal liquid, using an Olympus SZX7 stereomicroscope. Alternatively, dissection was done in alcohol with subsequent mounting on slides with Canada balsam, using a stereomicroscope MSP 2, and examination with a Leica DM 1000 microscope.

For the delimitation and descriptions of new species, the morphological species concept is used, supported in some cases by mitochondrial DNA (COI).

Redescriptions of known species were done based on additional original material that was collected with the holotypes/paratypes (same location and date), studied by the original authors, and specified as “additional material” in Lugo-Ortiz and McCafferty (1999): the morphology of these specimens is identical to the descriptions and drawings of the corresponding species in the original publication. Additionally, when available, recently collected material was considered as the original material had been stored in alcohol for up to 60 years. Moreover, the same material had been already in alcohol for up to 35 years at the time of the study by Lugo-Ortiz and McCafferty (1999). For *P.tuber* Lugo-Ortiz & McCafferty, no additional material was available, but we could verify the important characters with the help of photographs and videos of the holotype larva. *Papuanatulavaisisi* Lugo-Ortiz & McCafferty was described based on the description and drawings of Demoulin (1969) without investigation of the old material. The type material could not be investigated by us, but the original description and the one of Demoulin (1969) are sufficient for a differentiation. However, this species was only found on the island New Britain and it is unlikely that it also occurs on the main island of New Guinea.

The DNA of some specimens was extracted using non-destructive methods allowing subsequent morphological analysis (see Vuataz et al. 2011 for details). We amplified a 658 bp fragment of the mitochondrial gene cytochrome oxidase subunit 1 (COI) using the primers LCO 1490 and HCO 2198 (Folmer et al. 1994; see Kaltenbach and Gattolliat 2020 for details). Sequencing was done with Sanger’s method (Sanger et al. 1977). The genetic variability between two specimens was estimated using Kimura-2-parameter distances (K2P, Kimura 1980), calculated with the program MEGA 11 (Tamura et al. 2021, http://www.megasoftware.net).

The GenBank accession numbers are given in Table 1, and the nomenclature of gene sequences follows Chakrabarty et al. (2013).

**Table 1.** Sequenced specimens (COI).

Species	Location	Specimen voucher catalogue #	GenBank # (CO1)	GenSeq nomenclature
*P.bessa*	Papua New Guinea: Gulf Prov.	GBIFCH00976130	PQ669726	genseq-4 COI
		GBIFCH00976131	PQ669727	genseq-4 COI
	Papua New Guinea: Morobe Prov.	GBIFCH00976085	PQ669728	genseq-4 COI
	Papua New Guinea: Central Prov.	GBIFCH00976094	PQ669725	genseq-4 COI
*P.balkei* sp. nov.	Papua New Guinea: Eastern Highlands Prov.	GBIFCH00975797	PQ669729	genseq-2 COI
		GBIFCH00975798	PQ669730	genseq-2 COI
	Papua New Guinea: Western Highlands Prov.	GBIFCH00976095	PQ669731	genseq-4 COI
		GBIFCH00976096	PQ669732	genseq-4 COI
*P.dumspinae* sp. nov.	Indonesia: Papua Prov.	GBIFCH00976042	PQ669734	genseq-2 COI
		GBIFCH00975787	PQ669733	genseq-2 COI
*P.parabessa* sp. nov.	Papua New Guinea: Madang Prov.	GBIFCH00976133	PQ669737	genseq-2 COI
		GBIFCH00976137	PQ669740	genseq-2 COI
		GBIFCH00976135	PQ669739	genseq-2 COI
		GBIFCH00976134	PQ669738	genseq-2 COI
	Papua New Guinea: Enga Prov.	GBIFCH00976128	PQ669736	genseq-2 COI
	Papua New Guinea: Eastern Highlands Prov.	GBIFCH00976091	PQ669735	genseq-2 COI
*P.paracopis* sp. nov.	Papua New Guinea: Eastern Highlands Prov.	GBIFCH00976140	PQ669741	genseq-2 COI
*P.paralenos* sp. nov.	Papua New Guinea: Eastern Highlands Prov.	GBIFCH00976064	PQ669742	genseq-2 COI
*P.paratuber* sp. nov.	Indonesia: Papua Prov.	GBIFCH00976113	PQ669743	genseq-2 COI
*P.parvatubera* sp. nov.	Papua New Guinea: Madang Prov.	GBIFCH00976089	PQ669744	genseq-2 COI
*P.webbi* sp. nov.	Indonesia: Papua Barat	GBIFCH00975834	PQ669745	genseq-2 COI
*P.webbi* sp. nov.		GBIFCH00975835	PQ669746	genseq-2 COI

Photographs of larvae in toto were taken using a Canon EOS 6D camera and processed with the programs Adobe Photoshop Lightroom (http://www.adobe.com) and Helicon Focus v. 5.3 (http://www.heliconsoft.com). Photographs of larval, subimaginal and imaginal parts were taken with a DMC 4500 camera on a Leica M205C stereomicroscope, and an Olympus SC 50 camera on an Olympus BX43 microscope, processed with the program Olympus Cell Sense v. 3.2. SEM pictures were taken using a FEI Quanta FEC 250 electron microscope (Thermo Fisher; 15 nm cover of Au-Pd 60/40). Photographs were subsequently enhanced with Adobe Photoshop Elements 13.

The distribution maps were generated with the program SimpleMappr (https://simplemappr.net, Shorthouse 2010). Google Earth (http://www.google.com/earth/download/ge/) was used to attribute approximate GPS coordinates to sample locations in Lugo-Ortiz and McCafferty (1999). Coordinates of sample locations are given in Table 2.

**Table 2.** GPS coordinates of locations of *Papuanatula* specimens (TL: type locality).

Species	Location	TL	Coordinates
*P.bessa*	Papua New Guinea: Morobe Prov.	TL	07°20'14"S, 146°42'57"E
			07°20'15"S, 146°43'44"E
			06°51'04"S, 146°48'04"E
			07°14'49"S, 146°01'20"E
	Papua New Guinea: Gulf Prov.		07°05'40"S, 145°44'28"E
	Papua New Guinea: Central Prov.		08°20'31"S, 146°59'49"E
	Indonesia: Papua Prov.		02°35'36"S, 140°38'02"E
*P.copis*	Papua New Guinea: Morobe Prov.	TL	07°12'34"S, 146°48'55"E
			07°14'49"S, 146°01'20"E
*P.lenos*	Papua New Guinea: Morobe Prov.	TL	07°20'14"S, 146°42'57"E
*P.plana*	Papua New Guinea: Morobe Prov.	TL	07°20'15"S, 146°43'44"E
*P.tuber*	Papua New Guinea: Morobe Prov.	TL	07°20'15"S, 146°43'44"E
*P.balkei* sp. nov.	Papua New Guinea: Eastern Highlands Prov.	TL	07°01'42"S, 145°49'48"E
	Papua New Guinea: Western Highlands Prov.		05°16'20"S, 144°33'11"E
*P.cyclopomontana* sp. nov.	Indonesia: Papua Prov.	TL	02°27'45"S, 140°22'01"E
*P.dumspinae* sp. nov.	Indonesia: Papua Prov.	TL	01°06'35"S, 133°56'51"E
*P.duplex* sp. nov.	Papua New Guinea: Simbu Prov.	TL	06°42'29"S, 144°59'48"E
*P.epibessa* sp. nov.	Papua New Guinea: Simbu Prov.		05°49'02"S, 145°05'16"E
			05°48'03"S, 145°04'09"E
		TL	05°49'S, 145°04'30"E
*P.heterochaeta* sp. nov.	Indonesia: Papua Prov.	TL	02°27'45"S, 140°22'01"E
			02°35'36"S, 140°38'02"E
*P.normungulata* sp. nov.	Indonesia: Sulawesi	TL	02°57'36"S, 119°22'06"E
*P.obscura* sp. nov.	Indonesia: Papua Prov.	TL	04°05'32"S, 138°56'46"E
*P.obscurella* sp. nov.	Indonesia: Papua Prov.	TL	04°05'32"S, 138°56'46"E
*P.parabessa* sp. nov.	Papua New Guinea: Madang Prov.	TL	05°13'23"S, 144°37'17"E
			05°12'42"S, 144°35'31"E
	Papua New Guinea: Enga Prov.		05°38'06"S, 143°55'20"E
	Papua New Guinea: Eastern Highlands Prov.		05°52'45"S, 145°23'13"E
*P.paracopis* sp. nov.	Papua New Guinea: Eastern Highlands Prov.	TL	05°56'48"S, 145°22'14"E
*P.paralenos* sp. nov.	Papua New Guinea: Eastern Highlands Prov.	TL	07°01'42"S, 145°49'48"E
			05°52'45"S, 145°23'13"E
	Papua New Guinea: Morobe Prov.		07°14'49"S, 146°01'20"E
	Papua New Guinea: Enga Prov.		05°38'06"S, 143°55'20"E
	Papua New Guinea: Simbu Prov.		05°49'02"S, 145°05'16"E
	Papua New Guinea: Gulf Prov.		07°05'40"S, 145°44'28"E
	Papua New Guinea: Central Prov.		08°20'31"S, 146°59'49"E
*P.paratuber* sp. nov.	Indonesia: Papua Prov.	TL	01°06'35"S, 133°56'51"E
*P.parvatubera* sp. nov.	Papua New Guinea: Madang Prov.	TL	05°24'24"S, 145°38'13"E
*P.pilosa* sp. nov.	Indonesia: Papua Prov.	TL	01°06'35"S, 133°56'51"E
*P.pluresetae* sp. nov.	Papua New Guinea: Simbu Prov.		05°49'02"S, 145°05'16"E
		TL	05°48'03"S, 145°04'09"E
			05°49'S, 145°04'30"E
*P.webbi* sp. nov.	Indonesia: Papua Barat	TL	00°51'45"S, 132°49'48"E
*P.zebrata* sp. nov.	Indonesia: Papua Prov.	TL	04°05'32"S, 138°56'46"E
*P.stenophylla* sp. nov.	Indonesia: Papua Prov.	TL	02°27'45"S, 140°22'01"E
*P.tuberculata* sp. nov.	Indonesia: Papua Prov.	TL	04°05'32"S, 138°56'46"E

The dichotomous key was elaborated with the support of the program DKey v. 1.3.0 (http://drawwing.org/dkey, Tofilski 2018).

The terminology follows Kluge (2004). The term “blank” is used to describe an unpigmented area of cuticle (Kluge et al. 2023). The term “posterior seta/setae” is used for long setae in posterior position of the claw (approximately opposite to the distalmost denticle), as proposed by Kluge and Novikova (2014).

### ﻿﻿Abbreviations

**MZB**Museum Zoologicum Bogoriense (Indonesia)

**MZL**Muséum cantonal des Sciences Naturelles, Lausanne (Switzerland)

**SPbU** Saint-Petersburg State University (Russia)

**ZSM/SNSB**Zoologische Staatssammlungen München (Germany)

## ﻿﻿Results

### ﻿﻿ Papuanatula

Taxon classificationAnimaliaEphemeropteraBaetidae

Lugo-Ortiz & McCafferty, 1999

67323A68-5CF8-5B53-A343-ED508B4AE6DF

#### Type species.

*Papuanatulatuber* Lugo-Ortiz & McCafferty, 1999, by original designation (Lugo-Ortiz and McCafferty 1999)

#### Diagnosis.

**Larval characters.** The following combination of characters distinguishes *Papuanatula* from all other genera of Baetidae: antennal scapes basally narrow, distally broad; labrum much wider than long; labium with glossae much shorter than paraglossae; maxillary palp with two segments; both mandibles outside laterally with some long, robust, simple setae; outer side of each femur usually with single regular row of long, hair-like setae; femoral patch absent on all legs; patella-tibial suture usually present on all legs; tibia-tarsal condylus (originally located on outer side) turned to anterior side; anterior side of each tibia usually with regular row of setae similar to that on femur; anterior side of each tarsus usually with regular row of setae similar to femur and tibia; tarsus with conspicuous, long seta subdistally on inner margin; claw with single row of denticles and one or several posterior setae; hind protoptera absent or vestigial; tergalii present on abdominal segments II–VII; paraproct usually with prolongation on proximal margin; cerci usually longer than body length; paracercus strongly reduced or vestigial.

#### Redescription.

**Larva. *Head*. *Antenna*.** Scape basally narrow, distally broader. ***Labrum*** (Fig. 3a, b). Sub-rectangular, laterally convex to angulate, much wider than long. Distal margin with medial, shallow emargination. Ventrally with long, feathered setae on medial margin; several small, stout setae near anterolateral margin. ***Right mandible*** (Fig. 3e). Long, with incisor elongated (blade-like; usually worn toward the end of each instar); or shortened, incisor without elongation. Prostheca stick-like, distally denticulate; margin between prostheca and mola straight, usually with minute denticles; mola with setae on proximal corner. ***Left mandible*** (Fig. 3f). Long, with incisor elongated (blade-like; usually worn toward the end of each instar); or shortened, incisor without prolongation. Prostheca robust, distolaterally denticulate; margin between prostheca and mola straight, usually with minute denticles; mola with setae on proximal corner. Both mandibles with outer lateral margins almost straight or slightly convex, mediolaterally with some long, robust, simple setae near margin. ***Hypopharynx and superlinguae*** (Fig. 3d). Lingua at least slightly longer than superlinguae. Apex of lingua with well-developed, broad bunch of setae-like spines (exceptionally pair of bunches). Distolateral margin of superlinguae with medium, fine setae; lateral margins almost straight. ***Maxilla*** (Fig. 3g). Apically with three stout canines and three denti-setae; distal denti-seta tooth-like, slightly bent against canines; other denti-setae slender and pectinate; galea-lacinia usually with two stout, simple setae under canines; medially with one medium, feathered seta and 3–5 medium to long spine-like setae. Maxillary palp with two segments, apex with short point. ***Labium*** (Fig. 3c). Glossae basally broad, narrowing towards apex, shorter or much shorter than paraglossae; with several long setae at apex and usually one long seta near middle of ventral side. Apex of paraglossae with three rows of long, robust, distally pectinate setae; ventrally usually with one or several short, simple setae in distomedial area; dorsally with two or three long, spine-like setae near inner margin. Labial palp with three segments; segment II with distomedial protuberance hardly developed or absent, or with well-developed, distomedial protuberance; dorsally with row of several long, spine-like setae near outer, distolateral margin.

***Thorax*. *Sterna*** (Figs 106d, 108a). In some species, each thoracic sternum with pair of small protuberances: prothorax with pair of protuberances on lateral sides of prosternal sclerite; mesothorax and metathorax each with pair of such protuberances close to openings of sternal apodemes. ***Terga*.** Partly with conspicuous protuberances; surface usually with small, scattered scales, often with species-specific shape. Hind protoptera absent or vestigial. ***Legs*** (Figs 4a–e, 108b). Long and slender, with long femur and tibia and relatively short tarsus. ***Femur*.** Outer side of each femur usually with single regular row of long, hair-like setae; inner margin with row of short, spine-like setae; femoral patch absent on all legs; apex on posterior side usually with row of short, fine setae. ***Tibia*.** Patella-tibial suture usually present on all legs (the only exception is P. (Papuanatula) normungulata sp. nov. from Sulawesi). Tibia-tarsal condylus (originally located on outer side) turned to anterior side. Anterior (originally outer-anterior) side of each tibia usually with regular row of setae similar to that on femur. Posterior surface usually with scattered, very short, stout setae. ***Tarsus*.** Anterior (originally outer) side of each tarsus usually with regular row of setae similar to femur. ***Claw*.** Slender, apically bent, pointed, with one row of denticles; usually one, sometimes several posterior setae.

***Abdomen*. *Terga*** (Figs 8a, b, 12a, 13a). Partly with striking, species-specific protuberances; surface usually with small, scattered scales, often with species-specific shape. Posterior margins with denticles of different shape. ***Sterna*.** Posterior margins smooth, without denticles. ***Tergalii*.** Present on segments II–VII, in dorsolateral position. Margin smooth, sometimes with minute denticles; with short, simple setae. ***Paraproct*** (Figs 5h, 12e). Median margin usually with minute denticles; posterior margin often with prolongation. Cercotractor usually with minute, marginal spines. ***Caudalii*.** Cerci usually longer than body length, swimming setae strongly diminished and reduced, or vestigial. Paracercus short or vestigial.

#### Description.

**Male imago. *Fore wings*.** With marginal double intercalary veins. ***Hind wings*** absent. ***Genitalia*.** Sterno-styliger muscle completely absent. Gonostyli segment II without significant widenings. Segment III (terminal) of gonostyli nearly as wide as segment II, length varying from slightly exceeding width to twice width.

#### Distribution.

Papua New Guinea, Indonesia: New Guinea, Sulawesi.

### ﻿﻿Subgenus Papuanatula

Taxon classificationAnimaliaEphemeropteraBaetidae

s. str.

F7F36491-7598-5A59-BCB1-B831EB479A93

#### Type species.

Papuanatula (Papuanatula) tuber Lugo-Ortiz & McCafferty, 1999.

#### Diagnosis (larval characters).

Distal part of antenna asymmetrical: each flagellomere with anterior side more convex than posterior side, with brown hypodermal spot near apex of anterior side (Fig. 2b). ***Labrum*** dorsally with submarginal row of long, feathered setae (Fig. 3b). Each seta consists of a stout stem and long processes on both sides. ***Mandibles*.** Both mandibles long, with incisor strongly elongated (in contrast to shortened in Papuafiliola nov.); full-length mandibles present only at the beginning of each instar, often worn at the end of instar. Right mandible (Fig. 3e): kinetodontium deeply separated from incisor; incisor with three denticles; kinetodontium terminating with three or four denticles, with distal denticle longest. Left mandible (Fig. 3f): incisor and kinetodontium fused at most of its length; incisor with three denticles; kinetodontium terminating with three or four denticles, with distal denticle longest. ***Labium***: glossa usually approximately as long as half of paraglossa; with several long setae at apex and usually one long seta near middle of ventral side. Labial palp without real distomedian projection on segment II (Fig. 3c) (in contrast to Papuafiliola subgen. nov.). ***Legs*** (Figs 4a–e, 108b): Outer side of each femur usually with single regular row of long, hair-like setae bearing numerous fine, short branches on all sides. Anterior side of each tibia usually with regular row of setae similar to that on femur. Anterior side of each tarsus usually with regular row of setae similar to femur and tibia. Posterior side of each tarsus with regular row of short, stout setae and one (rarely two) much longer, thinner, pointed seta distad. ***Paraproct*** (Fig. 12e): posterior margin usually with prolongation. ***Pose of subimaginal gonostyli under larval cuticle*** (Fig. 76b). Subimaginal gonostyli developing under cuticle of last instar male larvae folded in “*Labiobaetis*-type” (Kluge 2004: fig. 29I): 1^st^ segment directed laterally, 2^nd^ and 3^rd^ segments directed medially, whereby the 2^nd^ segment is the closest to the posterior margin of the 9^th^ segment.

**Eggs** of examined species without regular relief (Fig. 37d, e).

##### ﻿﻿Species included in *Papuanatula* s. str. Lugo-Ortiz & McCafferty, 1999

###### ﻿Previously described species

*Papuanatulabessa* Lugo-Ortiz & McCafferty, 1999

*Papuanatulacopis* Lugo-Ortiz & McCafferty, 1999

*Papuanatulalenos* Lugo-Ortiz & McCafferty, 1999

*Papuanatulaplana* Lugo-Ortiz & McCafferty, 1999

*Papuanatulatuber* Lugo-Ortiz & McCafferty, 1999

*Papuanatulavaisisi* Lugo-Ortiz & McCafferty, 1999

###### ﻿New species

*Papuanatulabalkei* sp. nov.

*Papuanatulacyclopomontana* sp. nov.

*Papuanatuladumspinae* sp. nov.

*Papuanatuladuplex* sp. nov.

*Papuanatulaepibessa* sp. nov.

*Papuanatulaheterochaeta* sp. nov.

*Papuanatulanormungulata* sp. nov.

*Papuanatulaobscura* sp. nov.

*Papuanatulaobscurella* sp. nov.

*Papuanatulaparabessa* sp. nov.

*Papuanatulaparacopis* sp. nov.

*Papuanatulaparalenos* sp. nov.

*Papuanatulaparatuber* sp. nov.

*Papuanatulaparvatubera* sp. nov.

*Papuanatulapilosa* sp. nov.

*Papuanatulapluresetae* sp. nov.

*Papuanatulawebbi* sp. nov.

*Papuanatulazebrata* sp. nov.

### ﻿﻿ Papuanatula (Papuanatula) bessa

Taxon classificationAnimaliaEphemeropteraBaetidae

Lugo-Ortiz & McCafferty, 1999

3EF77030-6D6A-5AF7-B1A5-9EAC62EA7461

**Figure 1.**
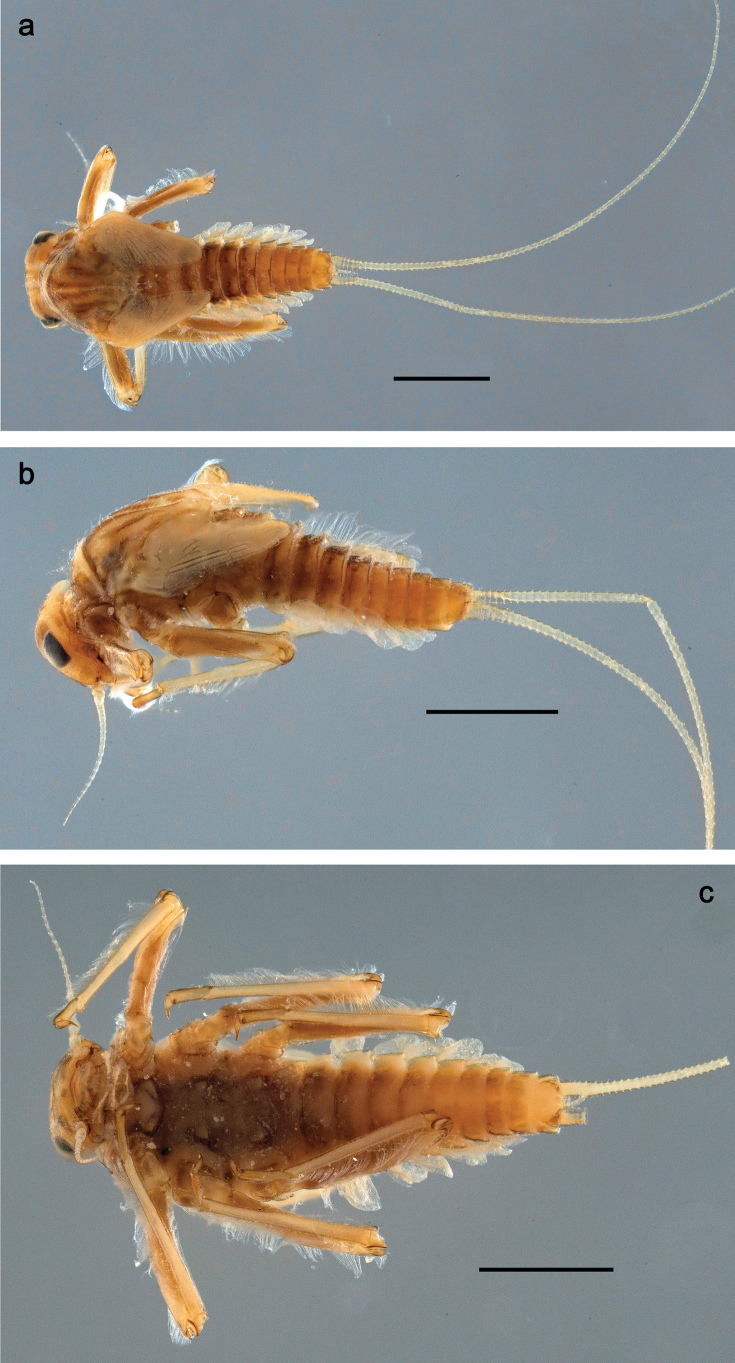
Papuanatula (Papuanatula) bessa, larva, habitus (type locality): **a** dorsal view **b** lateral view **c** ventral view. Scale bars: 1 mm.

**Figure 2.**
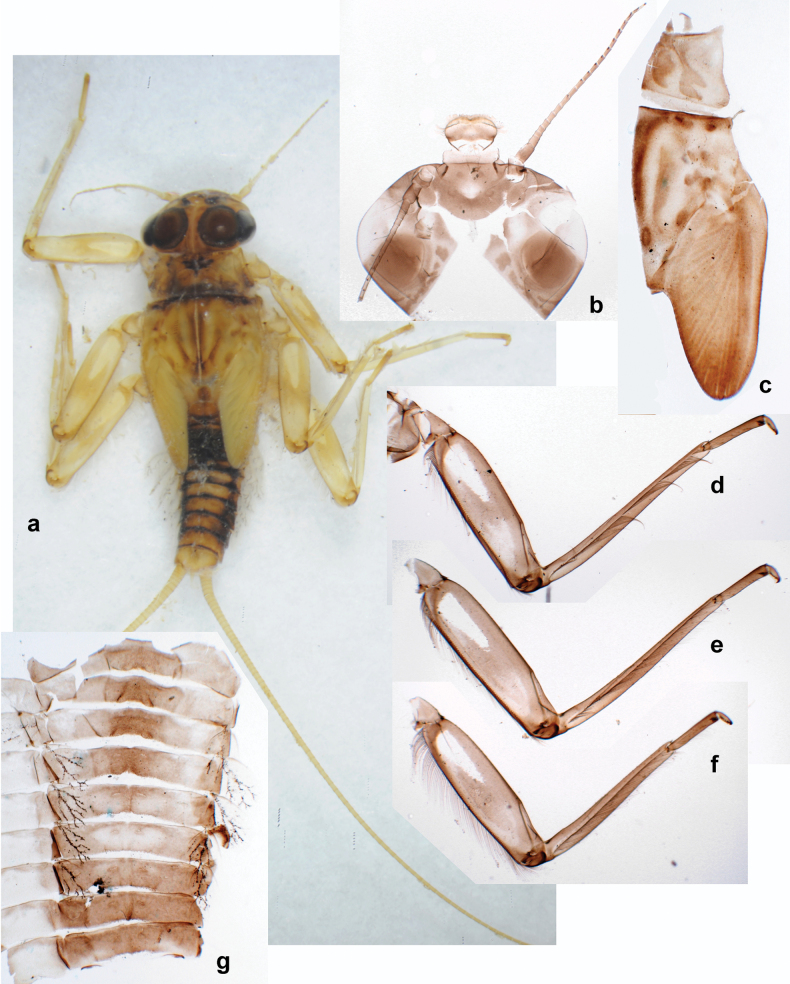
Papuanatula (Papuanatula) bessa, larva (from Waena): **a** male larva **b–g** exuviae: **b** head **c** pronotum and mesonotum **d–f** fore, middle, and hind legs **g** abdomen.

**Figure 3.**
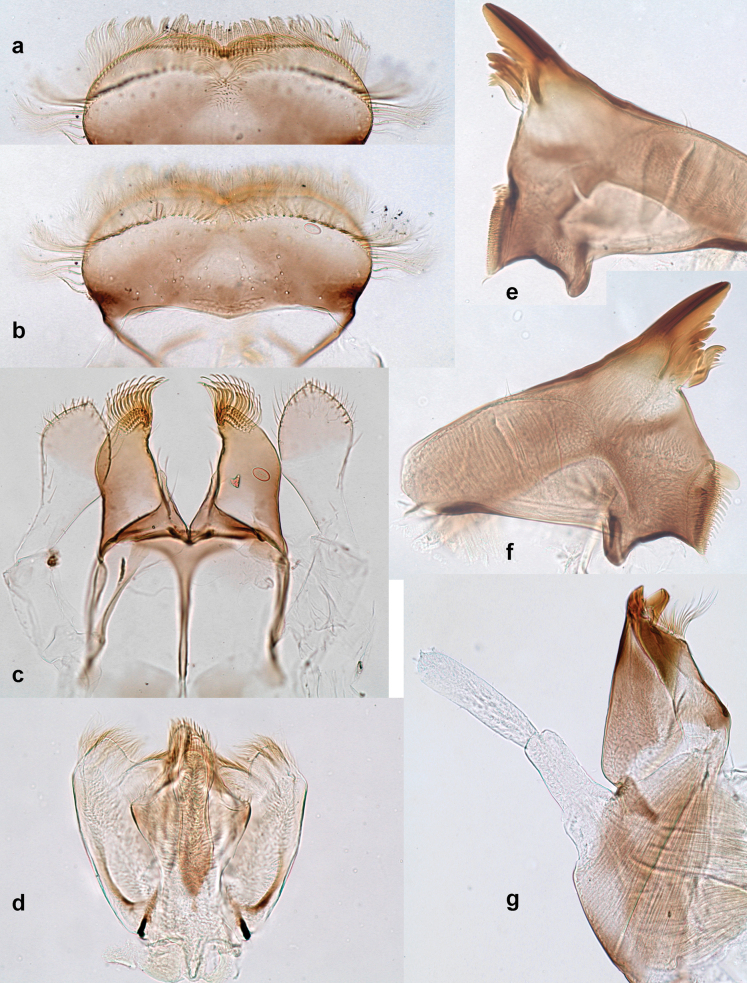
Papuanatula (Papuanatula) bessa, larva (from Waena): **a, b** labrum **c** labium **d** hypopharynx **e, f** left and right mandibles **g** maxilla.

**Figure 4.**
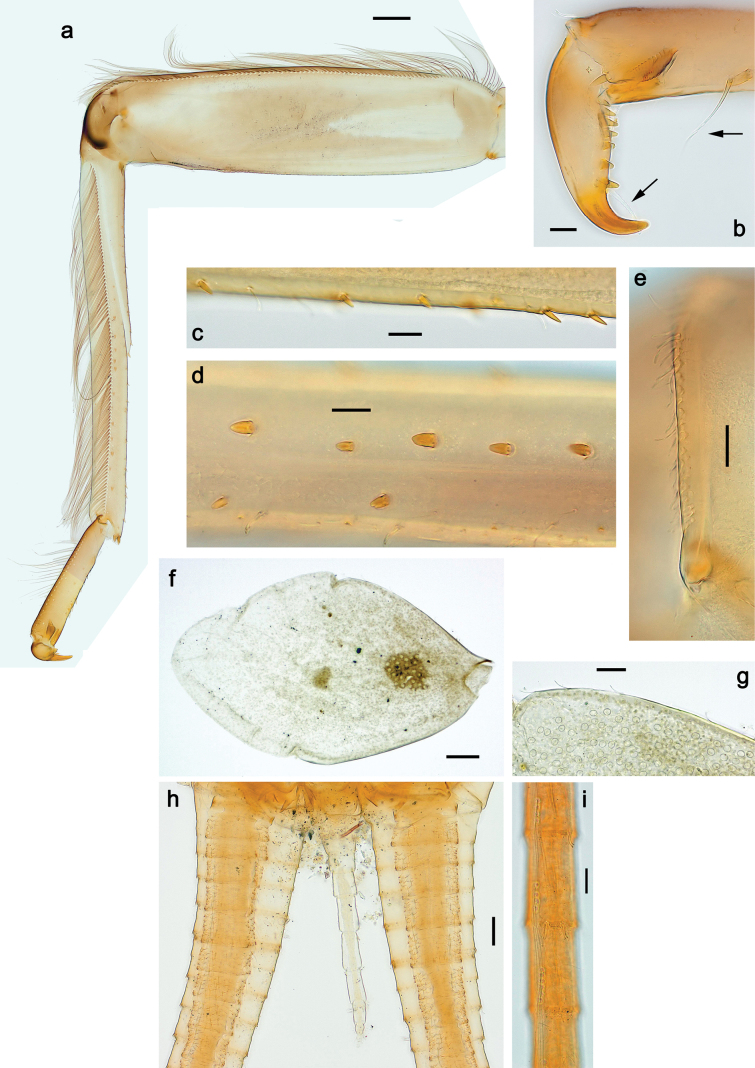
Papuanatula (Papuanatula) bessa, larva (**a–e** from Wagau, Herzog Mts.): **a** foreleg **b** fore claw **c** hind tibia, inner margin **d** hind tibia, posterior surface **e** femur, posterior apex **f, g** tergalius IV (type locality) **h** paracercus (type locality) **I** cercus section (type locality). Scale bars: 50 µm (**a**); 10 µm (**b–d, g, i**); 20 µm (**f, h**).

**Figure 5.**
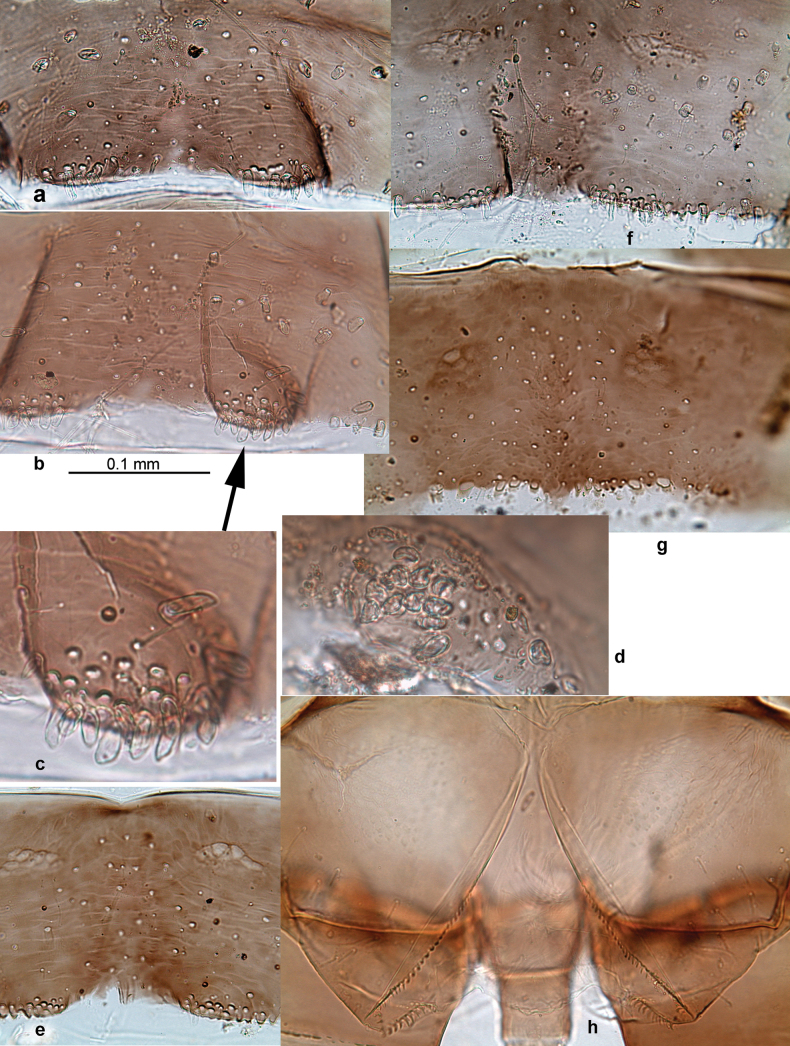
Papuanatula (Papuanatula) bessa, larva, abdomen (from Waena): **a** tergum I **b, c** tergum II **d** apex of protuberance on tergum III **e** tergum IV **f** tergum V **g** tergum IX **h** paraprocts.

**Figure 6.**
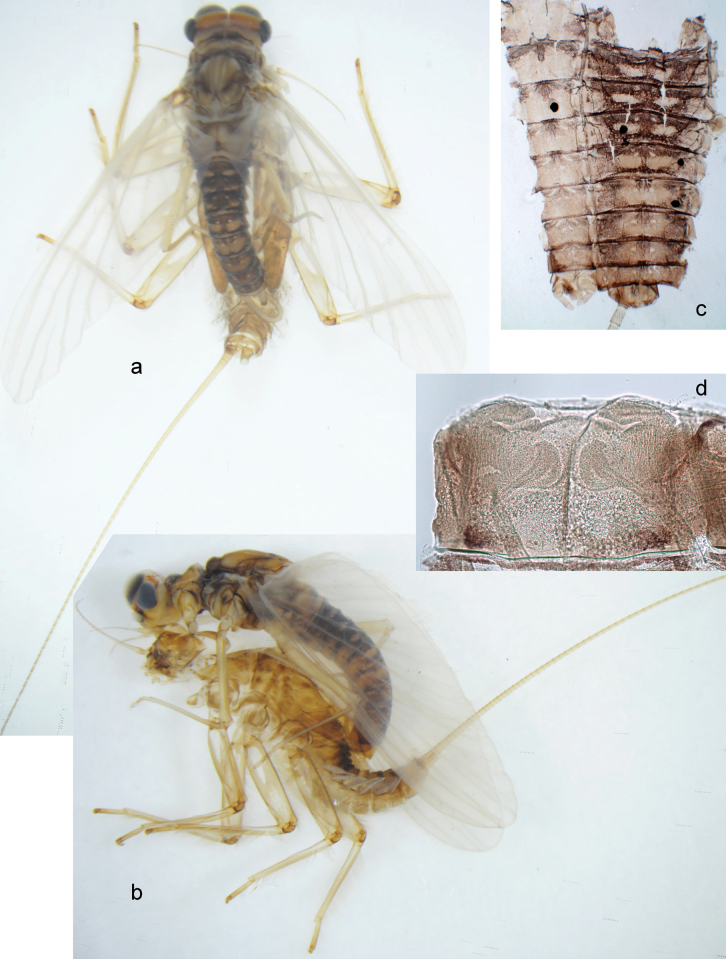
Papuanatula (Papuanatula) bessa (from Waena): **a, b** male subimago with larval exuviae **c** abdomen of male subimago **d** subimaginal gonostyli developing under larval cuticle.

Figs 1 , 2 , 3 , 4 , 5 , 6

Papuanatula bessa Lugo-Ortis & McCafferty, 1999: 63 (larva, ♂ and ♀ subimago).

#### Material examined.

**Type locality (‘additional material’ of original description).** Papua New Guinea • 15 larvae; Morobe Prov., E of Wau, Bulolo Riv.; 900 m; 18.x.1964; leg. WL and WG Peters; 3 on slides; GBIFCH00592634, GBIFCH00976069, GBIFCH01221771; MZL; 12 in alcohol; GBIFCH00976073, GBIFCH00975821; MZL.

#### Other material.

Papua New Guinea • 6 larvae; Gulf Prov., Marawaka, Mala; 07°05'40"S, 145°44'28"E; 1400 m; 11.xi.2006; leg. M. Balke & Kinibel; (PNG 90); 1 on slide; GBIFCH00976130; MZL; 5 in alcohol; GBIFCH00975772, GBIFCH00976022, GBIFCH00976129, GBIFCH00976131; MZL • 8 larvae; Morobe Prov., Menyamya, Mt Inji; near 07°14'49"S, 146°01'20"E; 1700 m; 14.xi.2006; leg. M. Balke & Kinibel; (PNG 96); in alcohol; GBIFCH00975779, GBIFCH00976085; MZL • 2 larvae; Central Prov., Tapini; 08°20'31"S, 146°59'49"E; 870 m; 29.x.2007; leg. Kinibel; (PNG 161); in alcohol; GBIFCH00975785, GBIFCH00976094; MZL • 9 larvae; Morobe Prov., Wagau, Herzog Mts; 06°51'04"S, 146°48'04"E; 1150 m; 19.xi.2006; leg. M. Balke & Kinibel; (PNG 102); 1 on slide; GBIFCH00592633; MZL; 8 in alcohol; GBIFCH00975777, GBIFCH00976081, GBIFCH00976082, GBIFCH00976083; MZL. INDONESIA • Papua, Waena; 8–13.viii.2012; coll. N. Kluge & L. Sheyko: 3 L-S♂, 13 larvae; SPbU.

#### Diagnosis.

**Larva.** The following combination of characters distinguishes *P.bessa* from other species of *Papuanatula* s. str.: body dorsally with irregular row of long, fine, simple setae along midline; metanotum and abdominal terga I–V with medioposterior, broad, paired humps; abdominal terga II–IV dark brown, with brighter oblong markings; tergum V yellow brown with T-shaped dark brown marking (Fig. 1a; Lugo-Ortiz and McCafferty 1999: fig. 5); femur brownish, with wedge-shaped blank in proximal 1/2; paracercus with eight segments.

#### Description.

**Larva** (Figs 1a–5h). Body length 3.8–4.9 mm, cerci much longer than body length (~ 1.5×).

***Cuticular coloration*.** Head, pronotum, mesonotum, and metanotum brownish, with darker and paler areas; fore protoptera with narrow paler lines corresponding to all longitudinal and intercalary veins (Fig. 2c). Thoracic pleura brownish, sterna with brownish and colorless areas. Cuticle of femur with wedge-shape colorless blank on proximal 1/2 and less distinct blank occupying most part of distal 1/2; other surface of femur brownish, apex bordered with darker brown (Figs 2d–f, 4a). Tibia and tarsus brownish (Fig. 2d–f). Abdominal terga mostly brownish, with lateral areas paler; terga V–VI more or less paler than others; sterna mostly colorless (Figs 1a, 2g). Cerci uniformly pale brownish.

***Hypodermal coloration*.** Legs without hypodermal markings. Abdominal terga with extensive, contrasting, dark brown markings of characteristic shape (Figs 1a, 2a). Tissues surrounding tracheae of tergalii partly with brown pigmentation.

***Head*.** Dorsally with row of long, fine, simple setae along midline (as in Figs 26b, 65b). ***Antenna*** (Fig. 2a, b). Length ~ 1.5× head length; otherwise, typical for subgenus. ***Developing turbinate eyes in last instar male larva*.** With larger facets in middle and smaller facets on periphery (as in Fig. 66h). ***Labrum*** (Fig. 3a, b) Length 0.5× maximum width, laterally convex; widened distally; 30–35 long, feathered setae on dorsal surface forming integral, regular transverse row; each seta consists of stout stem and numerous long processes on both sides. ***Right mandible*** (Fig. 3e). Incisor sharply pointed, with two denticles near base. Kinetodontium terminated with three denticles, with distal denticle longest. Otherwise, as typical for subgenus. ***Left mandible*** (Fig. 3f). Incisor sharply pointed, with two denticles near base. Kinetodontium terminated with three denticles, with distal denticle longest. Otherwise, as typical for subgenus. ***Hypopharynx*** (Fig. 3d). As typical for genus. ***Maxilla*** (Fig. 3g). Maxillary palp as long as galea-lacinia. Otherwise, as typical for genus. ***Labium*** (Fig. 3c). Paraglossae widened near middle, with lateral side forming concavity in proximal part; three apical setal rows sharply bent. Glossa as long as half of paraglossa, with finger-like (distal) portion as long as triangular (proximal) portion. Glossa with several long setae at apex and one long seta near middle of ventral side. Labial palp without distomedian projection on segment II; segment III with median margin longer than lateral margin.

***Thorax*. *Sterna*.** With small protuberances on sides of prosternum and close to openings of mesothoracic and metathoracic sternal apodemes (as in Fig. 108a). ***Terga*** with irregular row of long, fine, soft, colorless, simple setae along midline (as in Figs 26b, 65b). Metanotum with medioposterior broad, paired elevations. Metanotum without hind protoptera or their vestiges. ***Legs*** (Figs 2d–f, 4a–e). Fore femur slightly widened in proximal part; hind tibia shorter than others. Ratio of leg segments: fore leg 0.8:1.0:0.3:0.2, middle leg 0.9:1.0:0.3:0.2 and hind leg 1.0:1.0:0.3:0.2. ***Femur*.** Length ~ 4× maximum width. Outer side of each femur with single regular row of long, hair-like setae bearing numerous fine, short branches on all sides (as in Figs 41g, 68b). ***Tibia*.** Patella-tibial suture present on all legs, terminated near middle of inner margin of tibia. Tibia-tarsal condylus (originally located on outer side) turned to anterior side. Anterior (originally outer-anterior) side of each tibia with regular row of setae similar to that on femur. ***Tarsus*.** Anterior (originally outer) side of each tarsus with regular row of similar, but smaller (shorter and narrower) setae. Posterior (originally inner) side of each tarsus with regular row of short, stout, oval setae (looking pointed in profile) and one much longer, thinner, pointed seta distad of them. ***Claw*** with row of six or seven short denticles and one somewhat larger denticle distad of them; long, arched posterior seta on posterior side near distal denticle.

***Abdomen*. *Terga*** (Fig. 5a–g). Long, fine, soft, colorless setae irregularly situated along midline of all abdominal terga (as in Figs 26b, 65b). Each abdominal tergum with pair of submedian protuberances, more prominent on terga I–IV and less prominent to absent on more posterior terga; each protuberance with compact group of short, blunt, parallel-sided, colorless scales with round sockets. Other surface with fewer, sparse scales of such kind. Posterior margins of abdominal terga partly without denticles, partly with short, blunt denticles separated by spaces. ***Tergalii*** (Figs 2g, 4f, g) of abdominal segment I absent; tergalii II–VII subequal, oval. Each tergalius with costal and anal ribs narrow, present on proximal part of tergalius only. Tracheation poorly developed or absent; margins smooth, with short, fine, simple setae. ***Paraproct*** (Fig. 5h) with posterior prolongation bent toward bases of caudalii, with many small, equal denticles on median-posterior margin. ***Caudalii*** (Fig. 4h, i) without swimming setae; vestiges of swimming setae present on distal part of cerci. Paracercus short, consisting of ~ 7–10 segments.

***Pose of subimaginal gonostyli under larval cuticle*.** In mature larva ready to molt to subimago, subimaginal gonostyli packed under larval cuticle in ’*Labiobaetis*-type’ pose: 2^nd^ segments directed medially and bent proximally; 3^rd^ segment directed medially (as continuation of 2^nd^ segment) and narrowed apically, being deformed corresponding to space between subimaginal styliger and larval cuticle (Fig. 6d).

**Subimago. *Texture*.** On all legs of both sexes, each tarsomere covered mostly with blunt microlepides, with pointed microlepides near apex (as in Fig. 70i).

**Imago. *Imago, male*.** Unknown. Following features can be revealed based on subimaginal structure: Turbinate eyes narrow and cylindrical, with stem reddish and faceted surface small, round, black. Legs ochre, without brown hypodermal markings. Abdomen with characteristic hypodermal coloration forming brown and ochre areas (Fig. 6a–c).

***Imago, female*.** Unknown. Judging by description of subimago (Lugo-Ortiz and McCafferty 1999: 64), hypodermal coloration of abdominal terga as in male.

**Egg.** Unknown.

#### Dimension.

Fore wing length (and approximate body length): 4 mm.

#### Distribution.

New Guinea (Fig. 146).

### ﻿﻿ Papuanatula (Papuanatula) copis

Taxon classificationAnimaliaEphemeropteraBaetidae

Lugo-Ortiz & McCafferty, 1999

A7FE8EA9-8D57-58D8-9C9B-33EA0E6025CB

**Figure 7.**
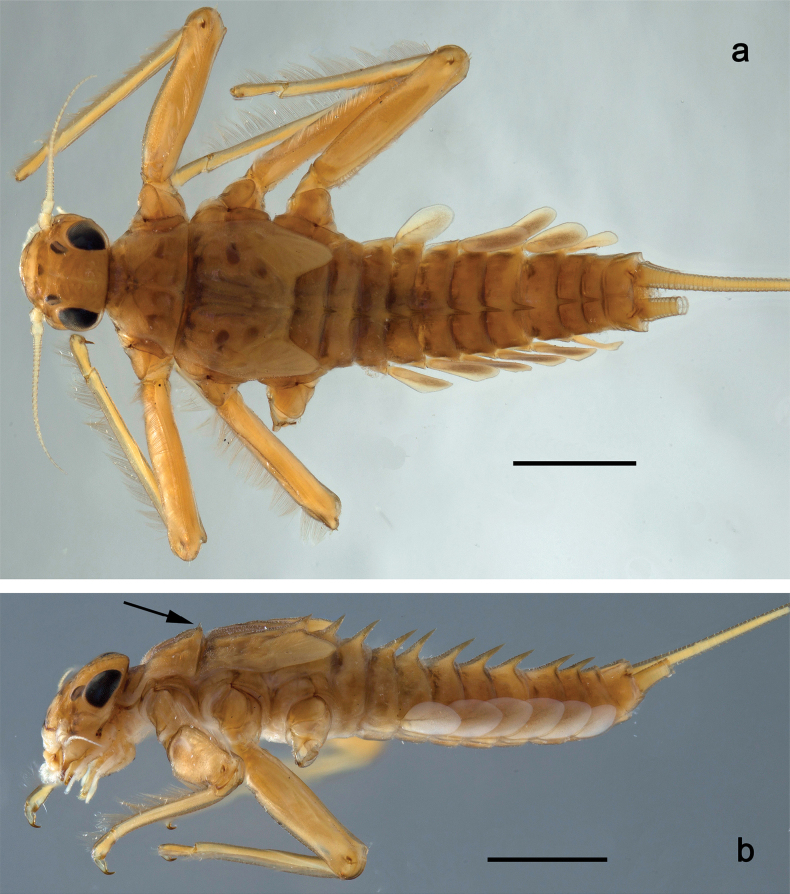
Papuanatula (Papuanatula) copis, larva, habitus (type locality): **a** dorsal view **b** lateral view. Scale bars: 1 mm.

**Figure 8.**
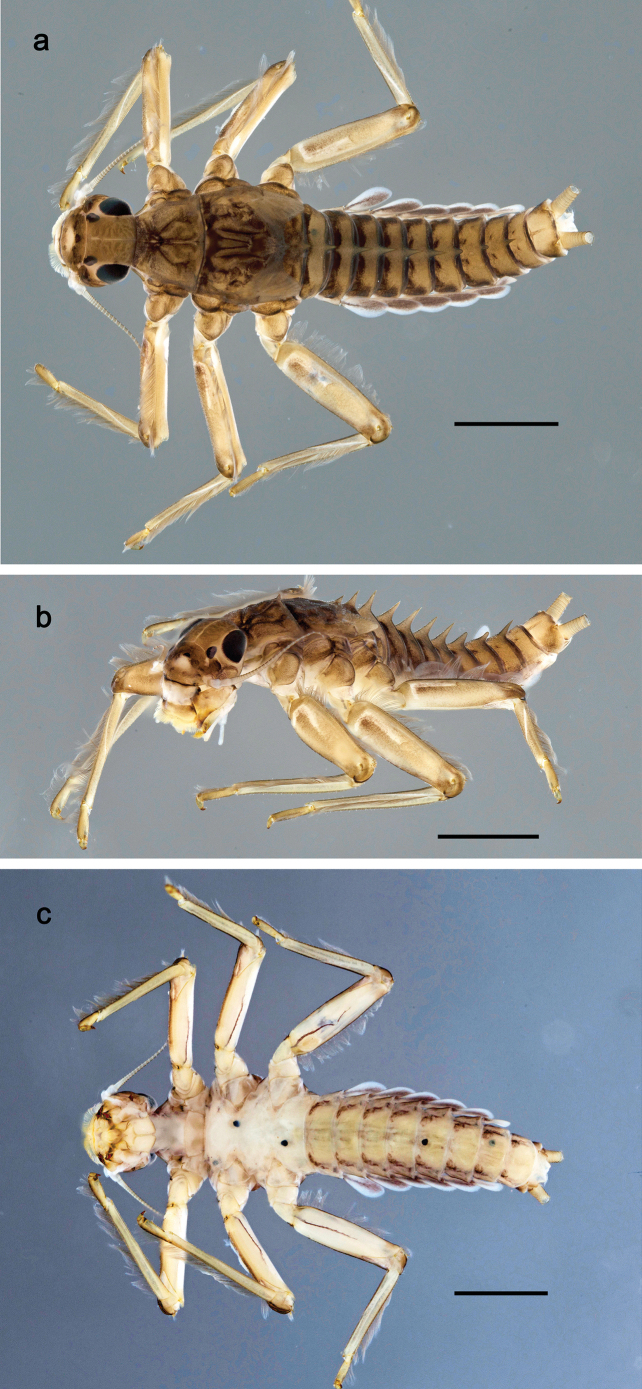
Papuanatula (Papuanatula) copis, larva, habitus: **a** dorsal view **b** lateral view **c** ventral view. Scale bars: 1 mm.

**Figure 9.**
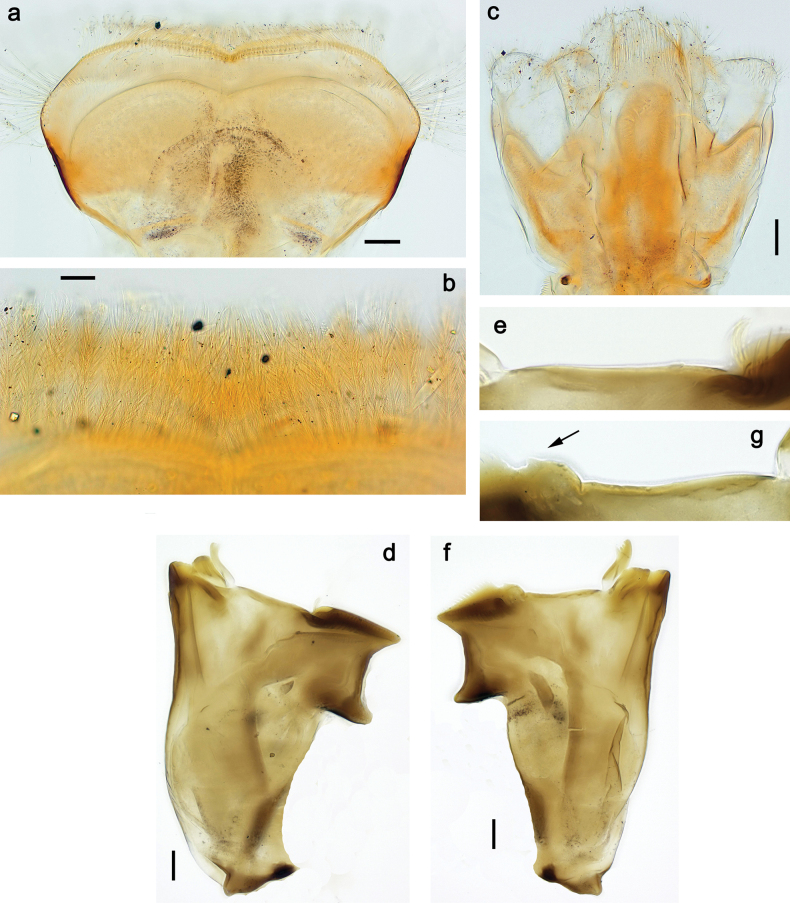
Papuanatula (Papuanatula) copis, larva (type locality): **a** labrum **b** labrum: dorsal, submarginal setae **c** hypopharynx and superlinguae **d** right mandible **e** right mandible: margin between prostheca and mola **f** left mandible **g** left mandible: margin between prostheca and mola (arrow: subtriangular process). Scale bars: 20 µm (**a, c, d, f**); 10 µm (**b**).

**Figure 10.**
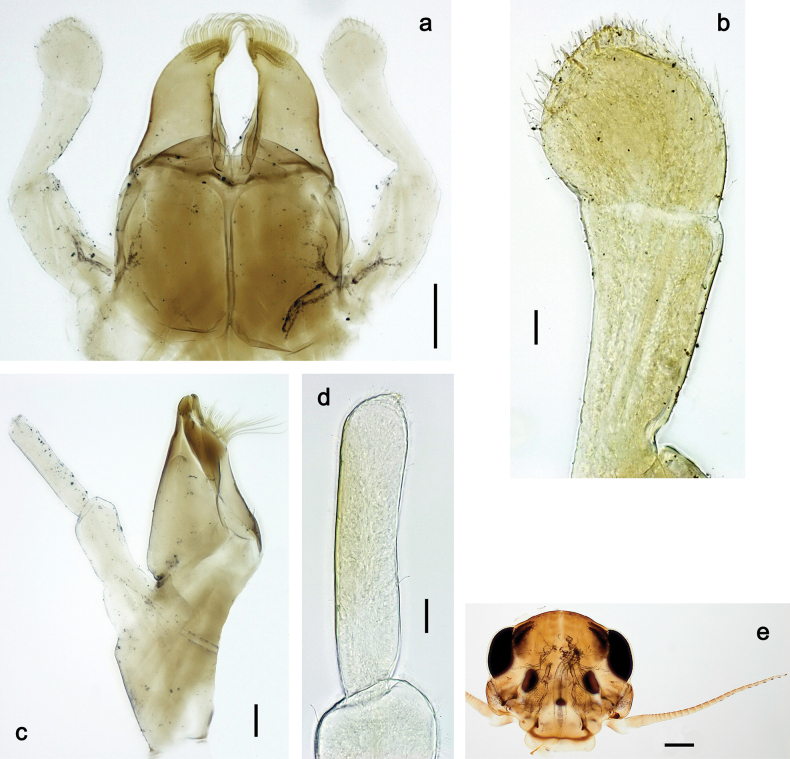
Papuanatula (Papuanatula) copis, larva: **a** labium (type locality) **b** labial palp (type locality) **c** maxilla **d** maxillary palp **e** male head, mature. Scale bars: 50 µm (**a**); 10 µm (**b, d**); 20 µm (**c**); 100 µm (**e**).

**Figure 11.**
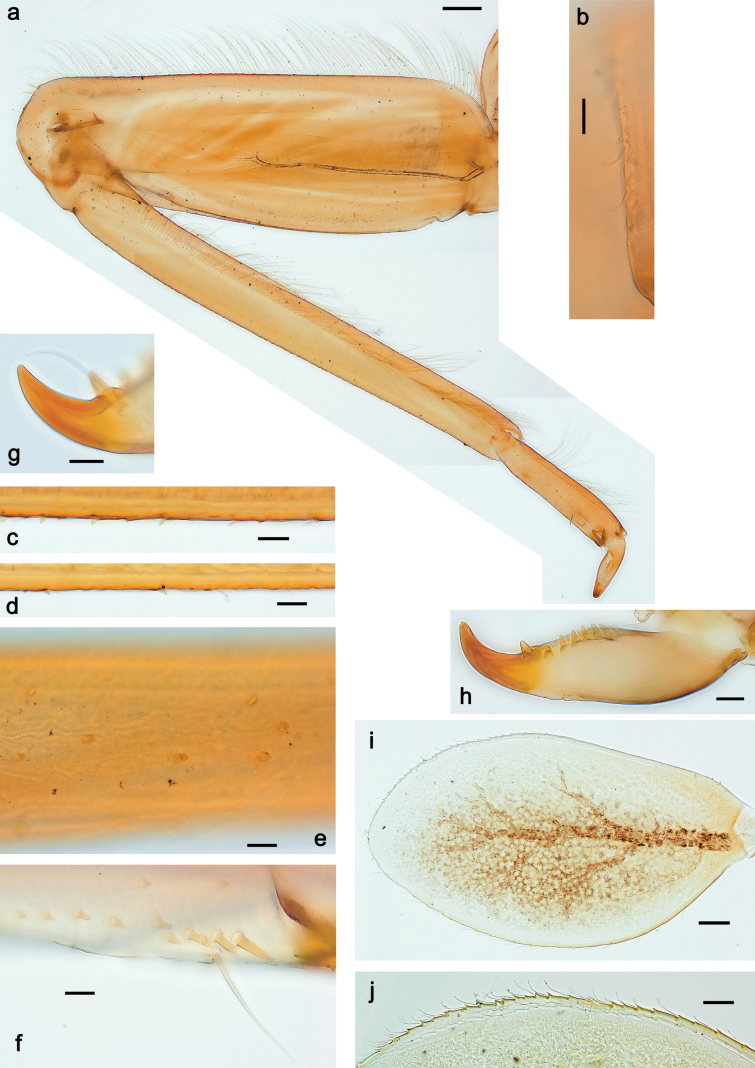
Papuanatula (Papuanatula) copis, larva: **a** foreleg (type locality) **b** middle femur, apex posterior (type locality) **c** middle femur, ventral margin (type locality) **d** middle tibia, ventral margin (type locality) **e** middle tibia, posterior apex (type locality) **f** middle tarsus, ventral margin **g, h** middle claw **I, j** tergalius IV, margin. Scale bars: 50 µm (**a**); 10 µm (**b–e, g, h, j**); 20 µm (**i**).

**Figure 12.**
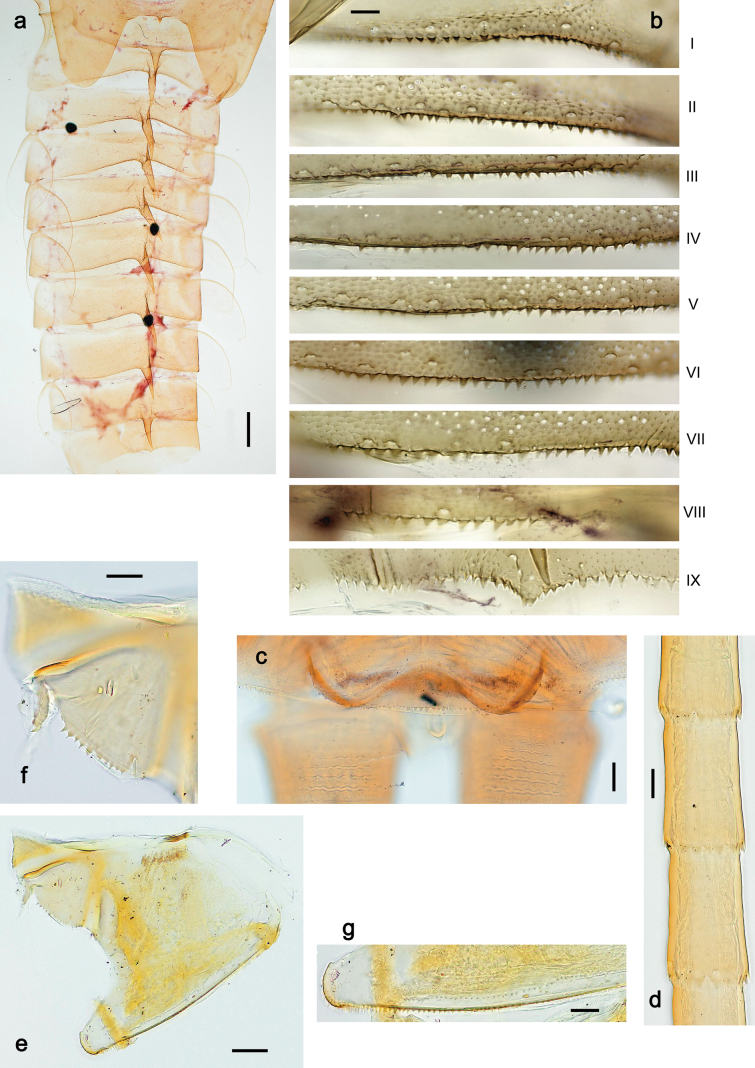
Papuanatula (Papuanatula) copis, larva: **a** metanotum and abdominal terga **b** abdominal terga **c** paracercus (type locality) **d** cercus (type locality) **e** paraproct (type locality) **f** cercotractor (type locality) **g** paraproct, distal margin (type locality). Scale bars: 100 µm (**a**); 10 µm (**b, d–g**); 20 µm (**c**).

**Figure 13.**
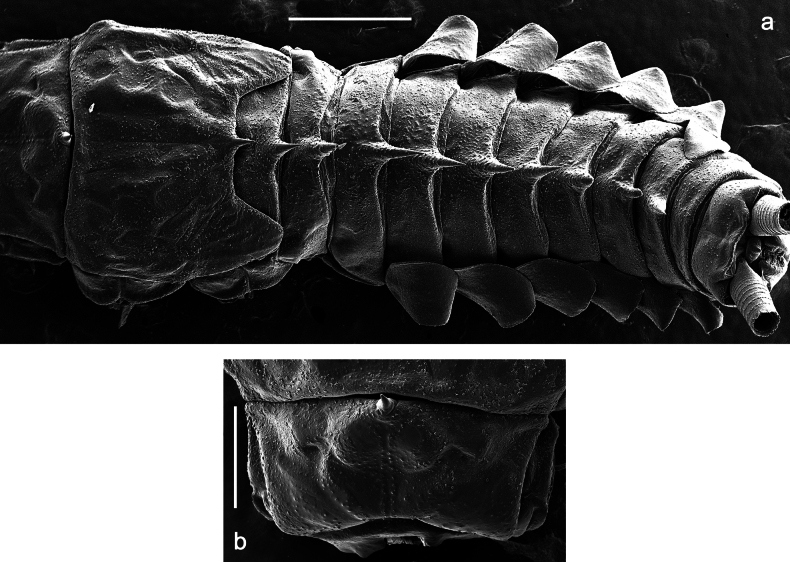
Papuanatula (Papuanatula) copis, larva, SEM: **a** dorsal view **b** pronotum. Scale bars: 0.5 mm (**a**); 300 µm (**b**).

Figs 7 , 8 , 9 , 10 , 11 , 12 , 13

Papuanatula copis : Lugo-Ortiz and McCafferty 1999: 64–65, figs 8–18.

#### Material examined.

**Type locality (‘additional material’ in original description).** Papua New Guinea • 2 larvae; Morobe Prov., Mt Missim, Poverty Cr.; 1600 m; 18.ix.1983; leg. JT and DL Polhemus; 1 in alcohol; GBIFCH00976072; 1 on slides; GBIFCH00592635, GBIFCH00592636; MZL.

#### Other material.

Papua New Guinea • 3 larvae; Morobe Prov., Menyamya, Mt Inji; near 07°14'49"S, 146°01'20"E; 1700 m; 14.xi.2006; leg. M. Balke & Kinibel; (PNG 96); 1 in alcohol; GBIFCH00976084; 2 on slides; GBIFCH00592585, GBIFCH00592586, GBIFCH00976086; MZL.

#### Diagnosis.

**Larva**. The following combination of characters distinguishes *P.copis* from other species of *Papuanatula* s. str.: body dorsally without row of long, fine, simple setae along midline; metanotum and abdominal terga I–VIII medially with conspicuous, long protuberances, with long, fine points, oriented dorsoposteriad, on abdominal segment(s) IX (X) vestigial; labial palp segment II without distomedial protuberance; segment III globular; femur with angulate blank in basal part; paracercus vestigial.

#### Description.

**Larva** (Figs 7–13). Body length 6.0–6.5 mm, cerci much longer than body length (~ 2×).

***Cuticular coloration*** (Figs 7a, b, 8a–c). Head, thorax and abdomen dorsally brown to dark brown. Thorax with complex markings. Abdominal segments I–VI (–IX) anteriorly and laterally darker, VII–IX at least laterally darker. Head and thorax ventrally ecru; abdomen ventrally pale yellow-brown, laterally with brown markings. Legs yellow-brown to brown, femur with angulate blank in basal part. Caudalii yellow-brown.

***Hypodermal coloration*.** Abdominal segments I–IX dorsally with transverse band on posterior margins (Fig. 8a).

***Head*. *Antenna*** (Fig. 10e). Length ~ 1.5× head length. As typical for subgenus. ***Developing turbinate eyes in last instar male larva*** (Fig. 10e) rather narrow, with big distance from each other. ***Labrum*** (Fig. 9a, b). Length 0.4× maximum width, laterally slightly angulate. Dorsal, sub-marginal arc with > 50 densely articulated, feathered setae (30–35 setae according to Lugo-Ortiz and McCafferty 1999: 65). ***Right mandible*** (Fig. 9d, e). Margin between prostheca and mola smooth, without denticles. Otherwise, as typical for subgenus. ***Left mandible*** (Fig. 9f, g). Margin between prostheca and mola, smooth, without denticles. Subtriangular process often undeveloped. Otherwise, as typical for subgenus. ***Hypopharynx*** (Fig. 9c). As typical for genus. ***Maxilla*** (Fig. 10c, d). Maxillary palp slightly longer than galea-lacinia; palp segment II slender, partly sclerotized, approx. as long as segment I, segment I thicker. Otherwise, as typical for genus. ***Labium*** (Fig. 10a, b). As typical for genus. Paraglossa with two spine-like setae near inner margin. Labial palp with segment I 0.8× length of segments II and III combined. Segment II without distomedial protuberance, dorsally with row of 2–5 spine-like setae near outer, distolateral margin. Segment III globular, slightly pointed, 0.7× length of segment II; inner dorsal margin with few feathered setae.

***Thorax*. *Sterna*.** Protuberances poorly developed. ***Terga*** (Figs 7a, b, 8a, b, 13b). Metanotum medially with conspicuous, long protuberance, oriented dorsoposteriad. Immature larva with short, acute, posteromedial protuberance on pro- and mesonotum. ***Legs*** (Fig. 11a–h). Ratio of leg segments: fore leg 0.9:1.0:0.3:0.1, middle leg 0.9:1.0:0.3:0.1 and hind leg 1.1:1.0:0.3:0.1. ***Femur*.** Length ~ 3× maximum width. ***Claw*** with one row of seven or eight denticles and one posterior seta.

***Abdomen*. *Terga*** (Figs 12a, b, 13a). Abdominal terga I–VIII medially with conspicuous, long protuberances, with long, fine points, oriented dorsoposteriad, on abdominal segment(s) IX (X) vestigial. Posterior margin of terga I–IX with small, triangular, pointed denticles. Surface with scattered small, conical, apically rounded scales. ***Tergalii*** (Fig. 11i, j) ovoid, tracheation developed; with brown pigmentation in middle area; margins with minute serration and many short, fine, simple setae. ***Paraproct*** (Fig. 12e–g). Posterior margin with prolongation and with row of many minute denticles. ***Caudalii*** (Fig. 12c, d). Cerci without swimming setae; sometimes few vestigial swimming setae or insertions still present. Paracercus vestigial.

***Pose of subimaginal gonostyli under cuticle*.** Unknown.

**Subimago.** Unknown.

**Imago.** Unknown.

**Egg.** Unknown.

#### Distribution.

New Guinea (Fig. 146).

### ﻿﻿ Papuanatula (Papuanatula) lenos

Taxon classificationAnimaliaEphemeropteraBaetidae

Lugo-Ortiz & McCafferty, 1999

1621D2C3-529B-5AFD-9CA6-5A062B5BA7FE

Figs 14 , 15 , 16

Papuanatula lenos . Lugo-Ortiz and McCafferty 1999: 65–66, figs 19–24.

#### Material examined.

**Type locality (‘additional material’ in original description).** Papua New Guinea • 3 larvae; Morobe Prov., Wau, Hospital Cr.; 1150 m; 20.x.1964; leg. WL and WG Peters; 1 in alcohol; GBIFCH00976076; 2 on slides; GBIFCH00592580, GBIFCH00592533; MZL.

#### Diagnosis.

**Larva.** The following combination of characters distinguishes *P.lenos* from other species of *Papuanatula* s. str.: body dorsally without row of long, fine, simple setae along midline; body dorsally without protuberances; thorax ventrally without protuberances; thorax dorsally without distinct markings; femur anteriorly with hypodermal, large, oblong to drop-shaped, dark brown to purple black marking in basal 1/2; paracercus with nine segments (immature larvae 11); paraproct without posterior prolongation;

#### Description.

**Figure 14.**
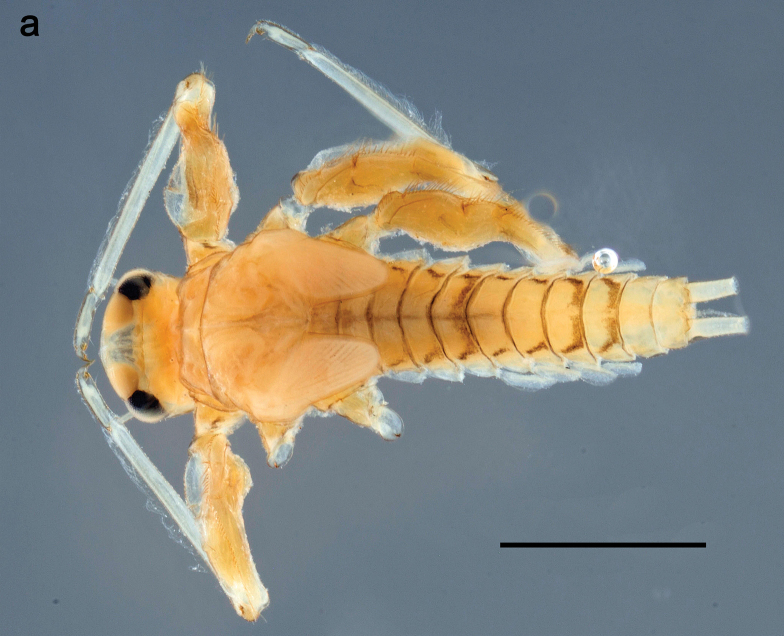
Papuanatula (Papuanatula) lenos, larva, habitus (type locality): **a** dorsal view. Scale bar: 1 mm.

**Figure 15.**
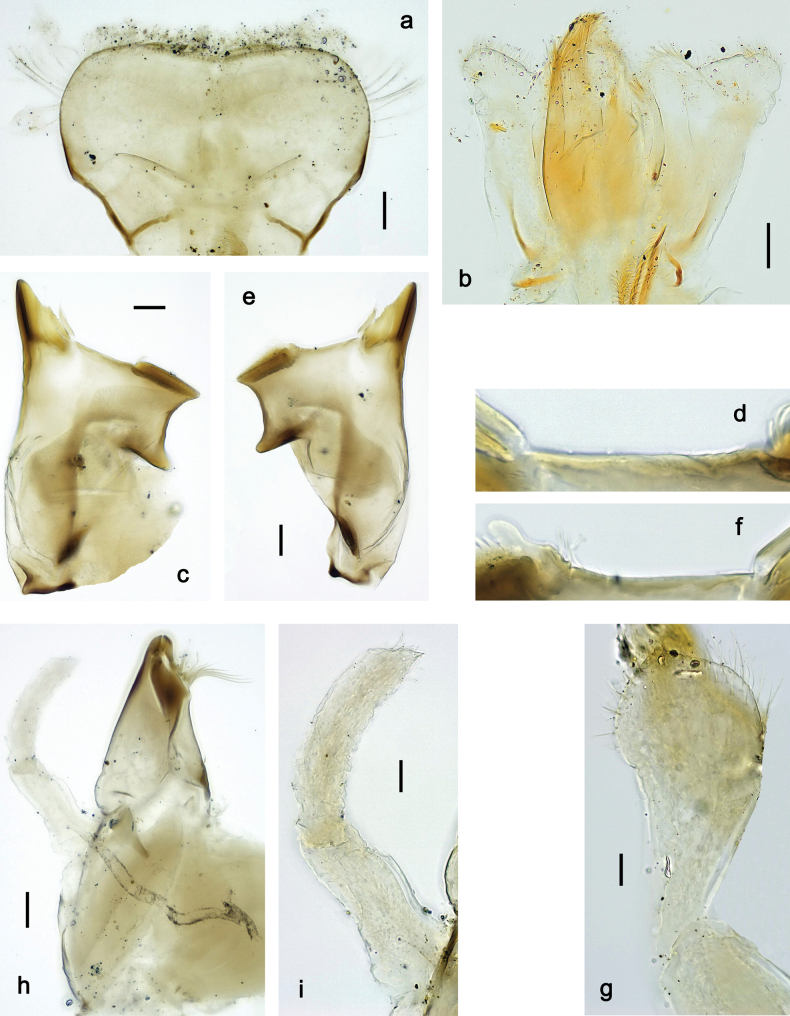
Papuanatula (Papuanatula) lenos, larva (type locality): **a** labrum **b** hypopharynx and superlinguae **c** right mandible **d** right mandible: margin between prostheca and mola **e** left mandible **f** left mandible: margin between prostheca and mola **g** labial palp **h** maxilla **i** maxillary palp. Scale bars: 20 µm (**a–c, e, h**); 10 µm (**g, i**).

**Figure 16.**
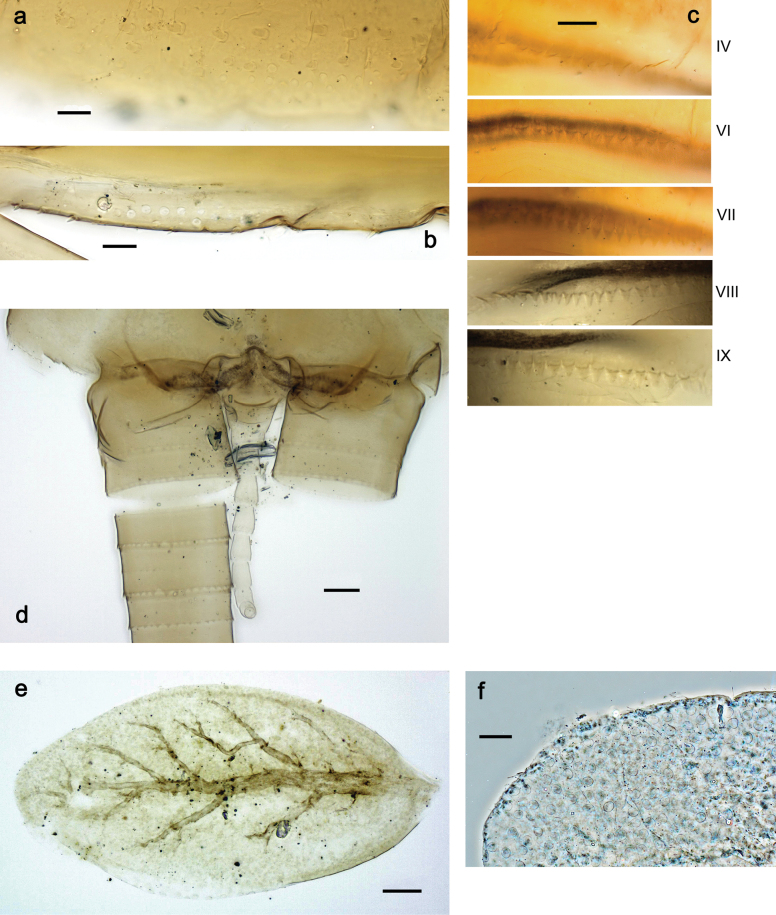
Papuanatula (Papuanatula) lenos, larva (type locality): **a** middle tibia, posterior surface **b** fore femur, ventral margin **c** abdominal terga **d** paracercus **e** tergalius II **f** tergalius IV, margin. Scale bars: 10 µm (**a–c, f**); 20 µm (**d, e**).

**Larva** (Figs 14–16; Lugo-Ortiz and McCafferty 1999: 65–66, figs 19–24). Body length 3.3–4.4 mm, cerci length unknown.

***Cuticular coloration*** (Fig. 14a). Head, thorax and abdomen dorsally yellow-brown to brown with pattern as in Fig. 14a, slightly variable; abdominal segments III–VIII with oblique, dark brown lateral markings, partly forming a trough-shaped pattern (segments III, VII, and VIII), segment IV with dark brown crown-like pattern. Legs yellow-brown to brown; Caudalii yellow-brown.

***Hypodermal coloration*.** Abdominal segments I–IX dorsally with transverse stripe on posterior margins (Fig. 14a). Femur anteriorly with large oblong to drop-shaped, dark brown to purple black marking in basal 1/2; posteriorly with long, broad, dark brown to purple black dashes.

***Head*. *Antenna*.** Length 1.5× head length. ***Developing turbinate eyes in last instar male larva*** unknown. ***Labrum*** (Fig. 15a). Length 0.5× maximum width, laterally convex. Dorsal, sub-marginal arc with 12–15 feathered setae. ***Right mandible*** (Fig. 15c, d). Margin between prostheca and mola smooth. Otherwise, as typical for subgenus. ***Left mandible*** (Fig. 15e, f). Margin between prostheca and mola smooth, with one spine close to subtriangular process. Otherwise, as typical for subgenus. ***Hypopharynx*** (Fig. 15b). As typical for genus. ***Maxilla*** (Fig. 15h, i). Maxillary palp subequal in length to galea-lacinia; palp segment II subequal in length to segment I. Otherwise, as typical for genus. ***Labium*** (Fig. 15g). As typical for genus. Paraglossa dorsally with one spine-like seta near inner, distolateral margin. Labial palp with segment I subequal in length to segments II and III combined. Segment II without distomedial protuberance, dorsally with row of five spine-like setae near outer, distolateral margin. Segment III bulbous, pointed, 0.8× length of segment II; inner dorsal margin with few feathered setae.

***Thorax*. *Sterna*** without protuberances. ***Terga*** without protuberances. ***Legs*** (Fig. 16a, b). Ratio of leg segments: fore leg 1.0:1.0:0.2:0.1, middle leg 0.9:1.0:0.2:0.1 and hind leg 1.1:1.0:0.3:0.1. ***Femur*.** Length ~ 3× maximum width. ***Claw*** with one row of six or seven denticles, one posterior seta.

***Abdomen*. *Terga*** (Fig. 16c). Abdominal terga without protuberances. Posterior margin of terga: I smooth, without denticles; II–IX with triangular, pointed denticles. ***Tergalii*** (Fig. 16e–f) ovoid, tracheation rather poorly developed; margins smooth, with short, fine, simple setae. ***Paraproct***. Posterior margin without prolongation; smooth, without denticles. ***Caudalii*** (Fig. 16d). Paracercus with nine segments, immature larvae up to 11 segments.

***Pose of subimaginal gonostyli under larval cuticle*.** Unknown.

**Subimago.** Unknown.

**Imago.** Unknown.

**Egg.** Unknown.

#### Distribution.

New Guinea (Fig. 146).

### ﻿﻿ Papuanatula (Papuanatula) plana

Taxon classificationAnimaliaEphemeropteraBaetidae

Lugo-Ortiz & McCafferty, 1999

EF608808-121A-551A-880C-A064B7D9A843

Figs 17 , 18 , 19 , 20 , 21 , 22 , 23 , 24

Papuanatula plana . Lugo-Ortiz and McCafferty 1999: 66–68, figs 25–30.

#### Material examined.

**Type locality (‘additional material’ in original description).** Papua New Guinea • 5 larvae; Morobe Prov., E of Wau, Bulolo Riv.; 900 m; 15.x.1964; leg. WL and WG Peters; 3 in alcohol; GBIFCH00976075, GBIFCH00976051; 2 on slides; GBIFCH00592549, GBIFCH00592582; 22 male adults; 20 in alcohol; GBIFCH00976071, GBIFCH00976052; 2 on slides; GBIFCH00592550, GBIFCH01221756; MZL.

#### Diagnosis.

**Larva**. The following combination of characters distinguishes *P.plana* from other species of *Papuanatula* s. str.: body dorsally without row of long, fine, simple setae along midline; body dorsally without protuberances; femur without distinct markings; abdominal segment IV with dark brown, medioposterior mark; paracercus with 9 segments.

#### Description.

**Figure 17.**
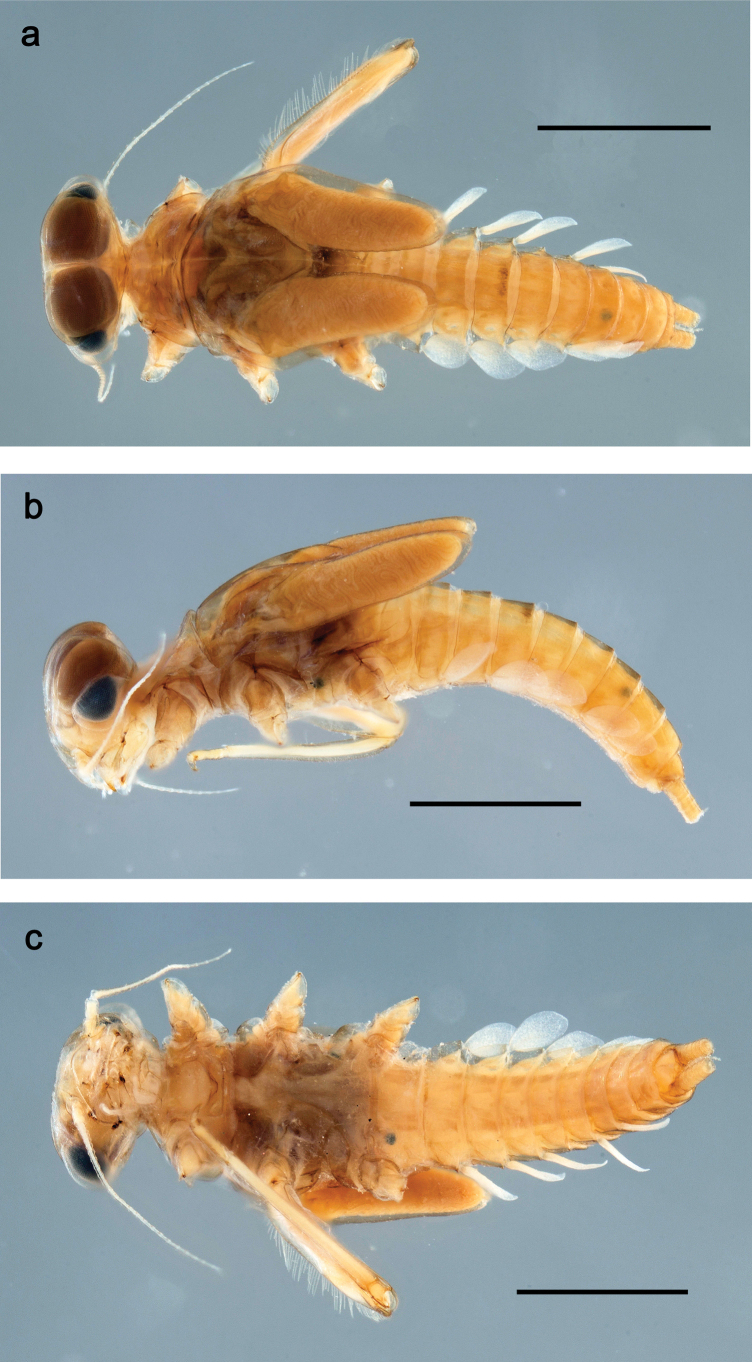
Papuanatula (Papuanatula) plana, larva, habitus (type locality): **a** dorsal view **b** lateral view **c** ventral view. Scale bars: 1 mm.

**Figure 18.**
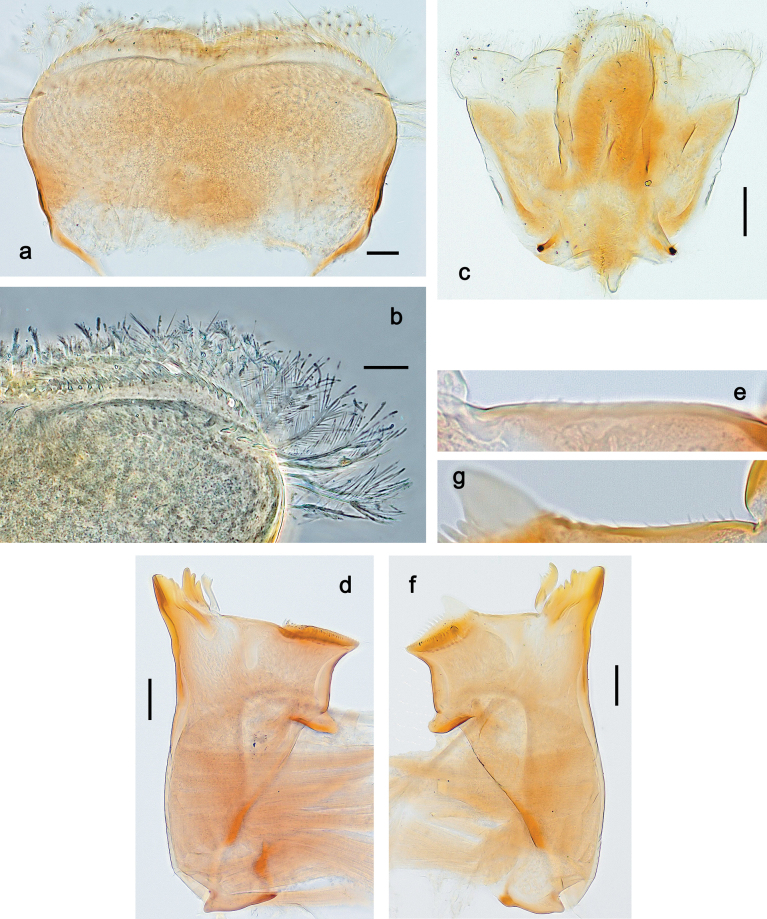
Papuanatula (Papuanatula) plana, larva (type locality): **a** labrum **b** labrum: dorsal, submarginal setae **c** hypopharynx and superlinguae **d** right mandible **e** right mandible: margin between prostheca and mola **f** left mandible **g** left mandible: margin between prostheca and mola. Scale bars: 10 µm (**a, b**); 20 µm (**c, d, f**).

**Figure 19.**
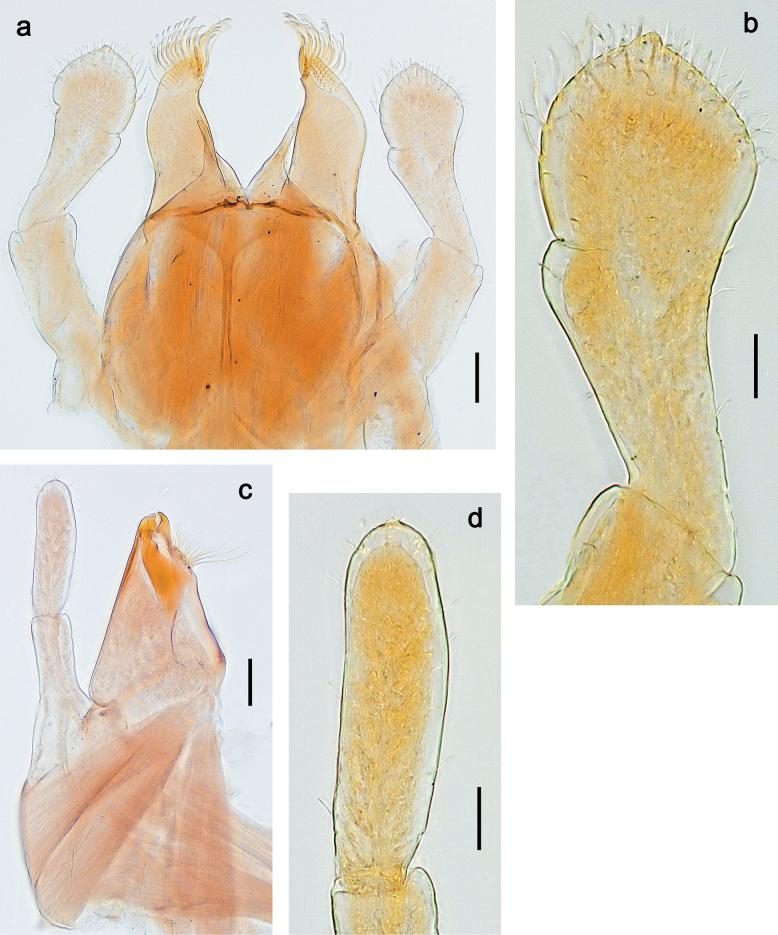
Papuanatula (Papuanatula) plana, larva (type locality): **a** labium **b** labial palp **c** maxilla **d** maxillary palp. Scale bars: 20 µm (**a, c**); 10 µm (**b, d**).

**Figure 20.**
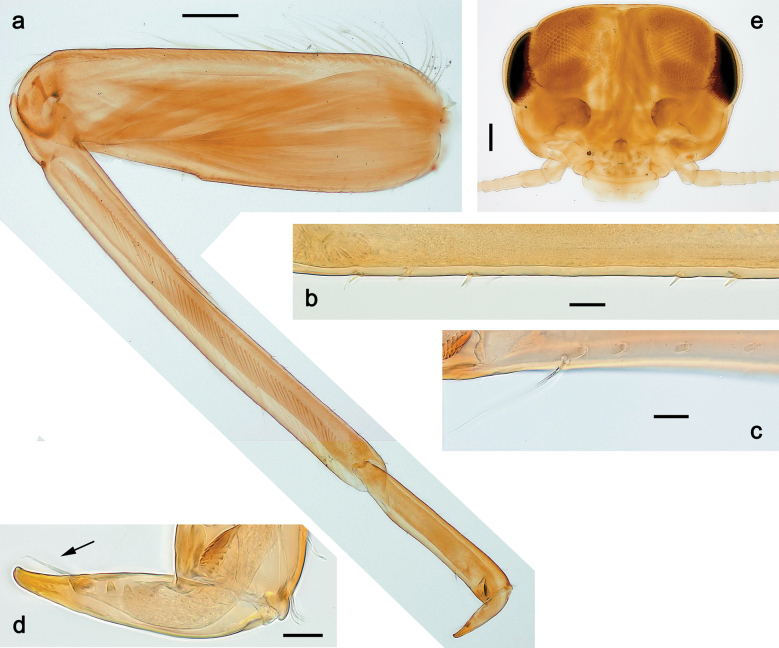
Papuanatula (Papuanatula) plana, larva (type locality): **a** foreleg **b** fore tibia, ventral margin **c** fore tarsus, ventral margin **d** fore claw (arrow: posterior seta) **e** male head, mature. Scale bars: 50 µm (**a, e**); 10 µm (**b, c, d**).

**Figure 21.**
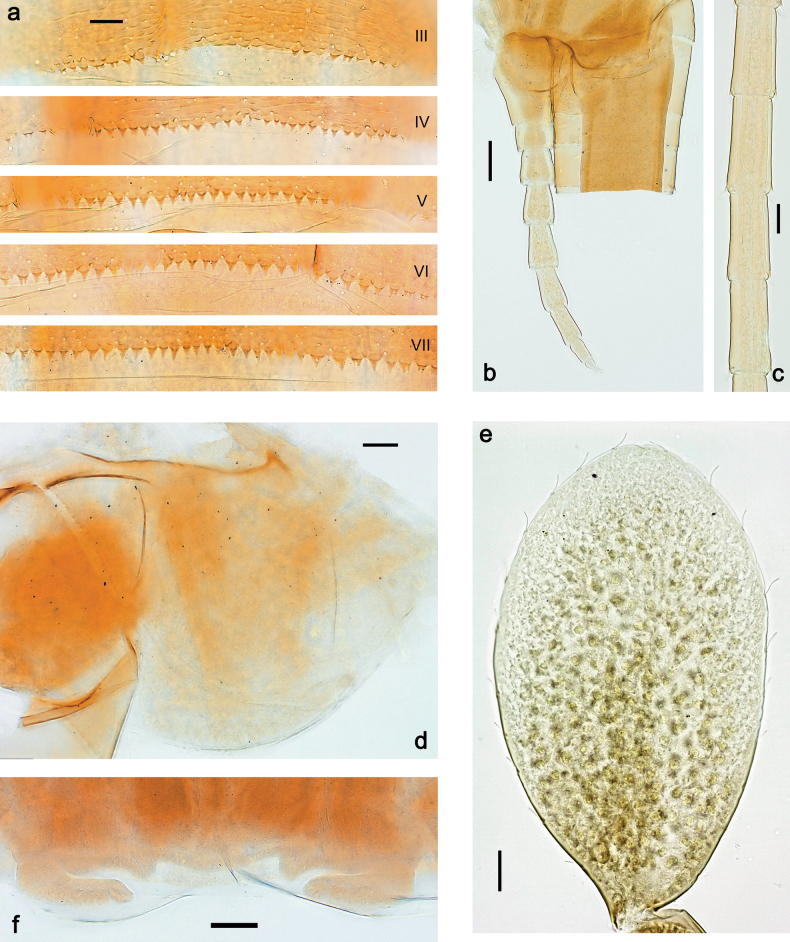
Papuanatula (Papuanatula) plana, larva (type locality): **a** abdominal terga **b** paracercus **c** cercus **d** paraproct **e** tergalius V **f** developing gonostyli. Scale bars: 10 µm (**a, c–e**); 20 µm (**b, f**).

**Figure 22.**
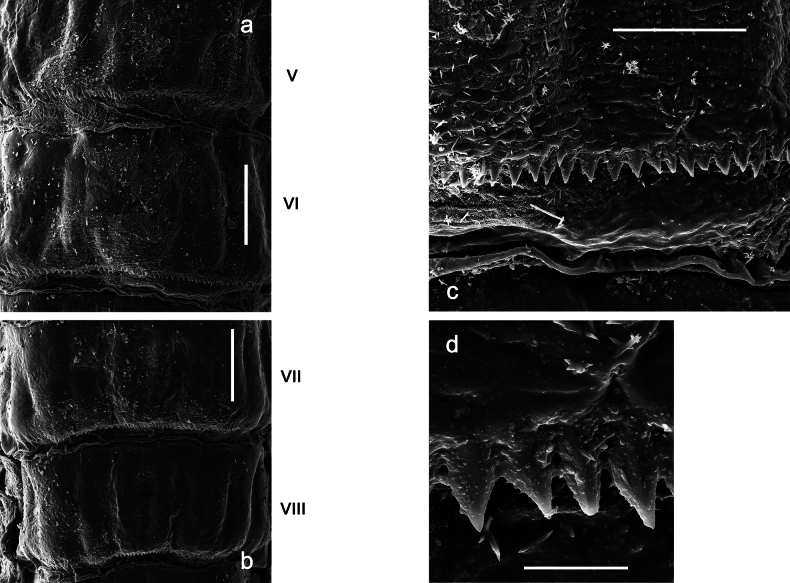
Papuanatula (Papuanatula) plana, larva, SEM: **a, b** abdominal terga **c** abdominal tergum VI **d** abdominal tergum VI. Scale bars: 100 µm (**a, b**); 40 µm (**c**); 10 µm (**d**).

**Larva** (Figs 17–22). Body length 3.7–4.1 mm, cerci much longer than body length.

***Coloration*** (Fig. 17a–c). Description see Lugo-Ortiz and McCafferty (1999: 66–67).

***Head*. *Antenna*** (Fig. 17a). Length 1.5× head length. As typical for the subgenus. ***Developing turbinate eyes in last instar male larva*** (Fig. 20e) large, subquadrangular, nearly touching each other in the middle. ***Labrum*** (Fig. 18a, b). Length 0.5× maximum width, laterally convex. Dorsal, sub-marginal arc with ~ 14 feathered setae. ***Right mandible*** (Fig. 18d, e). Margin between prostheca and mola with row of minute denticles. Otherwise, as typical for subgenus. ***Left mandible*** (Fig. 18f, g). Margin between prostheca and mola with row of minute denticles. Otherwise, as typical for subgenus. ***Hypopharynx*** (Fig. 18c). As typical for genus. ***Maxilla*** (Fig. 19c, d). Maxillary palp slightly longer than galea-lacinia, robust; palp segment II subequal in length to segment I. Otherwise, as typical for genus. ***Labium*** (Fig. 19a, b). As typical for the genus. Paraglossa dorsally with two spine-like setae near inner, distolateral margin. Labial palp with segment I subequal in length to segments II and III combined. Segment II with slight, broadly rounded, distomedial protuberance, dorsally with row of three spine-like setae near outer, distolateral margin. Segment III slightly pentagonal, pointed, 0.8× length of segment II.

***Thorax*. *Sterna*.** With small protuberances on sides of prosternum and close to openings of mesothoracic and metathoracic sternal apodemes (as in Fig. 108a). ***Terga*** without protuberances. ***Legs*** (Fig. 20a–d). Ratio of leg segments: fore leg 0.9:1.0:0.4:0.2, middle leg 0.9:1.0:0.3:0.2 and hind leg 1.0:1.0:0.4:0.2. ***Femur***. Length ~ 3× maximum width. ***Claw*** with one row of 5–8 denticles, distalmost denticle with distance to other denticles; one posterior seta.

***Abdomen*. *Terga*** (Figs 21a, 22a–d). Abdominal terga without protuberances. Posterior margin of terga: I and II smooth, without denticles; III–IX with triangular, pointed denticles, increasing in length toward VII. Surface with scattered small, sub rectangular, apically rounded scales. ***Tergalii*** (Fig. 21e). Narrow-elongate, tracheation absent or poorly developed; margins smooth, with short, fine, simple setae. ***Paraproct*** (Fig. 21d). Posterior margin without prolongation, smooth. ***Caudalii*** (Fig. 21b, c). Cerci without swimming setae. Paracercus with nine segments.

***Pose of subimaginal gonostyli under larval cuticle*** (Fig. 21f). As typical for the subgenus. Segment III conical.

**Subimago.** Unknown.

**Male and female imagos.** See description in Lugo-Ortiz and McCafferty (1999: 67, figs 31, 32).

**Figure 23.**
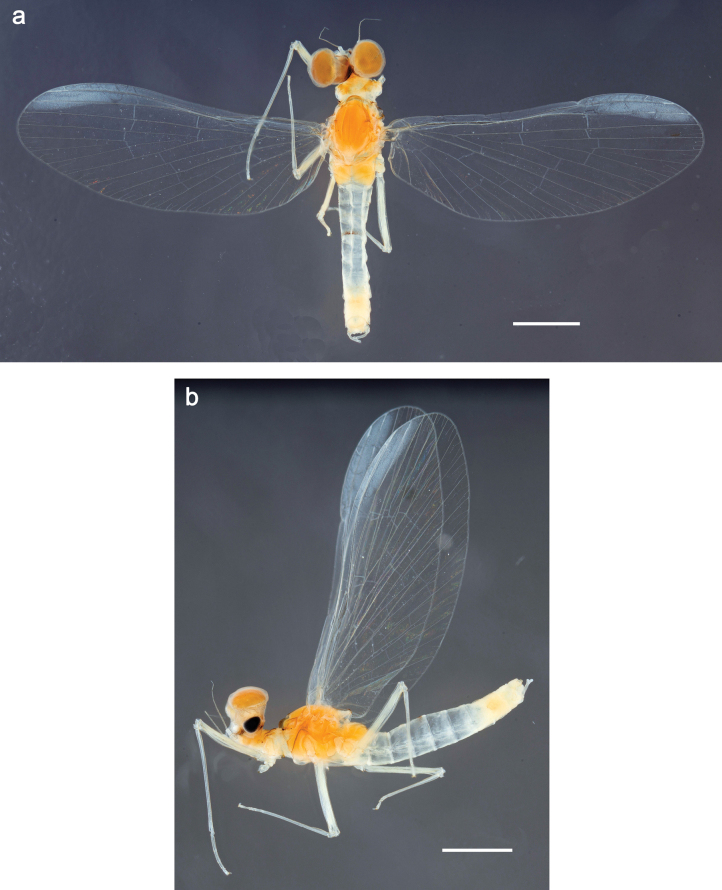
Papuanatula (Papuanatula) plana, male imago, habitus (type locality): **a** dorsal view **b** lateral view. Scale bars: 1 mm.

**Figure 24.**
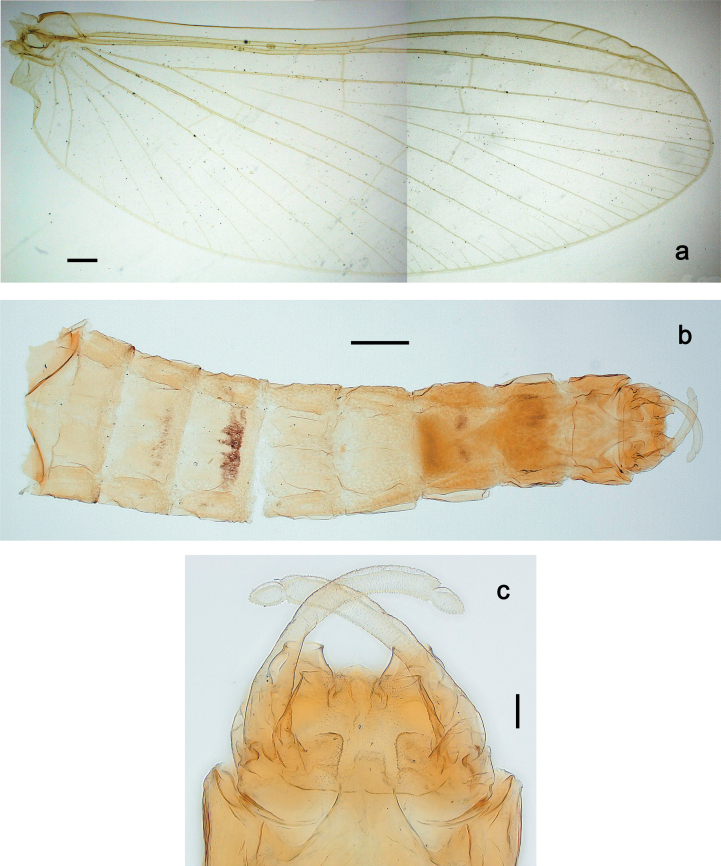
Papuanatula (Papuanatula) plana, male imago (type locality): **a** fore wing **b** abdomen **c** genitalia. Scale bars: 100 µm (**a, b**); 20 µm (**c**).

***Imago, male*** (Figs 23a, b, 24a, b). Head and thorax yellow-brown; legs pale yellow-brown. Turbinate eyes widened apically. Fore wings marginally with double intercalary veins. Pterostigma with four mostly incomplete, oblique cross veins. Hind wings absent. Abdominal segments I–VI translucent, abdominal tergum IV posteromedially with dark brown marking similar to larvae; abdominal segments VII–X pale yellow-brown.

***Genitalia*** (Fig. 24c). Sterno-styligeral muscle absent. Each unistyliger parallel-sided, equally wide at base and at apex. Gonostylus 1^st^ segment roundly-convex at apex, gradually turning to 2^nd^ segment. Second segment equally wide along its length. Third (terminal) segment of gonostylus nearly as wide as 2^nd^ segment, with length ~ 1.5× width. Penial bridge with slightly truncated trapezoid projection between unistyligers.

**Egg.** Unknown.

#### Comparison.

The most similar species is *P.obscurella* sp. nov., a detailed comparison is given under this species.

#### Distribution.

New Guinea (Fig. 146).

### ﻿﻿ Papuanatula (Papuanatula) tuber

Taxon classificationAnimaliaEphemeropteraBaetidae

Lugo-Ortiz & McCafferty, 1999

F4E32978-8935-5B8F-9D0B-CA0E10E01329

Fig. 25a, b

Papuanatula tuber . Lugo-Ortiz and McCafferty 1999: 68–69, figs 33–35.

#### Material examined.

***Holotype*.** Papua New Guinea • larva; Morobe Prov., E of Wau, Bulolo Riv.; 900 m; 15.x.1964; leg. W.L. and J.G. Peters; (photos of undissected larva).

#### Diagnosis.

**Larva.** The following combination of characters distinguishes *P.tuber* from other species of *Papuanatula* s. str.: body dorsally without row of long, fine, simple setae along midline; abdominal terga I–VIII (IX) with short, stout, dorsally oriented, medial protuberances; pronotum with paired, medioposterior protuberances; femur without distinct markings; paracercus vestigial; body size 2.7–3.4 mm.

#### Description.

**Figure 25.**
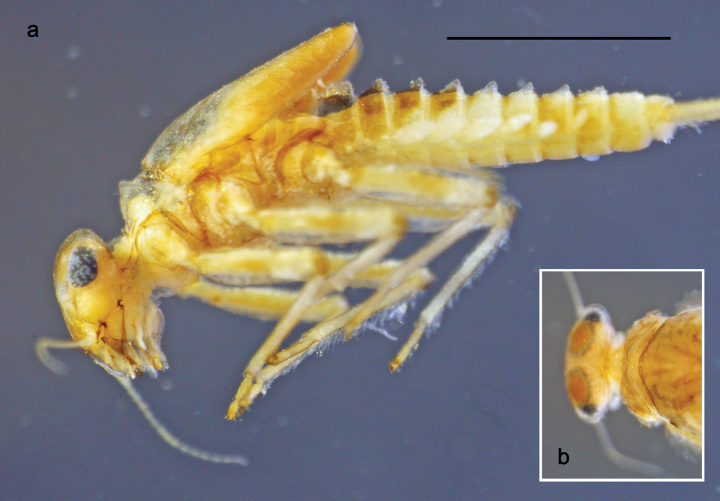
Papuanatula (Papuanatula) tuber, larva, habitus (holotype): **a** lateral view **b** male head, mature. Scale bar: 1 mm; (photographs C. C. Wirth, Purdue University).

**Larva** (Fig. 25a, b). See Lugo-Ortiz and McCafferty (1999: 68–69, figs 33–35).

**Subimago.** Unknown.

**Imago.** Unknown.

**Egg.** Unknown.

#### Distribution.

New Guinea (Fig. 146).

### ﻿﻿ Papuanatula (Papuanatula) vaisisi

Taxon classificationAnimaliaEphemeropteraBaetidae

Lugo-Ortiz & McCafferty, 1999

90E494EE-A5B6-5BCE-963A-9208928A618A

Papuanatula vaisisi . Lugo-Ortiz and McCafferty 1999: 69, figs 36, 37.
Pseudocloeon sp. 2. Demoulin 1969: 229–230, fig. 4a–i.

#### Diagnosis.

**Larva**. The following combination of characters distinguishes *P.vaisisi* from other species of *Papuanatula* s. str.: body dorsally without row of long, fine, simple setae along midline; abdominal terga without protuberances; femur with brown, triangular mark in posterior 1/2; maxillary palp robust, shorter than galea-lacinia; labial palp segment III with poorly developed lateral convexity.

#### Description.

**Larva.** See Lugo-Ortiz and McCafferty (1999: 69, figs 33–35) and Demoulin (1969: 229–230, fig. 4a–i).

**Subimago.** Unknown.

**Imago.** Unknown.

**Egg.** Unknown.

#### Distribution.

New Britain.

### ﻿﻿ Papuanatula (Papuanatula) balkei sp. nov.

Taxon classificationAnimaliaEphemeropteraBaetidae

A8CB813B-5D5B-586E-BAB1-3E874CF478B4

https://zoobank.org/C28B143D-DB51-4F86-8BB5-D41ADF068344

**Figure 26.**
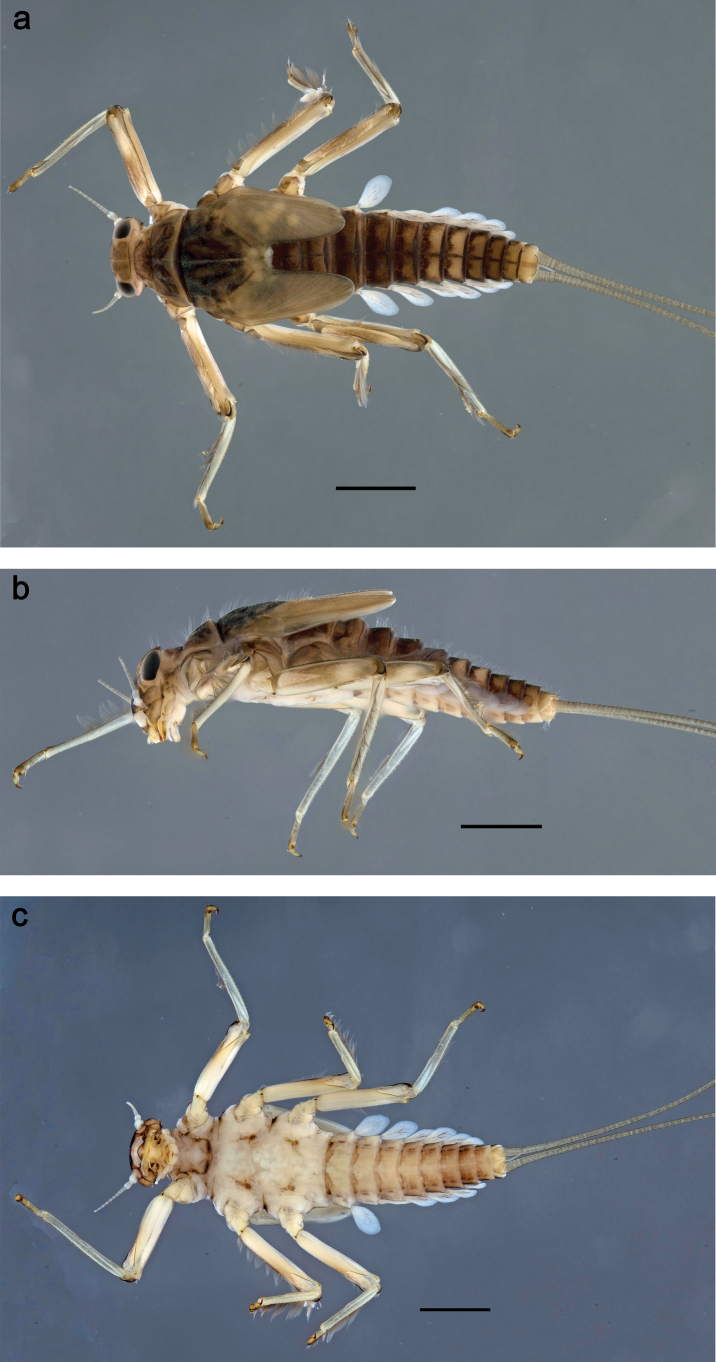
Papuanatula (Papuanatula) balkei sp. nov., larva, habitus: **a** dorsal view **b** lateral view **c** ventral view. Scale bars: 1 mm.

**Figure 27.**
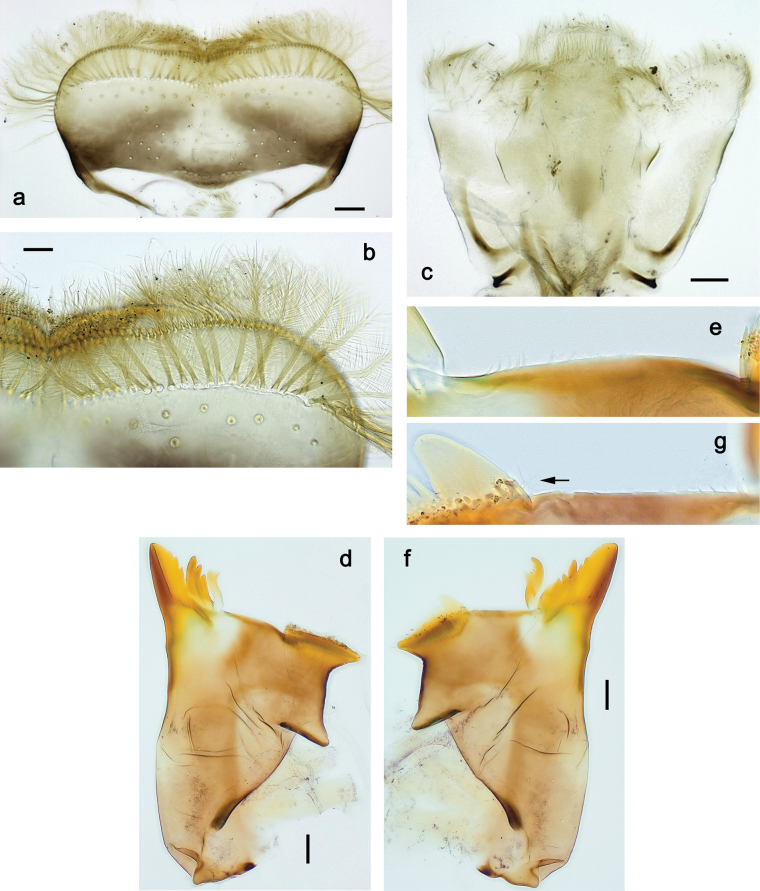
Papuanatula (Papuanatula) balkei sp. nov., larva: **a** labrum **b** labrum: dorsal submarginal setae **c** hypopharynx and superlinguae **d** right mandible **e** right mandible: margin between prostheca and mola **f** left mandible **g** left mandible: margin between prostheca and mola. Scale bars: 20 µm (**a, c, d, f**); 10 µm (**b**).

**Figure 28.**
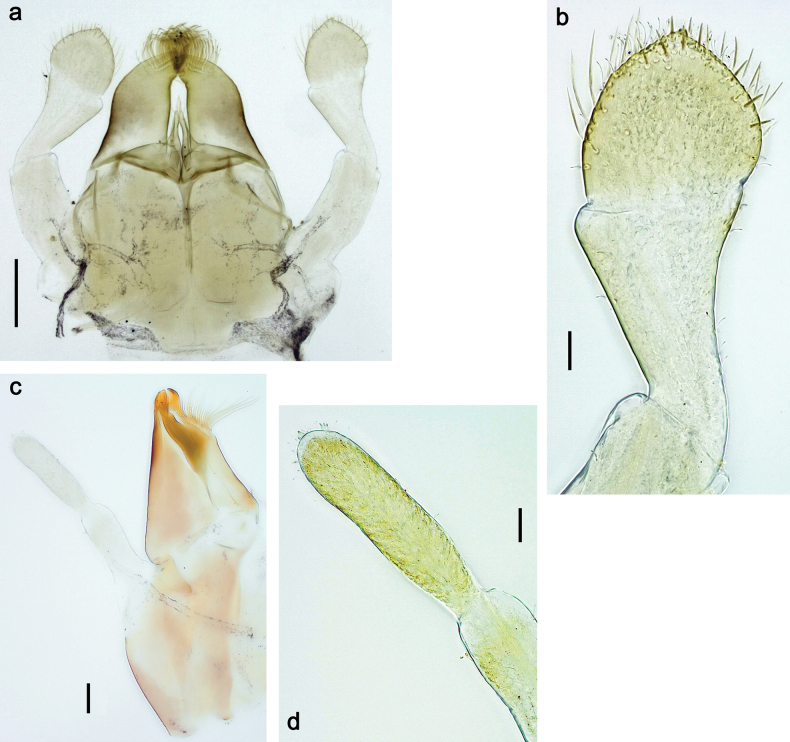
Papuanatula (Papuanatula) balkei sp. nov., larva: **a** labium **b** labial palp **c** maxilla **d** maxillary palp. Scale bars: 50 µm (**a**); 10 µm (**b, d**); 20 µm (**c**).

**Figure 29.**
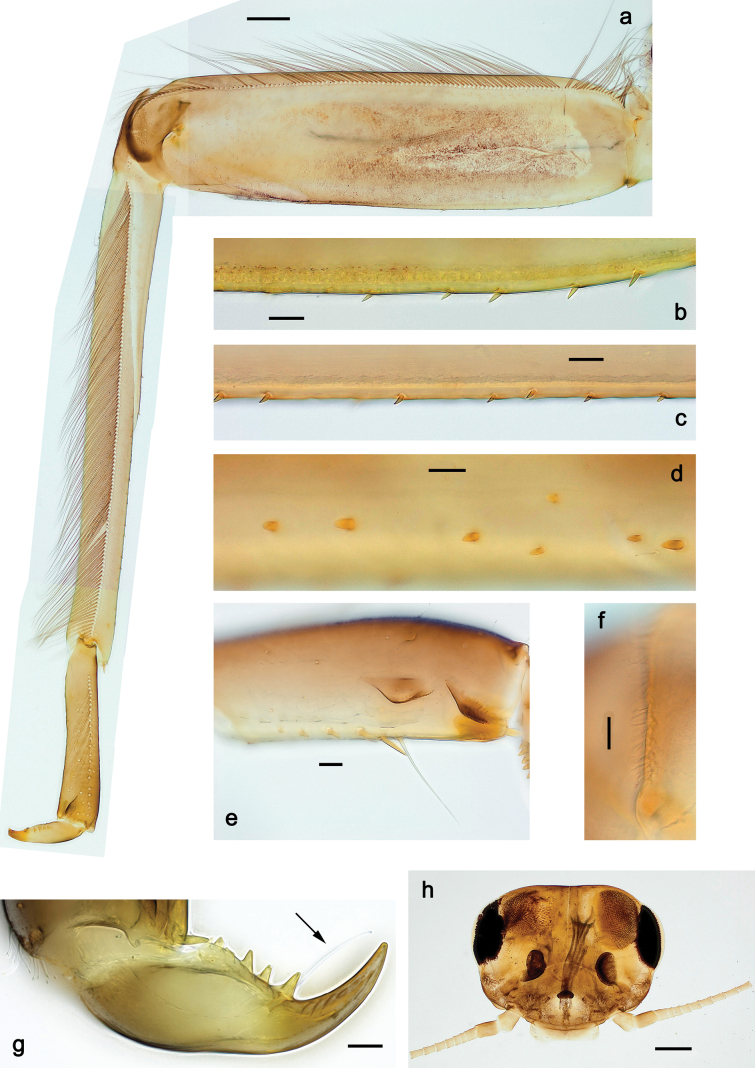
Papuanatula (Papuanatula) balkei sp. nov., larva: **a** middle leg **b** middle femur, ventral margin **c** middle tibia, ventral margin **d** middle tibia, posterior surface **e** middle tarsus, ventral margin **f** middle femur, posterior apex **g** fore claw (arrow: posterior seta) **h** male head, mature. Scale bars: 50 µm (**a**); 10 µm (**b–g**); 100 µm (**h**).

**Figure 30.**
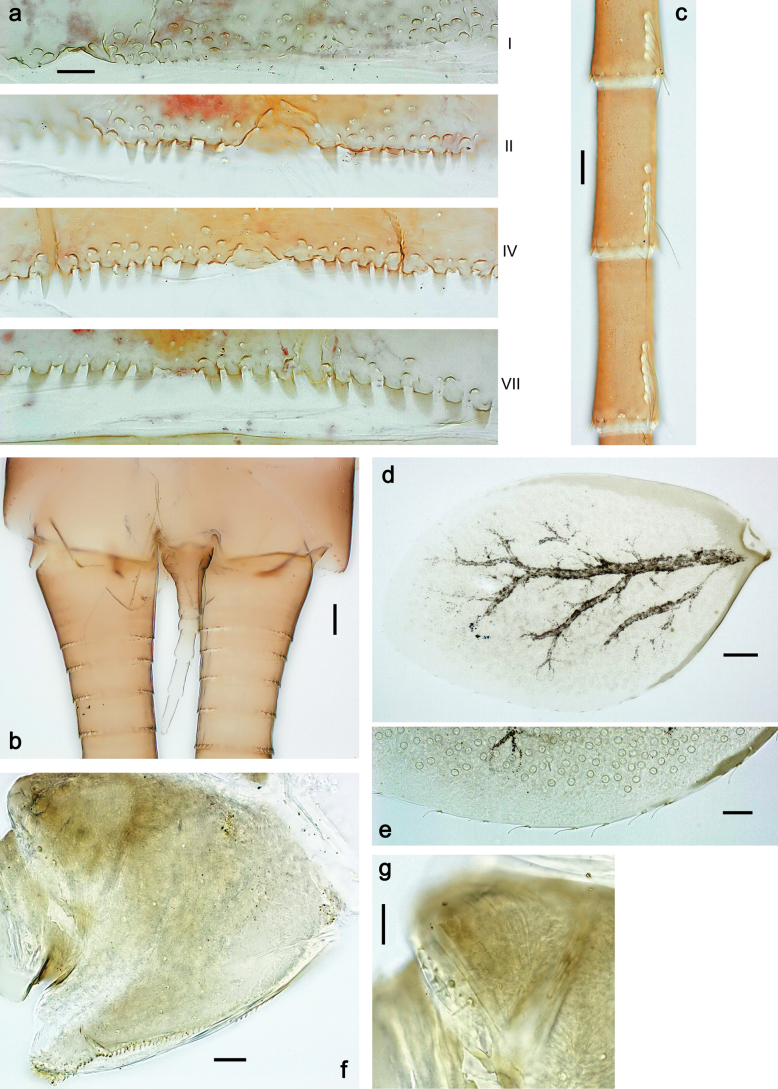
Papuanatula (Papuanatula) balkei sp. nov., larva: **a** abdominal terga **b** paracercus **c** cercus **d** tergalius IV **e** tergalius IV, margin **f** paraproct **g** cercotractor. Scale bars: 10 µm (**a, c, e–g**); 20 µm (**b, d**).

**Figure 31.**
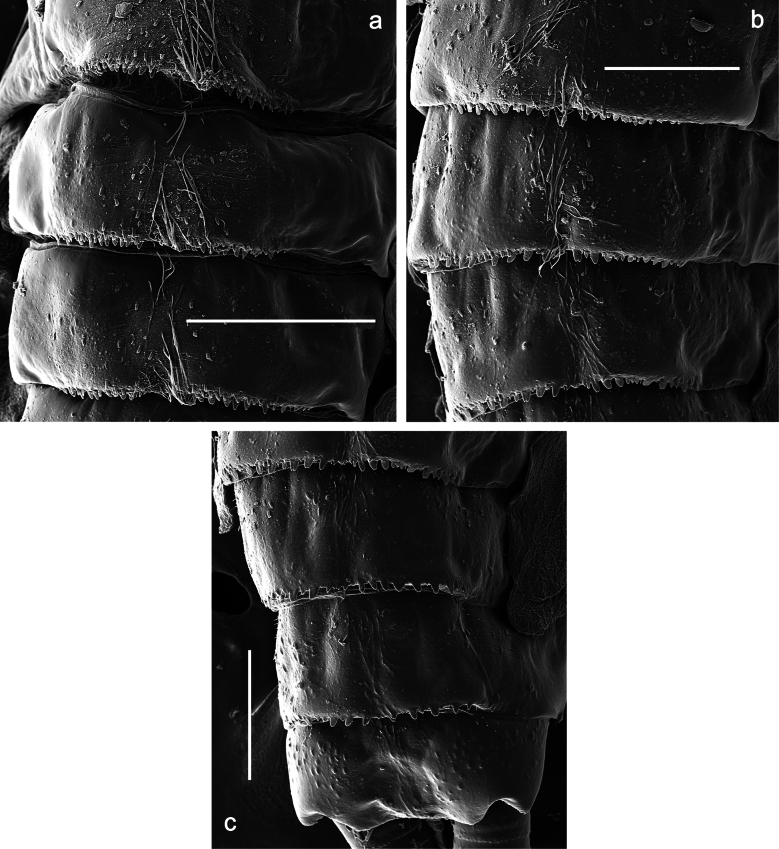
Papuanatula (Papuanatula) balkei sp. nov., larva (SEM): **a** abdominal terga II–IV **b** abdominal terga V–VII **c** abdominal terga VII–X. Scale bars: 300 µm (**a**); 200 µm (**b, c**).

Figs 26 , 27 , 28 , 29 , 30 , 31

#### Etymology.

The species is dedicated to Michael Balke (ZSM/SNSB), the collector of an important part of the material used in this study.

#### Material examined.

***Holotype*.** Papua New Guinea • larva; Eastern Highlands Prov., Marawaka, Ande; near 07°01'42"S, 145°49'48"E; 1700–1800 m, 9.xi.2006, leg. M. Balke & Kinibel; (PNG 87); on slide; GBIFCH00592545, GBIFCH00592547; ZSM/SNSB. ***Paratypes*.** 20 larvae; same data as holotype; 5 on slides; GBIFCH00592544, GBIFCH00592546, GBIFCH00592626, GBIFCH00975797, GBIFCH00975798, GBIFCH00976040; MZL; 15 in alcohol; GBIFCH00975770, GBIFCH00976050, GBIFCH00976108, GBIFCH00976141; MZL.

#### Other material.

Papua New Guinea • 26 larvae; Western Highlands Prov., Simbai; 05°16'20"S, 144°33'11"E; 1800–2000 m; 25.ii.2007; leg. Kinibel; (PNG 133); 2 on slides; GBIFCH00976049, GBIFCH00976095; MZL; 24 in alcohol; GBIFCH00975786, GBIFCH00976043, GBIFCH00976096, GBIFCH00976097; MZL.

#### Diagnosis.

**Larva.** The following combination of characters distinguishes *P.balkei* sp. nov. from other species of *Papuanatula* s. str.: body dorsally with irregular row of long, fine, simple setae along midline; abdominal terga without protuberances; femur proximally with wedge-shaped blank, overlaid with scattered brown color; abdominal terga brown, laterally darker, terga V, VI, and X brighter; tergalii with pigmented tracheation; paracercus with seven or eight segments; abdominal terga with triangular, apically rounded denticles on posterior margin; small scattered scales on abdominal terga elongate, slightly trapezoid.

#### Description.

**Larva** (Figs 26–31). Body length 4.2–5.6 mm, cerci much longer than body length (~ 1.5×).

***Cuticular coloration*** (Fig. 26a–c). Head, thorax and abdomen dorsally brown; thorax with complex pattern; abdominal segments laterally darker, V, VI, and X brighter. Femur proximally with wedge-shaped blank, overlaid with scattered brown color; medial area grey-brown, distal area yellow-brown to grey-brown; tibia grey; tarsus grey, distally brown. Head, thorax and abdominal segment I ventrally ecru, protuberances of thoracic sterna brown; abdominal segments II–X ventrally pale brown, darker toward end of abdomen. Cerci grey-brown.

***Hypodermal coloration*.** Each abdominal tergum I–IX with wide dark brown transverse band close to anterior margin and with narrower dark brown transverse band close to posterior margin (Fig. 26a).

***Head*** (Figs 26b, 29h). Dorsally with irregular row of long, fine, simple setae along midline. ***Antenna***. Length ~ 1.5× head length. Otherwise, as typical for subgenus. ***Developing turbinate eyes in last instar male larva*** (Fig. 29h) ovoid, with large distance to each other. ***Labrum*** (Fig. 27a, b). Length 0.5× maximum width, laterally convex. Dorsal, sub-marginal arc with 24–29 feathered setae. ***Right mandible*** (Fig. 27d, e). Margin between prostheca and mola with row of minute denticles. Otherwise, as typical for subgenus. ***Left mandible*** (Fig. 27f, g). Margin between prostheca and mola with row of minute denticles. Otherwise, as typical for subgenus. ***Hypopharynx*** (Fig. 27c). As typical genus. ***Maxilla*** (Fig. 28c, d). Maxillary palp subequal in length to galea-lacinia; palp segment II slightly longer than segment I. Otherwise, as typical for genus. ***Labium*** (Fig. 28a, b). As typical for genus. Paraglossa dorsally with two spine-like setae near inner, distolateral margin. Labial palp with segment I subequal in length to segments II and III combined. Segment II with minute distomedial protuberance, dorsally with row of four spine-like setae near outer, distolateral margin. Segment III slightly pentagonal, pointed; 0.7× length of segment II; inner dorsal margin with few feathered setae.

***Thorax*. *Sterna***. With small protuberances on sides of prosternum and close to openings of mesothoracic and metathoracic sternal apodemes (as in Fig. 108a). ***Terga*** (Fig. 26b) without protuberances; with irregular row of long, fine, simple setae along midline. Metanotum without hind protoptera or their vestiges. ***Legs*** (Fig. 29a–g). Ratio of leg segments: fore leg 0.8:1.0:0.4:0.1, middle leg 0.9:1.0:0.3:0.1 and hind leg 1.0:1.0:0.4:0.2. ***Femur***. Length ~ 3.7× maximum width. ***Claw*** with one row of 5–7 denticles and one or sometimes two posterior setae.

***Abdomen*. *Terga*** (Figs 30a, 31a–c) with irregular row of long, fine, simple setae along midline. Terga without protuberances, terga I–IV with slight, paired medioposterior elevations. Posterior margin of terga: I with rudimentary denticles, II–IX with triangular, apically rounded denticles. Surface with scattered small, elongate, slightly trapezoid, striated scales. ***Tergalii*** (Fig. 30d, e). Broad ovoid, tracheation well pigmented; margins smooth, with few short, fine, simple setae. ***Paraproct*** (Fig. 30f). Posterior margin with prolongation and row of minute denticles. ***Caudalii*** (Fig. 30b, c). Cerci apart from basal and distal part with 1–7 swimming setae per segment, initially increasing and then again decreasing toward distal part. Paracercus with seven or eight segments.

***Pose of subimaginal gonostyli under larval cuticle*.** Unknown.

**Subimago.** Unknown.

**Imago.** Unknown.

**Egg.** Unknown.

#### Distribution.

New Guinea (Fig. 146).

### ﻿﻿ Papuanatula (Papuanatula) cyclopomontana sp. nov.

Taxon classificationAnimaliaEphemeropteraBaetidae

835CD3DA-6248-5733-9328-0208AFAD25D1

https://zoobank.org/8C0BE9DD-584F-4667-861B-56A64F957C56

**Figure 32.**
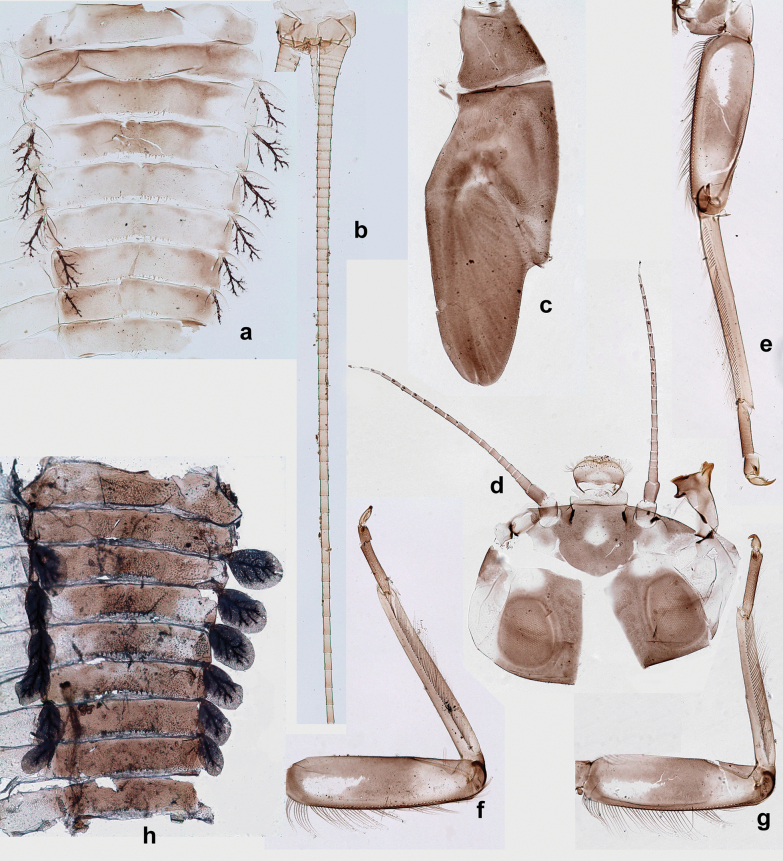
Papuanatula (Papuanatula) cyclopomontana sp. nov., larval exuviae (in one magnification): **a** abdominal segments I–IX **b** segment X and caudalii **c** pronotum and mesonotum **d** head **e–g** fore, middle, and hind legs **h** abdominal segments I–IX (dry) (c–g holotype).

**Figure 33.**
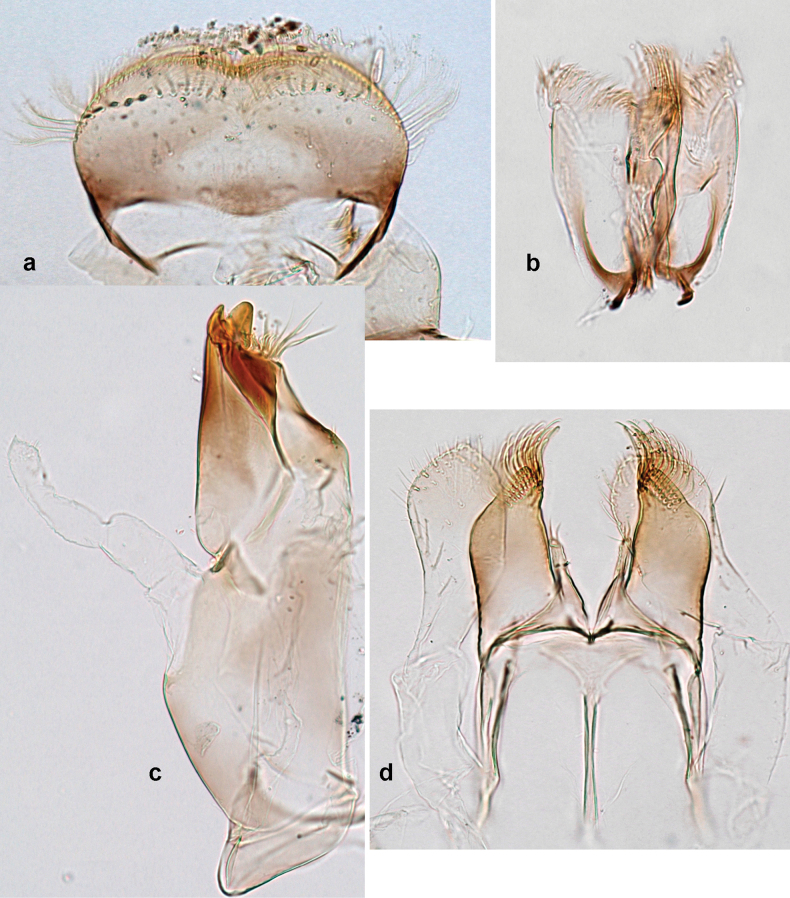
Papuanatula (Papuanatula) cyclopomontana sp. nov., larval exuviae (holotype): **a** labrum **b** hypopharynx **c** maxilla **d** labium.

**Figure 34.**
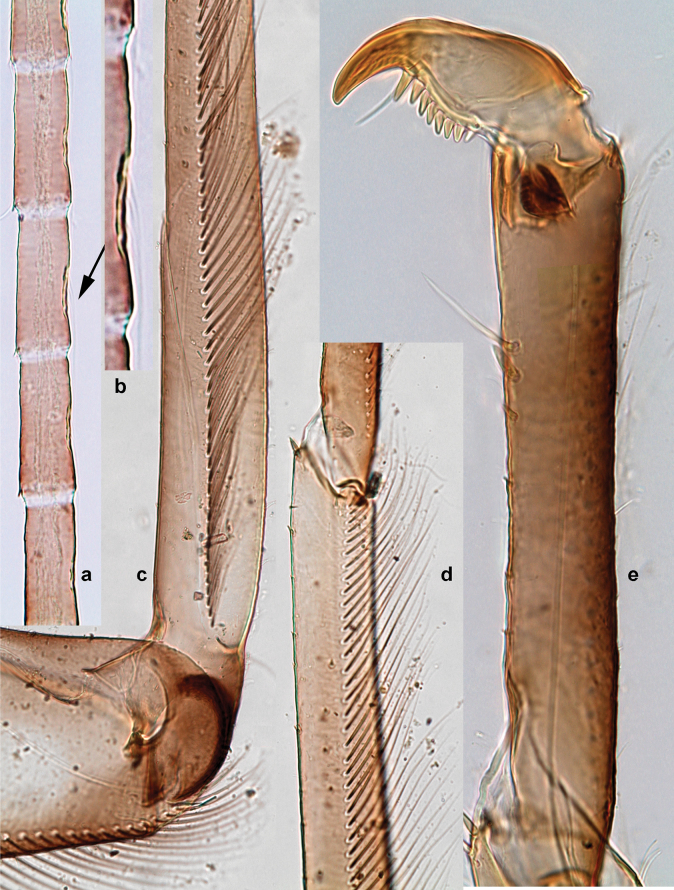
Papuanatula (Papuanatula) cyclopomontana sp. nov., larval exuviae (holotype): **a, b** fragment of cercus **c** apex of femur and base of tibia (middle leg) **d** apex of tibia and base of tarsus (fore leg) **e** tarsus and claw (fore leg).

**Figure 35.**
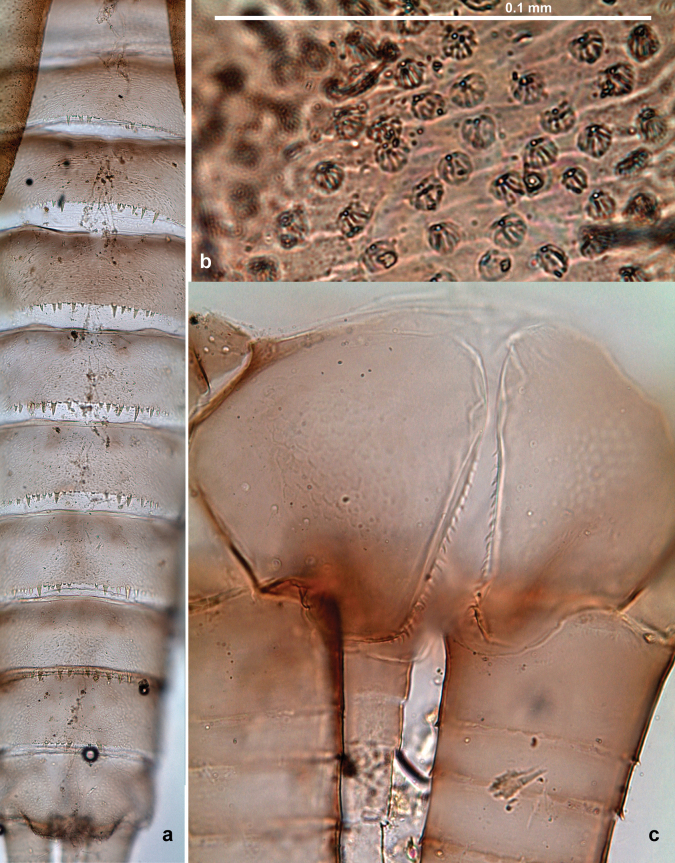
Papuanatula (Papuanatula) cyclopomontana sp. nov., larval exuviae: **a** abdominal terga **b** abdominal tergum III **c** paraprocts (**b, c** holotype).

**Figure 36.**
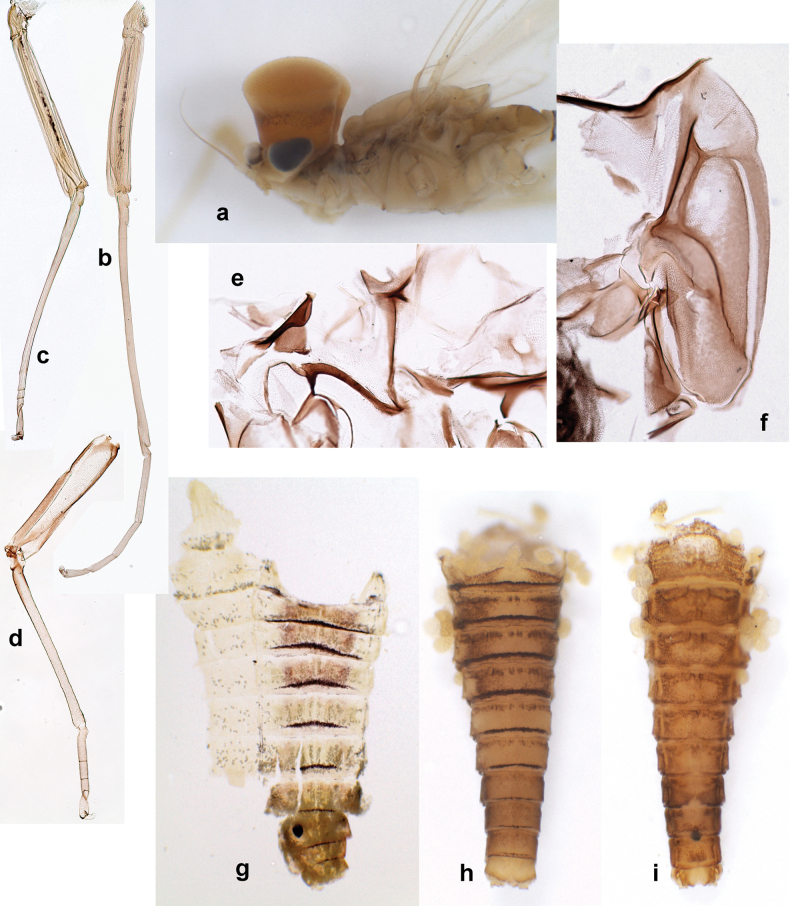
Papuanatula (Papuanatula) cyclopomontana sp. nov. **a** head and thorax of male imago **b, c** fore and middle legs of male imago **d** subimaginal exuviae of fore leg **e** subimaginal exuviae of mesopleuron **f** subimaginal exuviae of mesonotum **g** abdomen of male imago **h, i** abdomen of female subimago, dorsally and ventrally (**a–g** holotype).

**Figure 37.**
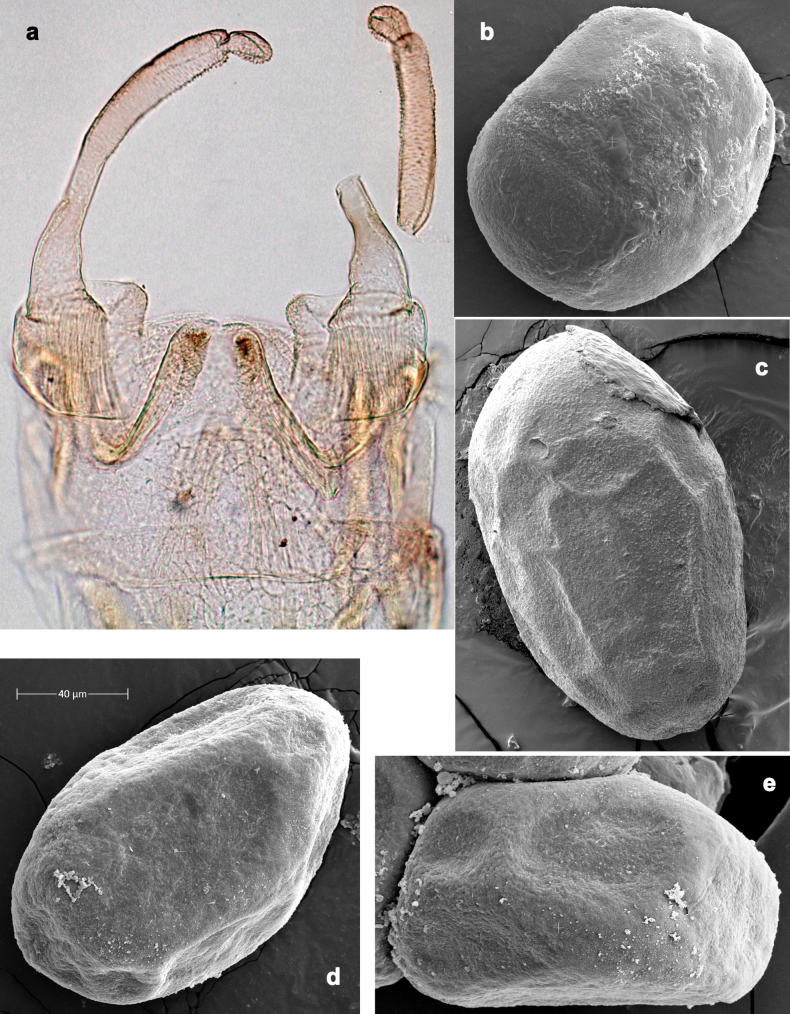
**a**Papuanatula (Papuanatula) cyclopomontana sp. nov., genitalia (holotype) **b–e** eggs of subgenus Papuanatula: **b**Papuanatula (Papuanatula) cyclopomontana sp. nov. (extracted from subimago) **c**Papuanatula (Papuanatula) obscurella sp. nov. (extracted from imago) **d, e**Papuanatula (Papuanatula) obscura sp. nov. (extracted from imago).

Figs 32 , 33 , 34 , 35 , 36 , 37

#### Etymology.

The species name *cyclopomontana* refers to Cyclops Mountain, at which foot this species was collected.

#### Material examined.

***Holotype*.** L-S-I♂ {specimen number [XX](5)B2012}; Indonesia • Papua, Depapre; 28.viii.2012; coll. N. Kluge & L. Sheyko; SPbU. ***Paratypes*.** Same data as holotype, 2 L-S♀; SPbU.

#### Diagnosis.

**Larva.** The following combination of characters distinguishes *P.cyclopomontana* sp. nov. from other species of *Papuanatula* s. str.: body dorsally with irregular row of long, fine, simple setae along midline; abdominal terga without protuberances; paraglossa with three straight setal rows (not bent at apex of paraglossa); femur basally with wedge-shaped blank and less contrasting blank on distal ¹⁄3; tergalii with extensive, brown pigmentation; paracercus with 6–8 segments; abdominal terga II–IX with various denticles, from long and pointed to short and blunt.

#### Description.

**Larva** (Figs 33–35). ***Cuticular coloration*.** Head, pronotum, mesonotum and metanotum brownish, with darker and paler areas; fore protoptera nearly uniformly brown (Fig. 32c, d). Thoracic pleura brownish, sterna mostly colorless. Cuticle of femur mostly brownish, with clearly outlined wedge-shape blank on proximal ¹⁄3 and less contrasting blank on distal ¹⁄3; apex of femur bordered with darker brown (Fig. 32e–g). Tibia and tarsus mostly brownish (Fig. 32e–g). Abdominal terga either mostly brownish, or with brown anterior margin and paler remainder part; terga V and VI paler than others (Fig. 32a). Sterna mostly colorless. Cerci uniformly pale brownish.

***Hypodermal coloration*.** Judging by hypodermal coloration of male imago and female subimagines, legs without hypodermal markings; each abdominal tergum I–IX with dark brown transverse band close to posterior margin (Fig. 36b, c, g). Tissues surrounding tracheae of tergalii (main trachea and its branches) with extensive brown pigmentation (Fig. 32a).

***Head*.** Dorsally with irregular row of long, fine, simple setae along midline. ***Antenna*** (Fig. 32d). Length ~ 1.5× head length. As typical for subgenus. ***Developing turbinate eyes in last instar male larva*** (Fig. 32d) with facets equally developed on middle and peripheral areas. ***Labrum*** (Fig. 33a) widened distally; long, feathered setae on dorsal surface numerous and forming integral, regular transverse row. ***Right mandible***. As typical for subgenus. ***Left mandible***. As typical for subgenus. ***Hypopharynx*** (Fig. 33b). As typical for genus. ***Maxilla*** (Fig. 33c). Maxillary palp shorter than galea-lacinia. Otherwise, as typical for genus. ***Labium*** (Fig. 33d). Paraglossae with proximal 1/2 nearly parallel-sided; three apical setal rows straight (not bent at apex of paraglossa). Glossa as long as half of paraglossa, with finger-like (distal) portion as long as triangular (proximal) portion. Labial palp without distomedial projection on segment II; segment III with median margin longer than lateral margin.

***Thorax*. *Sterna*.** With small protuberances on sides of prosternum and close to openings of mesothoracic and metathoracic sternal apodemes (as Fig. 108a). ***Terga*** without protuberances. Long, fine, soft, colorless setae irregularly situated along midline of all terga (as in Fig. 65b). Metanotum without hind protoptera or their vestiges. ***Legs*** (Figs 32e–g, 34c–e). Fore femur widened in proximal part; hind tibia shorter than others. ***Femur*.** Outer side of each femur with single regular row of long, hair-like setae bearing numerous fine, short branches on all sides (as in Figs 41g, 68b). ***Tibia*.** Patella-tibial suture present on all legs, terminated near middle of inner margin of tibia. Tibia-tarsal condylus turned to anterior side. Anterior side of each tibia with regular row of setae similar to that on femur. ***Tarsus*.** Anterior side of each tarsus with regular row of similar, but smaller (shorter and narrower) setae. Posterior side of each tarsus with regular row of short, stout, oval setae (looking pointed in profile) and one much longer, thinner, pointed seta distad of them. ***Claw*** with row of 5–8 short denticles and one somewhat larger denticle distad of them; long, arched, posterior seta.

***Abdomen*. *Terga*** (Figs 32a, 35a, b). Long, fine, soft, colorless setae irregularly situated along midline of all abdominal terga (as in Fig. 65b). Abdominal terga without dorsal unpaired or paired protuberances, only with slightly expressed unpaired, median elevations. Abdominal terga with small, roundish scales with small sockets and radial striation (visible in dry condition, but not in Canada balsam). Posterior margins of abdominal terga II–IX with various denticles, from long and pointed to short and blunt, more numerous and long on middle terga, few and short on terga II and IX. Posterior margin of tergum X with smaller, blunt denticles. ***Tergalii*** (Fig. 32a, h) II–VII subequal, oval. Each tergalius with costal and anal ribs narrow, smooth, present on proximal 1/2 of tergalius only. ***Paraproct*** (Fig. 35c) with regular row of small, pointed, equal denticles on median margin, without posterior prolongation. ***Caudalii*** (Fig. 34a, b) without swimming setae; vestiges of swimming setae present on distal part of cerci. Paracercus short, consisting of ~ 6–8 segments.

***Pose of subimaginal gonostyli under larval cuticle*.** Unknown.

**Subimago. *Cuticular coloration***. Pronotum and prosternum partly brown (as in Fig. 60f). Mesonotum pale brown with medioparapsidal suture colorless, other sutures darker brown (Fig. 36f). Meso- and metathoracic pleura and sterna with colorless, pale brownish and dark brown areas (Fig. 36e). Cuticle of wings colorless, with microtrichiae brownish. Legs nearly colorless, with pale brown bordering on femur and base of tibia (Fig. 36d). Abdomen diffusely colored with very pale brownish in distal part.

***Hypodermal coloration*.** As in imago.

***Texture*.** On all legs of both sexes, each tarsomere covered mostly with blunt microlepides, with pointed microlepides near apex (as in Fig. 70i).

**Imago. *Imago, male*.** Head pale ochre. Antennae ochre. Turbinate eyes yellow, widened apically. Thorax ochre, equally pale dorsally, laterally, and ventrally. Fore wing with membrane colorless, veins ochre. Pterostigma with three or four incomplete, oblique cross veins. Legs ochre (Fig. 36b, c). Abdominal terga mostly ochre, terga II–IV with median part pale brown; each tergum I–IX with darker brown transverse band close to posterior margin; sterna ochre (Fig. 36g).

***Genitalia*** (Fig. 37). Sterno-styligeral muscle absent. Each unistyliger nearly equally wide at base and at apex, with median margin concave. At lateral side of gonostylus, its 1^st^ segment roundly-convex and separated from 2^nd^ segment by concavity; at median side of gonostylus, 1^st^ segment gradually turns to 2^nd^ segment. 2^nd^ segment equally wide all over its length. Third (terminal) segment of gonostylus nearly as wide as 2^nd^, with length slightly exceeding width. Penial bridge without projection between unistyligers. Gonovectes dark brown. Each gonovectis parabolic, with lateral (basal) and median (apical) portions equally long, apex bent medially.

***Imago, female*.** Head and thorax dorsally ochre-brownish. Abdomen mostly ochre, terga and sterna with brownish markings, each tergum I–IX with dark brown transverse band close to posterior margin (Fig. 36h, i). Coloration of legs, wings, and cerci as in male.

**Egg** (Fig. 37b). Irregularly oval. Chorion without regular relief.

#### Dimension.

Fore wing length (and approximate body length) of male and female 3.5 mm.

#### Distribution.

New Guinea (Fig. 146).

### ﻿﻿ Papuanatula (Papuanatula) dumspinae sp. nov.

Taxon classificationAnimaliaEphemeropteraBaetidae

77FD5C6C-0E19-5936-AA81-1122427BE6EA

https://zoobank.org/6B99D6FE-158E-4A8A-AE85-A386F4F202A8

**Figure 38.**
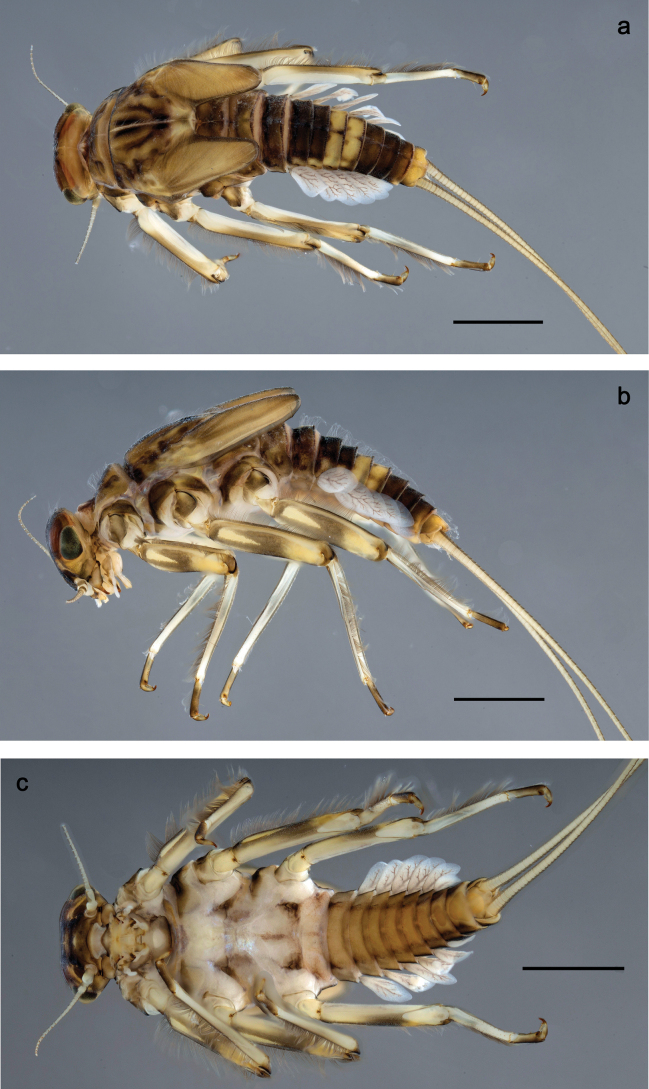
Papuanatula (Papuanatula) dumspinae sp. nov., larva, habitus: **a** dorsal view **b** lateral view **c** ventral view. Scale bars: 1 mm.

**Figure 39.**
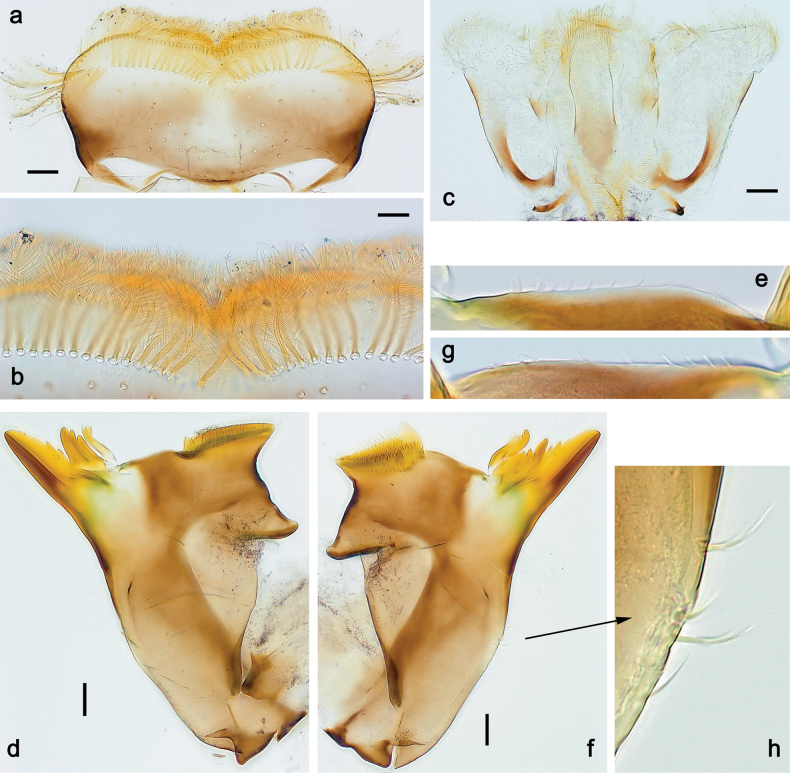
Papuanatula (Papuanatula) dumspinae sp. nov., larva: **a** labrum **b** labrum: dorsal submarginal setae **c** hypopharynx and superlinguae **d** right mandible **e** right mandible: margin between prostheca and mola **f** left mandible **g** left mandible: margin between prostheca and mola **h** left mandible: mediolateral setae. Scale bars: 20 µm (**a, c, d, f**); 10 µm (**b**).

**Figure 40.**
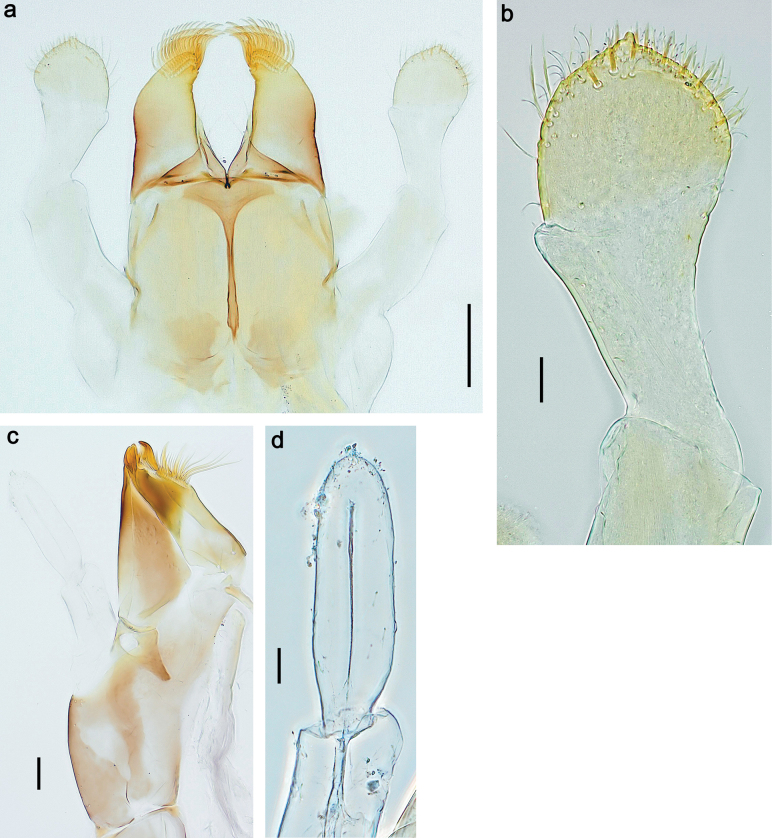
Papuanatula (Papuanatula) dumspinae sp. nov., larva: **a** labium **b** labial palp **c** maxilla **d** maxillary palp. Scale bars: 50 µm (**a**); 20 µm (**b, c**); 10 µm (**d**).

**Figure 41.**
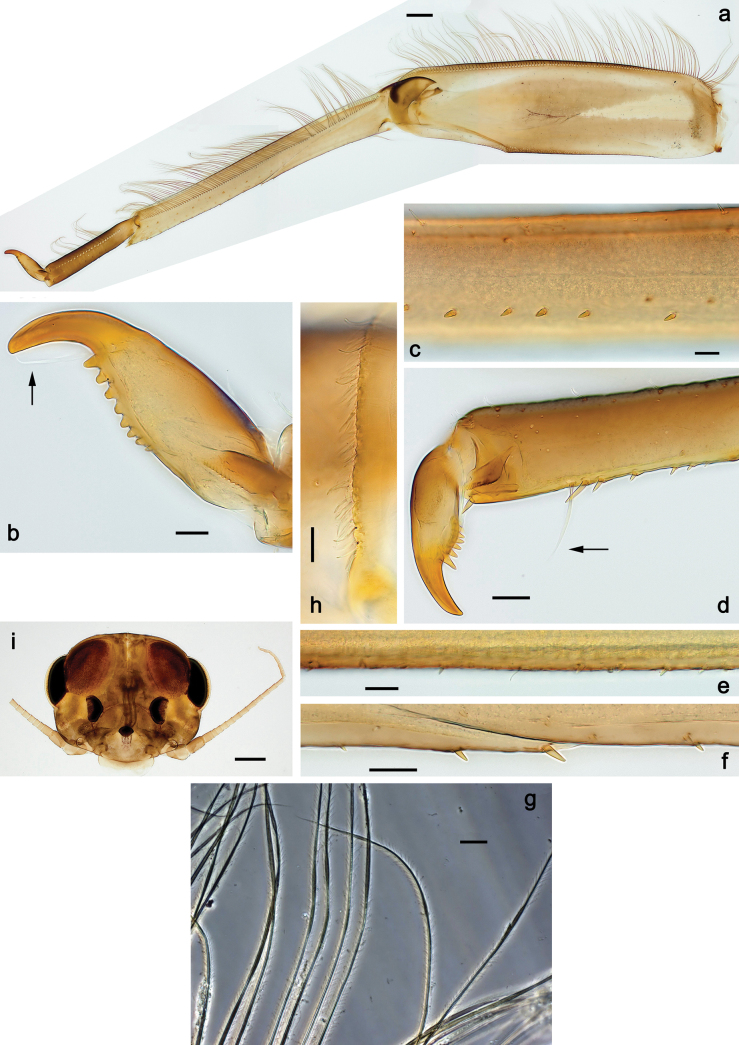
Papuanatula (Papuanatula) dumspinae sp. nov., larva: **a** hind leg **b** hind claw (arrow: posterior seta) **c** middle tibia, posterior surface **d** middle tarsus and claw **e** middle femur, ventral margin **f** hind tibia, ventral margin **g** hind femur: setae at dorsal margin **h** middle femur, posterior apex **i** male head, mature. Scale bars: 50 µm (**a**); 10 µm (**b, c, e–h**); 20 µm (**d**); 100 µm (**i**).

**Figure 42.**
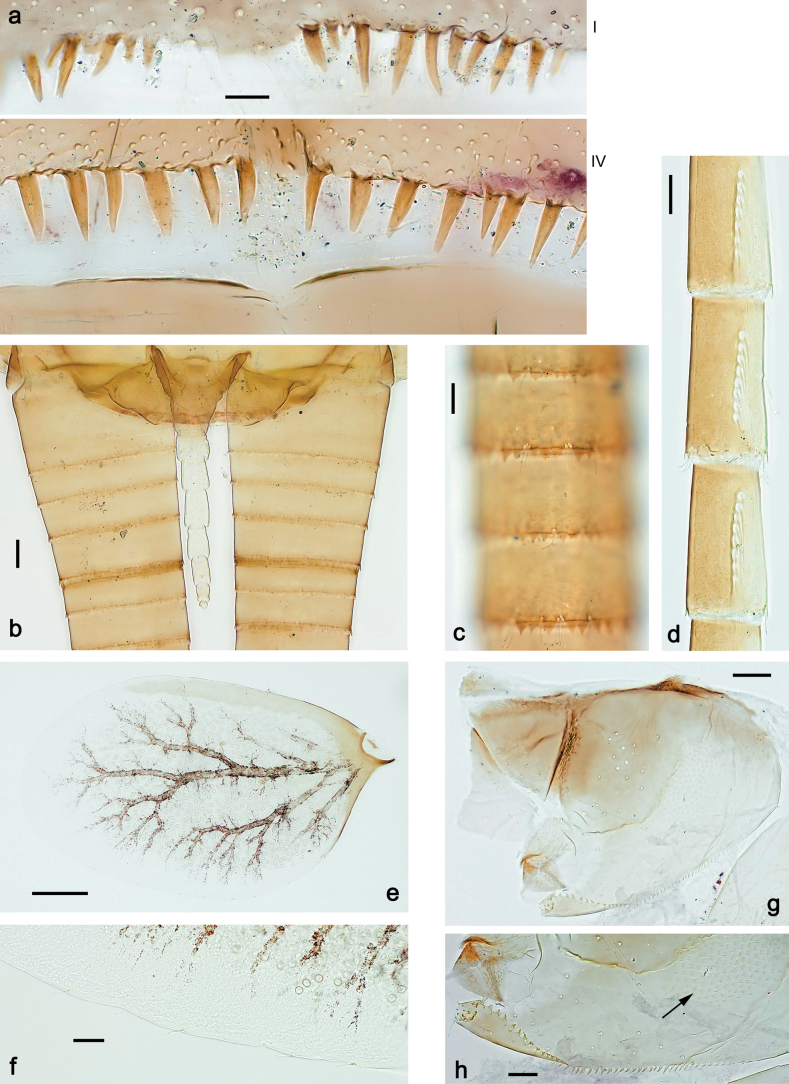
Papuanatula (Papuanatula) dumspinae sp. nov., larva: **a** abdominal terga **b** paracercus **c, d** cercus **e, f** tergalius IV, margin **g** paraproct **h** paraproct: distal margin (arrow: notched scales). Scale bars: 10 µm (**a, c, d, f, h**); 20 µm (**b, g**); 50 µm (**e**).

**Figure 43.**
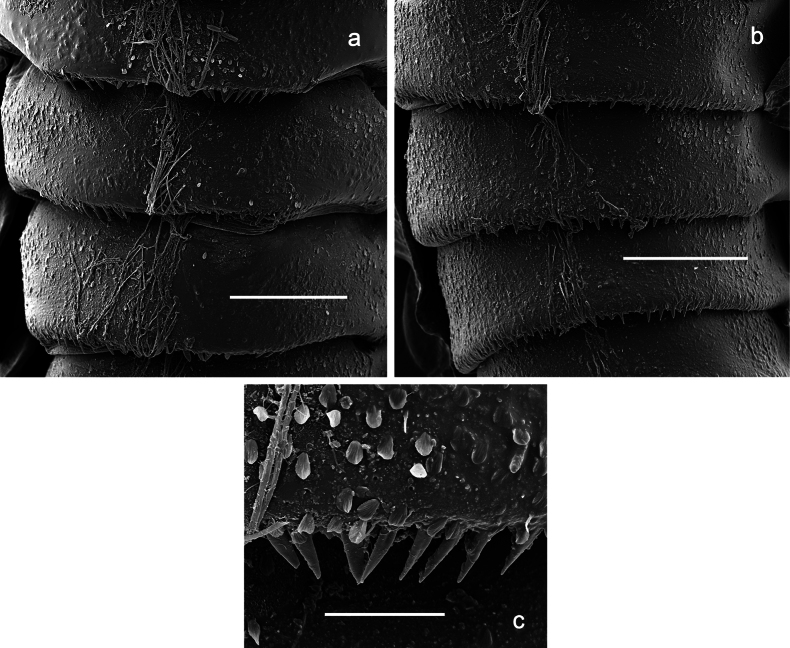
Papuanatula (Papuanatula) dumspinae sp. nov., larva (SEM): **a** abdominal terga III–V **b** abdominal terga VI–VIII **c** abdominal tergum III. Scale bars: 200 µm (**a, b**); 50 µm (**c**).

Figs 38 , 39 , 40 , 41 , 42 , 43

#### Etymology.

The species name is based on the Latin words *dum spinae* meaning “long spines”, referring to the long denticles at posterior margins of abdominal terga.

#### Material examined.

***Holotype*.** Indonesia • larva; Papua Prov.; Riv. Je, Loc. Arfak, E of Amber village; 01°06'35"S, 133°56'51"E; 1200 m; 16.vi.2016; leg. Sumoked and M. Balke; (BH68); on slide; GBIFCH00592625; MZB. ***Paratypes*.** 40 larvae; same data as holotype; 3 on slides; GBIFCH00592541, GBIFCH00592542, GBIFCH00975787, GBIFCH00976042; MZL; 37 in alcohol; GBIFCH00975791, GBIFCH00975792, GBIFCH00975794, GBIFCH00975795, GBIFCH00976046, GBIFCH00976047, GBIFCH00976048, GBIFCH00976059, GBIFCH00975830; MZL.

#### Other material.

Indonesia • 17 larvae; Papua Barat, Tamrau, Mts N of Kebar, sandy sunny riverbank; 00°47'02"S, 133°04'20"E; 758 m; 07.xi.2013; leg. M. Balke; (BH032); in alcohol; GBIFCH00975829; MZL.

#### Diagnosis.

**Larva.** The following combination of characters distinguishes *P.dumspinae* sp. nov. from other species of *Papuanatula* s. str.: body dorsally with row of long, fine, simple setae along midline; abdominal terga without protuberances; femur basally with wedge-shaped blank, medial area dark grey, distal area yellow-brown; paracercus with eight or nine segments; abdominal terga with very long, narrow, triangular denticles on posterior margins.

#### Description.

**Larva** (Figs 38–43). Body length 3.8–5.2 mm, cerci much longer than body length (~ 1.5×).

***Cuticular coloration*** (Fig. 38a–c). Head, thorax and abdomen dorsally brown; thorax with complex pattern; abdominal segments II–IV and VII–IX dark brown to black, V, VI and X yellow-brown. Femur basally with wedge-shaped blank, medial area dark grey, distal area yellow-brown; tibia ecru with grey; tarsus and claw grey-brown. Thorax and abdominal segment I ventrally ecru, protuberances of thoracic sterna dark brown, abdominal segments II–X ventrally brown. Cerci grey-brown.

***Hypodermal coloration*.** Each abdominal tergum I–IX with wide dark brown transverse band close to anterior margin and with narrower dark brown transverse band close to posterior margin (Fig. 38a).

***Head*.** Dorsally with irregular row of long, fine, simple setae along midline. ***Antenna*** (Fig. 41i). Length ~ 1.5× head length. As typical for subgenus. ***Developing turbinate eyes in mature male larva*** (Fig. 41i) ovoid, with large distance to each other. ***Labrum*** (Fig. 39a, b). Length 0.5× maximum width, laterally convex. Dorsal, sub-marginal arc with 22–29 feathered setae. ***Right mandible*** (Fig. 39d, e). Margin between prostheca and mola with row of minute denticles. Otherwise, as typical for subgenus. ***Left mandible*** (Fig. 39f, g). Margin between prostheca and mola with row of minute denticles. Otherwise, as typical for subgenus. ***Hypopharynx*** (Fig. 39c). As typical for genus. ***Maxilla*** (Fig. 40c, d). Maxillary palp somewhat shorter than galea-lacinia, robust; palp segment II ~ 1.3× length of segment I. Otherwise, as typical for genus. ***Labium*** (Fig. 40a, b). As typical for the genus. Paraglossa dorsally with two spine-like setae near inner, distolateral margin. Labial palp with segment I ~ 1.1× length of segments II and III combined. Segment II with minute distomedial protuberance, dorsally with row of four spine-like setae near outer, distolateral margin. Segment III slightly pentagonal, pointed; 0.7× length of segment II; inner dorsal margin with few feathered setae.

***Thorax*. *Sterna*.** With small protuberances on sides of prosternum and close to openings of mesothoracic and metathoracic sternal apodemes (as in Fig. 108a). ***Terga*** (Fig. 38b) without protuberances; with row of long, fine, simple setae along midline. ***Legs*** (Fig. 41a–h). Ratio of leg segments: fore leg 0.9:1.0:0.3:0.1, middle leg 0.9:1.0:0.3:0.1 and hind leg 1.1:1.0:0.3:0.2. ***Femur*.** Length ~ 3.5× maximum width. ***Claw*** with one row of 6–8 denticles and one posterior seta.

***Abdomen*. *Terga*** (Figs 42a, 43a–c) with row of long, fine, simple setae along midline. Terga without protuberances, terga I–IV with slight, paired, medioposterior elevations. Posterior margin of terga: (I)II–IX with long, narrow, triangular, pointed denticles. Surface with scattered small, paddle-like, striated scales with slightly serrate margin. ***Tergalii*** (Fig. 42e, f). Broad ovoid, tracheation well developed; margins smooth, with few short, fine, simple setae. ***Paraproct*** (Fig. 42g, h). Posterior margin with prolongation and row of minute denticles; on surface an area with minute, notched scales. ***Caudalii*** (Fig. 42b–d). Cerci apart from basal and distal part on ¾ of their length with up to ten swimming setae per segment, initially increasing and then again decreasing toward distal part. Paracercus with eight or nine segments.

***Pose of subimaginal gonostyli under larval cuticle*.** Unknown.

**Subimago.** Unknown.

**Imago.** Unknown.

**Egg.** Unknown.

#### Distribution.

New Guinea (Fig. 146).

### ﻿﻿ Papuanatula (Papuanatula) duplex sp. nov.

Taxon classificationAnimaliaEphemeropteraBaetidae

499A62E1-83BC-5CA0-A698-FC1A4219FE9E

https://zoobank.org/9EE270A3-651B-4589-AD09-BB9386148074

**Figure 44.**
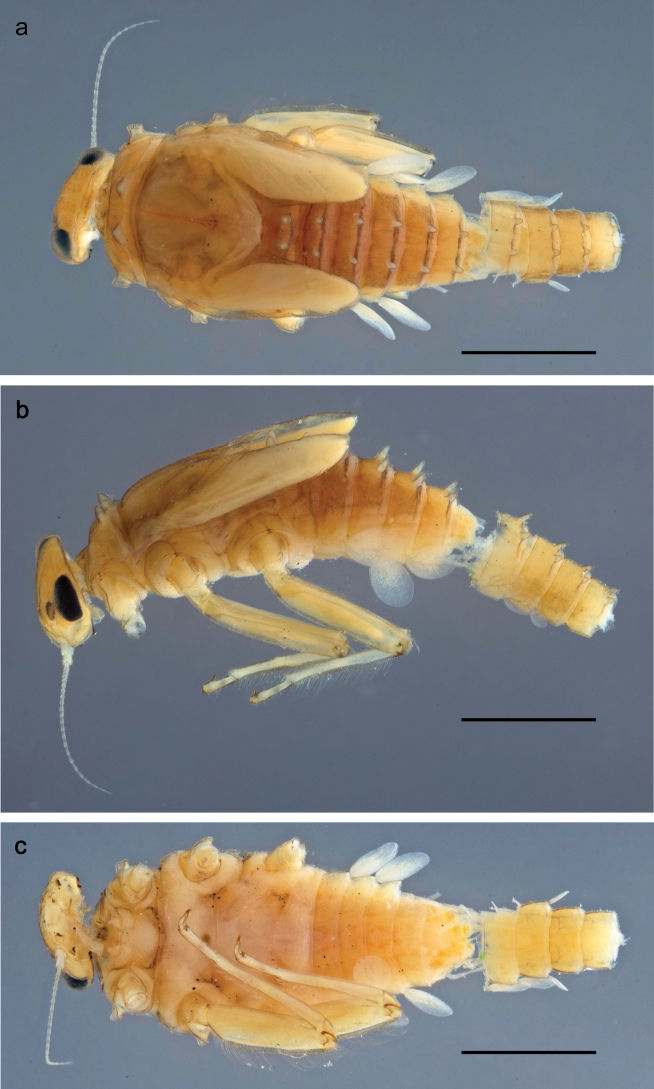
Papuanatula (Papuanatula) duplex sp. nov., larva, habitus **a** dorsal view **b** lateral view **c** ventral view. Scale bars: 1 mm.

**Figure 45.**
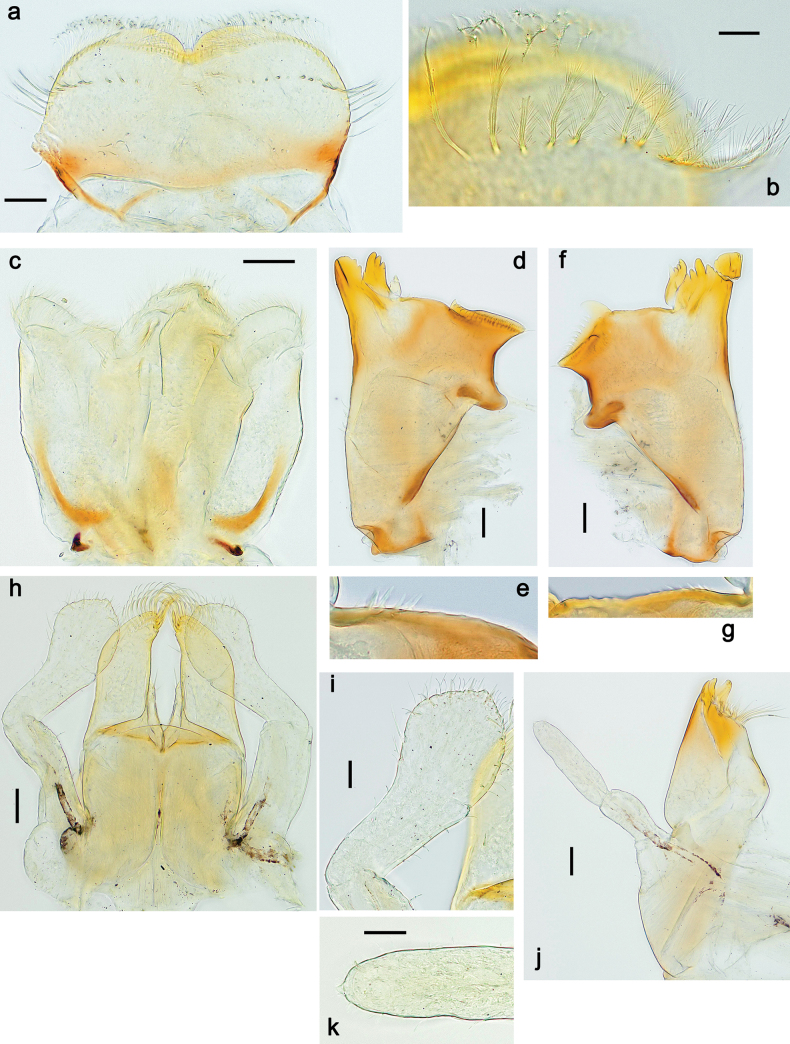
Papuanatula (Papuanatula) duplex sp. nov., larva **a** labrum **b** labrum: dorsal, submarginal setae **c** hypopharynx and superlinguae **d** right mandible **e** right mandible: margin between prostheca and mola **f** left mandible **g** left mandible: margin between prostheca and mola **h** labium **i** labial palp **j** maxilla **k** apex of maxillary palp. Scale bars: 20 µm (**a, c, d, f, h, j**); 10 µm (**b, i, k**).

**Figure 46.**
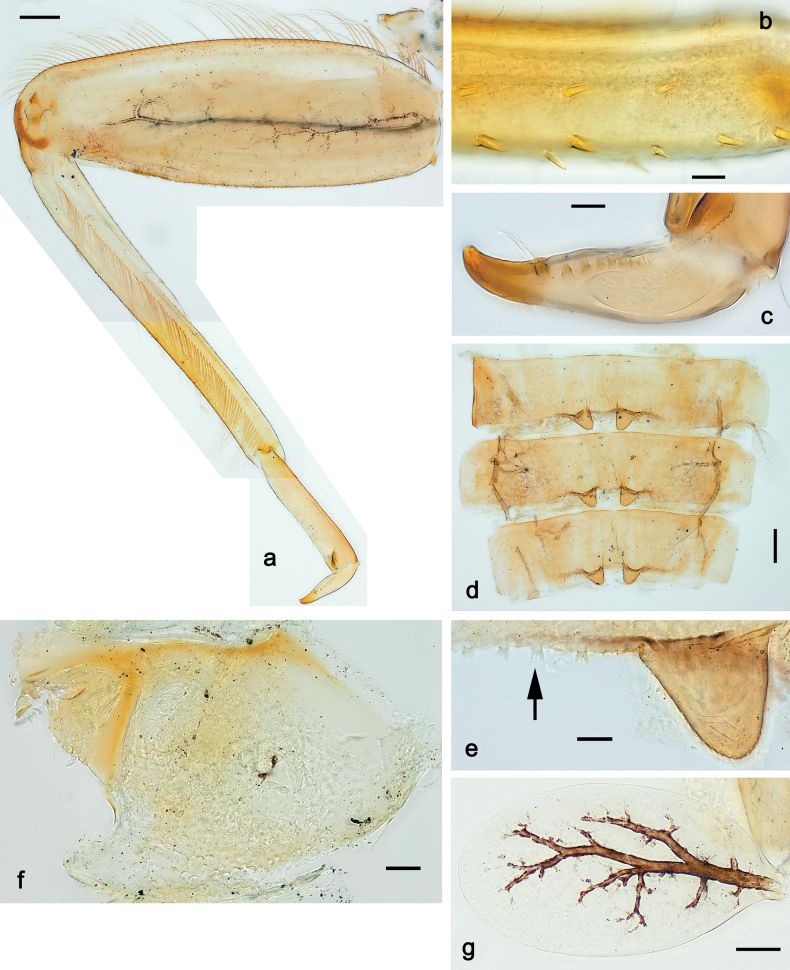
Papuanatula (Papuanatula) duplex sp. nov., larva: **a** foreleg **b** fore tibia, posterior surface **c** middle claw **d** abdominal terga VII–IX **e** abdominal tergum VI, posterior margin **f** paraproct **g** tergalius VII. Scale bars: 50 µm (**a**); 10 µm (**b, c, e, f**); 20 µm (**d, g**).

**Figure 47.**
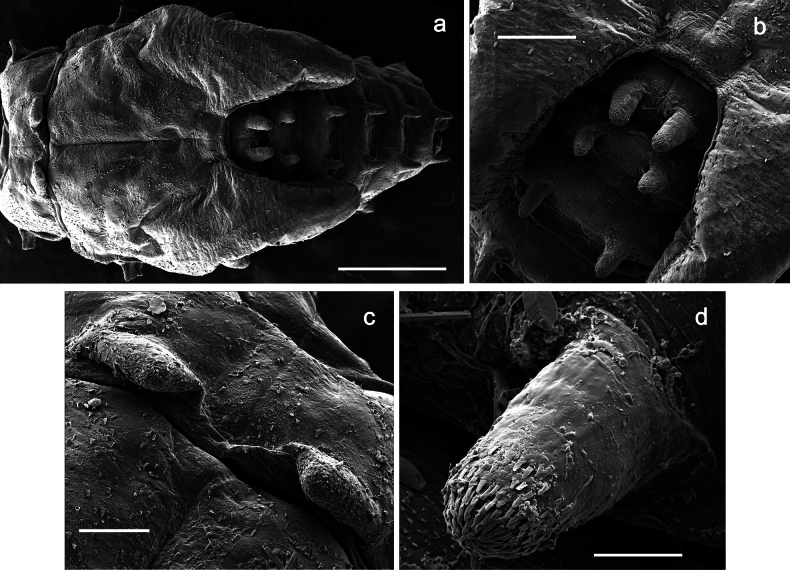
Papuanatula (Papuanatula) duplex sp. nov., larva (SEM): **a** pro-, meso-, metanotum, abdominal terga I–V **b** metanotum, abdominal terga I and II **c** pronotum **d** protuberance on metanotum. Scale bars: 500 µm (**a**); 200 µm (**b**); 100 µm (**c**); 40 µm (**d**).

Figs 44 , 45 , 46 , 47

#### Etymology.

The species name *duplex* is based on the Latin word for “double”, referring to the paired protuberances on abdominal terga.

#### Material examined.

***Holotype*.** Papua New Guinea • larva; Simbu Prov., Haia; ~ 750 m; 06.vii.2001; on slides; GBIFCH00592632, GBIFCH00976077; MZL.

#### Diagnosis.

**Larva**. The following combination of characters distinguishes *P.duplex* sp. nov. from other species of *Papuanatula* s. str.: body dorsally without row of long, fine, simple setae along midline; pronotum dorsally with posteromedial, paired, triangular protuberances; metanotum dorsally with posteromedial, paired, long, cylindrical, distally slightly conical protuberances; abdomen dorsally with posteromedial, paired protuberances: terga I–VI long, subcylindrical, distally slightly conical; terga VII–IX shorter, compressed, triangular.

#### Description.

**Larva** (Figs 44–47). Body length 4.5 mm, cerci broken.

***Cuticular coloration*** (Fig. 44a–c). Head, thorax and abdomen dorsally yellow-brown to brown; metanotum and abdominal terga I–IV darker than V–IX; Head, thorax, and abdominal segment I ventrally pale yellow-brown, abdominal segments II–IX yellow-brown; posterolateral protuberances on thorax brown. Legs yellow-brown; femur with long, narrow blank along dorsal margin.

***Hypodermal coloration*** (Fig. 44a). Abdominal segments I–IX dorsally with transverse band along posterior margins.

***Head*. *Antenna*** (Fig. 44a, b). Length ~ 1.5× head length. ***Developing turbinate eyes in last instar male larva*** unknown. ***Labrum*** (Fig. 45a, b). Length 0.5× maximum width, laterally convex. Dorsal, sub-marginal arc with ~ 12 feathered setae. ***Right mandible*** (Fig. 45d, e). Margin between prostheca and mola with row of short denticles. Otherwise, as typical for subgenus. ***Left mandible*** (Fig. 45f, g). Margin between prostheca and mola with row of short denticles. Otherwise, as typical for subgenus. ***Hypopharynx*** (Fig. 45c). As typical for genus. ***Maxilla*** (Fig. 45j, k). Maxillary palp subequal in length to galea-lacinia; palp segment II slightly longer than segment I. Otherwise, as typical for genus. ***Labium*** (Fig. 45h, i). As typical for the genus. Paraglossa with two spine-like setae on inner, distolateral margin. Labial palp with segment I 0.9× length of segments II and III combined. Segment II without distomedial protuberance, dorsally with row of five spine-like setae near outer, distolateral margin. Segment III slightly oblong, pointed, as long as segment II.

***Thorax*. *Sterna*.** With small protuberances on sides of prosternum and close to openings of mesothoracic and metathoracic sternal apodemes (as in Fig. 108a). ***Terga*** (Figs 44a, b, 47a–c) Pronotum with posteromedial, paired, triangular protuberances; metanotum with posteromedial, paired, long, cylindrical, distally slightly conical protuberances. ***Legs*** (Fig. 46a–c). Ratio of leg segments: fore leg 1.0:1.0:0.3:0.2, middle leg 1.0:1.0:0.3:0.2 and hind leg 1.0:1.0:0.4:0.2. ***Femur*.** Length ~ 3× maximum width. ***Claw*** with one row of eight denticles; one posterior seta.

***Abdomen*. *Terga*** (Figs 44a, b, 46d, e, 47a, b, d) I–IX with posteromedial, paired protuberances: I–VI long, subcylindrical, distally slightly conical; VII–IX shorter, compressed, triangular. Posterior margin of terga: I–V unknown; VI–IX with minute denticles, apically split with several points. Surface with scattered small, triangular, pointed, striated scales. ***Tergalii*** (Fig. 46g) ovoid, tracheation developed; margins smooth, with few short, fine, simple setae. ***Paraproct*** (Fig. 46f). Posterior margin with prolongation and row of minute denticles. ***Caudalii*.** Unknown.

***Pose of subimaginal gonostyli under larval cuticle***. Unknown.

**Subimago.** Unknown.

**Imago.** Unknown.

**Egg.** Unknown.

#### Distribution.

New Guinea (Fig. 146).

### ﻿﻿ Papuanatula (Papuanatula) epibessa sp. nov.

Taxon classificationAnimaliaEphemeropteraBaetidae

106D5D67-C348-50D1-8EA3-66C9811F1CF0

https://zoobank.org/87818654-CB3F-4E12-95F4-2E8C19DABD15

**Figure 48.**
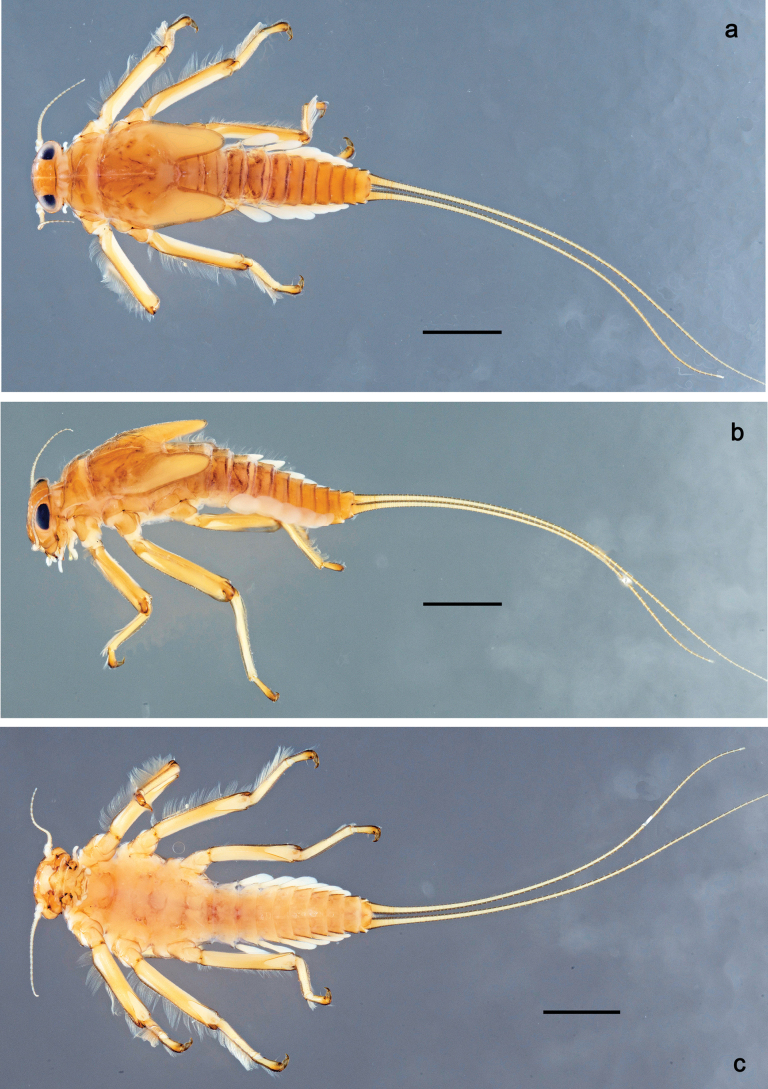
Papuanatula (Papuanatula) epibessa sp. nov., larva, female, habitus: **a** dorsal view **b** lateral view **c** ventral view. Scale bars: 1 mm.

**Figure 49.**
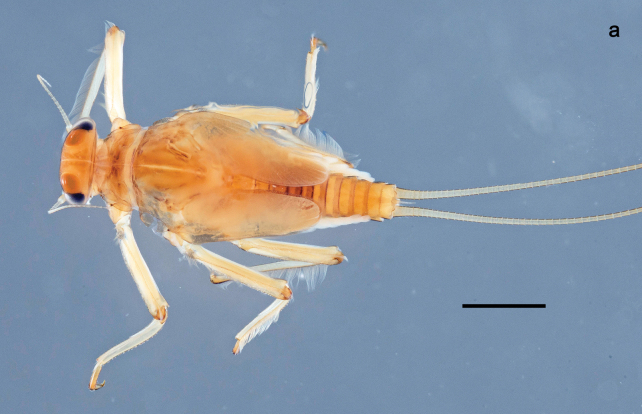
Papuanatula (Papuanatula) epibessa sp. nov., larva, male, habitus: **a** dorsal view. Scale bar: 1 mm.

**Figure 50.**
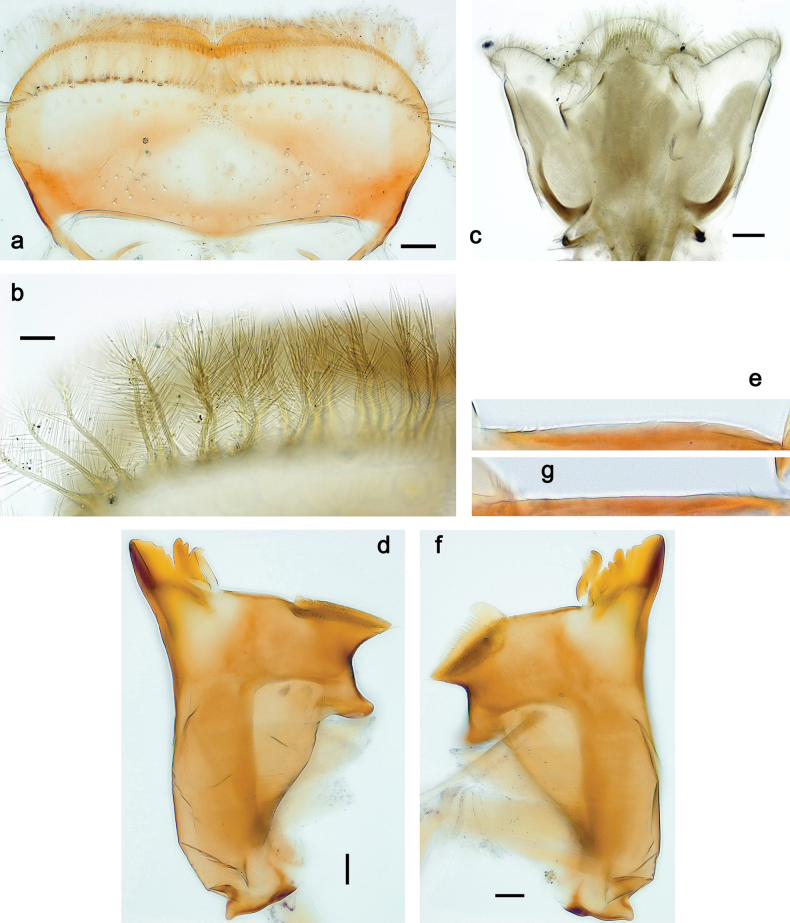
Papuanatula (Papuanatula) epibessa sp. nov., larva: **a** labrum **b** labrum: dorsal, submarginal setae **c** hypopharynx and superlinguae **d** right mandible **e** right mandible: margin between prostheca and mola **f** left mandible **g** left mandible: margin between prostheca and mola. Scale bars: 20 µm (**a, c, d, f**); 10 µm (**b**).

**Figure 51.**
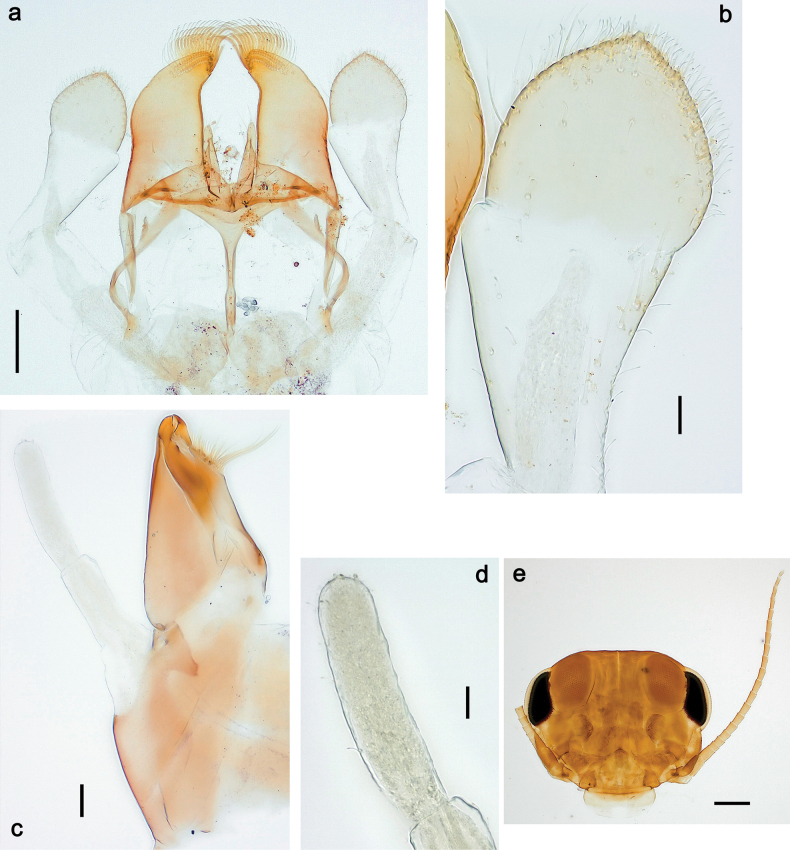
Papuanatula (Papuanatula) epibessa sp. nov., larva: **a** labium **b** labial palp **c** maxilla **d** maxillary palp **e** male head, mature. Scale bars: 50 µm (**a, c**); 20 µm (**b**); 10 µm (**d**); 100 µm (**e**).

**Figure 52.**
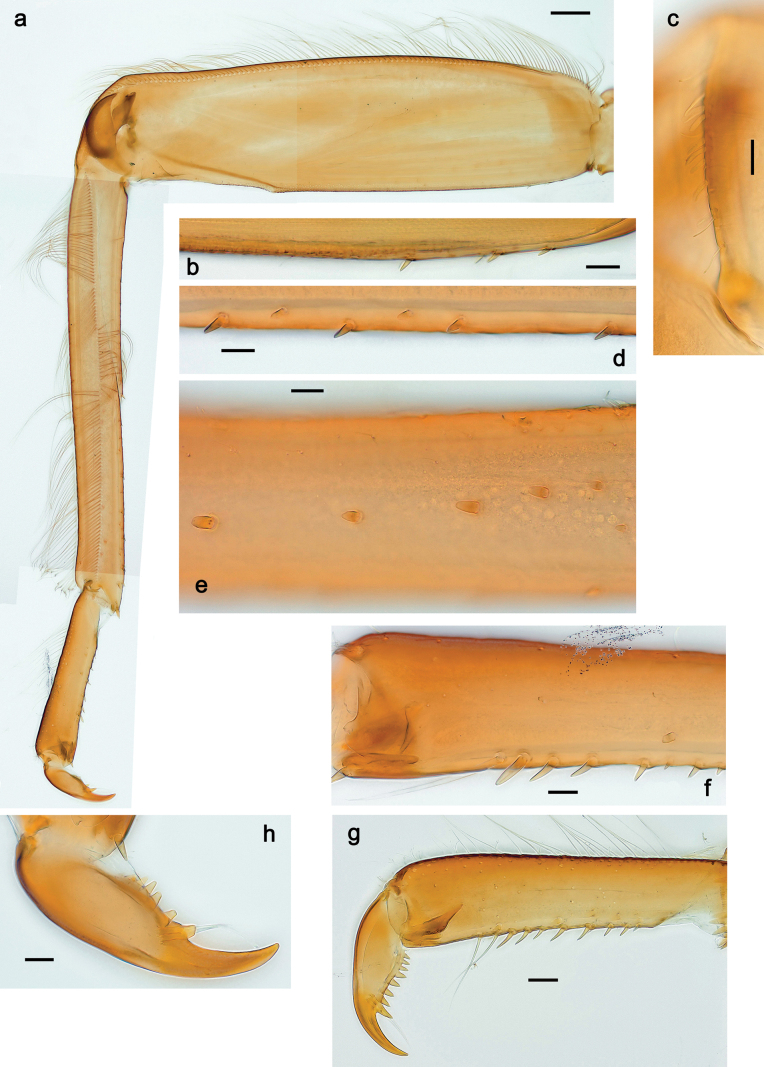
Papuanatula (Papuanatula) epibessa sp. nov., larva: **a** hind leg **b** hind femur: ventral margin **c** hind femur: posterior apex **d** hind tibia: ventral margin **e** hind tibia: posterior surface **f** hind tarsus: ventral surface **g** middle tarsus and claw **h** middle claw. Scale bars: a 50 µm (**a**); 10 µm (**b–f, h**); 20 µm (**g**).

**Figure 53.**
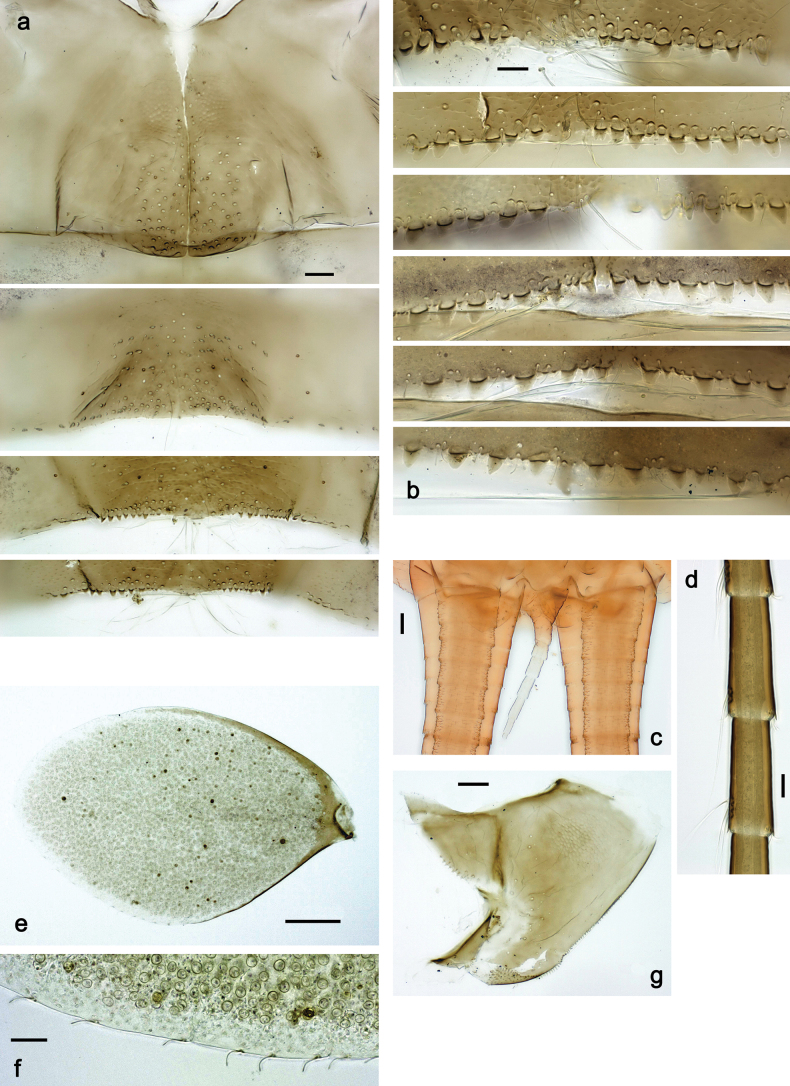
Papuanatula (Papuanatula) epibessa sp. nov., larva: **a** metanotum and abdominal terga I–III **b** abdominal terga IV–IX **c** paracercus **d** cercus **e** tergalius III **f** tergalius III, margin **g** paraproct. Scale bars: 20 µm (**a, c, g**); 10 µm (**b, d, f**); 50 µm (**e**).

**Figure 54.**
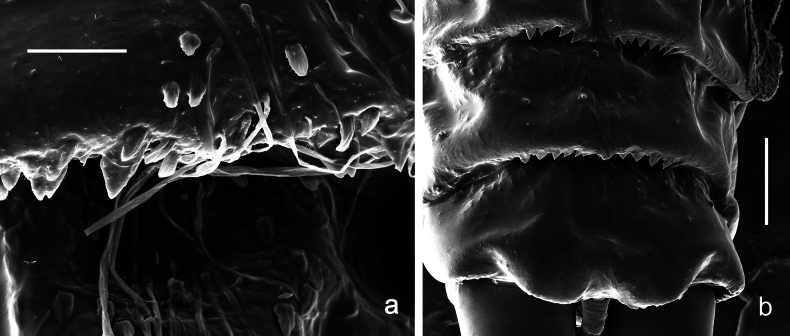
Papuanatula (Papuanatula) epibessa sp. nov., larva (SEM): **a** abdominal tergum V **b** abdominal terga VIII–X. Scale bars: 30 µm (**a**); 100 µm (**b**).

Figs 48 , 49 , 50 , 51 , 52 , 53 , 54

#### Etymology.

The species name *epibessa* refers to the morphological similarity with *P.bessa*.

#### Material examined.

***Holotype*.** Papua New Guinea • larva; Simbu Prov., Mt. Wilhelm, Pindaunde Creek, in forest, S3 (oria 4); 05°49'S, 145°04'30"E; 2900 m; 18.viii.1999; leg. L. Čížek; on slide; GBIFCH01221766; MZL. ***Paratypes*.** 31 larvae; same data as holotype; in alcohol; GBIFCH00976020, GBIFCH00976121; MZL • 93 larvae; Simbu Prov., Mt. Wilhelm, Pindaunde Creek, near fish farm, S4 (oria 5); 05°49'02"S, 145°05'16"E; 2600 m; 18.viii.1999; leg. L. Čížek; 3 on slides; GBIFCH01221759, GBIFCH01221761, GBIFCH01221767; MZL; 90 in alcohol; GBIFCH00976021, GBIFCH00976023, GBIFCH00976024, GBIFCH00976126; MZL • 18 larvae; Simbu Prov., Mt. Wilhelm, Pindaunde Creek, S2 (oria 2); 05°48'03"S, 145°04'09"E; 3210 m; 17.viii.1999; leg. L. Čížek; 1 on slide; GBIFCH01221762; 17 in alcohol; GBIFCH00976124; MZL.

#### Diagnosis.

**Larva**. The following combination of characters distinguishes *P.epibessa* sp. nov. from other species of *Papuanatula* s. str.: body dorsally with irregular row of long, fine, simple setae along midline; metanotum and abdominal terga I–III with medioposterior, broad, paired humps, poorly developed on terga I–III; femur yellow brown, basally with wedge-shaped blank; paracercus with 6–8 segments; claw with 1–3 posterior setae.

#### Description.

**Larva** (Figs 48–54). Body length 4.1–5.5 mm, cerci much longer than body length (~ 1.7×).

***Cuticular coloration*** (Figs 48a–c, 49a) Head, thorax and abdomen dorsally yellow brown to brown, thorax with complex markings. Abdominal terga II–V with brown markings along anterior margin, tergum III additionally with mediolateral, brown markings. Femur yellow brown, basally with wedge-shaped blank. Tibia pale yellow brown; tarsus yellow brown. Cerci pale yellow brown.

***Hypodermal coloration*** (Figs 48a, 49a) Abdominal terga with narrow, dark brown, transverse bands along posterior margin.

***Head*.** Dorsally with irregular row of long, fine, simple setae along midline. ***Antenna*** (Fig. 51e). Length ~ 1.5× head length. ***Developing turbinate eyes in last instar male larva*** (Fig. 51e) rather small, ovoid, with big distance to each other. ***Labrum*** (Fig. 50a, b). Length 0.5× maximum width, laterally convex. Dorsal, sub-marginal arc with ~ 35 feathered setae. ***Right mandible*** (Fig. 50d, e). Margin between prostheca and mola with few denticles close to prostheca. Otherwise, as typical for subgenus. ***Left mandible*** (Fig. 50f, g). Margin between prostheca and mola smooth, with few denticles close to prostheca. Otherwise, as typical for subgenus. ***Hypopharynx*** (Fig. 50c). As typical for genus. ***Maxilla*** (Fig. 51c, d). Maxillary palp subequal in length to galea-lacinia; palp segment II approximately as long as segment I. Otherwise, as typical for genus. ***Labium*** (Fig. 51a, b). As typical for the genus. Paraglossa dorsally with 2 spine-like setae near inner, distolateral margin. Labial palp with segment I approximately as long as segments II and III combined. Segment II with minute distomedial protuberance, dorsally with row of five or six spine-like setae near outer, distolateral margin. Segment III conical, pointed; 0.8× length of segment II; inner dorsal margin with few feathered setae.

***Thorax*. *Sterna*.** With small protuberances on sides of prosternum and close to openings of mesothoracic and metathoracic sternal apodemes (as Fig. 108a). ***Terga*** (Figs 48b, 53a) with irregular row of long, fine, simple setae along midline. Metanotum with medioposterior broad, paired humps, without hind protoptera or their vestiges. ***Legs*** (Fig. 52a–h). Ratio of leg segments: fore leg 0.8:1.0:0.3:0.1, middle leg 0.9:1.0:0.3:0.1 and hind leg 1.0:1.0:0.3:0.1. ***Femur*.** Length ~ 4× maximum width. Posterior side of apex with row of robust setae (contrary to fine, simple setae as it is usually the case in *Papuanatula* s. str.). ***Tarsus*.** Inner margin distally sometimes with two long setae (Fig. 52g). ***Claw*** with one row of 6–8(9) denticles and 1–3 posterior setae.

***Abdomen*. *Terga*** (Figs 48b, 53a, b, 54a, b) with irregular row of long, fine, simple setae along midline. Terga I–III with poorly developed, medioposterior, broad, paired humps. Terga II–IV protruding slightly medially on posterior margin. Posterior margin of terga: I smooth, without denticles; II–IX with triangular, apically rounded denticles, partly with minute, pointed denticles in between. Surface with scattered small, ovoid, striated scales with slightly serrate margin. ***Tergalii*** (Fig. 53e, f). Ovoid, tracheation poorly developed or absent; margins smooth, with short, fine, simple setae. ***Paraproct*** (Fig. 53g). Posterior margin with prolongation and row of minute denticles. ***Caudalii*** (Fig. 53c, d). Cerci in middle part with one to maximally five swimming setae per segment, initially increasing and then again decreasing toward distal part; sometimes total loss or maximally one swimming seta per segment. Paracercus with 6–8 segments.

***Pose of subimaginal gonostyli under larval cuticle*.** Unknown.

**Subimago.** Unknown.

**Imago.** Unknown.

**Egg.** Unknown.

#### Distribution.

New Guinea (Fig. 146).

### ﻿﻿ Papuanatula (Papuanatula) heterochaeta sp. nov.

Taxon classificationAnimaliaEphemeropteraBaetidae

0F1C9FB3-2081-5E09-80C3-B0BE679108BF

https://zoobank.org/F2A1E4AD-E2A2-4004-BA7C-6EEFBBAAA3E9

**Figure 55.**
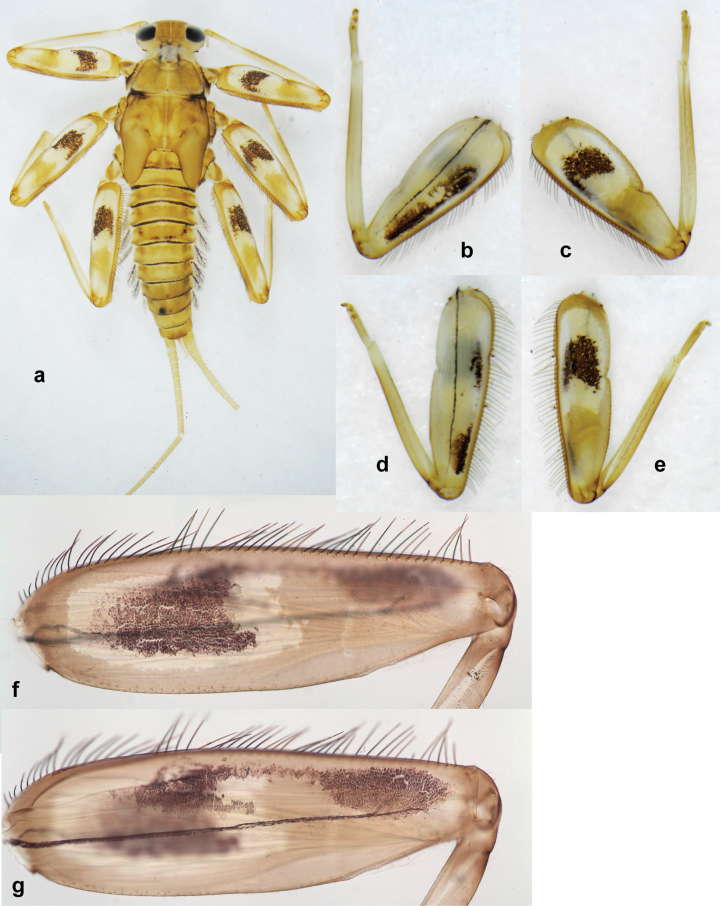
Papuanatula (Papuanatula) heterochaeta sp. nov., larva: **a** dorsal view **b**, **c** posterior and anterior sides of fore leg **d**, **e** posterior and anterior sides of hind leg **f, g** hind leg in Canada balsam with focus on anterior and posterior sides.

**Figure 56.**
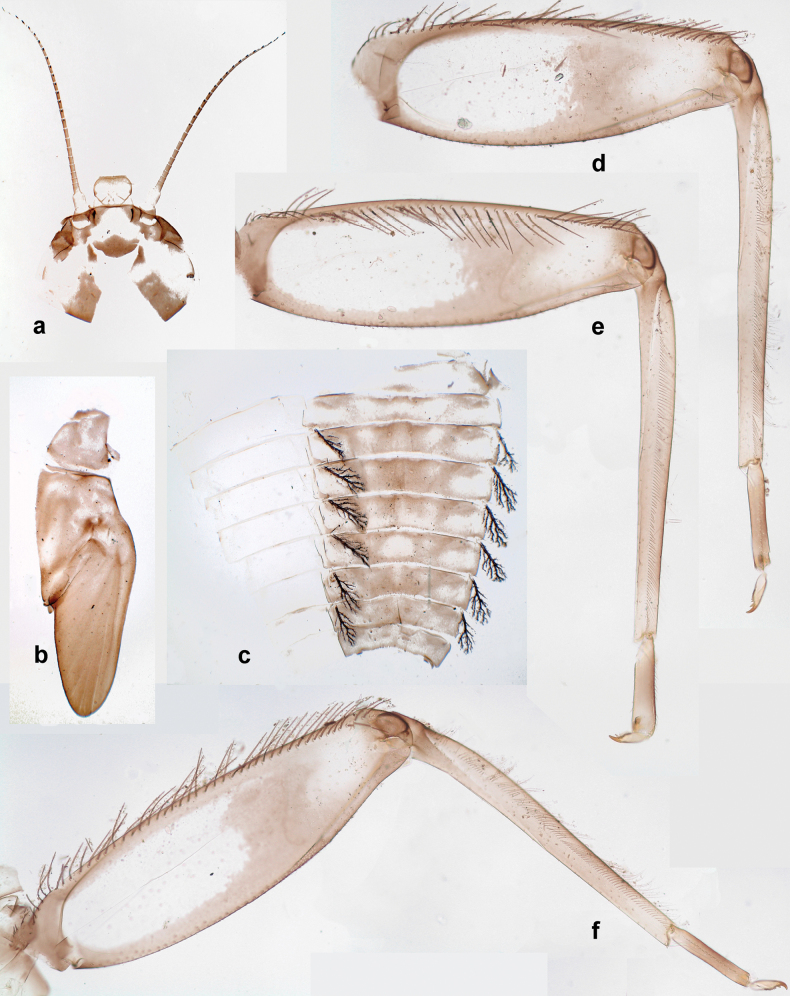
Papuanatula (Papuanatula) heterochaeta sp. nov., larval cuticle: **a** head **b** half of pronotum and mesonotum **c** abdomen **d, e** fore, middle, and hind legs (enlarged) (**b–f** holotype).

**Figure 57.**
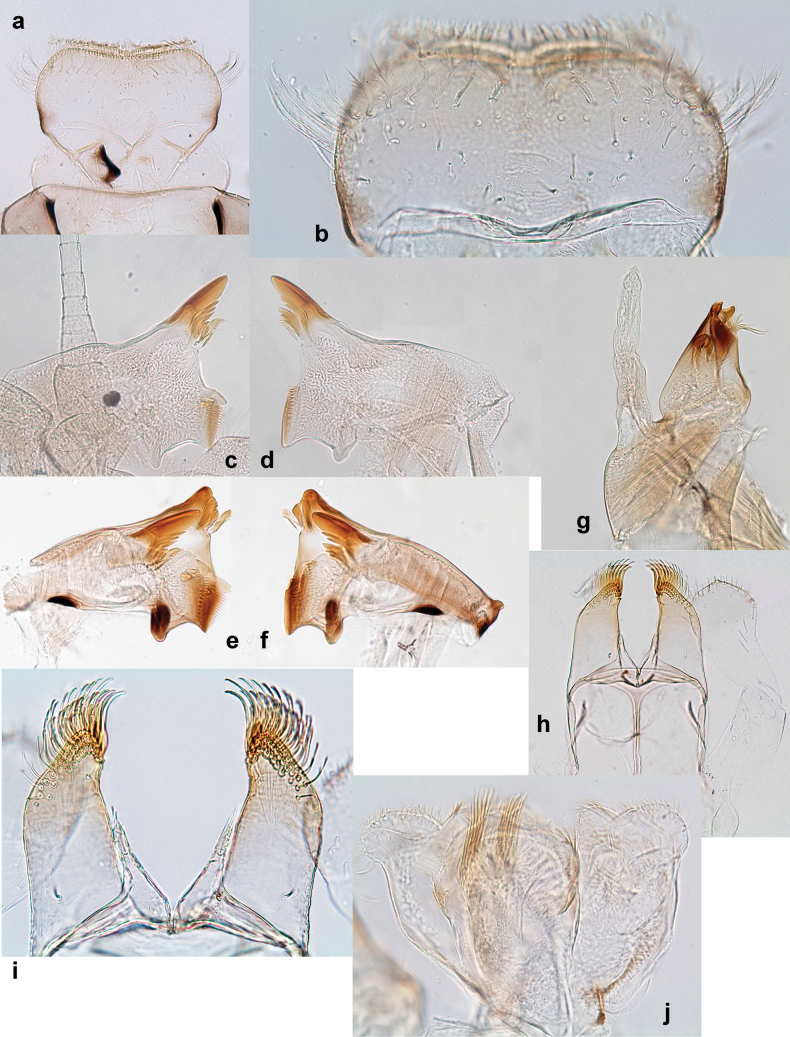
Papuanatula (Papuanatula) heterochaeta sp. nov., larva: **a, b** labrum **c, d** mandibles shortly after molt **e, f** mandibles before molt **g** maxilla before molt **h, i** labium **j** hypopharynx.

**Figure 58.**
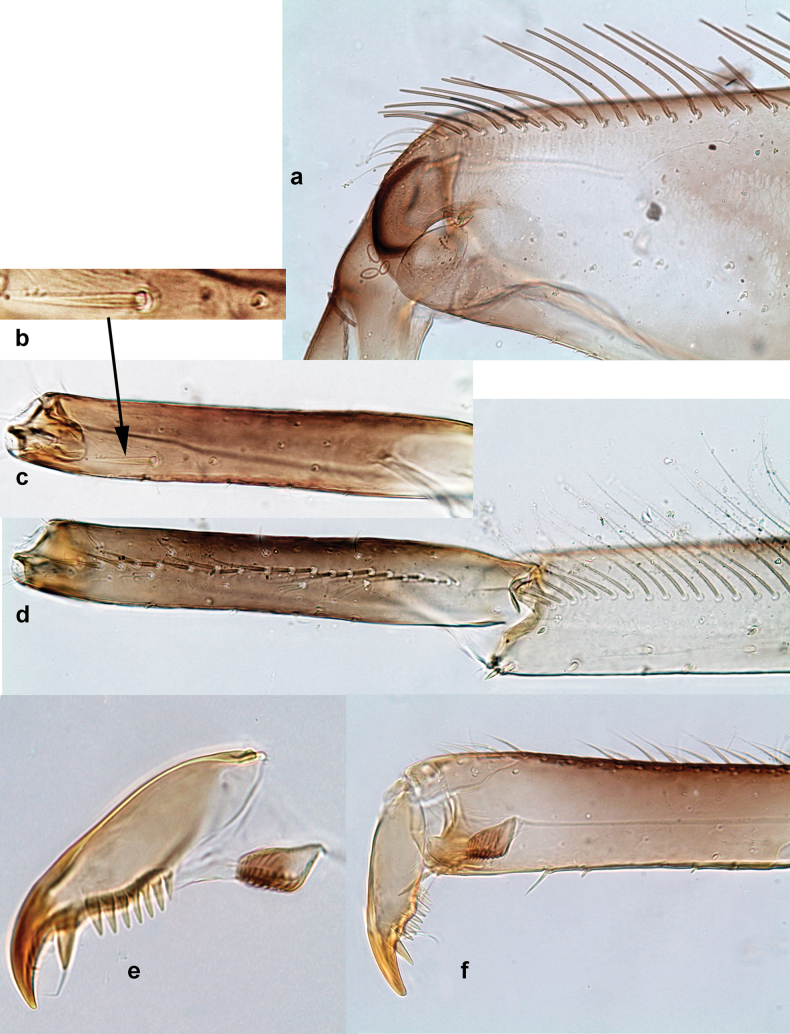
Papuanatula (Papuanatula) heterochaeta sp. nov., larva: **a** apex of femur **b, c** inner side of tarsus **d** outer side of tarsus and tibia **e** claw **f** claw and tarsus (**b–f** holotype).

**Figure 59.**
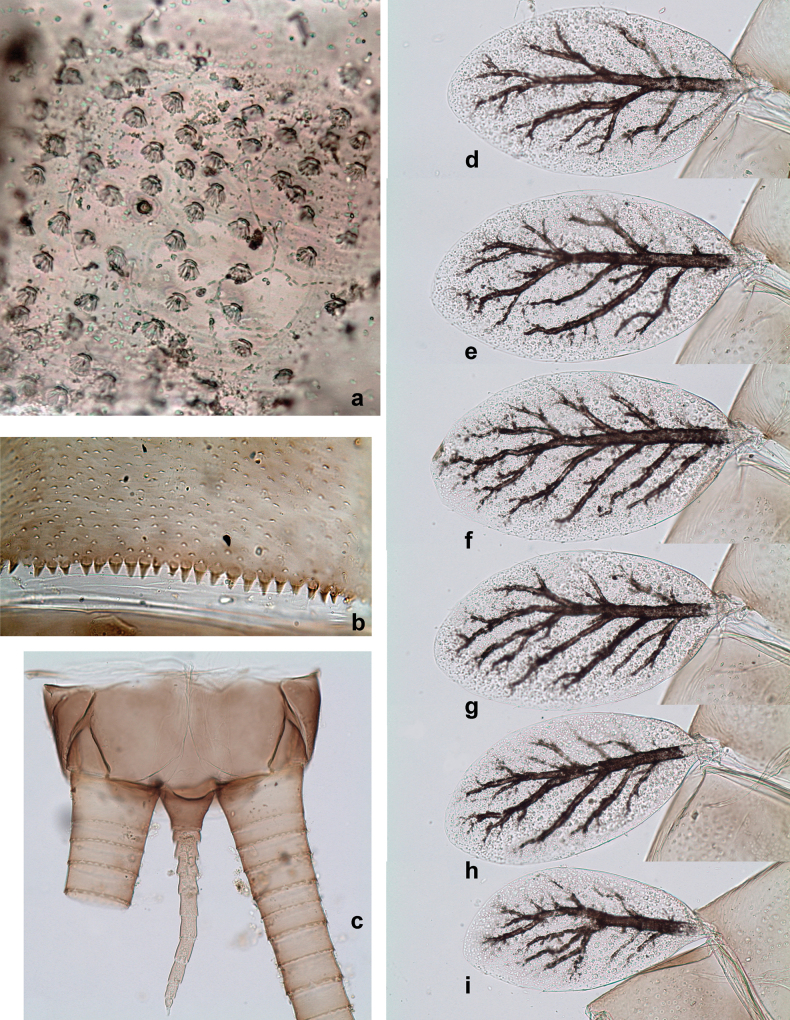
Papuanatula (Papuanatula) heterochaeta sp. nov., larva: **a** abdominal tergum III (dry) **b** hind margin of abdominal tergum V **c** paraprocts and paracercus **d–i** tergalii II–VII (**a–c** holotype).

**Figure 60.**
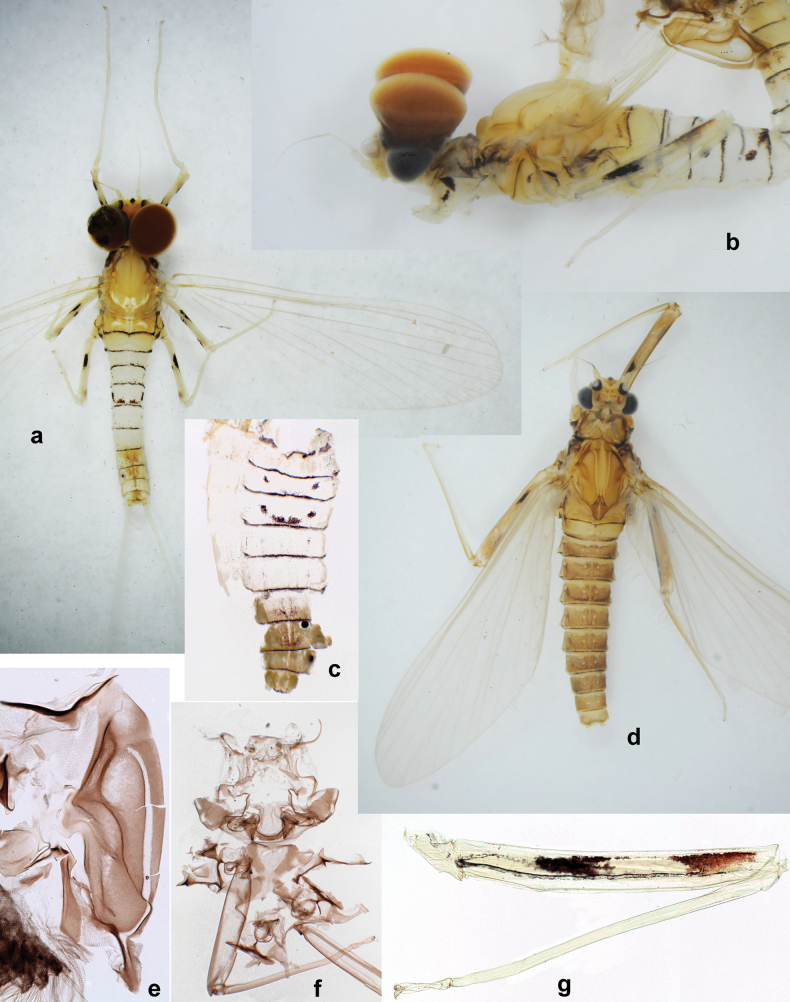
Papuanatula (Papuanatula) heterochaeta sp. nov. **a, b** male imagines **c** abdomen of male imago **d** female subimago **e** subimaginal exuviae of mesonotum **f** subimaginal exuviae of head, prothorax, sterna, and pleura of mesothorax and metathorax **g** middle leg of male imago (**c, e–g** holotype).

**Figure 61.**
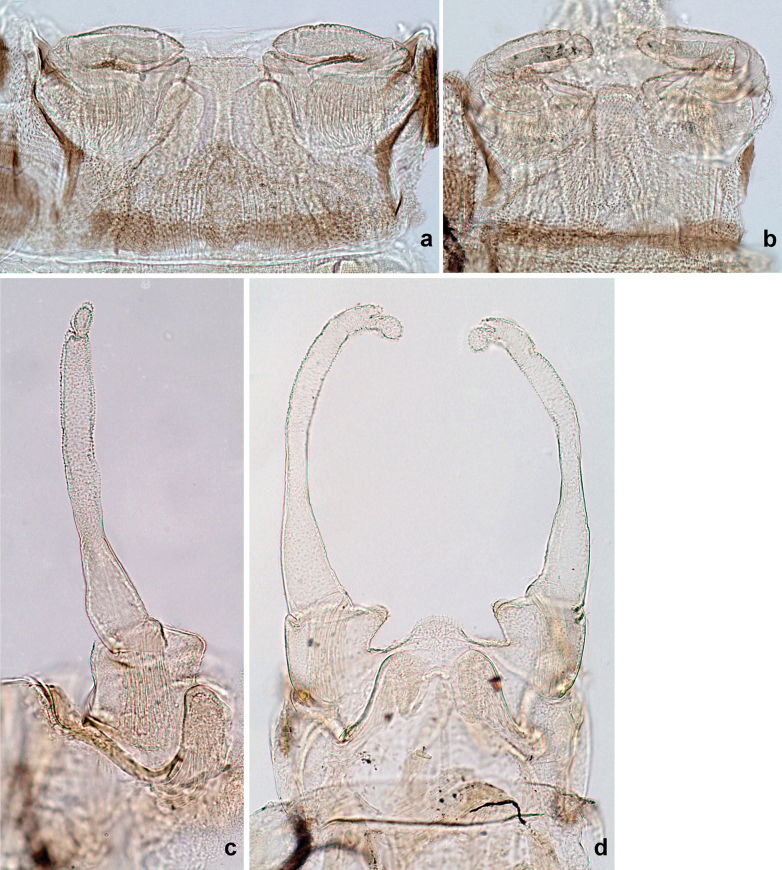
Papuanatula (Papuanatula) heterochaeta sp. nov., male genitalia: **a** subimaginal genitalia developing under larval cuticle **b** subimaginal genitalia starting to spread **c**, **d** imaginal genitalia (d holotype).

Figs 55 , 56 , 57 , 58 , 59 , 60 , 61

#### Etymology.

The species name *heterochaeta* refers to the sharp difference between blunt-ended setae which form the longitudinal row on outer side of larval femur and small hair-like setae which form continuation of this row on apex of the femur (Fig. 58a).

#### Material examined.

***Holotype*.** L-S-I♂ {specimen number [XX](5)A2012} Indonesia • Papua, Depapre; 28.viii.2012; coll. N. Kluge & L. Sheyko; SPbU. ***Paratypes*.** Same data as holotype; S-I♂ (reared together with holotype, larval exuviae lost); same data as holotype; 25–28.viii.2012; 1 S-I♂, 10 larvae. Indonesia • Waena; 8–13.viii.2012; coll. N. Kluge & L. Sheyko; 2 L-S♀, S/I♂, 6 larvae. All material in SPbU.

#### Diagnosis.

**Larva.** The following combination of characters distinguishes *P.heterochaeta* sp. nov. from other species of *Papuanatula* s. str.: body dorsally without irregular row of long, fine, simple setae along midline; abdominal terga without protuberances; hypopharynx apically with pair of bunches of setae-like spines (instead of one bunch as usually); femur with large, proximal, oval blank and with dark brown, shoe-shaped macula inside proximal blank; sharp difference between setae which form the longitudinal row on outer side of femur and setae which form continuation of this row on apex of femur; long, slender, preapical seta on tarsus absent; paracercus with ~ 10–12 segments.

#### Description.

**Larva** (Figs 55–59). ***Cuticular coloration*.** Head, pronotum, mesonotum and metanotum brownish with paler areas; fore protoptera with narrow paler lines corresponding to some longitudinal veins (Fig. 56a, b). Thoracic pleura brownish, sterna mostly colorless. Cuticle of femur with brownish margins and brownish transverse band separating large proximal blank from smaller distal blank; proximal blank oval (not wedge-shaped), occupying most part of proximal 1/2 (Fig. 56d–f). Tibia and tarsus mostly brownish (Fig. 56d–f). Abdominal terga brownish with paler blanks; median blank on tergum VI larger than others. Sterna mostly colorless (Fig. 56c). Cerci uniformly pale brownish.

***Hypodermal coloration*.** Anterior side of each femur with dark brown shoe-shaped macula on proximal 1/2, within proximal cuticular blank (Fig. 55f, g); posterior side of each femur with or without two longitudinal, brown maculae (Fig. 55d), sometimes connected one with another (Fig. 55b). Boundaries between abdominal terga narrowly bordered by dark brown; other brown markings on abdomen absent or present, most extensive on abdominal tergum IV (Fig. 55a). Tissues surrounding tracheae of tergalii (main trachea and its branches) with brown pigmentation (Fig. 59d–i).

***Head*. *Antenna*.** As typical for subgenus. ***Developing turbinate eyes in last instar male larva*** with facets equally developed on middle and peripheric areas (as in Fig. 32d). ***Labrum*** (Fig. 57a, b) very slightly widened distally; long setae on dorsal surface spaced and forming regular transverse row; each seta pointed, with moderately long processes on both sides. ***Right mandible*** (Fig. 57d, f). As typical for subgenus. ***Left mandible*** (Fig. 57c, e). Incisor and kinetodontium non-distinguishable, together with 5 denticles proximad of stretched apex of incisor. Otherwise, as typical for subgenus. ***Hypopharynx*** (Fig. 57j) apically with pair of bunches of stout setae-like spines. ***Maxilla*** (Fig. 57g). Maxillary palps long as galea-lacinia. Otherwise, as typical for genus. ***Labium*** (Fig. 57h, i). Paraglossae widest at base and narrowing toward apex; three apical setal rows bent at apex of paraglossa. Glossa shorter than half of paraglossa, with finger-like (distal) portion as long as triangular (proximal) portion. Glossa with several long setae at apex and one long seta near middle of ventral side. Labial palp without distomedian projection on segment II; segment III with median margin as long as lateral margin.

***Thorax*. *Sterna*** without protuberances. ***Terga*.** Without long setae along midline. Metanotum without hind protoptera or their vestiges. ***Legs*** (Figs 56d–f, 58a–f). Fore femur widened in proximal part; hind tibia shorter than others. ***Femur*.** Outer side of each femur with single regular row of long setae; each seta slender, flattened, narrowing toward apex and blunt apically, with numerous fine, short branches on all sides. Distally, close to femur-tibia articulation, setal row continued by several smaller, hair-like setae (similar to setae on tibia). Anterior side of femur with small, stout setae, sparsely and irregularly situated. Serrate area located at middle of anterior side, partly on brown transverse band, partly on proximal blank. ***Tibia*.** Patella-tibial suture present on all legs, terminated near middle of inner margin of tibia. Tibia-tarsal condylus turned to anterior side. Anterior side of each tibia with regular row of hair-like setae similar to setae near apex of femur. ***Tarsus*.** Anterior side of each tarsus with regular row of similar, but shorter (not narrower) setae. Long preapical seta absent; posterior side of each tarsus with regular row of few very short, stout setae and one longer, pointed seta of same thickness distad of them (Fig. 58b, c). ***Claw*** with row of 6–9 denticles and one somewhat larger denticle distad of them; long, arched, posterior seta (Fig. 58e, f).

***Abdomen*. *Terga*** (Figs 56c, 59a, b) without dorsal protuberances, only with slightly expressed, unpaired, median elevations; without long setae along midline. Abdominal terga with numerous small scales with small sockets and fan-like striation. Abdominal terga I–III without denticles on posterior margins; posterior margins of abdominal terga IV–IX with small, pointed denticles. Posterior margin of tergum X with very small denticles. ***Tergalii*** (Fig. 59d–i) of abdominal segment I absent; tergalii II–VII subequal, oval, relatively narrow. Each tergalius with costal and anal ribs narrow, smooth, present on proximal 1/2 of tergalius only. ***Paraproct*** (Fig. 59c). Margins membranous and smooth, lacking denticles. ***Caudalii*** (Fig. 59c) without swimming setae or their vestiges. Paracercus short, consisting of ~ 10–12 segments.

***Pose of subimaginal gonostyli under larval cuticle*** (Fig. 61a). In mature larva ready to molt to subimago, subimaginal gonostyli packed under larval cuticle in “*Labiobaetis*-type” pose, as typical for the genus. 2^nd^ segment directed medially and bent proximally; 3^rd^ segment directed medially (as continuation of 2^nd^ segment) and narrowed apically, being deformed corresponding to space between subimaginal styliger and larval cuticle.

**Subimago. *Cuticular coloration***. Pronotum and prosternum partly brown (Fig. 60f). Mesonotum pale brown with medioparapsidal suture colorless, other sutures darker brown (Fig. 60e). Meso- and metathoracic pleura and sterna with colorless, pale brownish and dark brown areas (Fig. 60f). Cuticle of wings colorless, with microtrichiae brownish. Legs nearly colorless, with pale brown bordering on femur and base of tibia (Fig. 60d). Abdomen very pale brownish with colorless sigilla. Cerci colorless with setae brownish.

***Hypodermal coloration*.** As in imago.

***Texture*.** On all legs of both sexes, each tarsomere covered mostly with blunt microlepides, with pointed microlepides near apex (as in Fig. 70i).

**Imago. *Imago, male*.** Head ochre. Antennae ochre. Turbinate eyes red, widened apically. Thorax ochre, equally pale dorsally, laterally, and ventrally, with dark brown hypodermal markings on lateral sides. Fore wing with membrane colorless, veins ochre. Pterostigma with three or four incomplete, oblique cross veins (Fig. 60a–c). Legs mostly ochre; on leg of each pair, anterior side of femur with contrasting, dark brown, longitudinal macula just proximad of midlength; posterior side of femur with reddish brown macula near apex (Fig. 60g). Abdomen mostly whitish or ochre; each tergum I–IX with dark brown transverse band close to posterior margin; each tergum III and IV with pair of brown spots; tergum IV with transverse brown macula posteriad-mediad of them (Fig. 60a–c). Cerci ochre.

***Genitalia*** (Fig. 61c, d). Sterno-styligeral muscle absent. Each unistyliger sharply widened apically on median side, so that median margins convergent distally. 1^st^ segment of gonostylus with lateral side convex, median side median side gradually turns to 2^nd^ segment. Second segment equally wide all over its length. Third (terminal) segment of gonostylus nearly as wide as 2^nd^, with length slightly exceeding width. Penial bridge with wide, blunt, membranous projection between unistyligers. Each gonovectis parabolic, with lateral (basal) and median (apical) portions equally long, apex bent medially-caudally.

***Imago, female*** (Fig. 60d). Unknown. Judging by subimago, coloration of head, thorax, and legs similar to that of male; abdominal terga with ochre-brown pigmentation.

**Egg.** Unknown.

#### Dimension.

Fore wing length (and approximate body length) 4–5 mm.

#### Distribution.

New Guinea (Fig. 147).

### ﻿﻿ Papuanatula (Papuanatula) normungulata sp. nov.

Taxon classificationAnimaliaEphemeropteraBaetidae

44836D37-1795-592F-9632-3CCBE3EAB69A

https://zoobank.org/AF701FE9-D968-4780-B90A-B8E225ABA58E

Figs 62 , 63 , 64

#### Etymology.

The species name *normungulata* refers to the larval claw structure, which lacks the arched posterior seta and the enlarged denticle associated with this seta that is normal for the plesiomorphon *Papuanatula*, being different from other species of *Papuanatula*.

#### Material examined.

***Holotype*.** female larva with partly developed subimaginal details; Indonesia • Sulawesi, tributary of river Mamasa, 5 km W Mamasa; 15–27.viii.2009; coll. N. Kluge & L. Sheyko; SPbU.

#### Diagnosis.

**Larva.** The following combination of characters distinguishes *P.normungulata* sp. nov. from other species of *Papuanatula* s. str.: body without irregular row of long, fine, simple setae along midline; abdominal terga I–VIII with unpaired, long, pointed protuberance close to posterior margin; patella-tibial suture reduced; posterior seta absent; wide stripe of densely situated setae instead of the regular setal row on outer side of femur and tibia.

#### Description.

**Figure 62.**
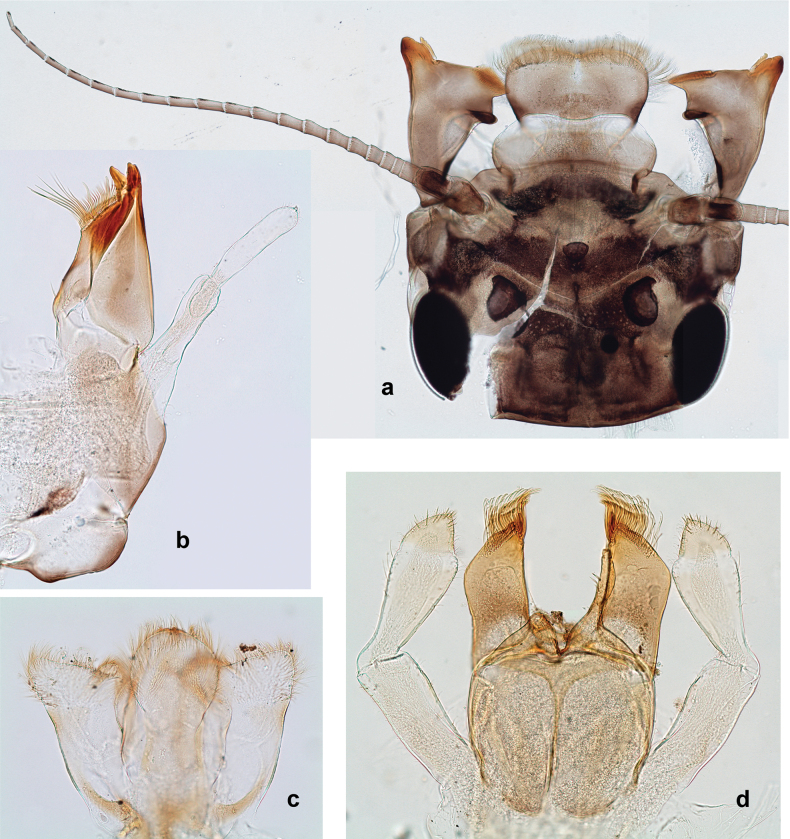
Papuanatula (Papuanatula) normungulata sp. nov., larva (holotype): **a** head **b** maxilla **c** hypopharynx **d** labium.

**Figure 63.**
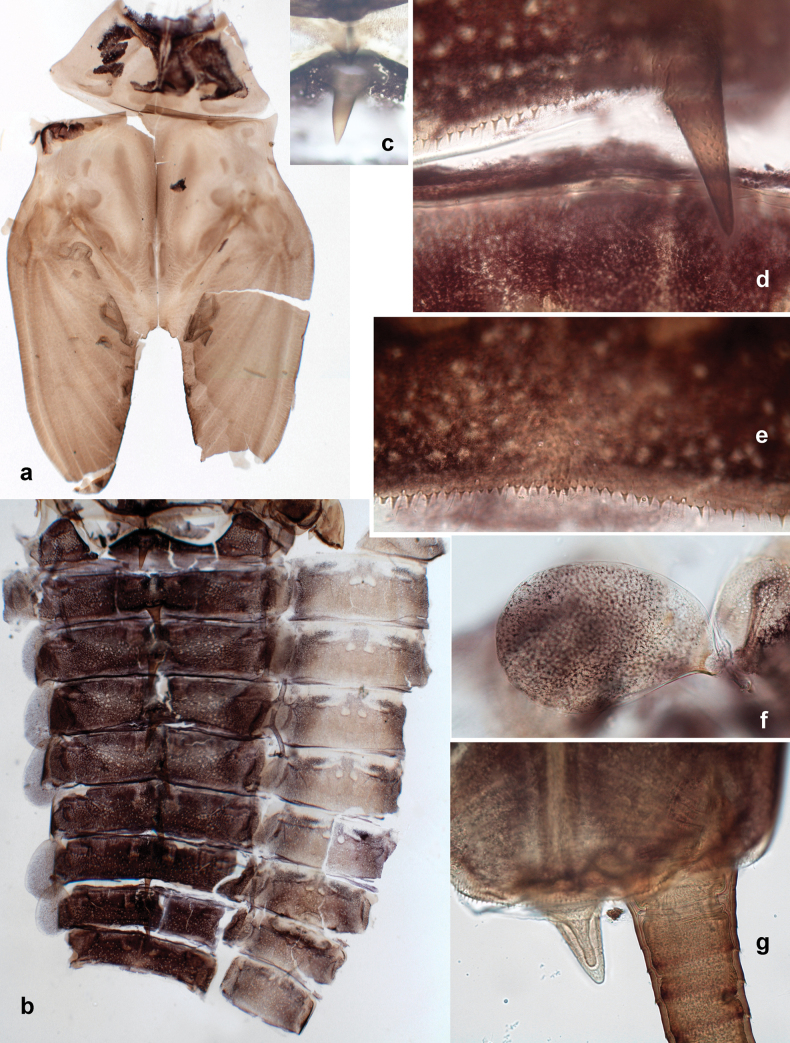
Papuanatula (Papuanatula) normungulata sp. nov., larva (holotype): larva **a** pronotum and mesonotum **b** abdomen **c** median spine on abdominal tergum VIII **d** metanotum and abdominal tergum I **e** abdominal tergum IX **f** tergalius II **h** abdominal tergum X and paracercus.

**Figure 64.**
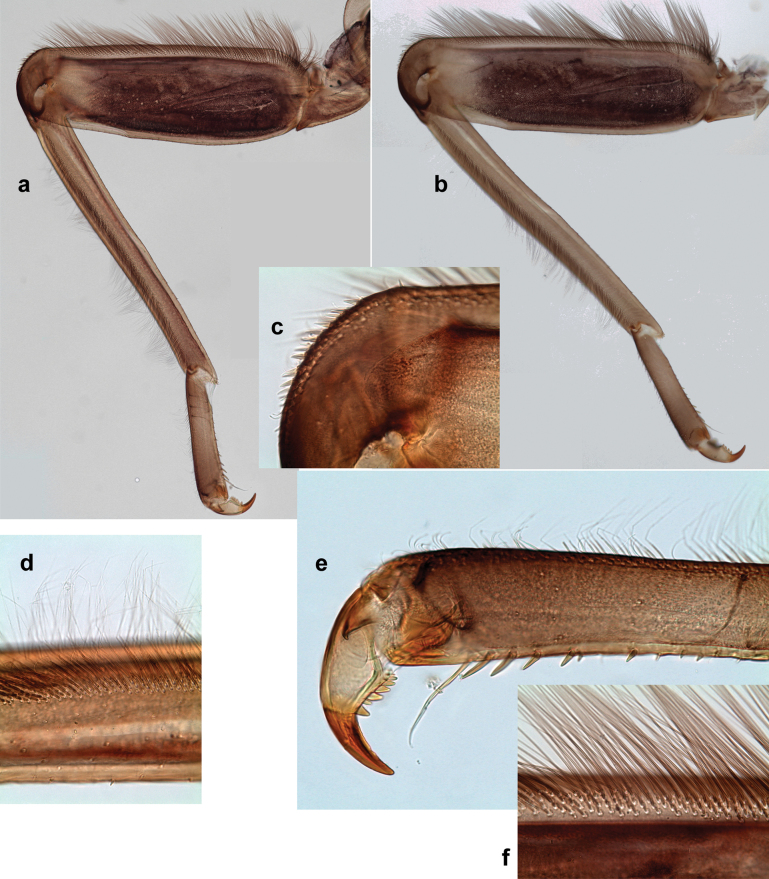
Papuanatula (Papuanatula) normungulata sp. nov., larva (holotype): larva **a, b** fore and middle legs **c** apex of femur **d** outer side of tibia **e** tarsus and claw **f** outer side of femur.

**Larva** (Figs 62–64). ***Cuticular coloration*.** Head brownish. Pronotum and mesonotum pale brownish with darker areas; fore protoptera with wide darker lines corresponding to convex veins and thin paler lines corresponding to concave veins (Figs 62a, 63a, b). Cuticle of femur mostly pale ochre-brownish, darker brown at apex. Tibia and tarsus ochre-brownish (Fig. 64a, b). Abdominal terga ochre-brownish with median spines darker brown. Cerci brow.

***Hypodermal coloration*.** In female larva with developed subimaginal wings, whole dorsal side of head, thorax, and abdomen uniformly dark brown; pleura of thorax ochre with dark brown; prosternum and mesosternum dark brown; abdominal sterna with ochre and brown areas (Figs 62a, 63a, b). On anterior side of each leg, proximal ¾ of femur entirely dark brown, distal ¼ ochre; tibia and tarsus ochre. Tissues of tergalii uniformly grey, without pigmentation associated with trachea, so tracheae poorly visible (Figs 63f, 64a, b).

***Head*. *Antenna*** (Fig. 62a). Length ~ 1.5 × head length. As typical for subgenus. ***Developing turbinate eyes in last instar male larvae*.** Unknown. ***Labrum*** (Fig. 62a) widened distally; long setae on dorsal surface numerous and forming integral, regular transverse row; each seta consists of stout stem and numerous long processes on both sides; setae and their processes intensively yellowish. ***Right mandible*** (Fig. 62a). As typical for subgenus. ***Left mandible*** (Fig. 62a). As typical for subgenus. ***Hypopharynx*** (Fig. 62c) apically evenly covered with setae-like spines. ***Maxilla*** (Fig. 62b). Maxillary palp as long as galea-lacinia. Otherwise, as typical for genus. ***Labium*** (Fig. 62d). Paraglossa with proximal 2/3 parallel-sided; three apical setal rows straight and continued by straight row on ventral side of paraglossa. Glossa as long as 3/4 of paraglossa, with slender distal portion twice longer than wide proximal portion; distal portion narrowed proximally, widened at middle, with lateral margin convex. Glossa with several long setae in distal 1/2 and with several long seta near middle of ventral side. Labial palp without distomedian projection on segment II; segment III cone-shaped, with median margin as long as lateral margin.

***Thorax*. *Sterna*.** With small protuberances on sides of prosternum and close to openings of mesothoracic and metathoracic sternal apodemes (as in Fig. 108a). ***Terga*** (Fig. 63d) without long setae on midline. Metanotum with unpaired, moderately long, pointed, spine-like protuberance close to posterior margin; without hind protoptera or their vestiges. ***Legs*** (Fig. 64a–f). Hind leg unknown. Fore femur widened in proximal part. ***Femur*.** Outer side of each femur with numerous long, pointed setae situated densely and irregularly, forming stripe of three or four setae width. Apex of femur with short, stout, pointed, spine-like setae. ***Tibia*.** Patella-tibial suture reduced, i.e., smoothed out and not crossing inner side of tibia. Tibia-tarsal condylus turned to anterior side. Anterior side of tibia with numerous long setae situated densely and irregularly, forming stripe of three to four setae width; each seta stout and brown in proximal part, hair-like and colorless in distal part. ***Tarsus*.** Anterior side of tarsus with stripe of setae similar to tibia, but smaller setae. Posterior side of each tarsus with regular row of short, stout, oval setae (looking pointed in profile) and one much longer, thinner, pointed seta distad of them. ***Claw*** with row of six or seven subequal denticles, without posterior seta.

***Abdomen*. *Terga*** (Fig. 63b–e) without long setae on midline. Each abdominal tergum I–VIII with unpaired, long, pointed, spine-like protuberance close to posterior margin. Abdominal terga I–VI without denticles on posterior margins; posterior margins of abdominal terga VII–X with small, sharply pointed denticles; some pointed denticles on surface of abdominal terga, including median spines. ***Tergalii*** (Fig. 63f) of abdominal segment I absent; tergalii II–VII oval, nearly subequal, tergalii II and VII slightly smaller than others. Each tergalius with costal and anal ribs narrow, smooth, present on proximal 1/2 of tergalius only. ***Paraproct*** with margins smooth, lacking denticles. ***Caudalii*** (Fig. 63h) without swimming setae or their vestiges. Paracercus small, conic, non-segmented.

***Pose of subimaginal gonostyli under larval cuticle*.** Unknown.

**Subimago. *Texture*.** On all legs of both sexes, each tarsomere covered mostly with blunt microlepides, with pointed microlepides near apex (as in Fig. 70i).

**Imago.** Unknown. Judging by hypodermal coloration of mature female larva, female imago has following coloration: head, thorax, and abdomen dorsally dark brown; femur of each leg pair dark brown except distal ¼.

**Egg**. Unknown.

#### Dimension.

Body length 6 mm.

#### Distribution.

Indonesia: Sulawesi Island (Fig. 147).

### ﻿﻿ Papuanatula (Papuanatula) obscura sp. nov.

Taxon classificationAnimaliaEphemeropteraBaetidae

F4D22BC4-BCBD-5ACA-8A67-42D9317FD67A

https://zoobank.org/266F555D-D44C-4ED3-BFDA-6E4B7F1E44DD

**Figure 65.**
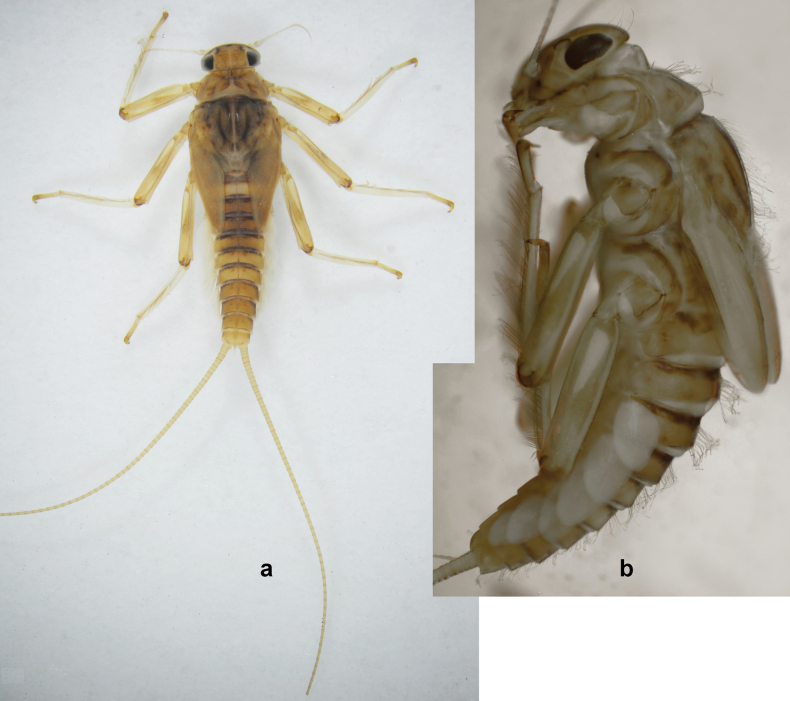
Papuanatula (Papuanatula) obscura sp. nov., larvae.

**Figure 66.**
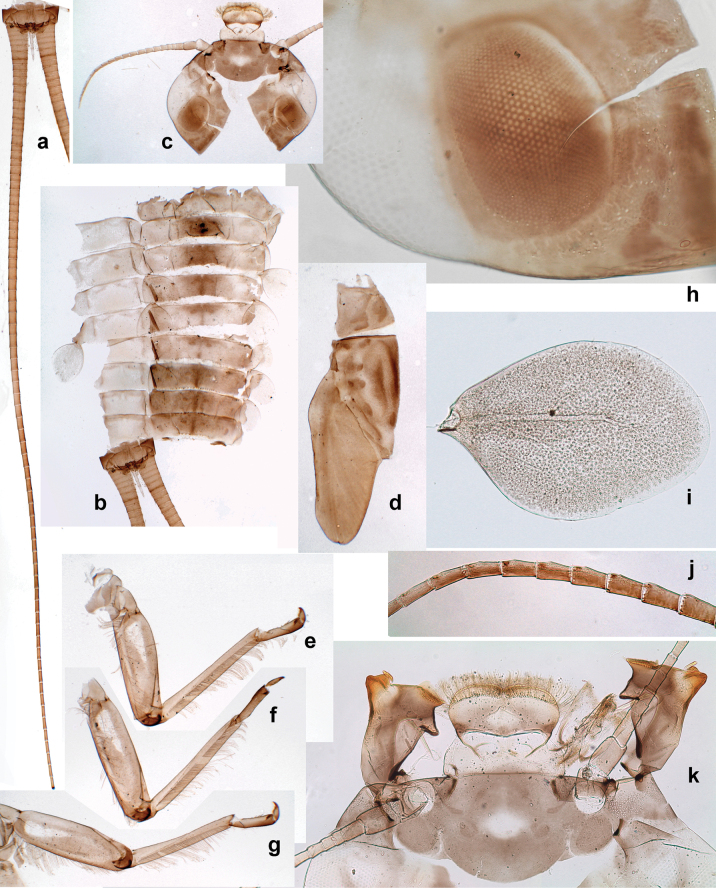
Papuanatula (Papuanatula) obscura sp. nov., larval exuviae: **a, b** abdomen with caudalii **c** head **d** half of pronotum and mesonotum **e–g** fore, middle, and hind legs **h** part of head with eye **i** tergalius III **j** flagellum of antenna **k** head with labrum, mandibles, and hypopharynx (**a–g** in one magnification, k holotype).

**Figure 67.**
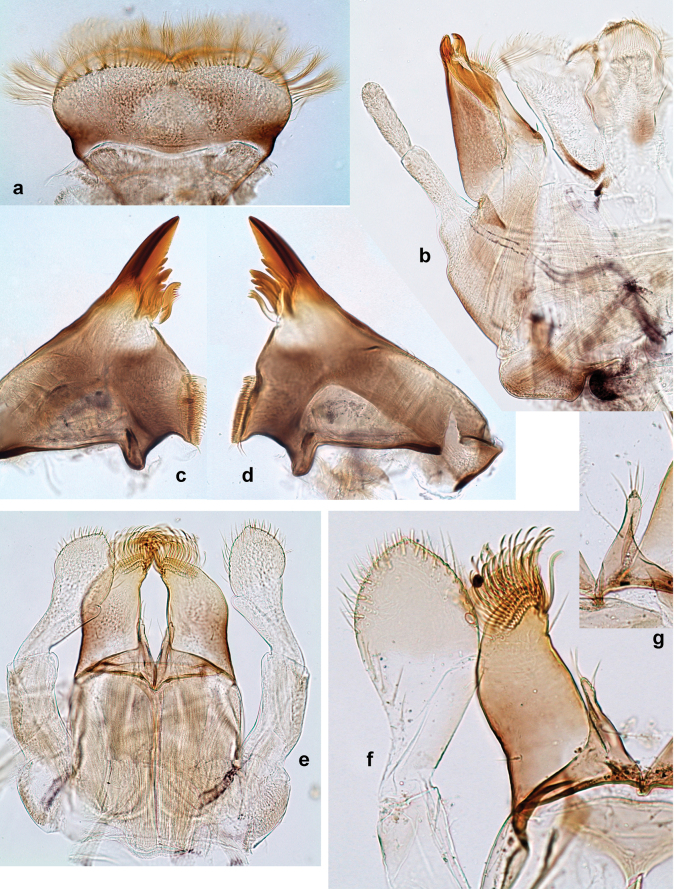
Papuanatula (Papuanatula) obscura sp. nov., larva: **a** labrum **b** maxilla and hypopharynx **c, d** left and right mandibles **e–g** labium.

**Figure 68.**
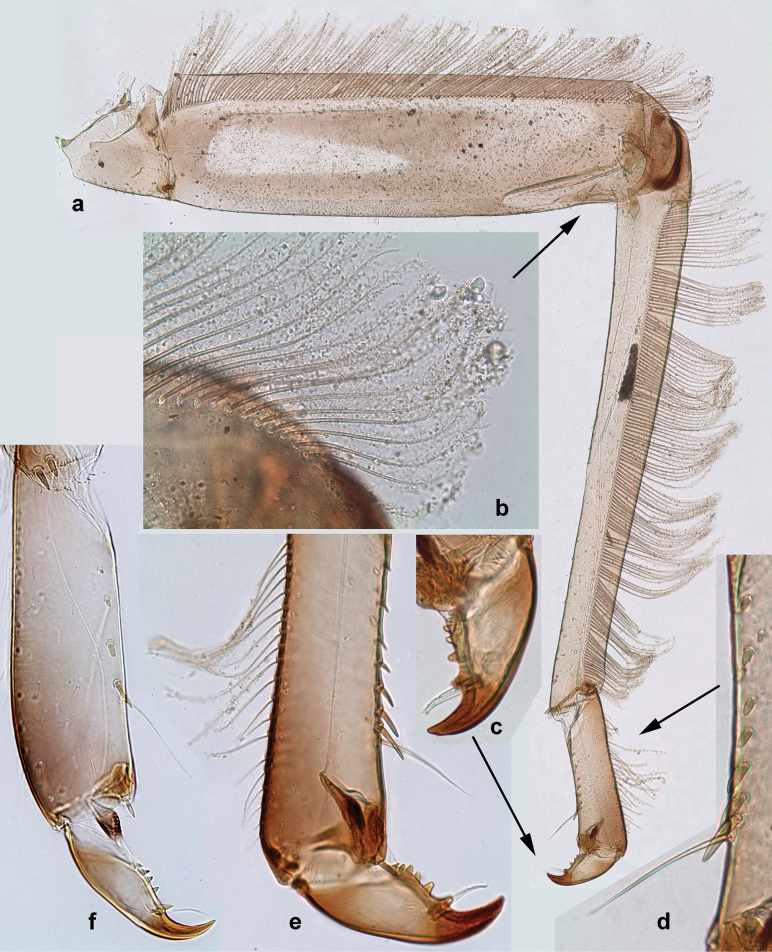
Papuanatula (Papuanatula) obscura sp. nov., larva: **a–d** middle leg and its parts **e** tarsus and claw of last instar larva **f** tarsus and claw of young larva.

**Figure 69.**
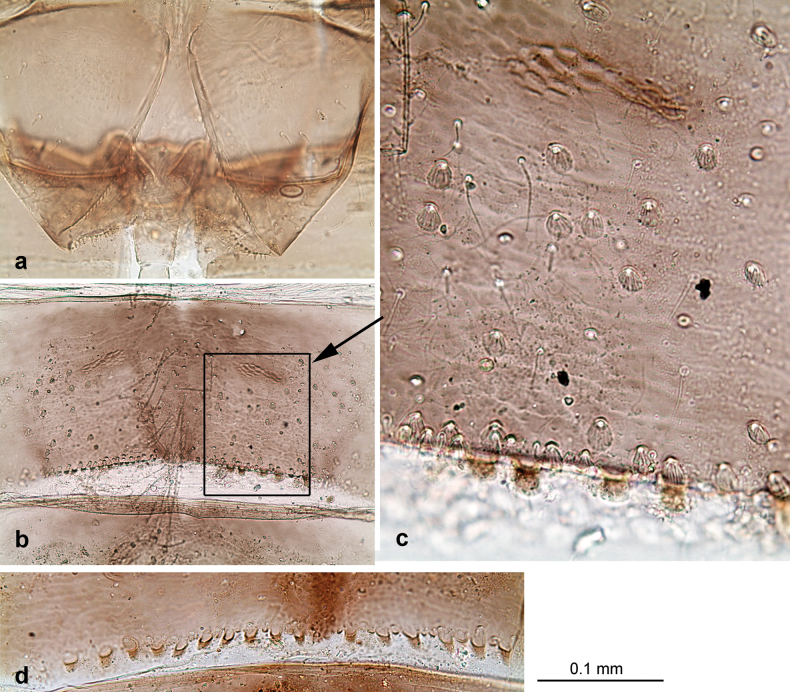
Papuanatula (Papuanatula) obscura sp. nov., larval exuviae: **a** paraprocts **b**, **c** abdominal tergum IV **d** posterior margin of tergum VII.

**Figure 70.**
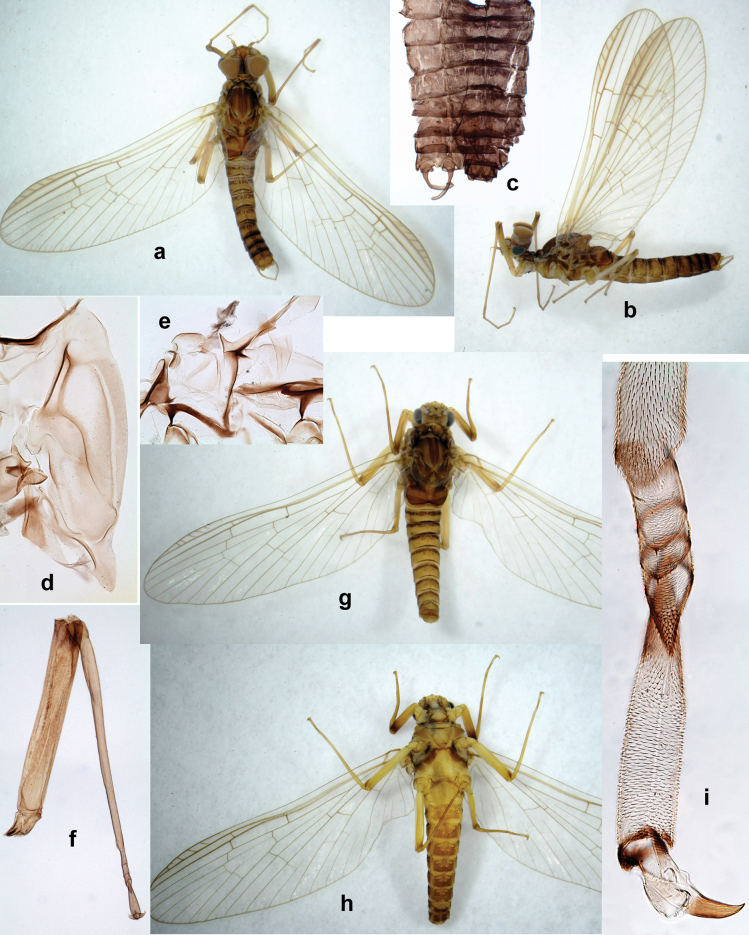
Papuanatula (Papuanatula) obscura sp. nov.: **a, b** male imago **c** abdomen of male imago **d** subimaginal exuviae of half of mesonotum **e** subimaginal exuviae of mesopleuron **f** middle leg of male imago **g, h** female imago **i** subimaginal exuviae of middle tarsus of female (**c, f** holotype).

**Figure 71.**
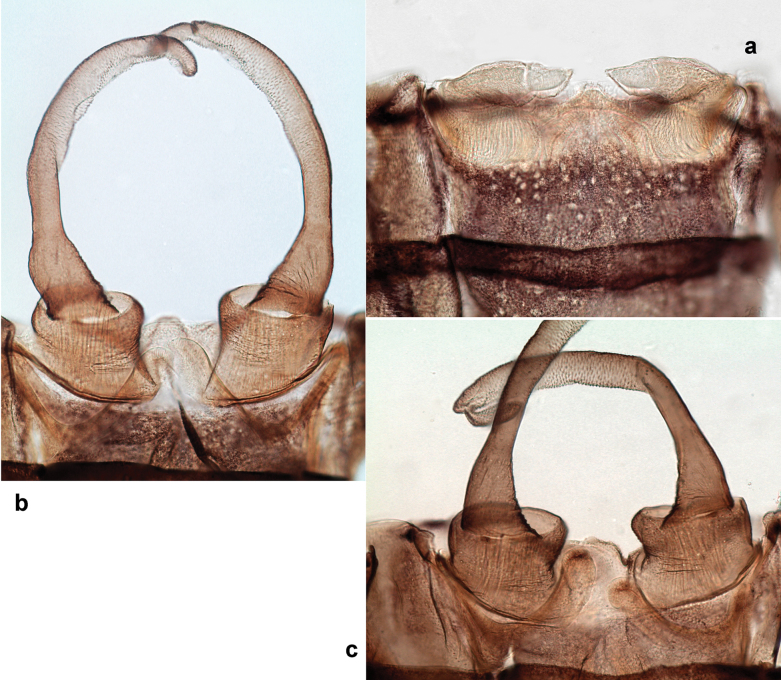
Papuanatula (Papuanatula) obscura sp. nov., male genitalia: **a** subimaginal genitalia developing under larval cuticle **b, c** imaginal genitalia (**c** holotype).

Figs 65 , 66 , 67 , 68 , 69 , 70 , 71

Papuanatula sp.: Kluge and Novikova 2014: fig. 37.

#### Etymology.

The species name *obscura* refers to the dark color of male imago (Fig. 70a, b).

#### Material examined.

***Holotype*.** L-S-I♂ {specimen number [VIII] (11) 20124}; Indonesia • Papua, Baliem valley, Wamena, river Elagaima; 19.viii.2012; coll. N. Kluge & L. Sheyko; SPbU. ***Paratypes*.** same locality and collectors; 15–19.viii.2012: 4 L-S-I♂, 5 L-S♂, 1 I♂, 2 L-S-I♀, 7 L-S♀, numerous larvae; SPbU.

#### Diagnosis.

**Larva.** The following combination of characters distinguishes *P.obscura* sp. nov. from other species of *Papuanatula* s. str.: body dorsally with irregular row of long, fine, simple setae on midline; abdominal terga without protuberances; femur with clearly outlined wedge-shaped blank on proximal 1/2; abdominal terga mostly brownish, terga I and V–VI brighter; posterior margins of abdominal terga II–IX with short, blunt denticles; tracheation of tergalii poorly visible.

#### Description.

**Larva** (Figs 65–69). ***Cuticular coloration*.** Head, pronotum, mesonotum and metanotum brownish, with darker and paler areas; fore protoptera nearly uniformly brownish (Fig. 66b–d, k). Thoracic pleura brownish, sterna mostly colorless with some areas pale brownish. Cuticle of femur mostly brownish, with clearly outlined wedge-shape blank on proximal 1/2; apex of femur bordered with darker brown (Fig. 66e–g). Tibia and tarsus mostly from colorless to pale brownish, distal end and outer side of tarsus darker brown (Fig. 66e–g). Abdominal terga mostly brownish, with lateral areas paler; terga I and V–VI more or less paler than others; sterna mostly colorless (Figs 65a, 66b). Cerci uniformly pale brownish.

***Hypodermal coloration*.** Legs without hypodermal markings. Each abdominal tergum I–IX with wide dark brown transverse band close to anterior margin and with narrower dark brown transverse band close to posterior margin (Fig. 65a). Tissues of tergalii colorless, without pigmentation associated with trachea, so tracheae poorly visible (Fig. 66i).

***Head*.** Long, fine, soft, colorless setae irregularly situated along midline (Fig. 65b). ***Antenna*** (Fig. 66c, j). Length ~ 1.5× head length. As typical for subgenus. ***Developing turbinate eyes in last instar male larva*** (Fig. 66h) with larger facets in middle and smaller facets on periphery; ovoid, rather small, with big distance to each other. ***Labrum*** (Fig. 67a) widened distally; long setae on dorsal surface numerous and forming integral, regular transverse row; each seta consists of stout stem and numerous long processes on both sides; setae and their processes intensively yellowish colored. ***Right mandible*** (Fig. 67d). As typical for subgenus. ***Left mandible*** (Fig. 67c). As typical for subgenus. ***Hypopharynx*** (Fig. 67b). As typical for genus. ***Maxilla*** (Fig. 67b). Maxillary palp as long as galea-lacinia. Otherwise, as typical for the genus. ***Labium*** (Fig. 67e–g). Paraglossae widened near middle, with lateral side forming concavity in proximal part; three apical setal rows sharply bent at apex of paraglossa. Glossa shorter than half of paraglossa, with finger-like (distal) portion as long as triangular (proximal) portion. Glossa with several long setae at apex and one long seta near middle of ventral side. Labial palp without distomedian projection on segment II; segment III with median margin longer than lateral margin.

***Thorax*. *Sterna*.** With small protuberances on sides of prosternum and close to openings of mesothoracic and metathoracic sternal apodemes (as in Fig. 108a). ***Terga*** (Fig. 65b). Long, fine, soft, colorless setae irregularly situated along midline of all terga. Metanotum without hind protoptera or their vestiges. ***Legs*** (Fig. 68a–f). Fore femur slightly widened in proximal part; hind tibia shorter than others. ***Femur*.** Outer side of each femur with single regular row of long, hair-like setae bearing numerous fine, short branches on all sides. ***Tibia*.** Patella-tibial suture present on all legs, terminated near middle of inner margin of tibia. Tibia-tarsal condylus turned to anterior side. Anterior side of each tibia with regular row of setae similar to that on femur. ***Tarsus*.** Anterior side of each tarsus with regular row of similar, but smaller (shorter and narrower) setae. Posterior side of each tarsus with regular row of short, stout, oval setae (looking pointed in profile) and one much longer, thinner, pointed seta distad of them. ***Claw*** with row of six short denticles and one somewhat larger denticle distad of them; one long, arched posterior setae (Fig. 68e); occasionally 2 such setae (Fig. 68c).

***Abdomen*. *Terga*** (Figs 65b, 69b–d). Long, fine, soft, colorless setae irregularly situated along midline of all abdominal terga. Abdominal terga without dorsal unpaired or paired protuberances, only with slightly expressed paired, submedian elevations. Abdominal terga with small, roundish scales with small sockets and radial striation. Posterior margins of abdominal terga II–IX with short, blunt denticles. Posterior margin of tergum X with smaller, blunt denticles. ***Tergalii*** (Fig. 66i) of abdominal segment I absent; tergalii II–VII subequal, oval. Each tergalius with costal and anal ribs narrow, smooth, present on proximal 1/2 of tergalius only. ***Paraproct*** (Fig. 69a) with posterior prolongation bent toward bases of caudalii, with many small, equal denticles on median-posterior margin (Kluge and Novikova 2014: fig. 37). ***Caudalii*** (Fig. 66a) without swimming setae; vestiges of swimming setae present on distal part of cerci. Paracercus short, consisting of ~ 6–8 segments.

***Pose of subimaginal gonostyli under larval cuticle*** (Fig. 71a). In mature larva ready to molt to subimago, subimaginal gonostyli packed under larval cuticle in “*Labiobaetis*-type” pose: 2^nd^ segments directed medially and bent proximally; 3^rd^ segment directed medially (as continuation of 2^nd^ segment) and narrowed apically, being deformed corresponding to space between subimaginal styliger and larval cuticle.

**Subimago. *Cuticular coloration*.** Pronotum and prosternum partly brown (as in Fig. 60f). Mesonotum pale brown with medioparapsidal suture colorless, other sutures darker brown (Fig. 70d). Meso- and metathoracic pleura and sterna with colorless, pale brownish and dark brown areas (Fig. 70e). Cuticle of wings colorless, with microtrichiae brownish. Legs nearly colorless, with pale brown bordering on femur and base of tibia (as in Fig. 36d). Abdomen diffusely colored with very pale brownish, mostly in distal part. Cerci colorless with setae brown.

***Hypodermal coloration*.** As in imago.

***Texture*.** (Fig. 70i). On all legs of both sexes, each tarsomere covered mostly with blunt microlepides, with pointed microlepides near apex.

**Imago. *Imago, male*** (Fig. 70a–c, f). Head dark brown. Antennae ochre. Turbinate eyes dark brown, high and narrow, cylindrical, with faceted surfaces round and widely separated. Thorax brown, equally dark dorsally, laterally, and ventrally. Fore wing with membrane mostly colorless, area of pterostigma slightly tinged with brownish; veins intensively ochre-brownish. Pterostigma with 5–10 oblique cross veins (Fig. 70a). Fore leg mostly pale brown, middle and hind legs mostly ochre; femur of each leg with brown apex (Fig. 70f). Abdominal coloration similar to that of female and larva, but darker: all terga and sterna brown, each tergum I–IX with wide darker brown transverse band close to anterior margin and with narrower dark brown transverse band close to posterior margin (Fig. 70a–c). Cerci brown.

***Genitalia*** (Fig. 71b, c). Sterno-styligeral muscle absent. Each unistyliger slightly widened apically, with median margin concave and apex thickened. At lateral side of gonostylus, its 1^st^ segment roundly-convex at apex and separated from 2^nd^ segment by concavity; at median side of gonostylus, 1^st^ segment more gradually turns to 2^nd^ segment. Second segment equally wide along its length. Third (terminal) segment of gonostylus nearly as wide as 2^nd^, with length twice exceeding width. Penial bridge with poorly expressed membranous projection between unistyligers. Each gonovectis semicircular, with lateral (basal) and median (apical) portions equally long, apex bent medially.

***Imago, female*** (Fig. 70g, h). Head and thorax dorsally dark brown, ventrally mostly ochre. Hypodermal abdominal coloration as in larva: mostly ochre, each tergum I–IX with wide dark brown transverse band close to anterior margin and with narrower dark brown transverse band close to posterior margin. Coloration of legs, wings, and cerci as in male.

**Egg** (Fig. 37d, e). Irregularly oval. Chorion without regular relief.

#### Dimension.

Fore wing length (and approximate body length): male 5.5 mm, female 7 mm.

#### Distribution.

New Guinea (Fig. 147).

### ﻿﻿ Papuanatula (Papuanatula) obscurella sp. nov.

Taxon classificationAnimaliaEphemeropteraBaetidae

A4617B4D-05B9-5EF2-8088-8D8E2A29A34B

https://zoobank.org/8972A7CB-19B4-4736-9542-170B47C3A4B3

**Figure 72.**
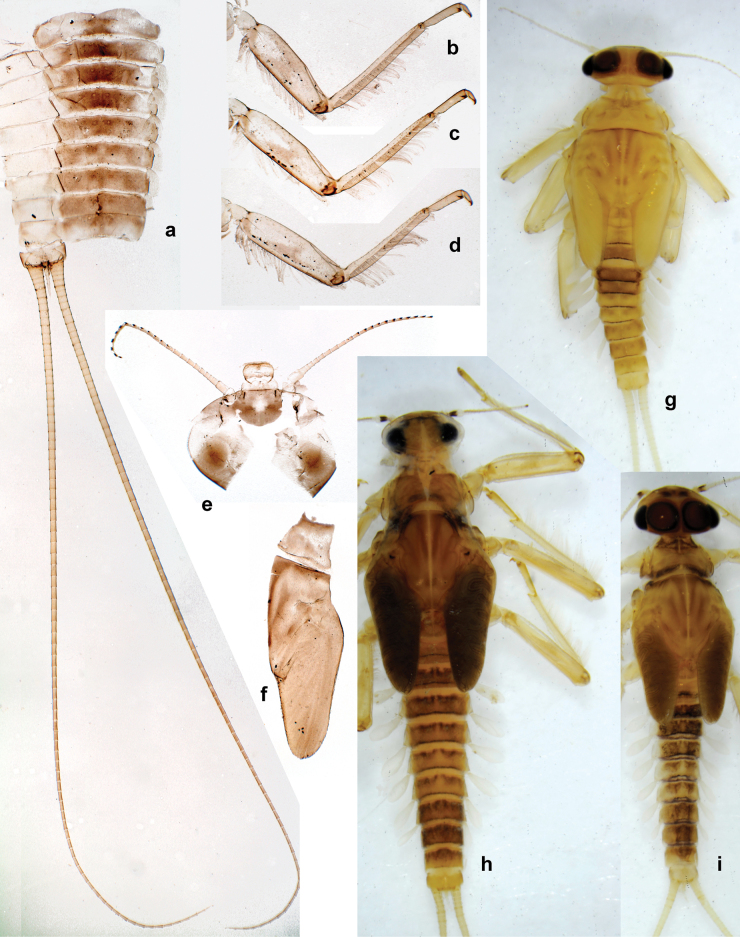
Papuanatula (Papuanatula) obscurella sp. nov., larva: **a–f** larval exuviae (holotype) **a** abdomen **b–d** fore, middle, and hind legs **e** head **f** half of pronotum and mesonotum **g** male larva **h, i** female and male larvae with hypodermal subimaginal coloration of abdomen visible through larval cuticle.

**Figure 73.**
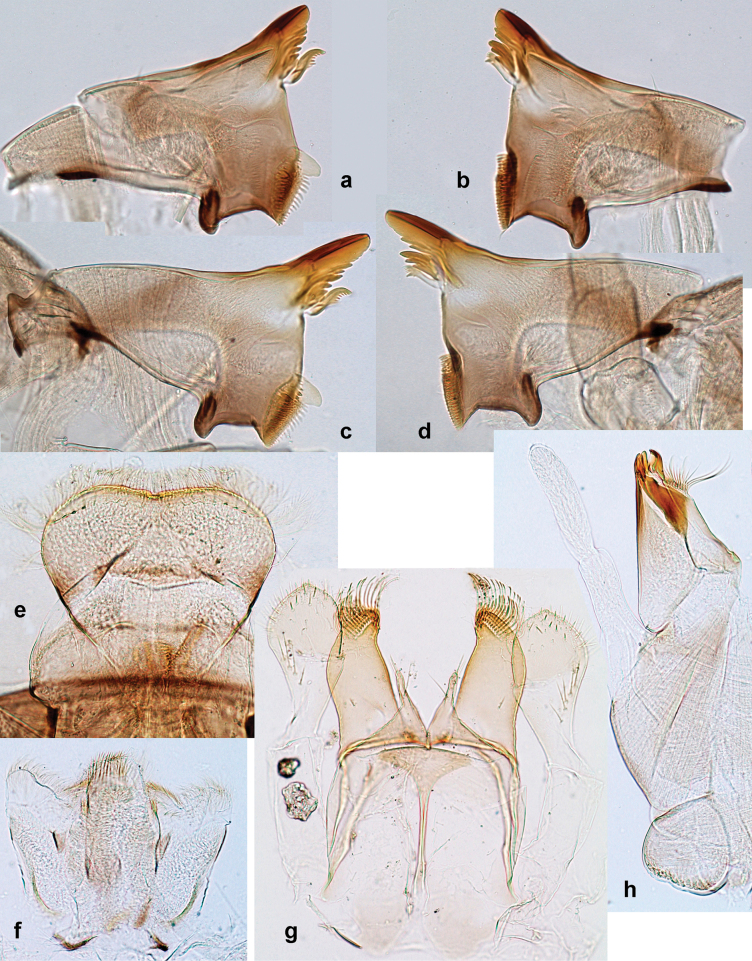
Papuanatula (Papuanatula) obscurella sp. nov., larva: **a, b** left and right mandibles of penultimate instar with mandibles of last instar developing inside **c, d** left and right mandibles with complete incisors **e** labrum **f** hypopharynx with superlinguae **g** labium **h** maxilla (**g** holotype).

**Figure 74.**
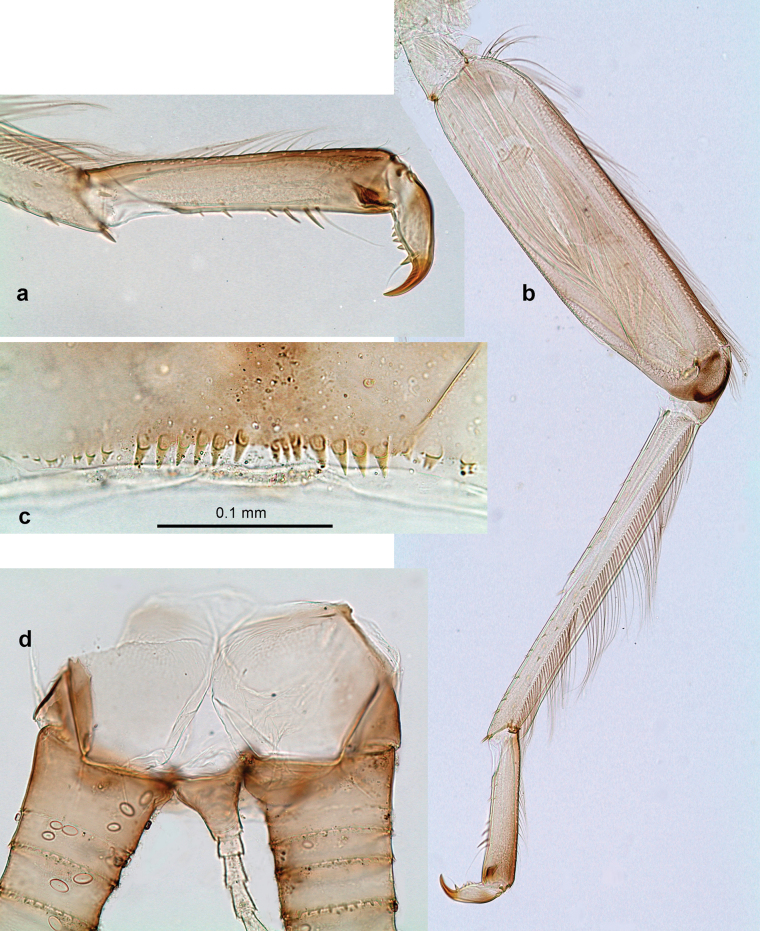
Papuanatula (Papuanatula) obscurella sp. nov., larva: **a** fore tarsus and claw **b** hind leg **c** tergum IX **d** paraprocts (**c** holotype).

**Figure 75.**
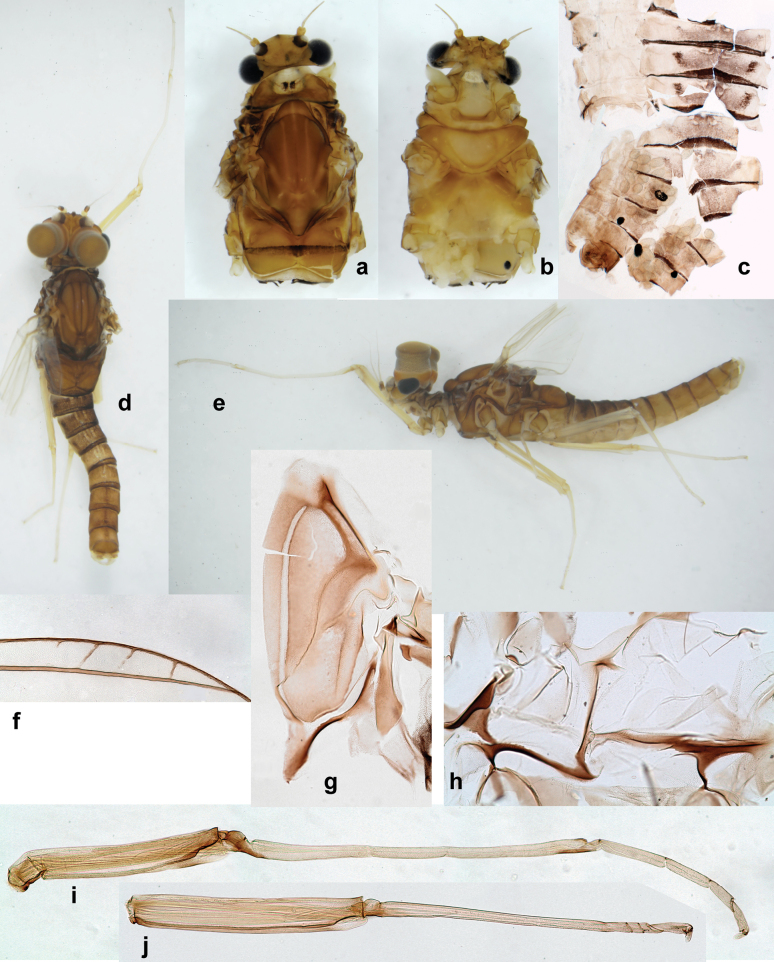
Papuanatula (Papuanatula) obscurella sp. nov.: **a, b** head and thorax of female imago dorsally and ventrally **c** abdomen of male imago **d, e** male imago **f** pterostigma **g** subimaginal exuviae of half of mesonotum **h** subimaginal exuviae of mesopleuron **I, j** fore and hind legs (**c–f, I, j** holotype).

**Figure 76.**
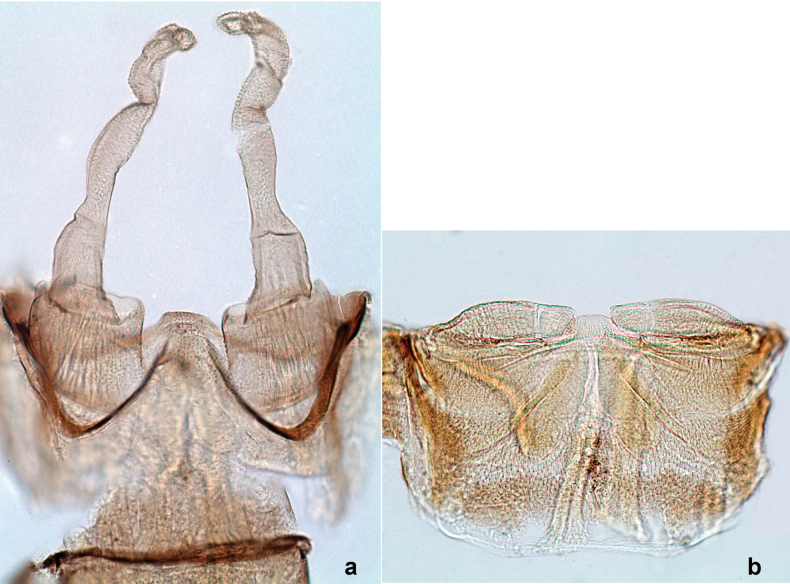
Papuanatula (Papuanatula) obscurella sp. nov., male genitalia: **a** genitalia of imago **b** subimaginal genitalia developing under larval cuticle (**a** holotype).

Figs 72 , 73 , 74 , 75 , 76

#### Etymology.

The species name *obscurella* refers to the dark color of male imago (Fig. 75d).

#### Material examined.

***Holotype*.** L-S-I♂ {specimen number [VIII(5)B2012}; Indonesia • Papua, Baliem valley, Wamena, river Elagaima; 19.viii.2012; coll. N. Kluge & L. Sheyko; SPbU. ***Paratypes*.** same locality and collectors, 15–19.viii.2012: 2 L-S♂, 1 L-S-I♀, 17 larvae; SPbU.

#### Diagnosis.

**Larva.** The following combination of characters distinguishes *P.obscurella* sp. nov. from other species of *Papuanatula* s. str.: body without long, fine, simple setae along midline; abdominal terga without protuberances; femur without hypodermal pigmentation; abdominal terga with pointed denticles on posterior margins; tergalii with non-pigmented tracheae; abdomen with hypodermal coloration (brown band on posterior margins of terga I–IX); turbinate eyes with diminished facetted surfaces.

#### Description.

**Larva** (Figs 72–74). ***Cuticular coloration*.** Head, pronotum, mesonotum and metanotum brownish, with darker and paler areas; fore protoptera nearly uniformly brownish (Fig. 72a, e, f). Thoracic pleura brownish, sterna mostly colorless. Cuticle of femur with wedge-shape colorless blank on proximal 1/2 and colorless blank occupying most part of distal 1/2; other surface of femur brownish, apex bordered with darker brown (Fig. 72b–d). Tibia and tarsus from colorless to pale brownish (Fig. 72b–d). Abdominal terga mostly brownish, with lateral areas paler; terga V–VI more or less paler than others; sterna mostly colorless (Fig. 72a). Cerci uniformly pale brownish.

***Hypodermal coloration*.** Legs without hypodermal markings. Hypodermal coloration of abdomen either non-expressed or represented by dark brown transverse band close to posterior margin of each tergum I–IX, sometime with other brown markings on abdominal terga (Fig. 72h, i). Tissues of tergalii colorless, without pigmentation associated with trachea, so tracheae poorly visible (Fig. 72g–i).

***Head*. *Antenna*** (Fig. 72e). Length ~ 2× head length. As typical for subgenus. ***Developing turbinate eyes of last instar male larva*** (Fig. 72e) with larger facets in middle and smaller facets on periphery. ***Labrum*** (Fig. 73e) widened distally; long setae on dorsal surface numerous and forming integral, regular transverse row; each seta consists of stout stem and numerous long processes on both sides. ***Right mandible*** (Fig. 73b, d). As typical for subgenus. ***Left mandible*** (Fig. 73a, c). As typical for subgenus. ***Hypopharynx*** (Fig. 73f). As typical for genus. ***Maxilla*.** (Fig. 73h). Maxillary palp as long as galea-lacinia. Otherwise, as typical for genus. ***Labium*.** (Fig. 73g) Paraglossae widened near middle, with lateral side forming concavity in proximal part; three apical setal rows sharply bent at apex of paraglossa. Glossa shorter than half of paraglossa, with finger-like (distal) portion as long as triangular (proximal) portion. Glossa with several long setae at apex and one long seta near middle of ventral side. Labial palp without distomedian projection on segment II.

***Thorax*. *Sterna*.** Without protuberances. ***Terga*** without protuberances; without long, fine setae on midline. Metanotum without hind protoptera or their vestiges. ***Legs*** (Figs 72b–d, 74a, b). Fore femur slightly widened in proximal part; hind tibia shorter than others. ***Femur*.** Outer side of each femur with single regular row of long, hair-like setae bearing numerous fine, short branches on all sides (as in Figs 41g, 68b). ***Tibia*.** Patella-tibial suture present on all legs, terminated near middle of inner margin of tibia. Tibia-tarsal condylus turned to anterior side. Anterior side of each tibia with regular row of setae similar to that on femur. ***Tarsus*.** Anterior side of each tarsus with regular row of similar, but smaller (shorter and narrower) setae. Posterior side of each tarsus with regular row of short, stout, oval setae (looking pointed in profile) and one much longer, thinner, pointed seta distad of them. ***Claw*** with row of four or five short denticles and one somewhat larger denticle distad of them; long, arched posterior seta.

***Abdomen*. *Terga*** (Figs 72a, 74c) without dorsal unpaired or paired protuberances, only with slightly expressed, unpaired, median elevations; without long, fine setae on midline. Abdominal terga and sterna without scales. Posterior margins of abdominal terga I–IX with short denticles, short and blunt on several anterior terga, longer and pointed on several posterior terga. Posterior margin of tergum X with very small denticles. ***Tergalii*** (Fig. 72a, g–i) of abdominal segment I absent; tergalii II–VII subequal. Each tergalius with costal and anal ribs narrow, smooth, present on proximal 1/2 of tergalius only (as in Fig. 66i). ***Paraprocts*** (Fig. 74d) without posterior projection; margins membranous, smooth, lacking denticles. ***Caudalii*** (Fig. 72a) without swimming setae or their vestiges. Paracercus short, consisting of ~ 6–8 segments.

***Pose of subimaginal gonostyli under larval cuticle*** (Fig. 76b). In mature larva ready to molt to subimago, subimaginal gonostyli packed under larval cuticle in “*Labiobaetis*-type” pose: 2^nd^ segments directed medially and bent proximally; 3^rd^ segment directed medially (as continuation of 2^nd^ segment) and narrowed apically, being deformed corresponding to space between subimaginal styliger and larval cuticle.

**Subimago. *Cuticular coloration*.** Pronotum and prosternum partly brown (as in Fig. 60f). Mesonotum pale brown with medioparapsidal suture colorless, other sutures darker brown (Fig. 75g). Meso- and metathoracic pleura and sterna with colorless, pale brownish and dark brown areas (Fig. 75h). Cuticle of wings colorless, with microtrichiae brownish. Legs nearly colorless, with pale brown bordering on femur and base of tibia (as in Fig. 36d). Abdomen diffusely colored with very pale brownish, mostly in distal part. Cerci colorless with setae brown.

***Hypodermal coloration*.** As in imago.

***Texture*.** On all legs of both sexes, each tarsomere covered mostly with blunt microlepides, with pointed microlepides near apex (as in Fig. 70i).

**Imago. *Imago, male*** (Fig. 75c–f). Head dark brown. Antennae with scape ochre, pedicel brown, flagellum ochre. Turbinate eyes dark brown, high and narrow, cylindrical, with faceted surfaces round and widely separated. Thorax brown, equally dark dorsally, laterally, and ventrally. Fore wing with membrane colorless, veins ochre. Pterostigma with three or four incomplete, oblique cross veins (Fig. 75f). Legs ochre (Fig. 75i, j). Abdominal terga brown, each tergum I–IX with darker brown transverse band close to posterior margin; sterna slightly paler, ochre-brown (Fig. 75c–e). Cerci brown.

***Genitalia*** (Fig. 76a): Sterno-styligeral muscle absent. Each unistyliger parallel-sided, equally wide at base and at apex. At lateral side of gonostylus, its 1^st^ segment roundly-convex at apex and separated from 2^nd^ segment by concavity; at median side of gonostylus, 1^st^ segment gradually turns to 2^nd^ segment. Second segment equally wide all over its length. 3^rd^ (terminal) segment of gonostylus nearly as wide as 2^nd^, with length slightly exceeding width. Penial bridge with truncated trapezoid projection between unistyligers. Gonovectes dark brown. Each gonovectis parabolic, with lateral (basal) and median (apical) portions equally long, apex slightly bent medially.

***Imago, female*** (Fig. 75a, b). Head and thorax dorsally ochre-brown, ventrally mostly ochre. Abdomen mostly ochre, each tergum I–IX with dark brown transverse band close to posterior margin. Coloration of legs, wings, and cerci as in male.

**Egg** (Fig. 37c). Irregularly oval. Chorion without regular relief.

#### Dimension.

Fore wing length (and approximate body length): male 4.5 mm, female 6 mm.

#### Comparison.

Larva of *P.obscurella* sp. nov. resembles *P.plana* by absence of hypodemal pigmentation on femora, absence of tubercles and long setae on abdominal terga, pointed denticles on posterior margins of abdominal terga and non-pigmented tracheae of tergalii. At the same time, male larva and male imago of *P.obscurella* sp. nov. differ from *P.plana* by abdominal hypodermal coloration (which is represented by brown band equally developed on posterior margins of each tergum I–IX in *P.obscurella* sp. nov. vs brown spot on tergum IV in *P.plana*) and by shape of turbinate eyes (with diminished faceted surfaces in *P.obscurella* sp. nov. vs wide faceted surfaces in *P.plana*).

#### Distribution.

New Guinea (Fig. 147).

### ﻿﻿ Papuanatula (Papuanatula) parabessa sp. nov.

Taxon classificationAnimaliaEphemeropteraBaetidae

D638F3D2-F20A-5E38-8658-0FBAF1717051

https://zoobank.org/BAA28880-F87F-46CF-90D2-2DB1CB2BB547

**Figure 77.**
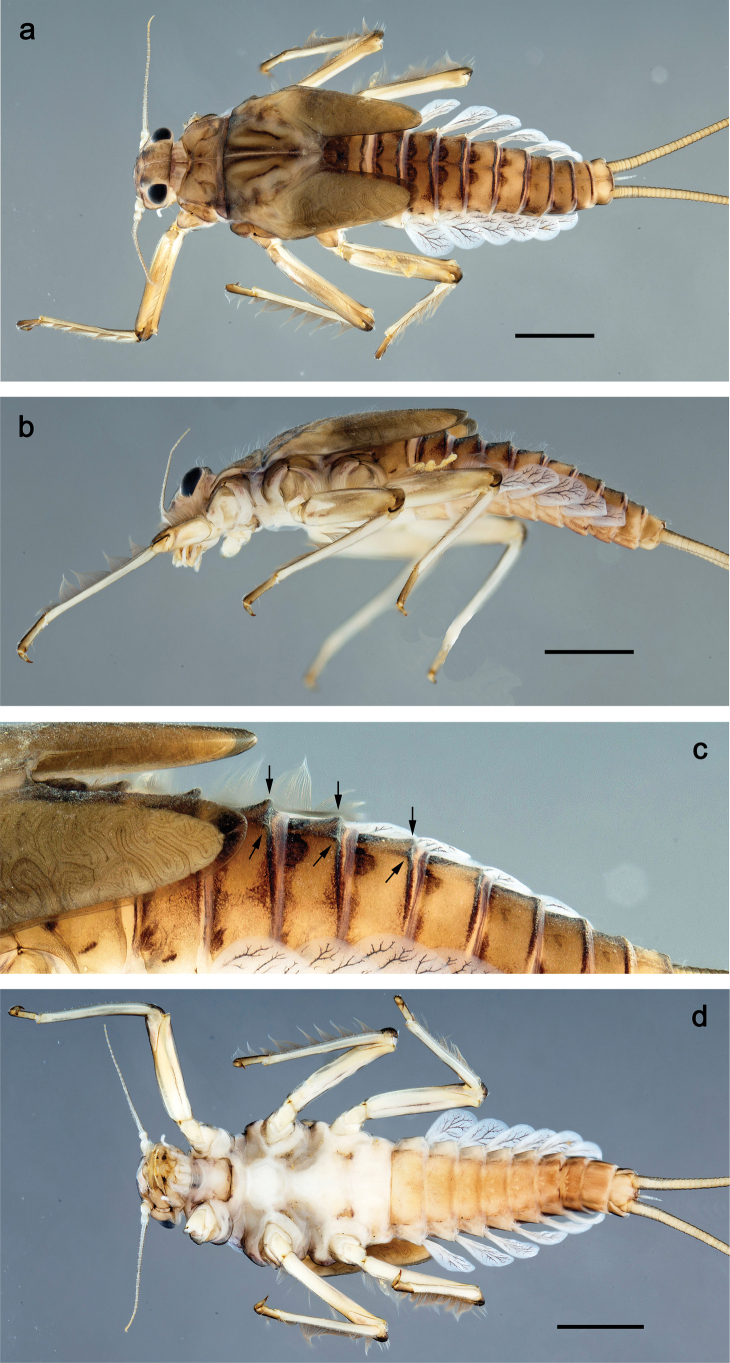
Papuanatula (Papuanatula) parabessa sp. nov., larva, habitus: **a** dorsal view **b** lateral view **c** dorsolateral view (arrows: paired humps) **d** ventral view. Scale bars: 1 mm.

**Figure 78.**
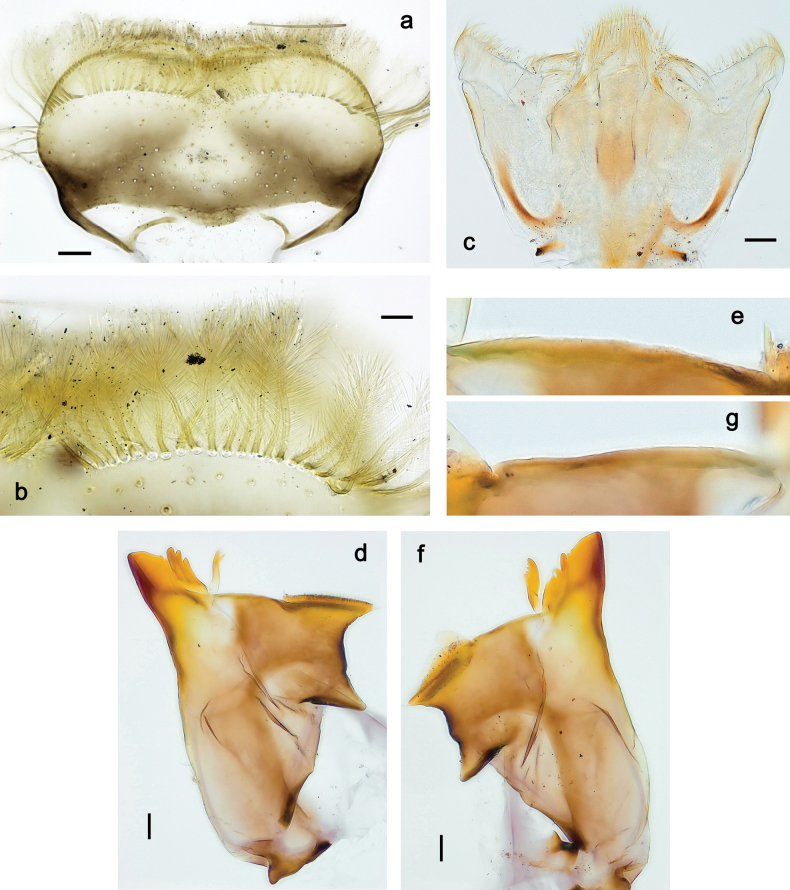
Papuanatula (Papuanatula) parabessa sp. nov., larva: **a** labrum **b** labrum: dorsal, submarginal setae **c** hypopharynx and superlinguae **d** right mandible **e** right mandible: margin between prostheca and mola **f** left mandible **g** left mandible: margin between prostheca and mola. Scale bars: 20 µm (**a, c, d, f**); 10 µm (**b**).

**Figure 79.**
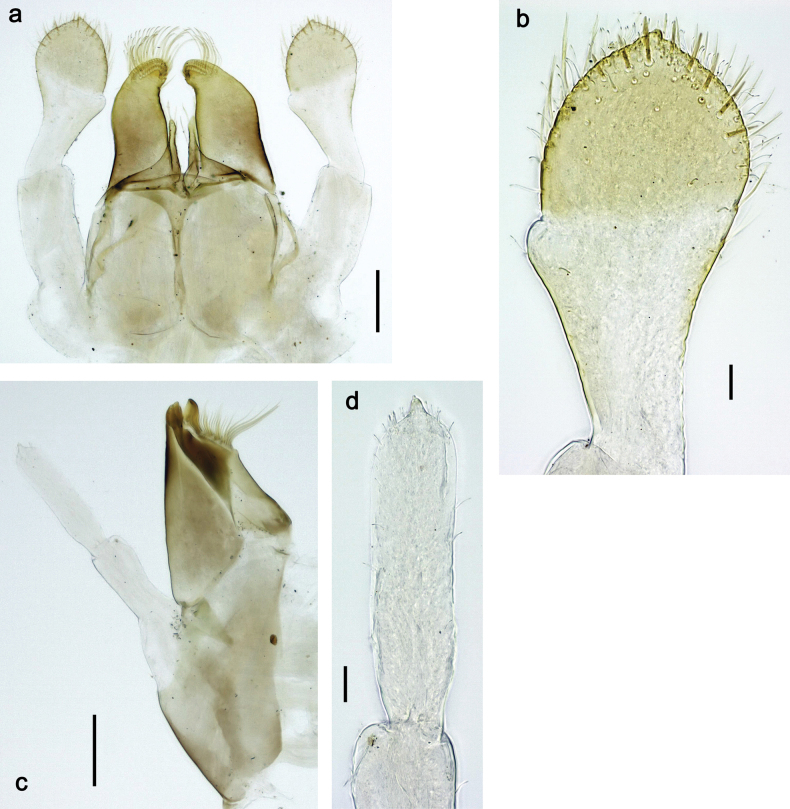
Papuanatula (Papuanatula) parabessa sp. nov., larva: **a** labium **b** labial palp **c** maxilla **d** maxillary palp. Scale bars: 50 µm (**a, c**); 10 µm (**b, d**).

**Figure 80.**
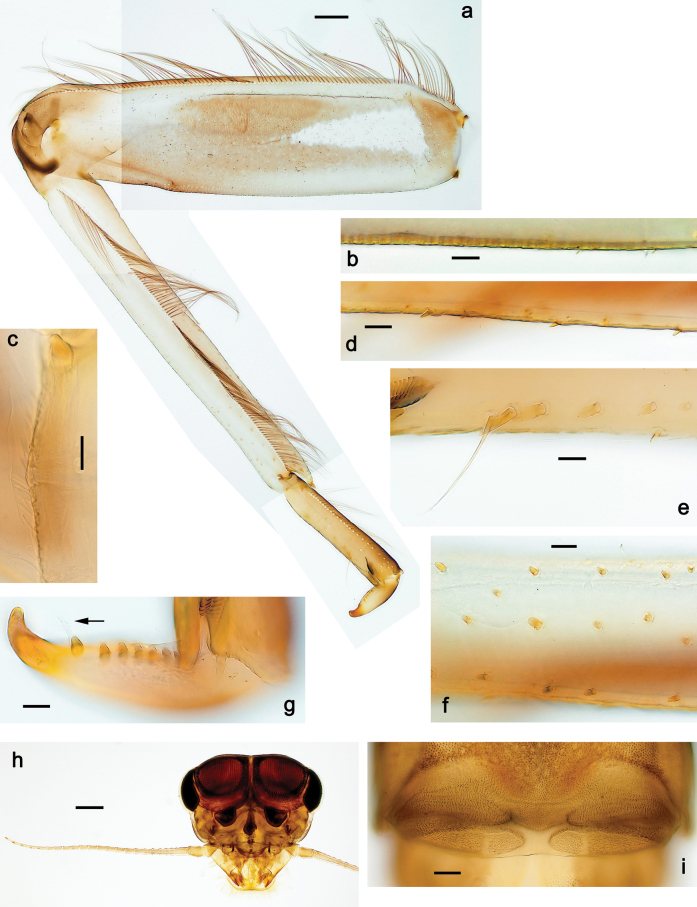
Papuanatula (Papuanatula) parabessa sp. nov., larva: **a** hind leg **b** hind femur: ventral margin **c** hind femur: posterior apex **d** hind tibia, ventral margin **e** hind tarsus: ventral margin **f** hind tibia: posterior surface **g** fore claw **h** male head, mature **i** developing gonostyli. Scale bars: 50 µm (**a**); 10 µm (**b–g, i**); 100 µm (**h**).

**Figure 81.**
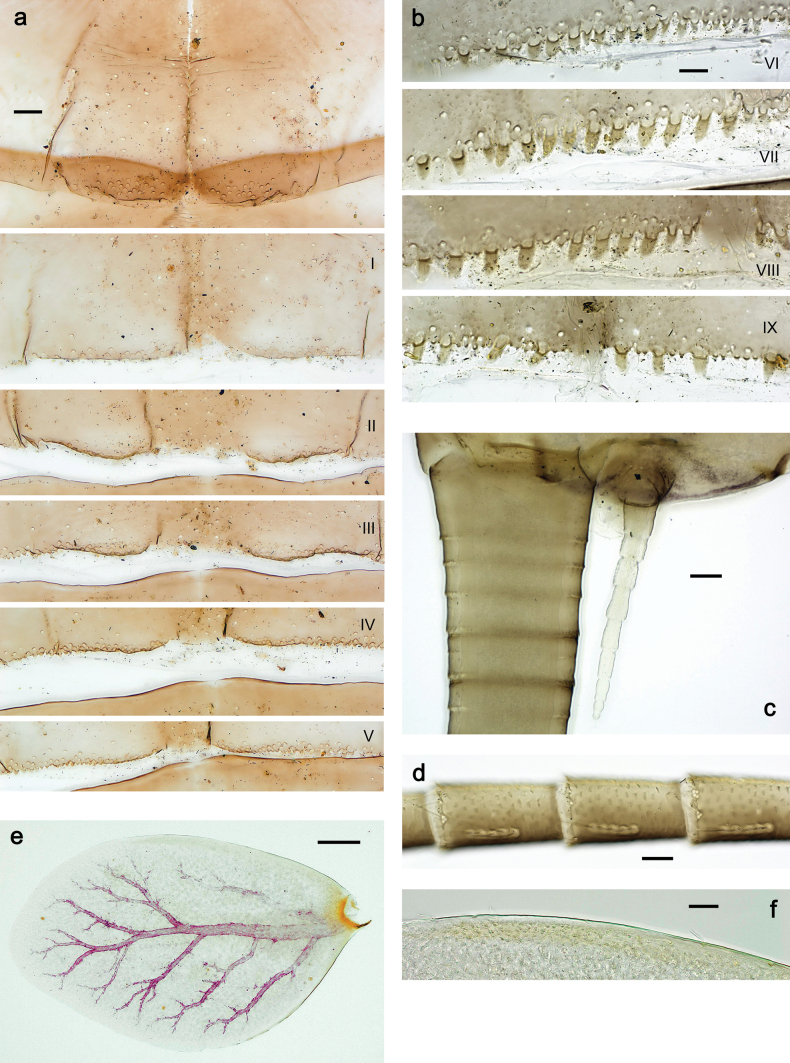
Papuanatula (Papuanatula) parabessa sp. nov., larva: **a** metanotum and abdominal terga **b** abdominal terga **c** paracercus **d** cercus **e** tergalius IV **f** tergalius IV, margin. Scale bars: 20 µm (**a, c**); 10 µm (**b, d, f**); 50 µm (**e**).

**Figure 82.**
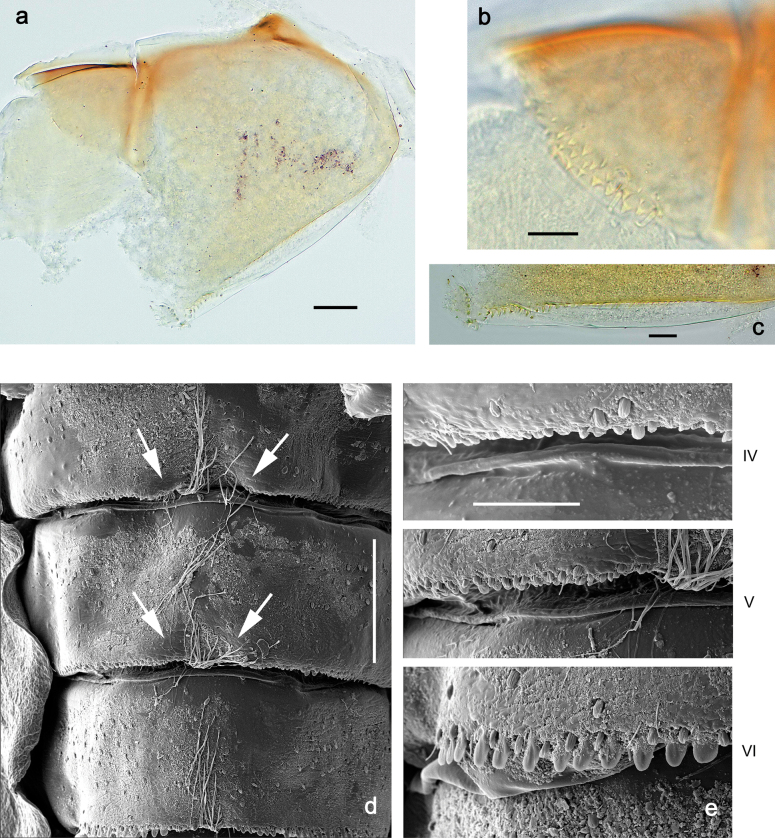
Papuanatula (Papuanatula) parabessa sp. nov., larva: **a** paraproct **b** cercotractor **c** paraproct, distal margin **d** abdominal terga IV–VI (SEM; arrows: paired humps) **e** abdominal terga (SEM). Scale bars: 20 µm (**a**); 10 µm (**b, c**); 200 µm (**d**); 50 µm (**e**).

Figs 77 , 78 , 79 , 80 , 81 , 82

#### Etymology.

The species name *parabessa* refers to the morphological similarity with *P.bessa*.

#### Material examined.

***Holotype*.** Papua New Guinea • larva; Madang Prov., Simbai area; 05°12'42"S, 144°35'31"E; 1800–2400 m; 8.iii.2007; leg. Kinibel; (PNG 151); on slide; GBIFCH00592628; ZSM/SNSB. ***Paratypes*.** 14 larvae; same data as holotype; 3 on slides; GBIFCH00976134, GBIFCH00976137, GBIFCH01221772; MZL; 11 in alcohol; GBIFCH00975778, GBIFCH00976055, GBIFCH00976068, GBIFCH00976135, GBIFCH00976136, GBIFCH00976138; MZL • 2 larvae; Papua New Guinea; Madang Prov., Simbai area, 05°13'23"S, 144°37'17"E; 1200 m, 10.iii.2007; leg. Kinibel; (PNG 152); in alcohol; GBIFCH00975776, GBIFCH00976133; MZL • 3 larvae; Enga Prov., Wapanamanda; 05°38'06"S, 143°55'20"E; 1500 m; 6.xii.2006; leg. M. Balke & Kinibel; (PNG 128); in alcohol; GBIFCH00975773, GBIFCH00976128; MZL • 8 larvae; Eastern Highlands Prov., Akameku - Brahmin, Bismarck Range; near 05°52'45"S, 145°23'13"E; 1200 m; 24.xi.2006; leg. M. Balke & Kinibel; (PNG 110); in alcohol; GBIFCH00975782, GBIFCH00976091; MZL.

#### Diagnosis.

**Larva**. The following combination of characters distinguishes *P.parabessa* sp. nov. from other species of *Papuanatula* s. str.: body dorsally with row of long, fine, simple setae along midline; metanotum and abdominal terga I–V with medioposterior, broad, paired humps; abdominal terga II–VI with paired, semicircular, dark brown markings; femur grey-brown, basally with wedge-shaped blank, dorsally with submarginal, dark grey-brown streak; paracercus with nine segments.

#### Description.

**Larva** (Figs 77–82). Body length 4.2–5.8 mm, cerci much longer than body length (~ 2×).

***Cuticular coloration*** (Fig. 77a–d) Head and thorax dorsally grey-brown, with complex pattern. Abdomen dorsally brown, terga V, VI, and X pale brown; terga II–VI with paired, semicircular, dark brown markings; terga VII–IX with small, paired, dark brown spots. Femur grey-brown, basally with wedge-shaped blank, dorsally with submarginal, dark grey-brown streak. Tibia pale grey-brown; tarsus brown. Head, thorax and abdominal segment I ventrally ecru; protuberances of thoracic sterna brown. Abdominal segments II–VI and X yellow-brown, VII–IX brown. Cerci pale brown.

***Hypodermal coloration*** (Fig. 77a, c). Abdominal terga with narrow, dark brown to blackish, transverse band along posterior margin.

***Head*** (Fig. 77b). Dorsally with irregular row of long, fine, simple setae along midline. ***Antenna*** (Fig. 80h). Length ~ 1.5× head length. As typical for genus. ***Developing turbinate eyes in last instar male larva*** (Fig. 80h) large, subquadrangular, broadly touching each other. ***Labrum*** (Fig. 78a, b). Length 0.5× maximum width, laterally convex. Dorsal, sub-marginal arc with ~ 32 feathered setae. ***Right mandible*** (Fig. 78d, e). Margin between prostheca and mola with few denticles. Otherwise, as typical for subgenus. ***Left mandible*** (Fig. 78f, g). Margin between prostheca and mola smooth, without denticles. Otherwise, as typical for subgenus. ***Hypopharynx*** (Fig. 78c). As typical for genus. ***Maxilla*** (Fig. 79c, d). Maxillary palp subequal in length to galea-lacinia; palp segment II approximately as long as segment I. Otherwise, as typical for genus. ***Labium*** (Fig. 79a, b). As typical for the genus. Paraglossa dorsally with two spine-like setae near inner, distolateral margin. Labial palp with segment I 0.9× length of segments II and III combined. Segment II with minute distomedial protuberance, dorsally with row of five spine-like setae near outer, distolateral margin. Segment III conical, pointed; 0.7× length of segment II; inner dorsal margin with few feathered setae.

***Thorax*. *Sterna*.** With small protuberances on sides of prosternum and close to openings of mesothoracic and metathoracic sternal apodemes (as in Fig. 108a). ***Terga*** (Figs 77b, 81a) with irregular row of long, fine, simple setae along midline. Metanotum with medioposterior, broad, paired humps. ***Legs*** (Fig. 88a–g). Ratio of leg segments: fore leg 0.8:1.0:0.3:0.1, middle leg 0.9:1.0:0.3:0.1 and hind leg 1.0:1.0:0.3:0.2. ***Femur***. Length ~ 4× maximum width. ***Claw*** with one row of seven or eight denticles and one posterior seta.

***Abdomen*. *Terga*** (Figs 77b, 81a, b, 82d, e) with irregular row of long, fine, simple setae along midline. Terga I–V with medioposterior, broad, paired humps. Posterior margin of terga: I–II smooth, without denticles; III–IX with triangular, apically rounded denticles, partly with minute, pointed denticles in between. Surface with scattered small, ovoid, striated scales. ***Tergalii*** (Fig. 81e, f). Ovoid, tracheation well developed; margins smooth, with short, fine, simple setae. ***Paraproct*** (Fig. 82a–c). Posterior margin with prolongation and row of minute denticles. ***Caudalii*** (Fig. 81c, d). Cerci apart from basal and distal part with 1–5 swimming setae per segment, initially increasing and then again decreasing toward distal part. Paracercus with nine segments.

***Pose of subimaginal gonostyli under larval cuticle*** (Fig. 80i). In mature larva ready to molt to subimago, subimaginal gonostyli packed under larval cuticle in “*Labiobaetis*-type” pose: 2^nd^ segments directed medially and bent proximally; 3^rd^ segment directed medially (as continuation of 2^nd^ segment) and narrowed apically.

**Subimago.** Unknown.

**Imago.** Unknown.

**Egg.** Unknown.

#### Distribution.

New Guinea (Fig. 147).

### ﻿﻿ Papuanatula (Papuanatula) paracopis sp. nov.

Taxon classificationAnimaliaEphemeropteraBaetidae

5D94675E-DBEE-563F-91DF-3A37F5829D8D

https://zoobank.org/BD19E649-08DB-467A-9FB5-006FFBB3E351

**Figure 83.**
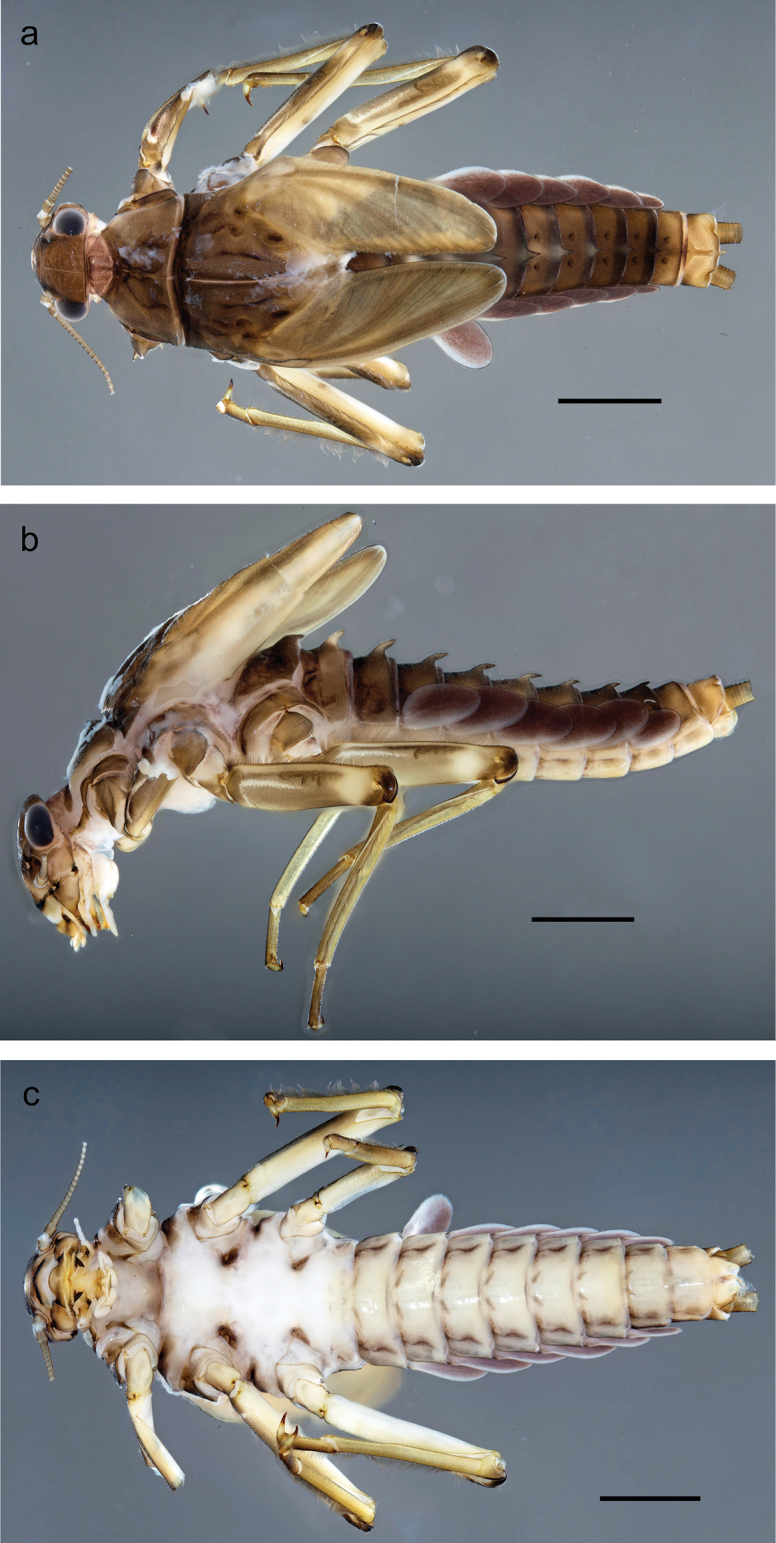
Papuanatula (Papuanatula) paracopis sp. nov., larva, habitus: **a** dorsal view **b** lateral view **c** ventral view. Scale bars: 1 mm.

**Figure 84.**
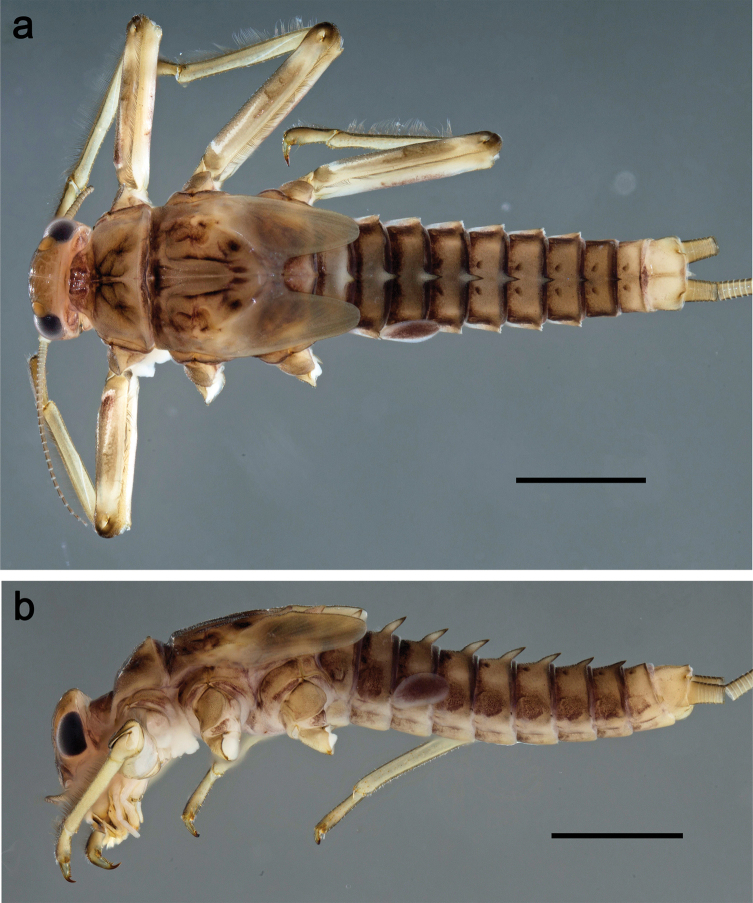
Papuanatula (Papuanatula) paracopis sp. nov., immature larva, habitus: **a** dorsal view **b** lateral view. Scale bars: 1 mm.

**Figure 85.**
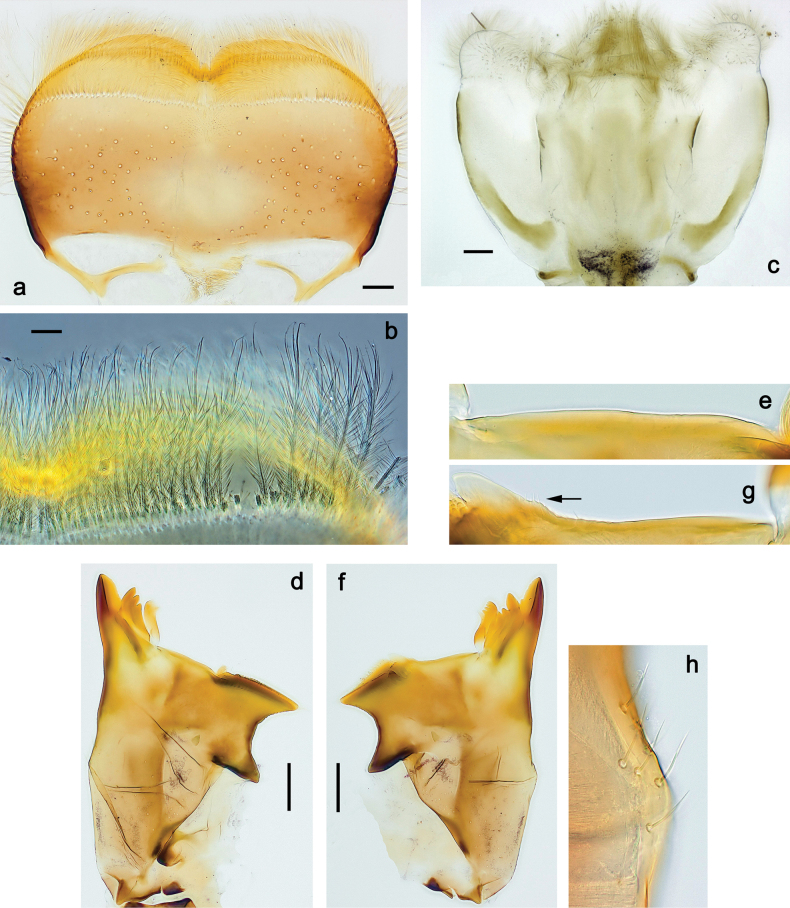
Papuanatula (Papuanatula) paracopis sp. nov., larva: **a** labrum **b** labrum: dorsal, submarginal setae **c** hypopharynx and superlinguae **d** right mandible **e** right mandible: margin between prostheca and mola **f** left mandible **g** left mandible: margin between prostheca and mola **h** left mandible: mediolateral setae. Scale bars: 20 µm (**a, c**); 10 µm (**b**); 50 µm (**d, f**).

**Figure 86.**
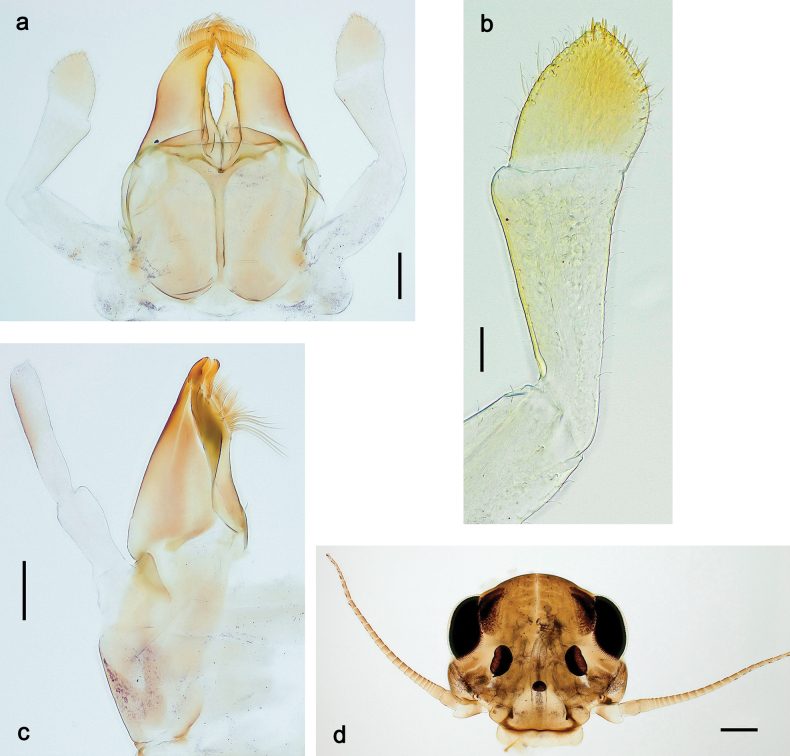
Papuanatula (Papuanatula) paracopis sp. nov., larva: **a** labium **b** labial palp **c** maxilla **d** male head, mature. Scale bars: 50 µm (**a, c**); 20 µm (**b**); 100 µm (**d**).

**Figure 87.**
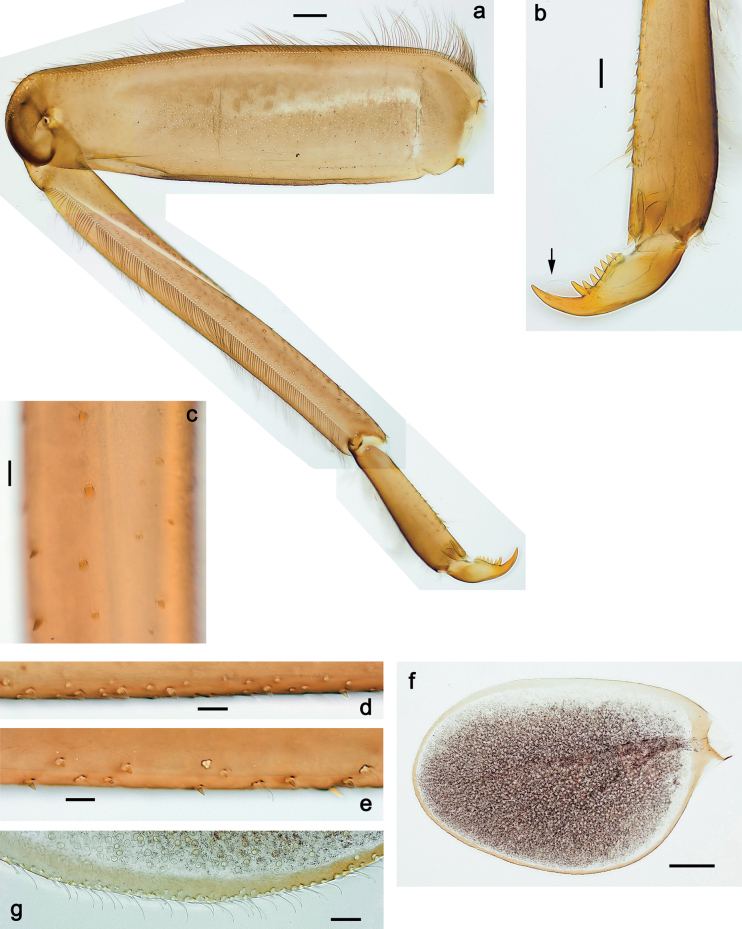
Papuanatula (Papuanatula) paracopis sp. nov., larva: **a** middle leg **b** middle tarsus and claw **c** middle tibia, posterior surface **d** middle femur, ventral margin **e** middle tibia, ventral margin **f** tergalius II **g** tergalius II, margin. Scale bars: 50 µm (**a**); 10 µm (**b–f, h**); 20 µm (**g**).

**Figure 88.**
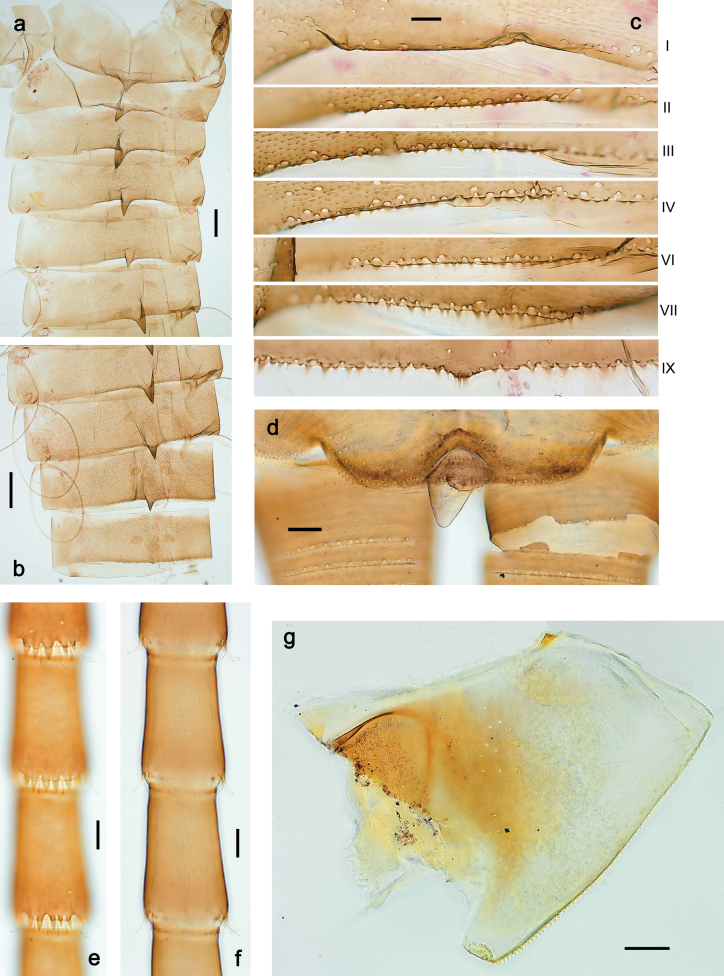
Papuanatula (Papuanatula) paracopis sp. nov., larva: **a** metanotum and abdominal terga I–V **b** abdominal terga VI–IX **c** abdominal terga **d** paracercus **e, f** cercus **g** paraproct. Scale bars: 100 µm (**a, b**); 10 µm (**c, e, f**); 20 µm (**d, g**).

**Figure 89.**
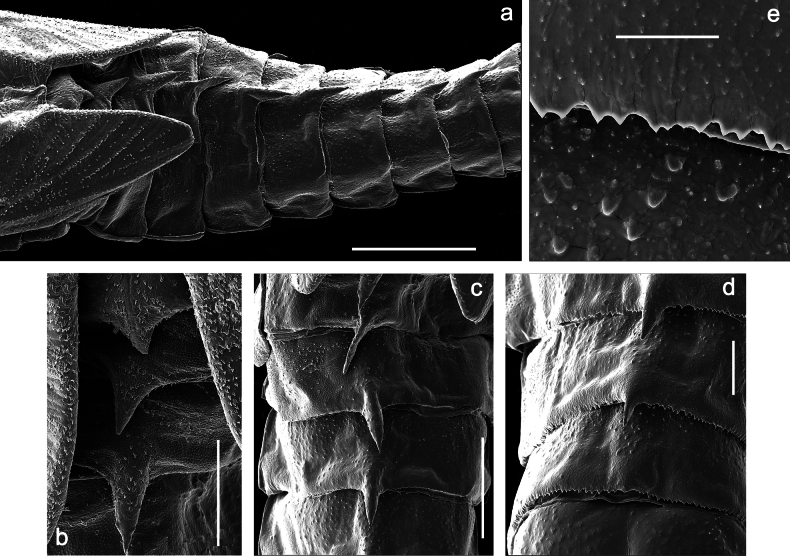
Papuanatula (Papuanatula) paracopis sp. nov., larva (SEM): **a** mesonotum, metanotum, abdominal terga I–IX **b** metanotum, abdominal terga I–II **c** abdominal terga III–V **d** abdominal terga VII–IX **e** abdominal tergum VI. Scale bars: 500 µm (**a**); 200 µm (**b**); 300 µm (**c**); 100 µm (**d**); 30 µm (**e**).

Figs 83 , 84 , 85 , 86 , 87 , 88 , 89

#### Etymology.

The species name *paracopis* refers to the morphological similarity with *P.copis*.

#### Material examined.

***Holotype*.** Papua New Guinea • larva; Eastern Highlands Prov., Akameku - Brahmin, Bismarck Range; 05°56'48"S, 145°22'14"E; 2200 m; 23.xi.2006; leg. M. Balke & Kinibel; (PNG 106); on slide; GBIFCH00592627; ZSM/SNSB. ***Paratypes*.** 4 larvae; same data as holotype; 3 on slides; GBIFCH00592583, GBIFCH00592584, GBIFCH00976109, GBIFCH00976140; MZL; 1 in alcohol; GBIFCH00975775; MZL.

#### Diagnosis.

**Larva**. The following combination of characters distinguishes *P.paracopis* sp. nov. from other species of *Papuanatula* s. str.: body dorsally without row of long, fine, simple setae along midline; metanotum and abdominal terga I–VIII medially with conspicuous, long, hook-like protuberance, bent posteriad, on abdominal segment(s) IX (X) vestigial; labial palp segment II with small, distolateral protuberance; segment III oblong, conical; femur anteriorly with angulate blank in basal 1/2; paracercus vestigial.

#### Description.

**Larva** (Figs 83–89). Body length ~ 6.8 mm, cerci much longer than body length.

***Cuticular coloration*** (Figs 83a–c, 84a, b). Head, thorax and abdomen dorsally brown to dark brown, abdominal segments X and partly IX brighter. Thorax with complex markings. Abdominal segments I–IX anteriorly and laterally darker, segments IV–IX with two dark brown dots in anteromedial area. Head and thorax ventrally ecru, thorax with pale brown to dark brown, paired protuberances near distolateral corners of sterna; abdomen ventrally pale yellow-brown, laterally with brown markings. Legs yellow-brown to brown, femur medially with submarginal brown streak and basally with angulate blank. Caudalii yellow-brown.

***Hypodermal coloration*** (Figs 83a, 84a). Abdominal terga I–IX with wide dark brown transverse band close to anterior margin and narrower dark brown transverse band close to posterior margin.

***Head*. *Antenna*** (Fig. 86d). Length ~ 1.5× head length. As typical for subgenus. ***Developing turbinate eyes in last instar male larva*** (Fig. 86d) rather narrow, with big distance to each other. ***Labrum*** (Fig. 85a, b). Length 0.6× maximum width, laterally convex. Dorsal, sub-marginal arc with > 50 densely articulated, feathered setae. ***Right mandible*** (Fig. 85d, e). Margin between prostheca and mola smooth, without denticles. Otherwise, as typical for subgenus. ***Left mandible*** (Fig. 85f–h). Margin between prostheca and mola with minute denticles toward subtriangular process. Otherwise, as typical for subgenus. ***Hypopharynx*** (Fig. 85c). As typical for genus. ***Maxilla*** (Fig. 86c). Maxillary palp slightly longer than galea-lacinia; palp segment II slender, partly sclerotized, approx. 1.2× as long as segment I, segment I thicker than segment II. Otherwise, as typical for genus. ***Labium*** (Fig. 86a, b). As typical for genus. Paraglossa dorsally with two spine-like setae near inner, distolateral margin. Labial palp with segment I 0.8× length of segments II and III combined. Segment II with very small, rounded, distomedial protuberance, dorsally with row of four or five spine-like setae near outer, distolateral margin. Segment III oblong, pointed, 0.6× length of segment II; inner dorsal margin with few feathered setae.

***Thorax*. *Sterna*.** Protuberances poorly developed. ***Terga*** (Figs 83b, 84b, 88a, 89a). Mesonotum with small, posteromedial protuberance; metanotum posteromedially with hook-like, pointed protuberance, bent posteriorly. Immature larva with short, acute, posteromedial protuberance on pronotum. Surface of fore protoptera along developing veins and on inner margins with small, triangular, pointed scales. ***Legs*** (Fig. 87a–e). Ratio of leg segments: fore leg 0.9:1.0:0.3:0.2, middle leg 1.0:1.0:0.3:0.2 and hind leg 1.1:1.0:0.3:0.2. ***Femur*.** Length ~ 3.5× maximum width. ***Claw*** with one row of nine denticles, 1^st^ denticle longer than other ones, and one posterior seta.

***Abdomen*. *Terga*** (Figs 83b, 84b, 88a–c, 89a–e). Abdominal terga I–VIII posteromedially with conspicuous, long, hook-like protuberance, bent posteriad, on abdominal segment(s) IX (X) vestigial; Posterior margin of terga: I smooth, without spines; II–IX with small, triangular, pointed denticles, increasing in length toward IX. Surface with scattered small, triangular, pointed scales. ***Tergalii*** (Fig. 87f, g). Broad oblique ovoid; tracheation developed, hardly visible due to brown pigmentation in large middle part; margins with minute serration and many short, fine, simple setae. ***Paraproct*** (Fig. 88g). Posterior margin with prolongation and with row of many minute denticles. ***Caudalii*** (Fig. 88d–f). Cerci without swimming setae; sometimes few rudimentary swimming setae or insertions still present. Paracercus vestigial.

***Pose of subimaginal gonostyli under larval cuticle*.** Unknown.

**Subimago.** Unknown.

**Imago.** Unknown.

**Egg.** Unknown.

#### Distribution.

New Guinea (Fig. 147).

### ﻿﻿ Papuanatula (Papuanatula) paralenos sp. nov.

Taxon classificationAnimaliaEphemeropteraBaetidae

75065161-45BE-5F8D-A017-EF837EBC172B

https://zoobank.org/9F92CBA3-0908-499D-A7FE-2B425C8689F8

**Figure 90.**
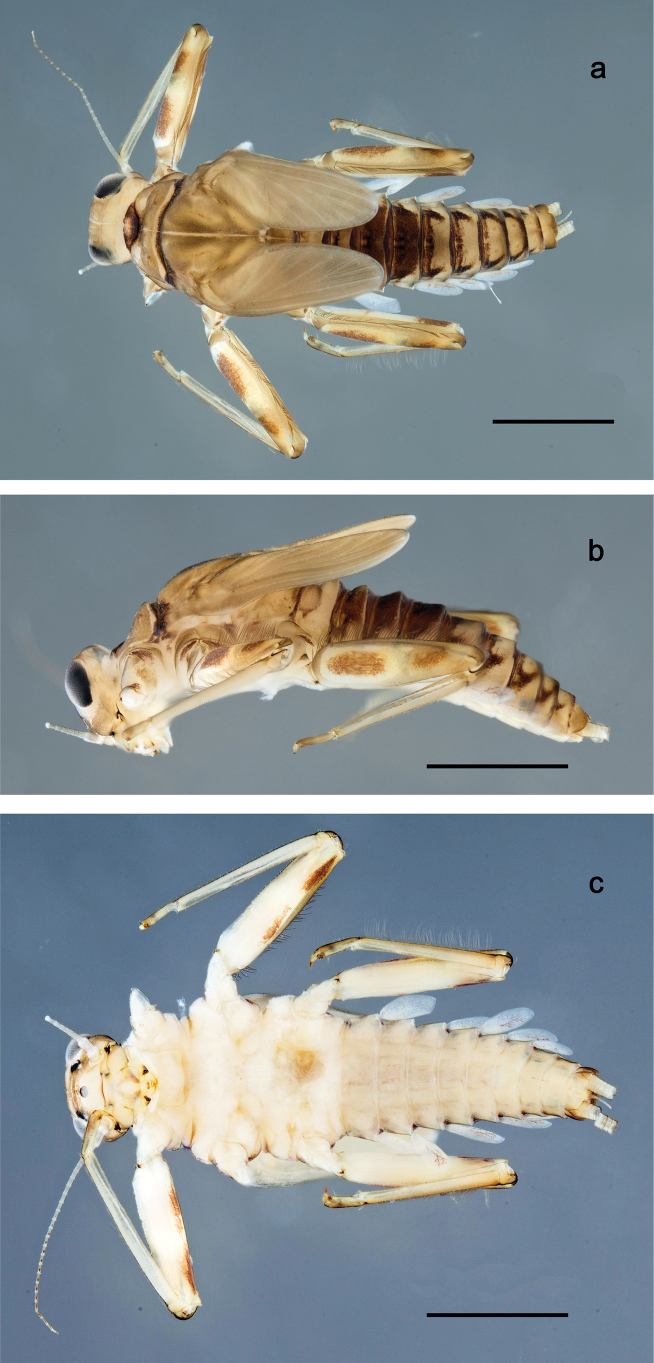
Papuanatula (Papuanatula) paralenos sp. nov., larva, habitus: **a** dorsal view **b** lateral view **c** ventral view. Scale bars: 1 mm.

**Figure 91.**
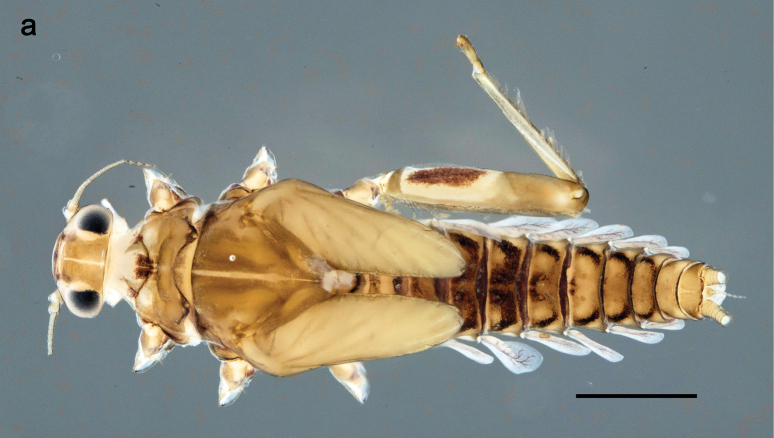
Papuanatula (Papuanatula) paralenos sp. nov., larva, habitus: **a** dorsal view. Scale bar: 1 mm.

**Figure 92.**
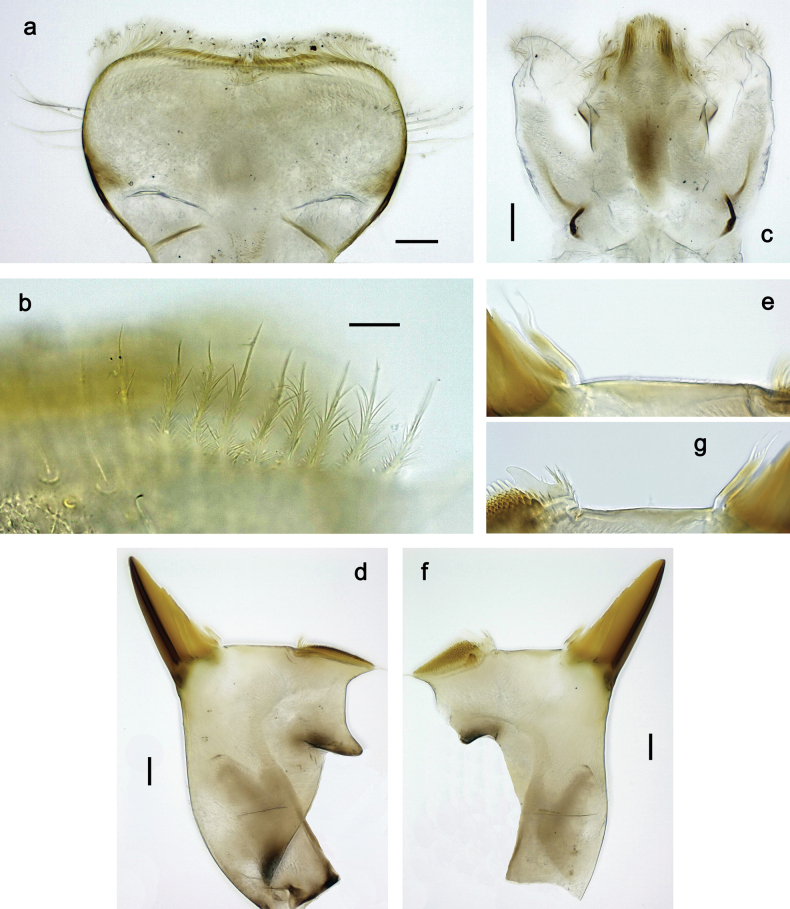
Papuanatula (Papuanatula) paralenos sp. nov., larva: **a** labrum **b** labrum: dorsal, submarginal setae **c** hypopharynx and superlinguae **d** right mandible **e** right mandible: margin between prostheca and mola **f** left mandible **g** left mandible: margin between prostheca and mola. Scale bars: 20 µm (**a, c, d, f**); 10 µm (**b**).

**Figure 93.**
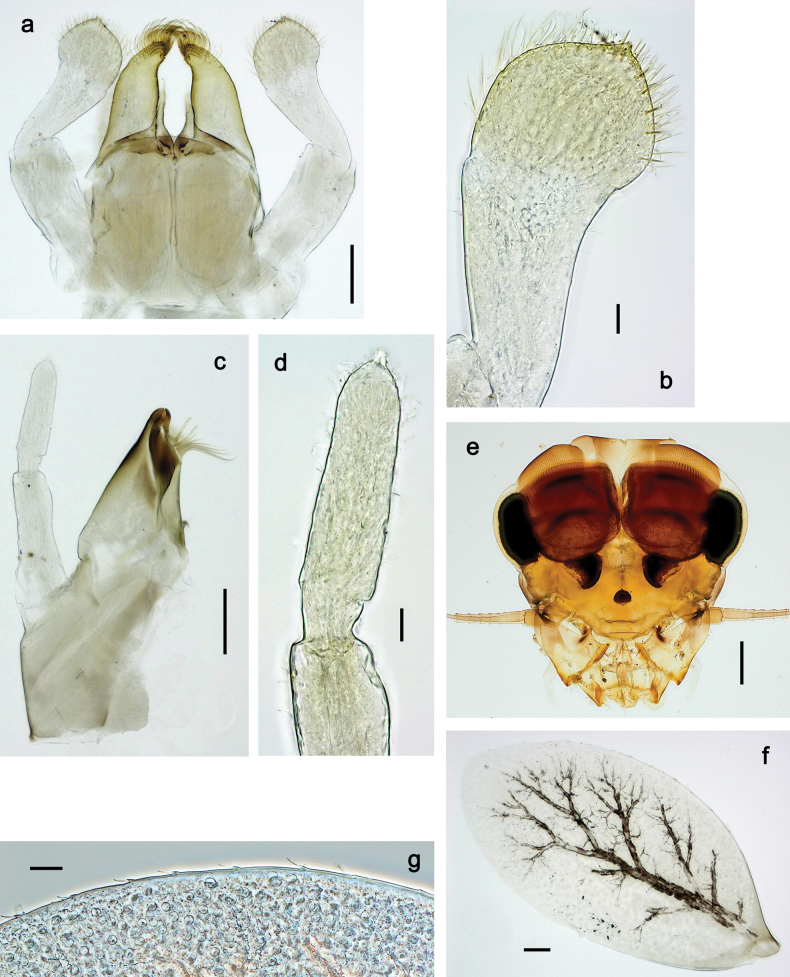
Papuanatula (Papuanatula) paralenos sp. nov., larva: **a** labium **b** labial palp **c** maxilla **d** maxillary palp **e** male head, mature **f** tergalius IV **g** tergalius IV, margin. Scale bars: 50 µm (**a, c**); 10 µm (**b, d, g**); 100 µm (**e**); 20 µm (**f**).

**Figure 94.**
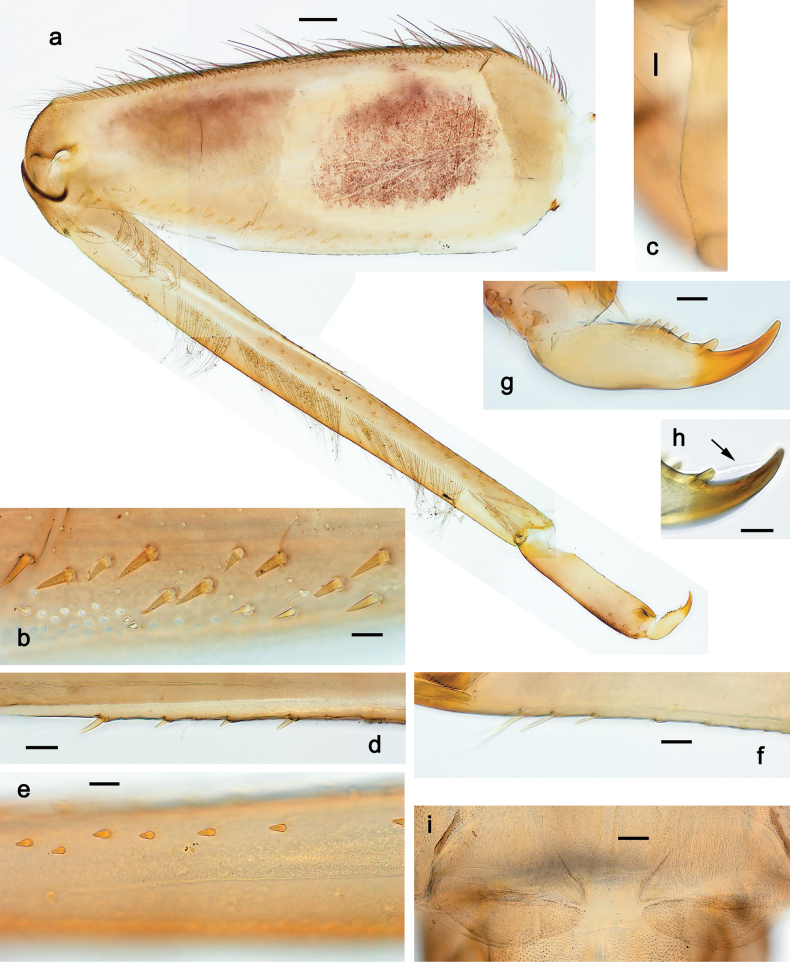
Papuanatula (Papuanatula) paralenos sp. nov., larva: **a** fore leg **b** fore femur: ventral margin **c** fore femur: posterior apex **d** fore tibia: ventral margin **e** hind tibia: posterior surface **f** fore tarsus: ventral margin **g** fore claw **h** fore claw (arrow: posterior seta) **i** developing gonostyli. Scale bars: 50 µm (**a**); 10 µm (**b–h**); 20 µm (**i**).

**Figure 95.**
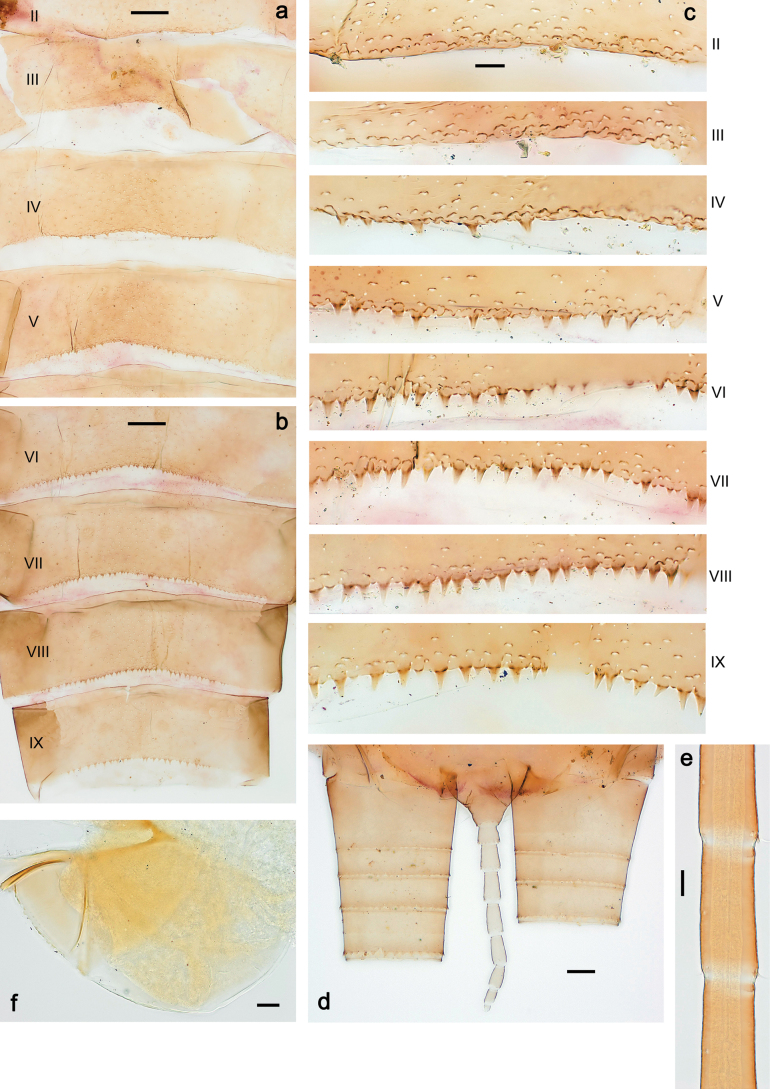
Papuanatula (Papuanatula) paralenos sp. nov., larva: **a–c** abdominal terga **d** paracercus **e** cercus **f** paraproct. Scale bars: 10 µm (**a–c, e, f**); 20 µm (**d**).

**Figure 96.**
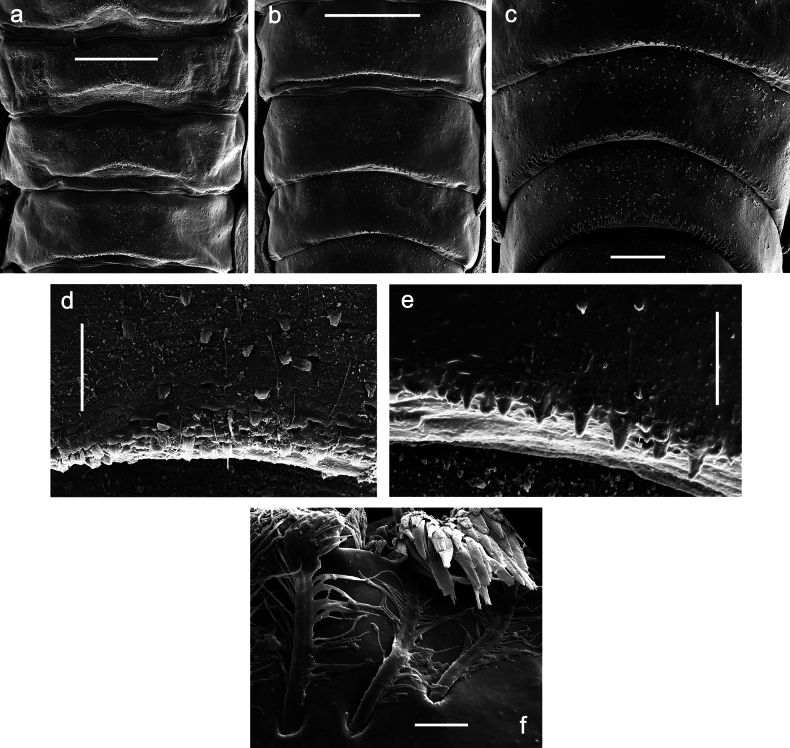
Papuanatula (Papuanatula) paralenos sp. nov., larva (SEM): **a** abdominal terga I–IV **b** abdominal terga V–VII **c** abdominal terga VII–IX **d** abdominal tergum V **e** abdominal tergum VII **f** labrum: dorsal submarginal setae. Scale bars: 300 µm (**a, b**); 100 µm (**c**); 40 µm (**d, e**); 10 µm (**f**).

Figs 90 , 91 , 92 , 93 , 94 , 95 , 96

#### Etymology.

The species name *paralenos* refers to the morphological similarity with *P.lenos*.

#### Material examined.

***Holotype*.** Papua New Guinea • larva; Eastern Highlands Prov., Marawaka, Ande; near 07°01'42"S, 145°49'48"E; 1700–1800 m, 9.xi.2006, leg. M. Balke & Kinibel; (PNG 87); on slide; GBIFCH00976054; ZSM/SNSB. ***Paratypes*.** 30 in alcohol; same data as holotype; 3 on slides; GBIFCH00976142, GBIFCH01221768, GBIFCH01221769; MZL; 27 in alcohol; GBIFCH00976019, GBIFCH00976039, GBIFCH00976064, GBIFCH00976065, GBIFCH00975796, GBIFCH00975771; MZL.

#### Other material.

Papua New Guinea • 1 larva; Gulf Prov., Marawaka, Mala; 07°05'40"S, 145°44'28"E; 1400 m; 11.xi.2006; leg. M. Balke & Kinibel; (PNG 90); in alcohol; GBIFCH00975772; MZL • 1 larva; Eastern Highlands Prov., Akameku - Brahmin, Bismarck Range; near 05°52'45"S, 145°23'13"E; 1200 m; 24.xi.2006; leg. M. Balke & Kinibel; (PNG 110); in alcohol; GBIFCH00976092; MZL • 1 larva; Central Prov., Tapini; 08°20'31"S, 146°59'49"E; 870 m; 29.x.2007; leg. Kinibel; (PNG 161); in alcohol; GBIFCH00976093; MZL. • 2 larvae; Morobe Prov., Menyamya, Mt Inji; near 07°14'49"S, 146°01'20"E; 1700 m; 14.xi.2006; leg. M. Balke & Kinibel; (PNG 96); in alcohol; GBIFCH00976067; MZL • 1 larva; Enga Prov., Wapanamanda; 05°38'06"S, 143°55'20"E; 1500 m; 6.xii.2006; leg. M. Balke & Kinibel; (PNG 128); in alcohol; GBIFCH00976066; MZL • 32 larvae; Simbu Prov., Mt. Wilhelm, Pindaunde Creek, near fish farm, S4 (oria 5); 05°49'02"S, 145°05'16"E; 2600 m; 18.viii.1999; leg. L. Čížek; 31 in alcohol; GBIFCH00976125, GBIFCH00976098, GBIFCH00976099; 1 on slide; GBIFCH00592639, GBIFCH00592640; MZL.

#### Diagnosis.

**Larva**. The following combination of characters distinguishes *P.lenos* from other species of *Papuanatula* s. str.: body dorsally without row of long, fine, simple setae along midline; body dorsally without protuberances; thorax ventrally without protuberances; pronotum dorsally with large, medial, dark brown marking at anterior margin, and fine, dark brown band along posterior margin; mesothorax with dark brown band along anterior margin; femur anteriorly with large, oblong, red brown to dark brown marking in basal 1/2, and pale red brown to dark brown marking in mediodistal area; paracercus with 6–8 segments; paraproct without posterior prolongation.

#### Description.

**Larva** (Figs 90–96). Body length 4.0–5.5 mm, cerci much longer than body length.

***Cuticular coloration*** (Figs 90a–c, 91a). Head and thorax dorsally yellow-brown to grey-brown; Abdomen dorsally yellow-brown to dark brown; segments II–IV entirely dark brown, or segment IV with dark brown, crown-like marking; segments I–IX with oblique, dark brown to blackish lateral markings. Head and thorax ventrally ecru, abdomen ventrally pale yellow-brown. Legs yellow-brown to brown; Caudalii yellow-brown.

***Hypodermal coloration*** (Figs 90a–c, 91a). Pronotum with large, dark brown marking medially at anterior margin and fine, dark brown band along posterior margin; mesothorax with narrow, dark brown band along anterior margin. Abdominal terga I–IX with wide dark brown transverse band close to anterior margin and narrower dark brown transverse band close to posterior margin. Femur anteriorly with large, oblong, red brown to dark brown marking in basal 1/2 (somewhat variable in shape), and pale red brown to dark brown marking in mediodistal area; posteriorly with two red-brown to dark brown streaks close to dorsal margin.

***Head*. *Antenna*** (Fig. 90a, c). Length ~ 2× head length. As typical for subgenus. ***Developing turbinate eyes in last instar male larva*** (Fig. 93e) large, subquadrangular, nearly touching each other in the middle. ***Labrum*** (Figs 92a, b, 96f). Length 0.5× maximum width, laterally convex. Dorsal, sub-marginal arc with 15–17 feathered setae. ***Right mandible*** (Fig. 92d, e). Margin between prostheca and mola smooth. Otherwise, as typical for subgenus. ***Left mandible*** (Fig. 92f, g). Margin between prostheca and mola smooth, with several spines close to subtriangular process. Otherwise, as typical for subgenus. ***Hypopharynx*** (Fig. 92c). As typical for genus. ***Maxilla*** (Fig. 93c, d). Maxillary palp slightly longer than galea-lacinia; palp segment II slightly shorter than segment I. Otherwise, as typical for genus. ***Labium*** (Fig. 93a, b). As typical for genus. Paraglossa with one spine-like seta on inner, distolateral margin. Labial palp with segment I subequal in length to segments II and III combined. Segment II without distomedial protuberance, dorsally with row of four spine-like setae near outer, distolateral margin. Segment III slightly bulbous, pointed, 0.8× length of segment II; inner dorsal margin with few feathered setae.

***Thorax*. *Sterna*** without protuberances. ***Terga*** without protuberances. ***Legs*** (Fig. 94a–h). Ratio of leg segments: fore leg 0.9:1.0:0.3:0.1, middle leg 1.0:1.0:0.3:0.1 and hind leg 1.1:1.0:0.3:0.1. ***Femur*.** Length ~ 2.6× maximum width. Many medium, pointed, spine-like setae along ventral margin. ***Claw*** with one row of 5–7 denticles, distalmost denticle with distance to other denticles; one posterior seta.

***Abdomen*. *Terga*** (Figs 95a–c, 96a–e). Abdominal terga without protuberances. Posterior margin of terga: I–III smooth, without denticles; IV–IX with triangular, pointed denticles. Surface with scattered small, trapezoid, striated, apically serrate scales. ***Tergalii*** (Fig. 93f, g). Ovoid, tracheation rather poorly developed; costal margin with minute serration and short, fine, simple setae; anal margin smooth, with short, fine, simple setae. ***Paraproct*** (Fig. 95f). Posterior margin without prolongation, smooth, without denticles. ***Caudalii*** (Fig. 95d, e). Cerci without swimming setae; sometimes one or two insertions still present. Paracercus with 6–8 segments.

***Pose of subimaginal gonostyli under larval cuticle*** (Fig. 94i). As typical for subgenus. Segment III conical.

**Subimago.** Unknown.

**Imago.** Unknown.

**Egg.** Unknown.

#### Distribution.

New Guinea (Fig. 147).

### ﻿﻿ Papuanatula (Papuanatula) paratuber sp. nov.

Taxon classificationAnimaliaEphemeropteraBaetidae

69934095-D95F-52A1-81A8-F295840862F3

https://zoobank.org/AB376816-DEFE-4FB4-91AF-091B16381C7B

**Figure 97.**
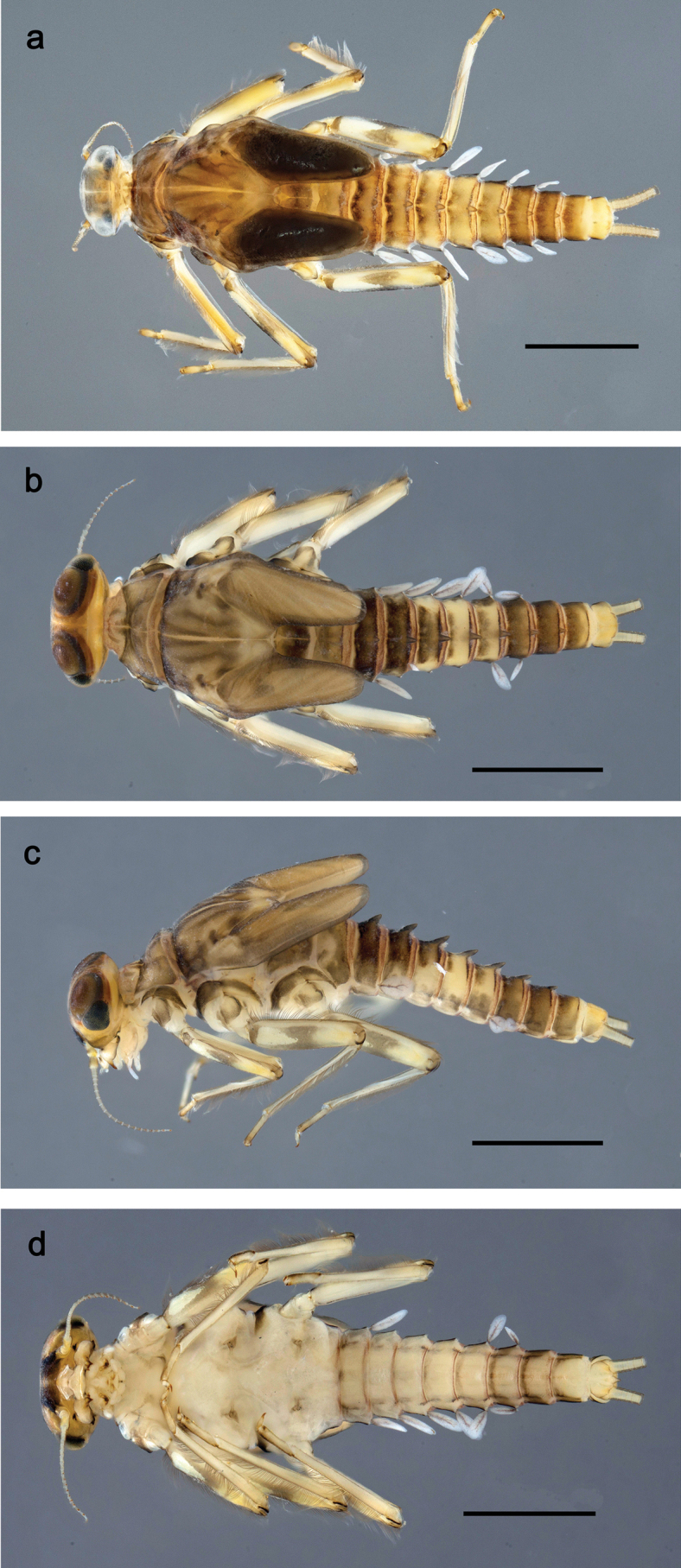
Papuanatula (Papuanatula) paratuber sp. nov., larva, habitus: **a** dorsal view, female **b** dorsal view, male **c** lateral view, male **d** ventral view, male. Scale bars: 1 mm.

**Figure 98.**
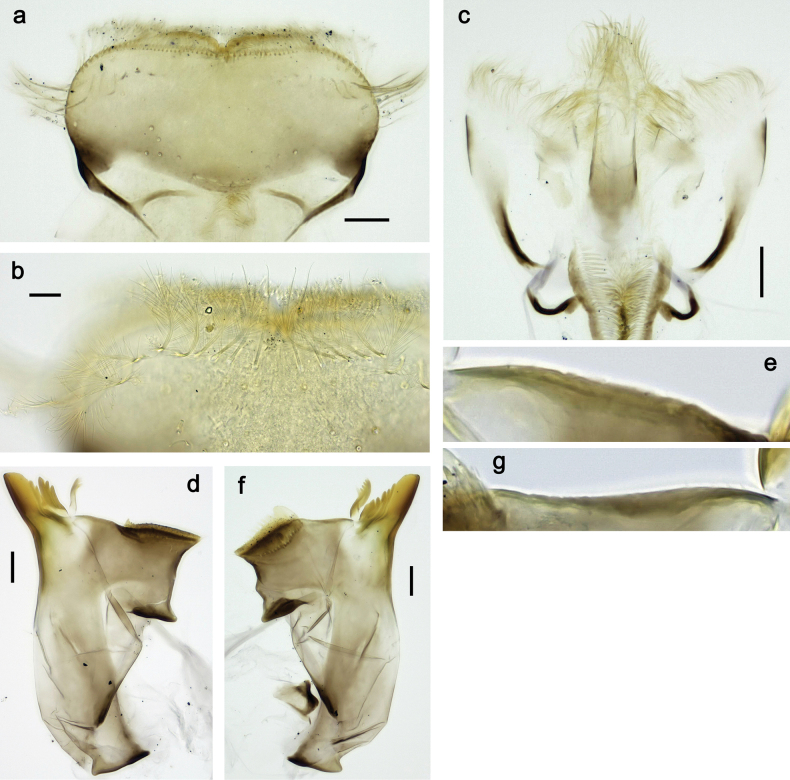
Papuanatula (Papuanatula) paratuber sp. nov., larva: **a** labrum **b** labrum: dorsal, submarginal setae **c** hypopharynx and superlinguae **d** right mandible **e** right mandible: margin between prostheca and mola **f** left mandible **g** left mandible: margin between prostheca and mola. Scale bars: 20 µm (**a, c, d, f**); 10 µm (**b**).

**Figure 99.**
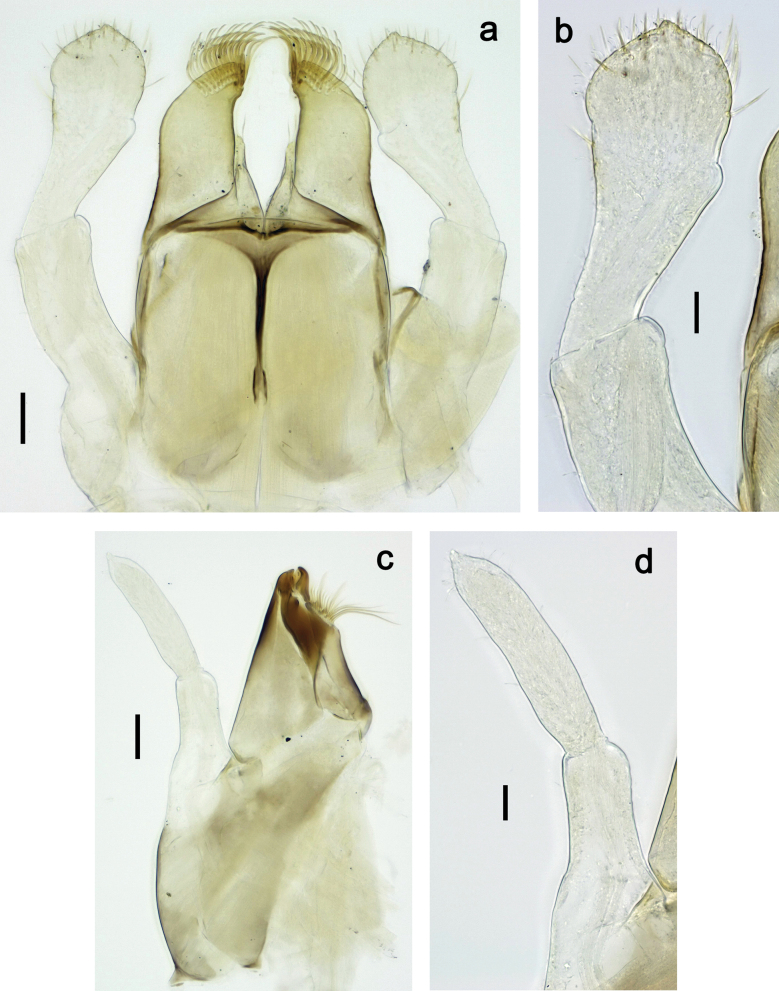
Papuanatula (Papuanatula) paratuber sp. nov., larva: **a** labium **b** labial palp **c** maxilla **d** maxillary palp. Scale bars: 20 µm (**a, c**); 10 µm (**b, d**).

**Figure 100.**
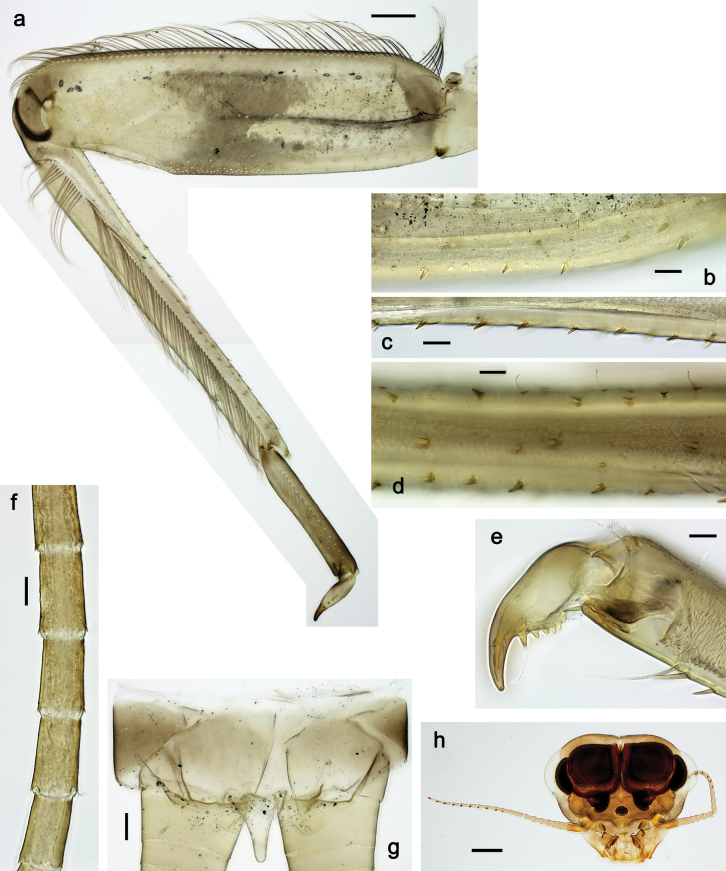
Papuanatula (Papuanatula) paratuber sp. nov., larva: **a** hind leg **b** hind femur, ventral margin **c** hind tibia: ventral margin **d** hind tibia: posterior surface **e** middle claw **f** cercus **g** paracercus **h** male head, mature. Scale bars: 50 µm (**a**); 10 µm (**b–f**); 20 µm (**g**); 100 µm (**h**).

**Figure 101.**
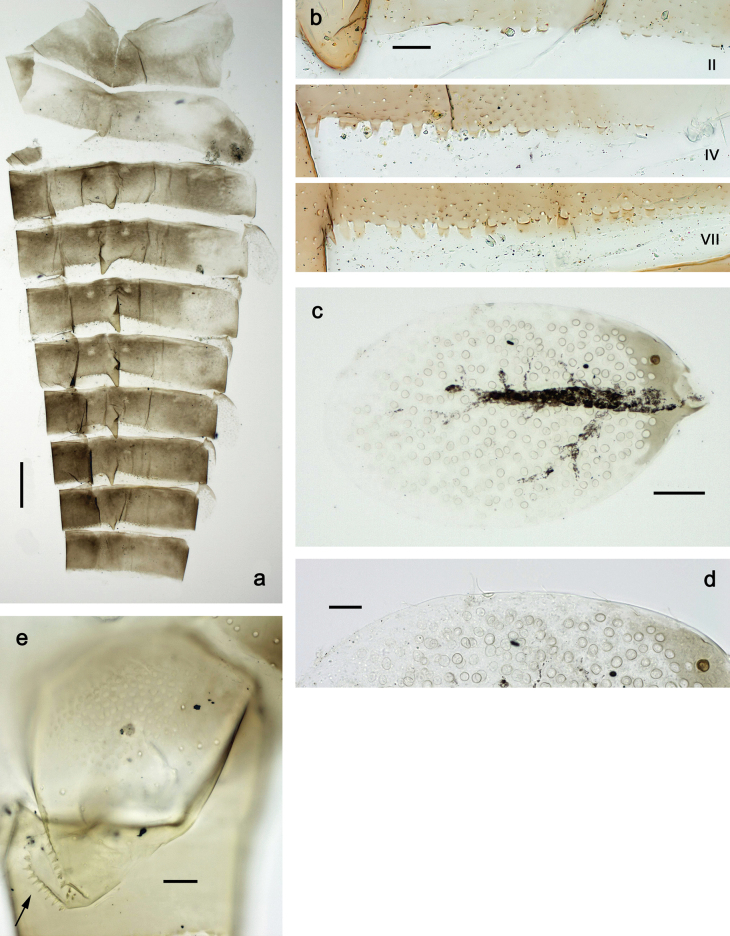
Papuanatula (Papuanatula) paratuber sp. nov., larva: **a** metanotum, abdominal terga I–IX **b** abdominal terga **c** tergalius IV **d** tergalius IV, margin **e** paraproct. Scale bars: 100 µm (**a**); 10 µm (**b, d, e**); 20 µm (**c**).

**Figure 102.**
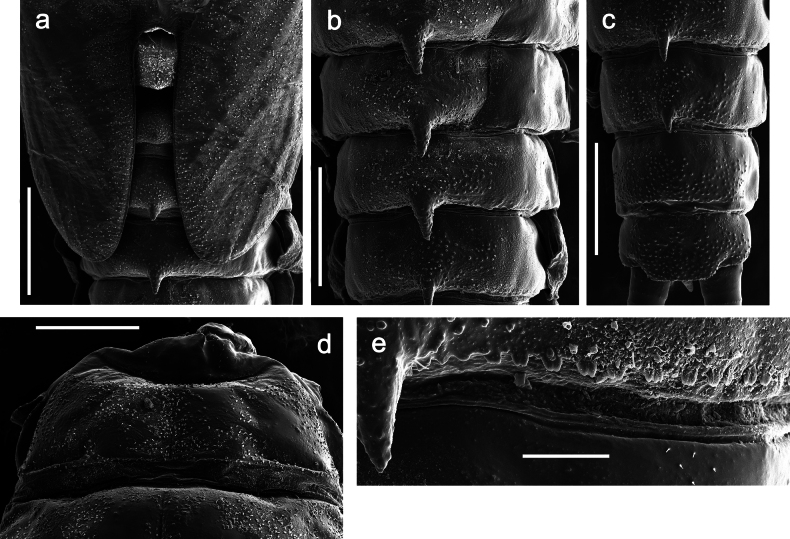
Papuanatula (Papuanatula) paratuber sp. nov., larva (SEM): **a** metanotum, abdominal terga I–III **b** abdominal terga IV–VII **c** abdominal terga VII–X **d** pronotum **e** abdominal tergum VII. Scale bars: 400 µm (**a**); 300 µm (**b–d**); 50 µm (**e**).

Figs 97 , 98 , 99 , 100 , 101 , 102

#### Etymology.

The species name *paratuber* refers to the morphological similarity with *P.tuber*.

#### Material examined.

***Holotype*.** Indonesia • larva; Papua Prov.; Riv. Je, Loc. Arfak, E of Amber village; 01°06'35"S, 133°56'51"E; 1200 m; 16.vi.2016; leg. Sumoked and M. Balke; (BH68); on slide; GBIFCH01221783; MZB. ***Paratypes*.** 38 larvae; same data as holotype; 3 on slides; GBIFCH00976113, GBIFCH01221784; GBIFCH01221803; 35 in alcohol; GBIFCH00976053, GBIFCH00976112, GBIFCH00976114, GBIFCH00976062; GBIFCH00975823, GBIFCH00975824, GBIFCH00975831; MZL.

#### Diagnosis.

**Larva**. The following combination of characters distinguishes *P.paratuber* sp. nov. from other species of *Papuanatula* s. str.: body dorsally without row of long, fine, simple setae along midline; abdominal terga I–VIII (IX) with medium, pointed, dorsoposteriorly oriented, medial protuberances; pronotum without protuberances; femur with medial, grey marking; paracercus vestigial; body size 4.3–5.2 mm.

#### Description.

**Larva** (Figs 97–102). Body length 4.3–5.2 mm, cerci much longer than body length (~ 1.3×).

***Cuticular coloration*** (Fig. 97a–d). Head, thorax and abdomen dorsally brown or grey-brown; thorax with complex pattern; abdominal segments II–IV and VII–IX darker, V–VI and X brighter. Legs grey to yellow-brown; femur medially with grey marking, yellow-brown in distal area, blank area in basal part. Head and thorax ventrally pale grey, protuberances on thoracic sterna darker; abdomen yellow-brown to grey, sterna VII and VIII darker.

***Hypodermal coloration*** (Fig. 97a, b). Abdominal segments I–IX dorsally with dark brown, narrow, transverse band on posterior margins; intersegmental membranes slightly reddish-grey.

***Head*. *Antenna*** (Fig. 100h). Length 1.5× head length. As typical for subgenus. ***Developing turbinate eyes in last instar male larva*** (Fig. 100h) large, sub quadrangular, nearly touching each other in the middle. ***Labrum*** (Fig. 98a, b). Length 0.5× maximum width, laterally convex. Dorsal, sub-marginal arc with ~ 9 feathered setae. ***Right mandible*** (Fig. 98d, e). Margin between prostheca and mola smooth. Otherwise, as typical for subgenus. ***Left mandible*** (Fig. 98f, g). Margin between prostheca and mola smooth. Otherwise, as typical for subgenus. ***Hypopharynx*** (Fig. 98c). As typical for genus. ***Maxilla*** (Fig. 99c, d). Maxillary palp ~ 1.2× length of galea-lacinia; palp segment II ~ 1.1× length of segment I. Otherwise, as typical for genus. ***Labium*** (Fig. 99a, b). As typical for genus. Paraglossa dorsally with two spine-like setae near inner, distolateral margin. Labial palp with segment I ~ 1.2× length of segments II and III combined. Segment II with slight, broadly rounded, distomedial protuberance, dorsally with row of four spine-like setae near outer, distolateral margin. Segment III slightly pentagonal, pointed, 0.8× length of segment II.

***Thorax*. *Sterna*.** With small protuberances on sides of prosternum and close to openings of mesothoracic and metathoracic sternal apodemes (as Fig. 108a). ***Terga*** (Fig. 102a, d). Pro-, meso- and metanotum without protuberances. ***Legs*** (Fig. 100a–e). Ratio of leg segments: fore leg 0.9:1.0:0.4:0.1, middle leg 0.9:1.0:0.3:0.1 and hind leg 1.0:1.0:0.4:0.2. ***Femur*.** Length ~ 3.4× maximum width; inner margin with short, spine-like setae along margin. ***Claw*** with one row of five or six denticles and one posterior seta.

***Abdomen*. *Terga*** (Figs 101a, b, 102a–c, e). Terga I–VIII with medium, pointed, dorsoposteriorly oriented, medial protuberances. Posterior margin of terga: I smooth, without denticles; II with rudimentary denticles; III–IX with small, triangular denticles, apically sometimes split. ***Tergalii*** (Fig. 101c, d). Ovoid, tracheation mainly limited to trunk; margins smooth, with short, fine, simple setae. ***Para­proct*** (Fig. 101e). Posterior margin with prolongation and row of minute denticles. ***Caudalii*** (Fig. 100f, g). Cerci without swimming setae. Paracercus vestigial.

***Pose of subimaginal gonostyli under larval cuticle*.** Unknown.

**Subimago.** Unknown.

**Imago.** Unknown.

**Egg.** Unknown.

#### Distribution.

New Guinea (Fig. 147).

### ﻿﻿ Papuanatula (Papuanatula) parvatubera sp. nov.

Taxon classificationAnimaliaEphemeropteraBaetidae

43ABA01C-E8F5-5DD4-A468-D4334657B92B

https://zoobank.org/ECEB0462-73E6-41F2-B425-A438E04FE80C

**Figure 103.**
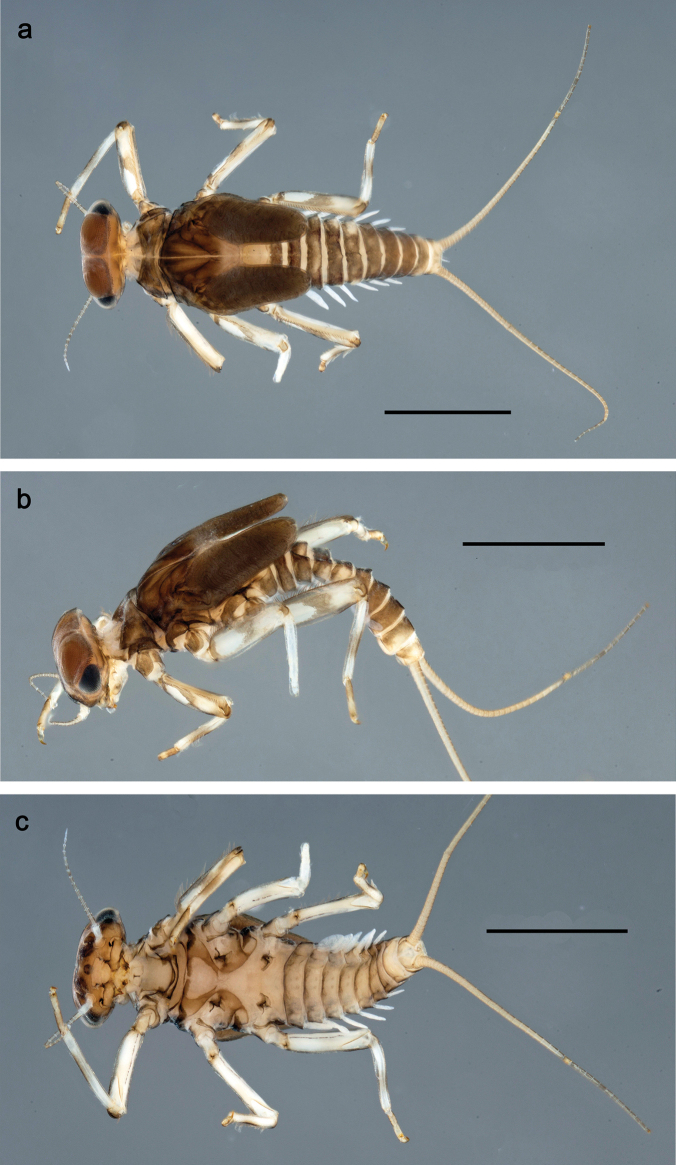
Papuanatula (Papuanatula) parvatubera sp. nov., larva, habitus: **a** dorsal view **b** lateral view **c** ventral view. Scale bars: 1 mm.

**Figure 104.**
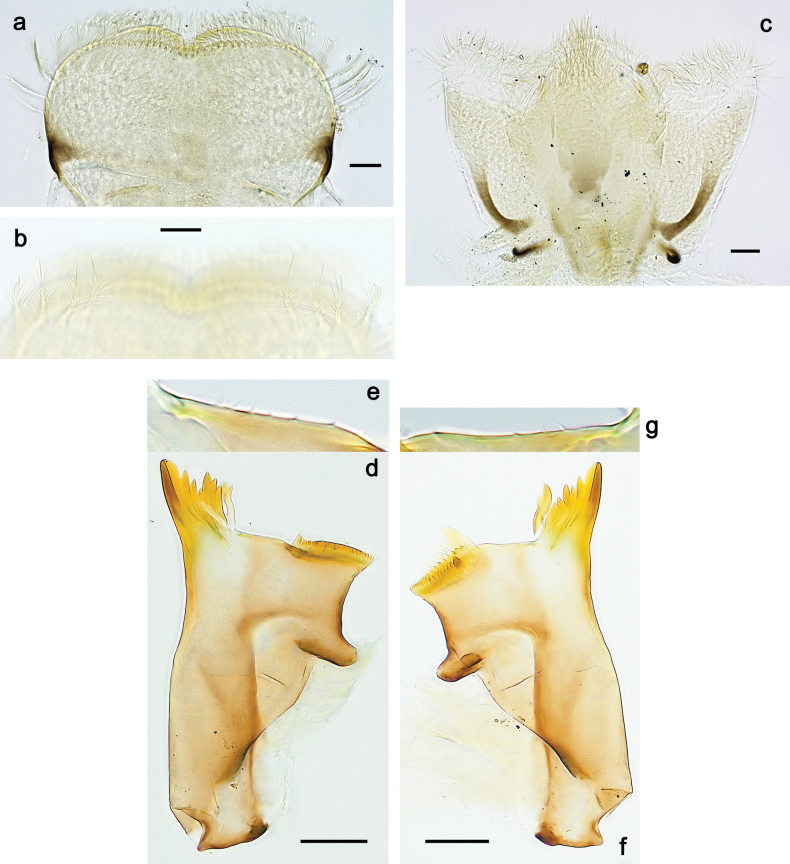
Papuanatula (Papuanatula) parvatubera sp. nov., larva: **a** labrum **b** labrum: dorsal, submarginal setae **c** hypopharynx and superlinguae **d** right mandible **e** right mandible: margin between prostheca and mola **f** left mandible **g** left mandible: margin between prostheca and mola. Scale bars: 10 µm (**a–c**); 20 µm (**d, f**).

**Figure 105.**
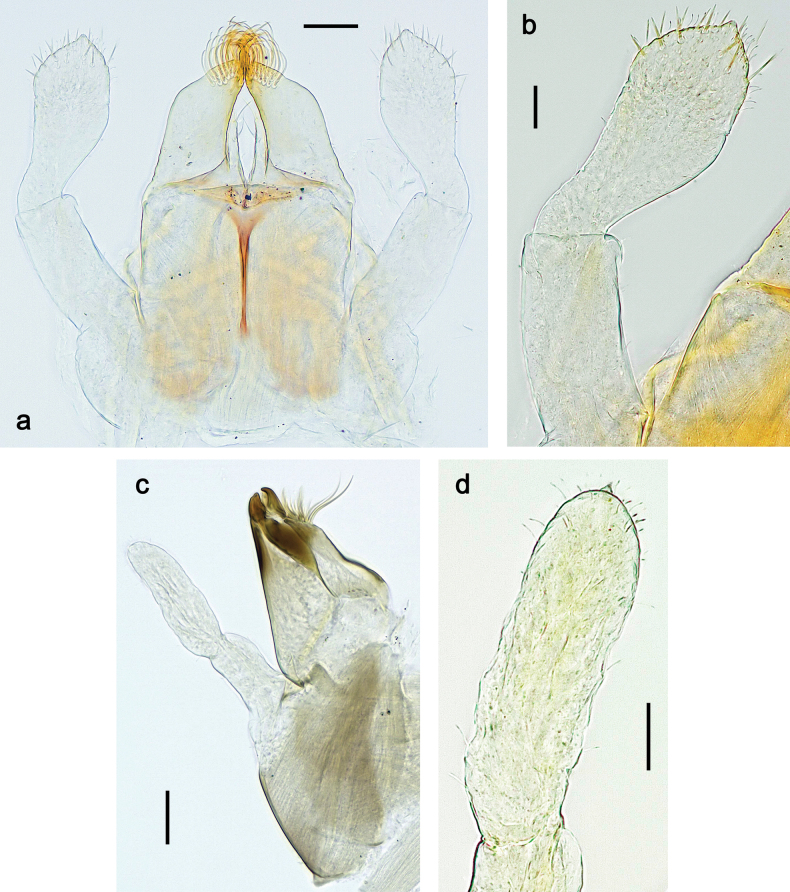
Papuanatula (Papuanatula) parvatubera sp. nov., larva: **a** labium **b** labial palp **c** maxilla **d** maxillary palp. Scale bars: 20 µm (**a, c**); 10 µm (**b, d**).

**Figure 106.**
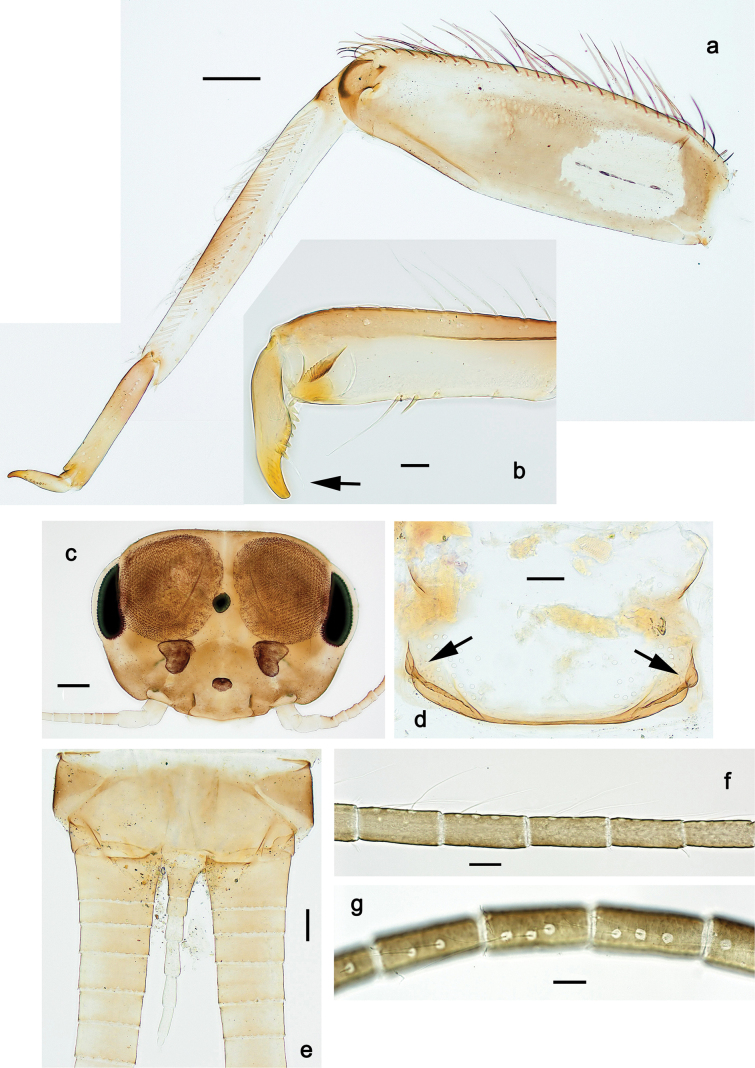
Papuanatula (Papuanatula) parvatubera sp. nov., larva: **a** hind leg **b** middle tarsus and claw (arrow: posterior seta) **c** male head, mature **d** prosternum **e** paracercus **f** cercus, dorsal view **g** cercus, lateral view. Scale bars: 50 µm (**a**); 10 µm (**b, f, g**); 100 µm (**c**); 20 µm (**d, e**).

**Figure 107.**
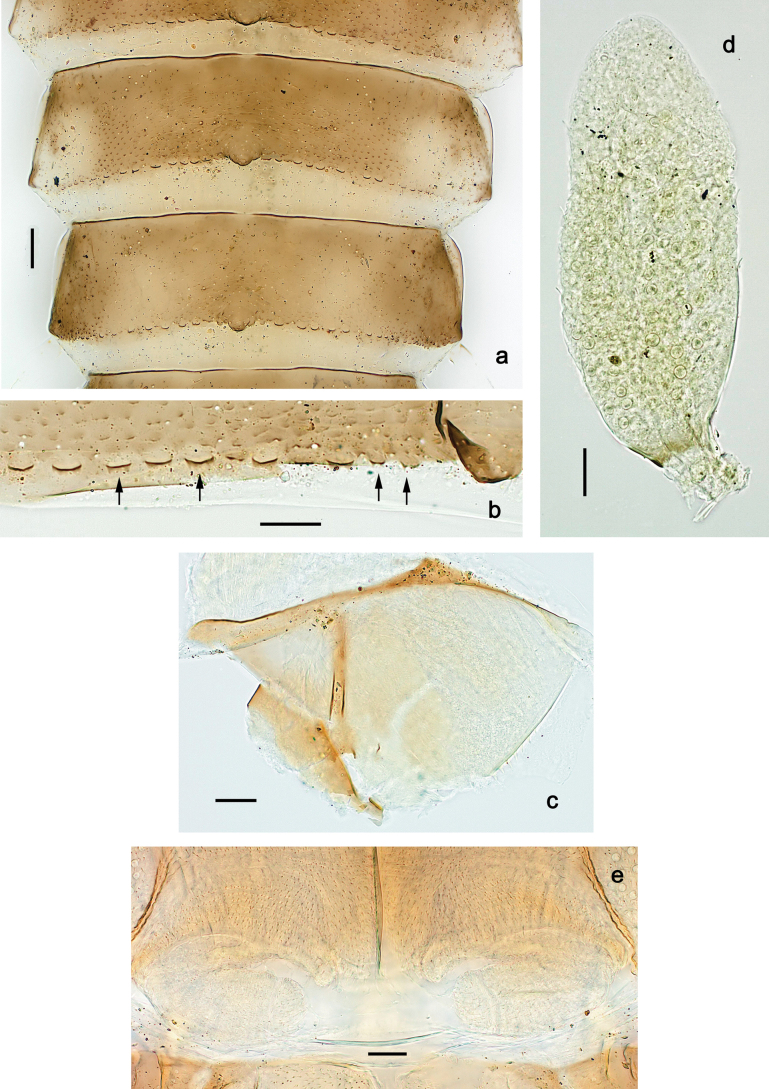
Papuanatula (Papuanatula) parvatubera sp. nov., larva: **a** abdominal terga III–V **b** abdominal tergum V (arrows: minute, acute spines) **c** paraproct **d** tergalius IV **e** developing gonostyli. Scale bars: 20 µm (**a**); 10 µm (**b–e**).

**Figure 108.**
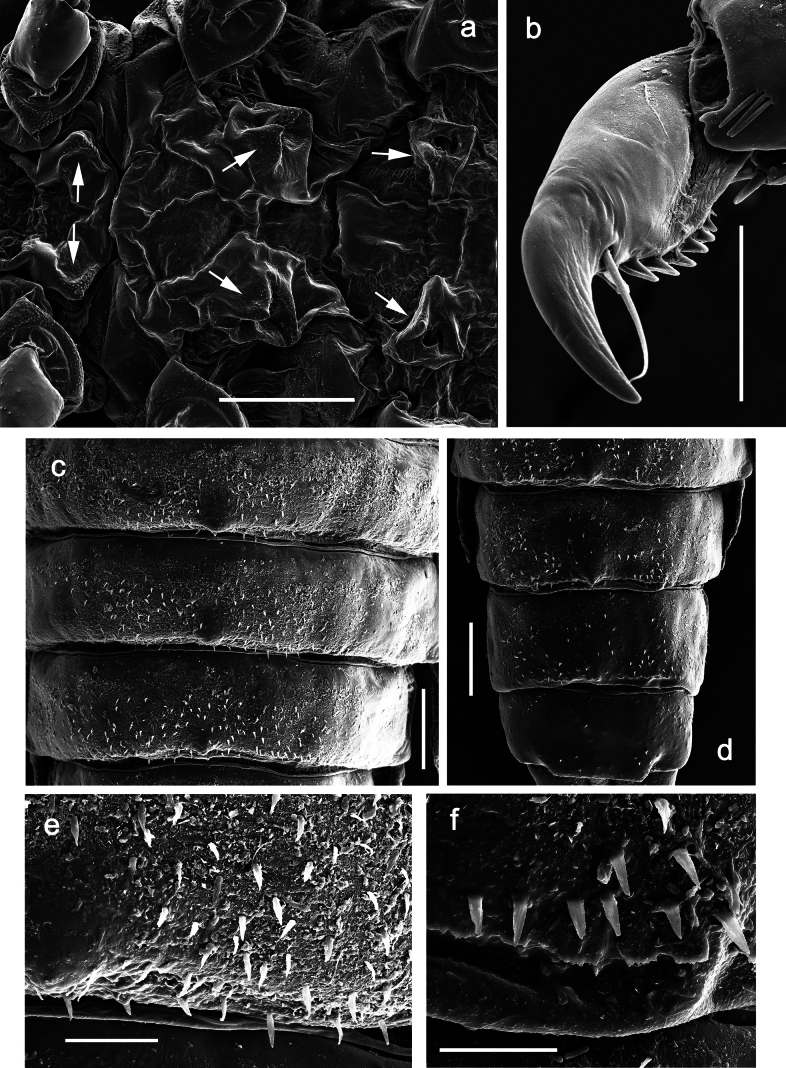
Papuanatula (Papuanatula) parvatubera sp. nov., larva (SEM): **a** pro-, meso-, and metasternum (arrows: protuberances) **b** hind claw **c** abdominal terga III–V **d** abdominal terga VII–X **e** abdominal tergum IV **f** abdominal tergum VIII. Scale bars: 200 µm (**a**); 40 µm (**b**); 100 µm (**c, d**); 30 µm (**e**); 20 µm (**f**).

Figs 103 , 104 , 105 , 106 , 107 , 108

#### Etymology.

The species name *parvatubera* is based on the Latin words *parva tubera* meaning “small humps”, referring to the specific abdominal protuberances.

#### Material examined.

***Holotype*.** Papua New Guinea • larva; Madang Prov., Highway nr Madang, ford; 05°24'24"S, 145°38'13"E; 80 m; 26.xi. / 2.–3.xii.2006; leg. Binatang Boys; (PNG 117); on slide; GBIFCH00592594; ZSM/SNSB. ***Paratypes*.** 12 larvae; same data as holotype; 5 on slides; GBIFCH00592638, GBIFCH00592595, GBIFCH00592534, GBIFCH00592535, GBIFCH00976089; MZL; 7 in alcohol; GBIFCH00975780, GBIFCH00976088, GBIFCH00976090, GBIFCH00976045; MZL.

#### Diagnosis.

**Larva**. The following combination of characters distinguishes *P.parvatubera* sp. nov. from other species of *Papuanatula* s. str.: body dorsally without row of long, fine, simple setae along midline; abdominal terga (III) IV–VIII medially with small, triangular protuberance, oriented posteriorly, terga II, III and IX sometimes with vestigial protuberances; femur medially with broad, transversal marking and large blanks in basal and distal area; paracercus with seven or eight segments.

#### Description.

**Larva** (Figs 103–108). Body length 2.8–3.1 mm, cerci somewhat shorter than body length (~ 0.8×).

***Cuticular coloration*** (Fig. 103a–c). Head, thorax and abdomen dorsally brown to grey-brown; thorax with complex pattern; abdominal segments IV and V laterally pale grey-brown, X pale grey-brown. Femur medially with grey-brown transversal marking, basally and distally with large blanks; tibia ecru with grey-brown in medial area; tarsus distally yellow-brown, basally brown. Head, thorax and abdomen ventrally pale brown to brown, protuberances of thoracic sterna dark brown, abdominal segments IX and X beige. Cerci pale grey-brown.

***Hypodermal coloration*.** None (apart from antennal flagellum, as typical for subgenus).

***Head*. *Antenna*** (Fig. 103a, c). Length 1.5× head length. As typical for subgenus. ***Developing turbinate eyes in last instar male larva*** (Fig. 106c) large, nearly touching each other, slightly trapezoid. ***Labrum*** (Fig. 104a, b). Relatively small, length 0.5× maximum width, laterally convex. Dorsal, sub-marginal arc with ~ 10 feathered setae. ***Right mandible*** (Fig. 104d, e). Margin between prostheca and mola with row of minute denticles. Otherwise, as typical for subgenus. ***Left mandible*** (Fig. 104f, g). Margin between prostheca and mola with row of minute denticles. Otherwise, as typical for subgenus. ***Hypopha­rynx*** (Fig. 104c). As typical for genus. ***Maxilla*** (Fig. 105c, d). Maxillary palp subequal in length to galea-lacinia, robust; palp segment II ~ 1.3× length of segment I. Otherwise, as typical for genus. ***Labium*** (Fig. 105a, b). As typical for genus. Paraglossa dorsally with two spine-like setae near inner, distolateral margin. Labial palp with segment I ~ 1.1× length of segments II and III combined. Segment II without distomedial protuberance, dorsally with row of four spine-like setae near outer, distolateral margin. Segment III conical, pointed, 0.7× length of segment II.

***Thorax*. *Sterna*** (Figs 106d, 108a). With small protuberances on sides of prosternum and close to openings of mesothoracic and metathoracic sternal apodemes. ***Terga*** without protuberances. ***Legs*** (Figs 106a, b, 108b). Ratio of leg segments: fore leg 0.9:1.0:0.3:0.2, middle leg 1.0:1.0:0.3:0.2 and hind leg 0.9:1.0:0.3:0.2. ***Femur*.** Length ~ 3× maximum width. ***Claw*** with one row of seven denticles, distalmost denticle with distance to other denticles; one posterior seta.

***Abdomen*. *Terga*** (Figs 107a, b, 108c–f). Terga (III) IV–VIII posteromedially with small, triangular protuberance, oriented posteriorly, terga II, III and IX sometimes with vestigial protuberance. Posterior margin of terga: I–II smooth, without spines; III–IX with short, rounded denticles, apically carrying minute, fine, acute spines. Surface with scattered small, long-triangular, pointed, striated scales. ***Tergalii*** (Fig. 107d). Narrow oblong, tracheation undeveloped; margins smooth, with few short, fine, simple setae. ***Paraproct*** (Fig. 107c). Posterior margin with prolongation and row of minute denticles. ***Caudalii*** (Fig. 106e–g). Cerci in distal 1/2 with 1–4 swimming setae per segment. Paracercus with seven or eight segments.

***Pose of subimaginal gonostyli under larval cuticle*** (Fig. 107e). As typical for the subgenus.

**Subimago.** Unknown.

**Imago.** Unknown.

**Egg.** Unknown.

#### Distribution.

New Guinea (Fig. 147).

### ﻿﻿ Papuanatula (Papuanatula) pilosa sp. nov.

Taxon classificationAnimaliaEphemeropteraBaetidae

BBCFDF52-34BD-518E-A410-75D89BEB1557

https://zoobank.org/86310879-9E47-448B-925C-0797EE2829A9

**Figure 109.**
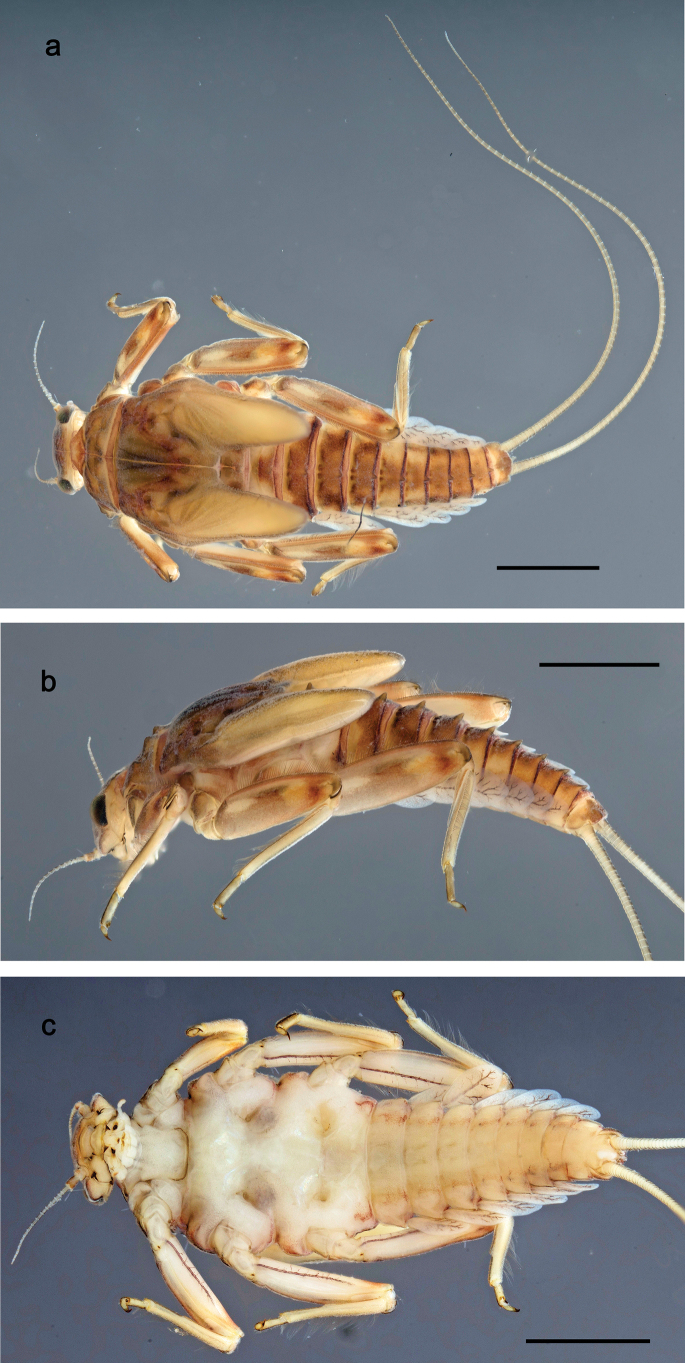
Papuanatula (Papuanatula) pilosa sp. nov., larva, habitus: **a** dorsal view **b** lateral view **c** ventral view. Scale bars: 1 mm.

**Figure 110.**
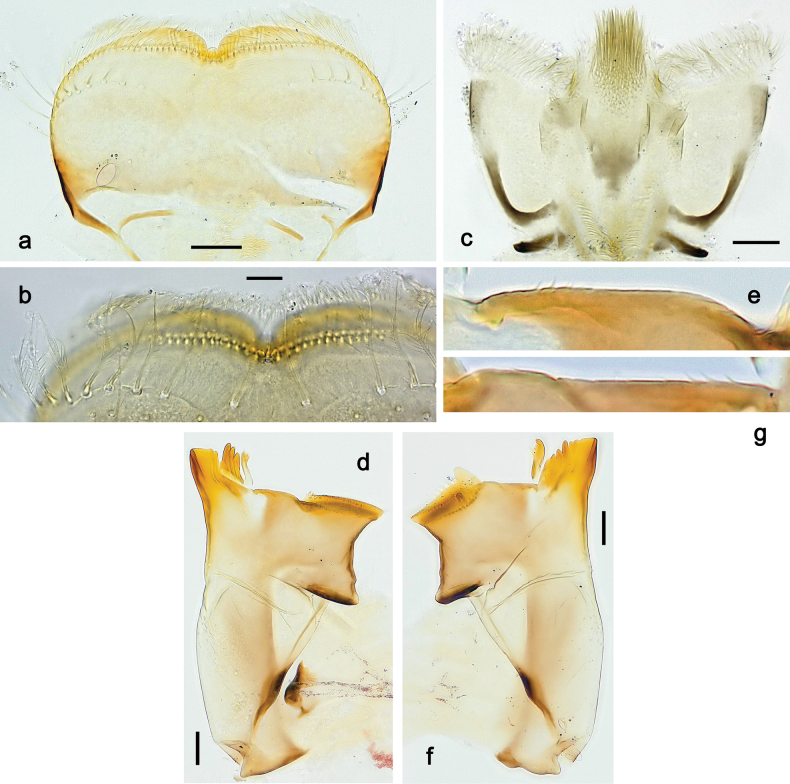
Papuanatula (Papuanatula) pilosa sp. nov., larva: **a** labrum **b** labrum: dorsal, submarginal setae **c** hypopharynx and superlinguae **d** right mandible **e** right mandible: margin between prostheca and mola **f** left mandible **g** left mandible: margin between prostheca and mola. Scale bars: 20 µm (**a, c, d, f**); 10 µm (**b**).

**Figure 111.**
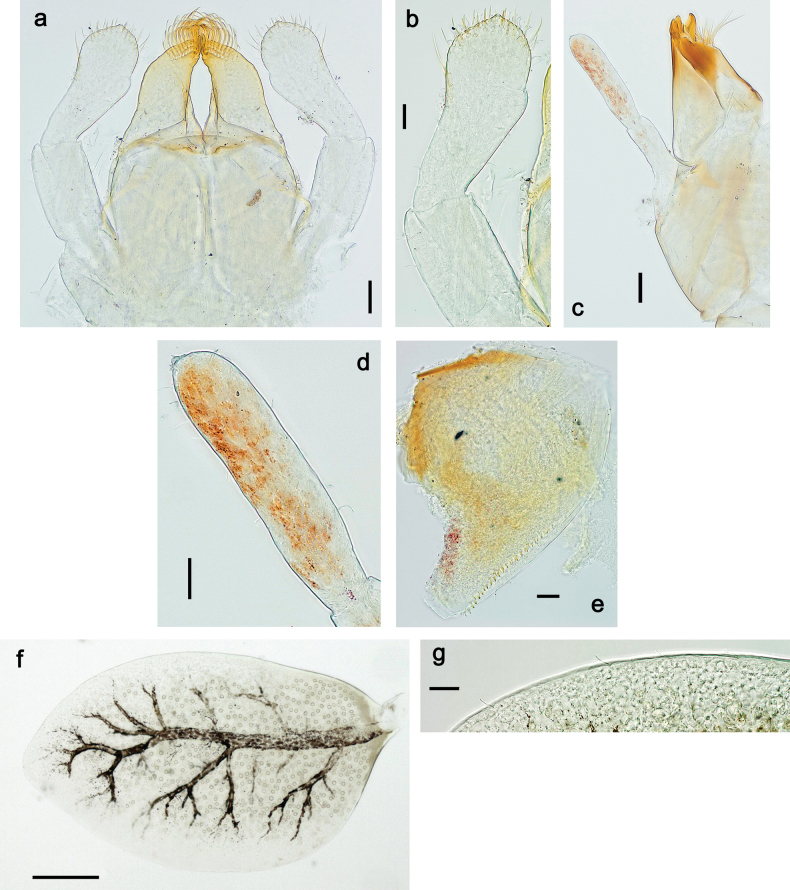
Papuanatula (Papuanatula) pilosa sp. nov., larva: **a** labium **b** labial palp **c** maxilla **d** maxillary palp segment III **e** paraproct **f** tergalius IV **g** tergalius IV: margin. Scale bars: 20 µm (**a, c**); 10 µm (**b, d, e, g**); 50 µm (**f**).

**Figure 112.**
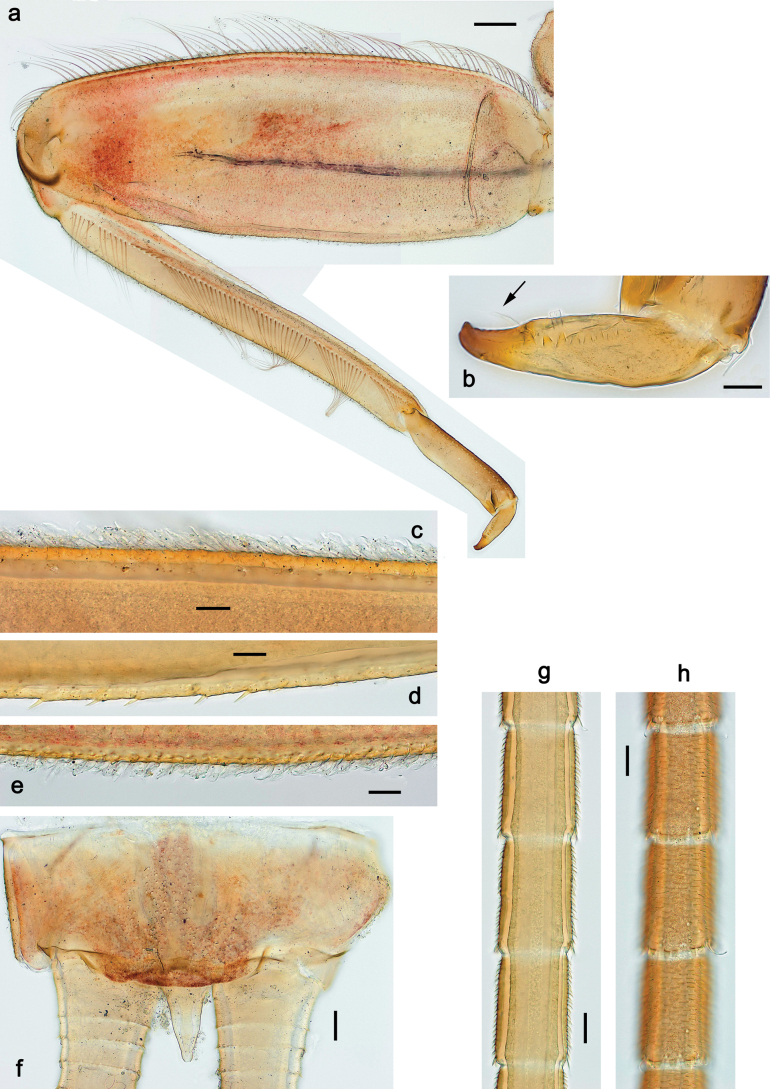
Papuanatula (Papuanatula) pilosa sp. nov., larva: **a** middle leg **b** middle claw **c** middle tibia: outer margin **d** middle tibia: inner margin **e** middle femur: inner margin **f** paracercus **g, h** cercus. Scale bars: 50 µm (**a**); 10 µm (**b–e, g, h**); 20 µm (**f**).

**Figure 113.**
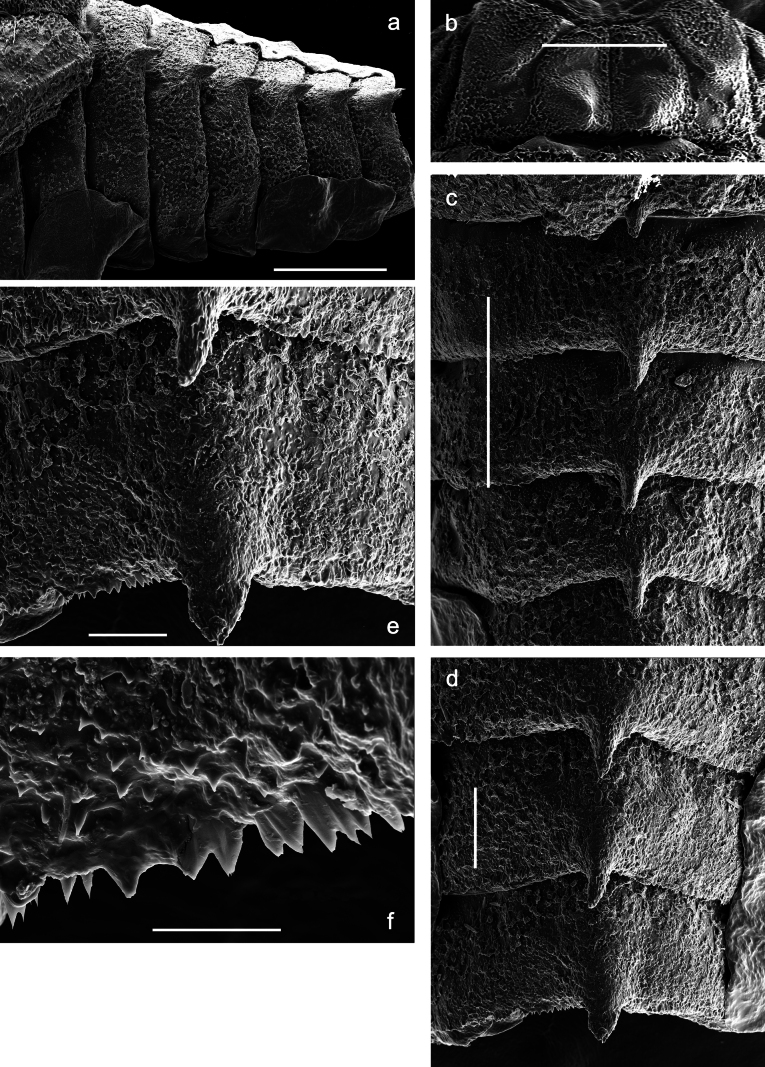
Papuanatula (Papuanatula) pilosa sp. nov., larva (SEM): **a** abdominal terga III–IX **b** pronotum **c** abdominal terga III–VI **d** abdominal terga VII–IX **e** abdominal terga VIII–IX **f** abdominal tergum IX, posterior margin. Scale bars: 400 µm (**a**); 300 µm (**b, c**); 100 µm (**d**); 50 µm (**e**); 20 µm (**f**).

Figs 109 , 110 , 111 , 112 , 113

#### Etymology.

The species name is based on the Latin word *pilosus* meaning “hairy” and refers to the rows of fine setae on inner margin of femur, outer margin of tibia, and laterally on cerci.

#### Material examined.

***Holotype*.** Indonesia • larva; Papua Prov.; Riv. Je, Loc. Arfak, E of Amber village; 01°06'35"S, 133°56'51"E; 1200 m; 16.vi.2016; leg. Sumoked and M. Balke; (BH68); on slide; GBIFCH00976044; MZB.

#### Diagnosis.

**Larva**. The following combination of characters distinguishes *P.pilosa* sp. nov. from other species of *Papuanatula* s. str.: body dorsally without row of long, fine, simple setae along midline; abdominal terga I–VIII (IX) with medium, pointed, dorsoposteriorly oriented, medial protuberances; pronotum with paired, medioposterior protuberances; femur with medial, grey marking; inner margin of femur and outer margin of tibia with irregular rows of medium, fine setae; cerci bilaterally with row of short, fine setae; paracercus vestigial; body size 4.5 mm.

#### Description.

**Larva** (Figs 109–113). Body length 4.5 mm, cerci much longer than body length (~ 1.2×).

***Cuticular coloration*** (Fig. 109a–c). Head, thorax and abdomen dorsally reddish-brown; thorax with complex pattern; abdominal segments I, V–VI and X slightly brighter. Legs reddish-brown; femur medially with darker marking, red-brown in distal area, bright area in basal part. Head and thorax ventrally ecru, protuberances on thoracic sterna darker; abdomen yellow-brown.

***Hypodermal coloration*** (Fig. 109a, b). Abdominal segments I–IX dorsally with narrow, dark brown, transverse band on posterior margins; intersegmental membranes slightly reddish-grey.

***Head*. *Antenna*** (Fig. 109a–c). Length ~ 1.5× head length. As typical for subgenus. ***Developing turbinate eyes in last instar male larva*** unknown. ***Labrum*** (Fig. 110a, b). Length 0.5× maximum width, laterally convex. Dorsal, sub-marginal arc with ~ 9 feathered setae. ***Right mandible*** (Fig. 110d, e). Margin between prostheca and mola with some minute denticles toward prostheca. Otherwise, as typical for subgenus. ***Left mandible*** (Fig. 110f, g). Margin between prostheca and mola with some minute denticles toward prostheca. Otherwise, as typical for subgenus. ***Hypopharynx*** (Fig. 110c). As typical for genus. ***Maxilla*** (Fig. 111c, d). Maxillary palp slightly longer than galea-lacinia, slender; palp segment II ~ 1.3× length of segment I. Otherwise, as typical for genus. ***Labium*** (Fig. 111a, b). As typical for the genus. Paraglossa dorsally with two spine-like setae near inner, distolateral margin. Labial palp with segment I ~ 1.2× length of segments II and III combined. Segment II with slight, broadly rounded, distomedial protuberance, dorsally with row of five spine-like setae near outer, distolateral margin. Segment III slightly pentagonal, pointed, 0.6× length of segment II.

***Thorax*. *Sterna*.** With small protuberances on sides of prosternum and close to openings of mesothoracic and metathoracic sternal apodemes (as Fig. 108a). ***Terga*** (Figs 109b, 113b). Pronotum with paired, blunt, posteromedial protuberances. Metanotum with medium, pointed, dorsally oriented, medial protuberance. ***Legs*** (Fig. 112a–e). Ratio of leg segments: fore leg 1.0:1.0:0.3:0.1, middle leg 1.1:1.0:0.3:0.1 and hind leg 1.2:1.0:0.3:0.1. ***Femur*.** Length ~ 3× maximum width; inner margin with irregular, dense row of medium, fine setae. ***Tibia*.** Outer margin with irregular, dense row of medium, fine setae. ***Claw*** with one row of eight or nine denticles and one posterior seta.

***Abdomen*. *Terga*** (Fig. 113a, c–f). Terga I–IX with medium, pointed, dorsoposteriorly oriented, medial protuberances. Posterior margin of terga III–IX with variable, triangular, pointed denticles, spaced on terga III–VI. ***Tergalii*** (Fig. 111f, g). Skew ovoid, tracheation well developed; margins smooth, with short, fine, simple setae. ***Paraproct*** (Fig. 111e). Posterior margin membranous, with prolongation and row of minute denticles. ***Caudalii*** (Fig. 112f–h). Cerci without swimming setae, with bilateral rows of minute setae. Paracercus vestigial.

***Pose of subimaginal gonostyli under larval cuticle*.** Unknown.

**Subimago.** Unknown.

**Imago.** Unknown.

**Egg.** Unknown.

#### Distribution.

New Guinea (Fig. 147).

### ﻿﻿ Papuanatula (Papuanatula) pluresetae sp. nov.

Taxon classificationAnimaliaEphemeropteraBaetidae

EDD2250F-CAF4-5D02-B9FD-A7198F527BE3

https://zoobank.org/2ACB9D14-BEC8-4D16-ABA6-D999B05F73B2

**Figure 114.**
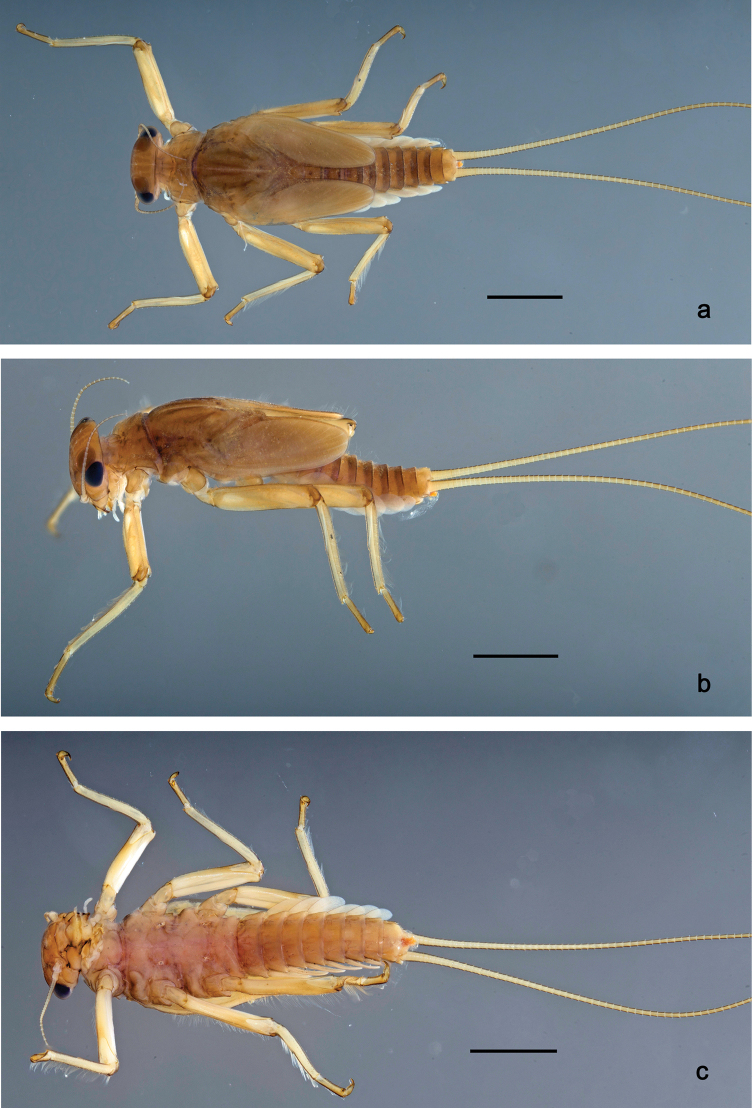
Papuanatula (Papuanatula) pluresetae sp. nov., larva, habitus: **a** dorsal view **b** lateral view **c** ventral view. Scale bars: 1 mm.

**Figure 115.**
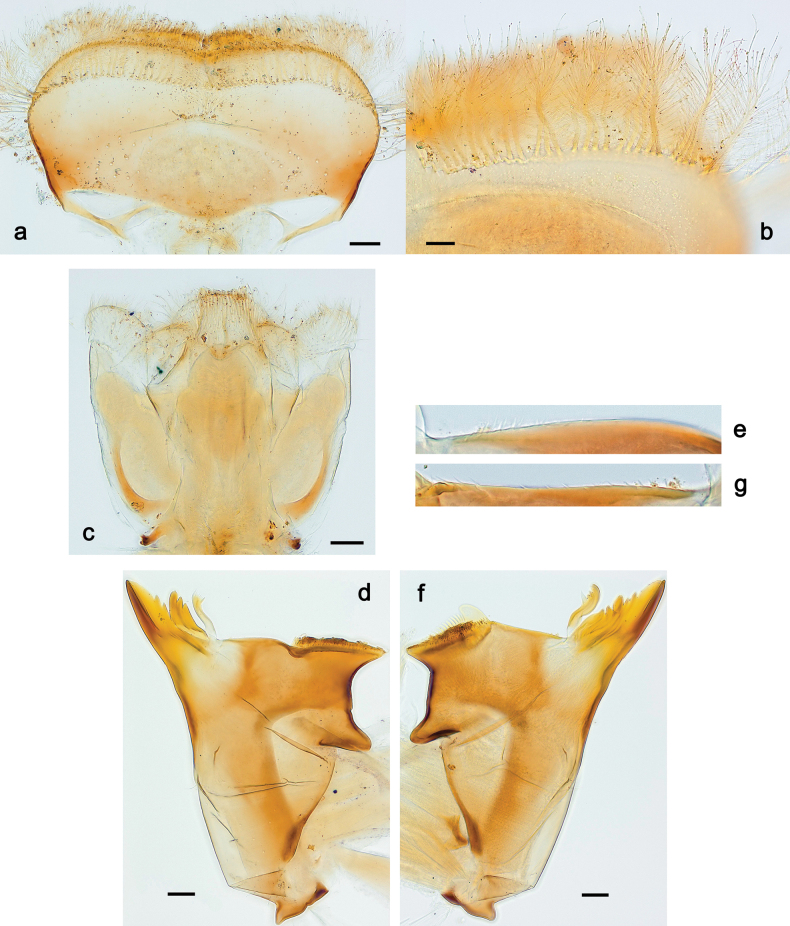
Papuanatula (Papuanatula) pluresetae sp. nov., larva: **a** labrum **b** labrum: dorsal submarginal setae **c** hypopharynx and superlinguae **d** right mandible **e** right mandible: margin between prostheca and mola **f** left mandible **g** left mandible: margin between prostheca and mola. Scale bars: 20 µm (**a, c, d, f**); 10 µm (**b**).

**Figure 116.**
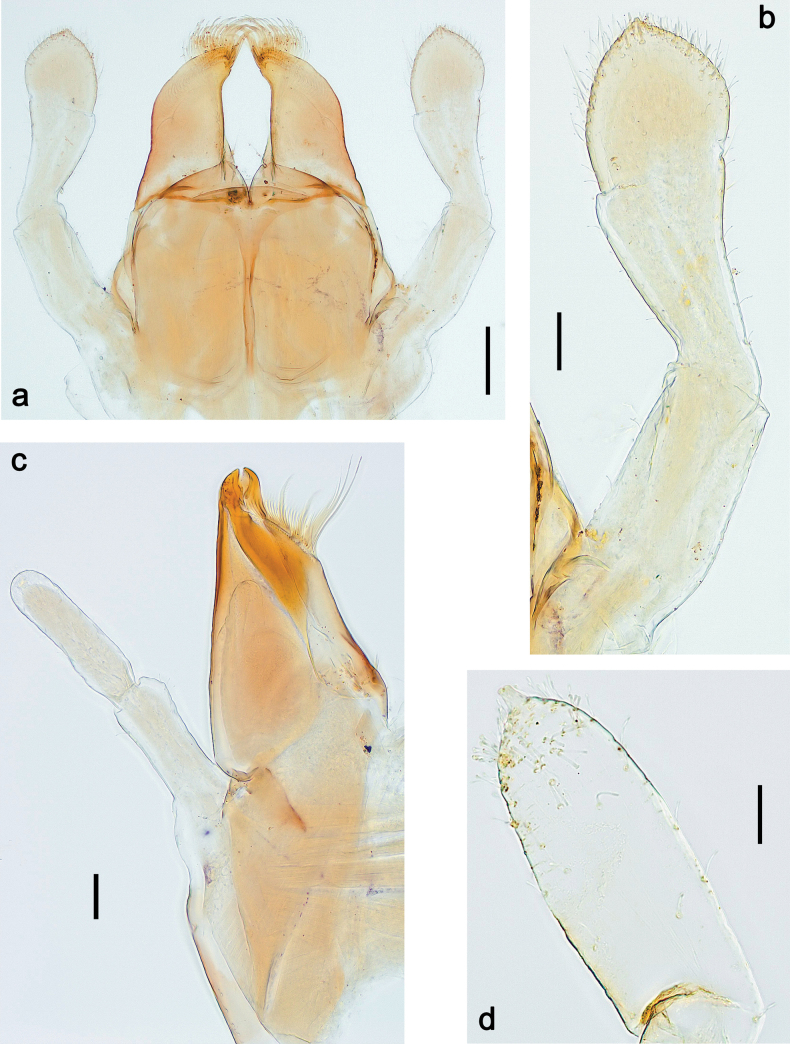
Papuanatula (Papuanatula) pluresetae sp. nov., larva: **a** labium **b** labial palp **c** maxilla **d** maxillary palp. Scale bars: 50 µm (**a**); 20 µm (**b, c**); 10 µm (**d**).

**Figure 117.**
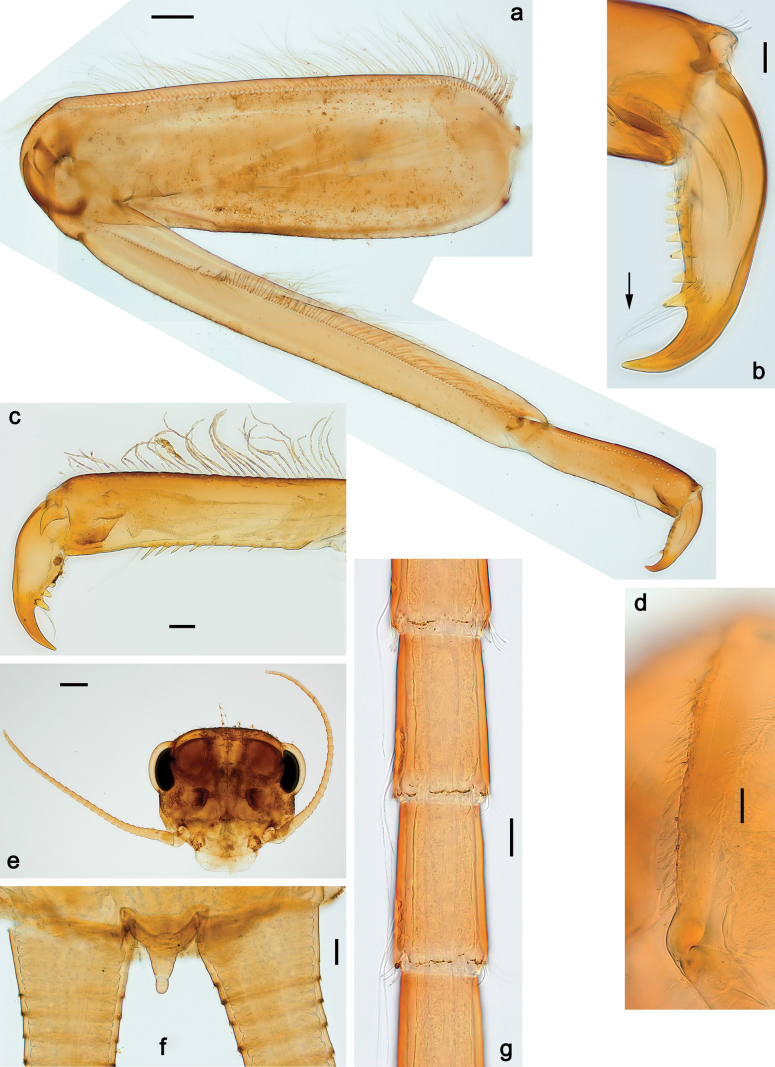
Papuanatula (Papuanatula) pluresetae sp. nov., larva: **a** fore leg **b** fore claw (arrow: posterior setae) **c** fore tarsus and claw **d** middle femur, posterior apex **e** male head, mature **f** paracercus **g** cercus. Scale bars: 50 µm (**a**); 10 µm (**b–g**); 100 µm (**h**).

**Figure 118.**
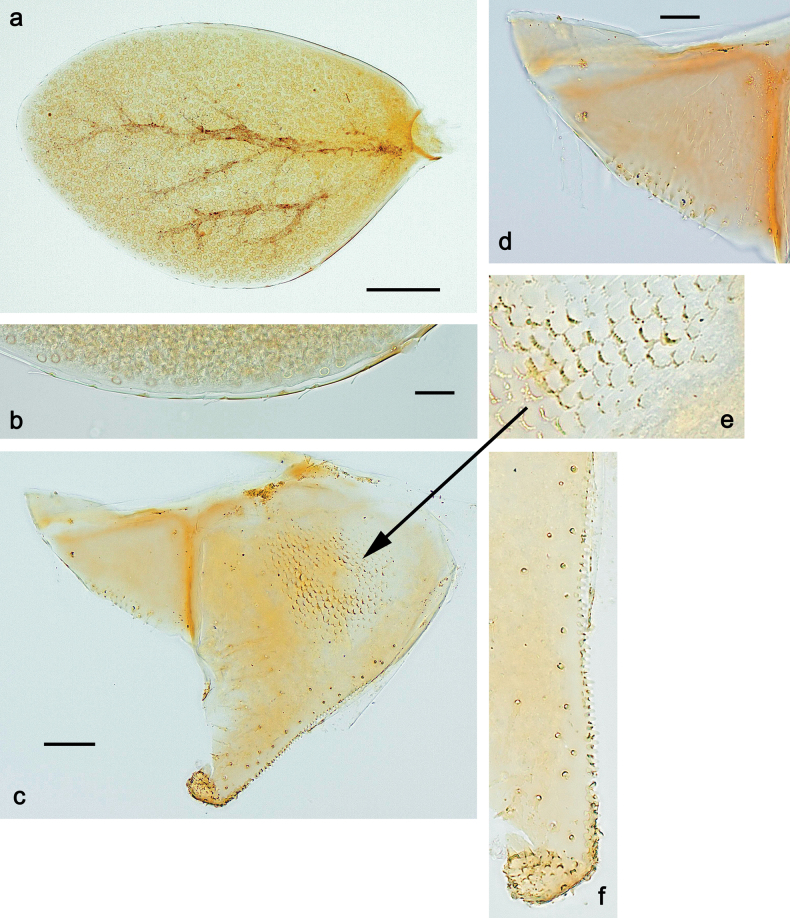
Papuanatula (Papuanatula) pluresetae sp. nov., larva: **a** tergalius IV **b** tergalius IV, margin **c** paraproct **d** cercotractor **e** section of paraproct with notched scales **f** paraproct, distal margin. Scale bars: 50 µm (**a**); 10 µm (**b, d**); 20 µm (**c**).

**Figure 119.**
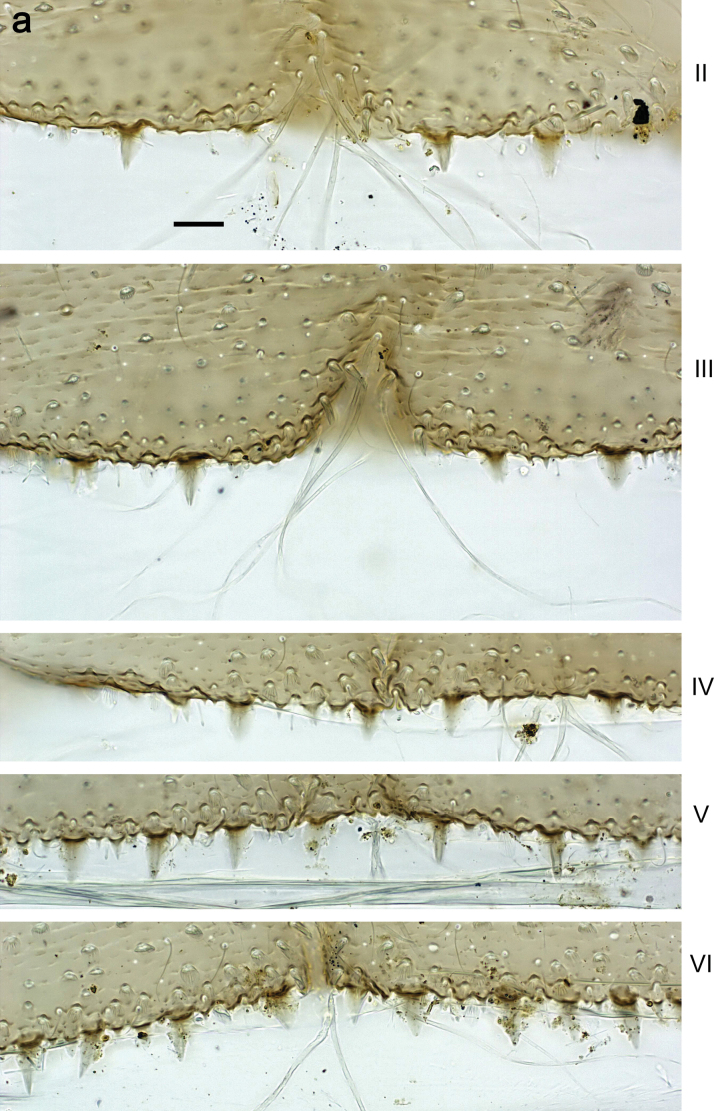
Papuanatula (Papuanatula) pluresetae sp. nov., larva: **a** abdominal terga. Scale bar: 10 µm (**a**).

**Figure 120.**
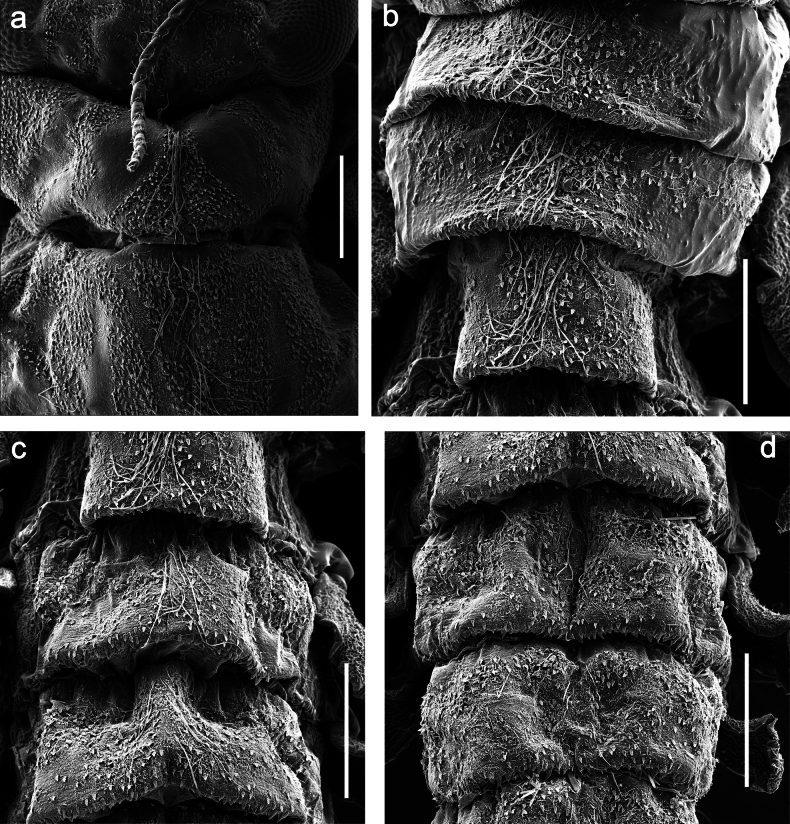
Papuanatula (Papuanatula) pluresetae sp. nov., larva (SEM): **a** pro- and mesonotum **b** abdominal terga II–IV **c** abdominal terga IV–VI **d** abdominal terga VI–VIII. Scale bars: 200 µm.

Figs 114 , 115 , 116 , 117 , 118 , 119 , 120

#### Etymology.

The species name *pluresetae* is based on the Latin words *plures setae* meaning “several setae” and refers to the three or four posterior setae on the claws.

#### Material examined.

***Holotype*.** Papua New Guinea • larva; Simbu Prov., Mt. Wilhelm, Pindaunde Creek, S2 (oria 3); 05°48'03"S, 145°04'09"E; 3210 m; 17.viii.1999; leg. L. Čížek; on slide; GBIFCH00592587, GBIFCH00592588; MZL. ***Paratypes*.** 78 larvae; same data as holotype; 6 on slides; GBIFCH00592548, GBIFCH00592578, GBIFCH00592579, GBIFCH00592589, GBIFCH00592590, GBIFCH00592591, GBIFCH00592592, GBIFCH01221756; 72 in alcohol; GBIFCH00976070, GBIFCH00976100, GBIFCH00976101, GBIFCH00976120; MZL • 62 larvae; Simbu Prov., Mt. Wilhelm, Pindaunde Creek, near fish farm, S4 (oria 5); 05°49'02"S, 145°05'16"E; 2600 m; 18.viii.1999; leg. L. Čížek; in alcohol; GBIFCH00976123; MZL • 5 larvae; Simbu Prov., Mt. Wilhelm, Pindaunde Creek, in forest, S3 (oria 4); 05°49'S, 145°04'30"E; 2900 m; 18.viii.1999; leg. L. Čížek; in alcohol; GBIFCH00976116; MZL.

#### Diagnosis.

**Larva**. The following combination of characters distinguishes *P.pluresetae* sp. nov. from other species of *Papuanatula* s. str.: body dorsally with irregular row of long, fine, simple setae along midline; abdominal terga without protuberances; femur basally with short, wedge-shaped blank; otherwise, brown (after 35 years in alcohol); paracercus vestigial; claw with three or four posterior setae.

#### Description.

**Larva** (Figs 114–120). Body length 4.5–5.8 mm, cerci much longer than body length (~ 1.3×).

***Cuticular coloration*** (Fig. 114a–c; after 35 years in alcohol). Head, thorax and abdomen dorsally brown. Femur basally with short, wedge-shaped blank; otherwise, brown. Tibia pale brown; tarsus darker brown. Head, thorax, and abdomen ventrally brown. Cerci pale brown.

***Hypodermal coloration*** (Fig. 114a, b. Abdominal terga I–IX with narrow, dark brown transverse band along posterior margins.

***Head*.** Dorsally with irregular row of long, fine, simple setae along midline (as in Fig. 26b). ***Antenna*** (Fig. 117e). Length ~ 1.5× head length. ***Developing turbinate eyes in last instar male larva*** (Fig. 117e) roundish, rather small, with large distance to each other. ***Labrum*** (Fig. 115a, b). Length 0.5× maximum width, laterally convex. Dorsal, sub-marginal arc with ~ 40 feathered setae. ***Right mandible*** (Fig. 115d, e). Margin between prostheca and mola with row of minute denticles. Otherwise, as typical for subgenus. ***Left mandible*** (Fig. 115f, g). Margin between prostheca and mola with row of minute denticles. Otherwise, as typical for subgenus. ***Hypopharynx*** (Fig. 115c). As typical for genus. ***Maxilla*** (Fig. 116c, d). Maxillary palp subequal in length to galea-lacinia, robust; palp segment II as long as segment I. Otherwise, as typical for genus. ***Labium*** (Fig. 116a, b). As typical for the genus. Paraglossa dorsally with two spine-like setae near inner, distolateral margin. Labial palp with segment I 0.9× length of segments II and III combined. Segment II with minute distomedial protuberance, dorsally with row of four spine-like setae near outer, distolateral margin. Segment III conical, pointed; 0.7× length of segment II; inner dorsal margin with few feathered setae.

***Thorax*. *Sterna*.** With small protuberances on sides of prosternum and close to openings of mesothoracic and metathoracic sternal apodemes (as Fig. 108a). ***Terga*** (Fig. 120a) without protuberances; with irregular row of long, fine, simple setae along midline (as in Fig. 26b). ***Legs*** (Fig. 117a, d). Ratio of leg segments: fore leg 0.9:1.0:0.3:0.2, middle leg 0.9:1.0:0.3:0.2 and hind leg 1.1:1.0:0.4:0.2. ***Femur*.** Length ~ 3× maximum width. ***Claw*** with one row of seven or eight denticles and three or four posterior setae.

***Abdomen*. *Terga*** (Figs 119a, 120b–d) with irregular row of long, fine, simple setae along midline (as in Fig. 26b). Terga without protuberances, terga I–IV with slight, paired, medioposterior elevations. Posterior margin of terga: I smooth, without denticles; II–IX with triangular, pointed denticles, partly with minute, pointed denticles in between. Surface with scattered small, triangular, pointed, striated scales. ***Tergalii*** (Fig. 118a, b). Ovoid, tracheation poorly developed; margins smooth, with short, fine, simple setae. ***Paraproct*** (Fig. 118c–f). Posterior margin with prolongation and row of minute denticles; on surface an area with notched scales. ***Caudalii*** (Fig. 117f, g). Cerci apart from basal and distal part with 1–4 swimming setae per segment, initially increasing and then again decreasing toward distal part. Paracercus vestigial.

***Pose of subimaginal gonostyli under larval cuticle*.** As typical for the subgenus.

**Subimago.** Unknown.

**Imago.** Unknown.

**Egg.** Unknown.

#### Distribution.

New Guinea (Fig. 148).

### ﻿﻿ Papuanatula (Papuanatula) webbi sp. nov.

Taxon classificationAnimaliaEphemeropteraBaetidae

0C5C2BE9-C0A7-56DF-9D30-E0C0C10D840B

https://zoobank.org/FCC39C92-1B65-474C-BAFA-AEE5FAE3BA3F

**Figure 121.**
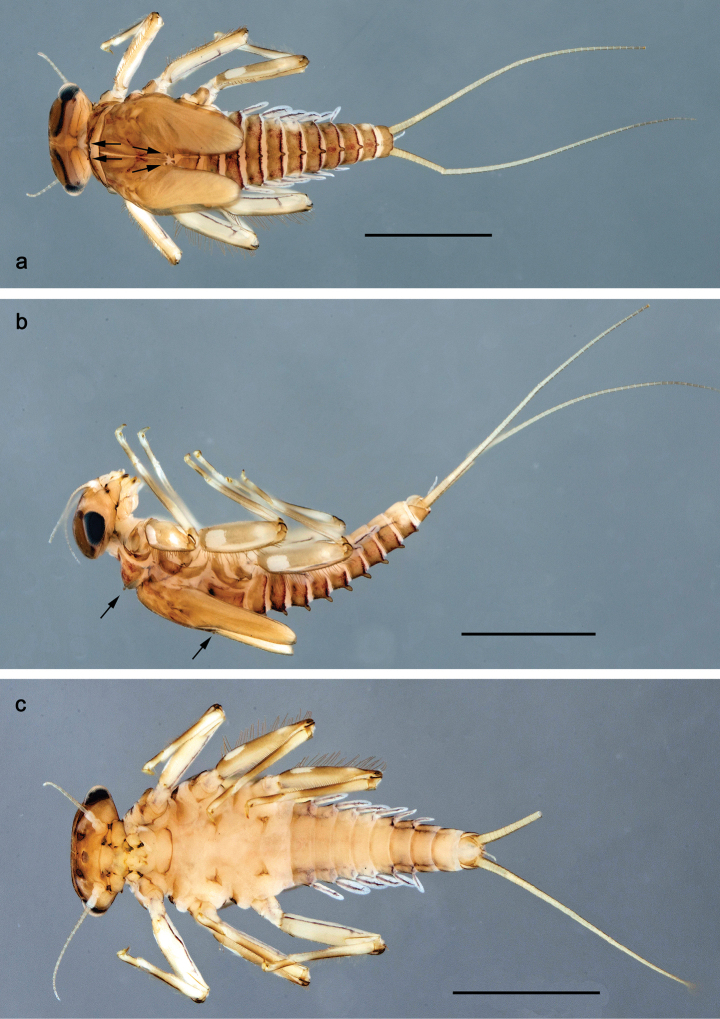
Papuanatula (Papuanatula) webbi sp. nov., larva, habitus: **a** dorsal view **b** lateral view **c** ventral view. Scale bars: 1 mm.

**Figure 122.**
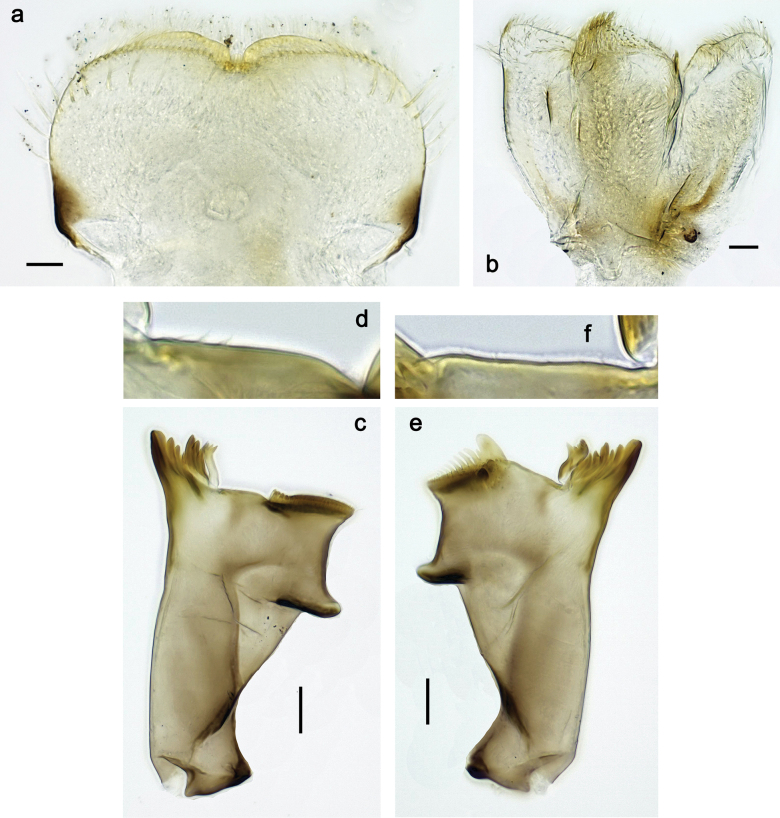
Papuanatula (Papuanatula) webbi sp. nov., larva: **a** labrum **b** hypopharynx and superlinguae **c** right mandible **d** right mandible: margin between prostheca and mola **e** left mandible **f** left mandible: margin between prostheca and mola. Scale bars: 10 µm (**a, b**); 20 µm (**c, e**).

**Figure 123.**
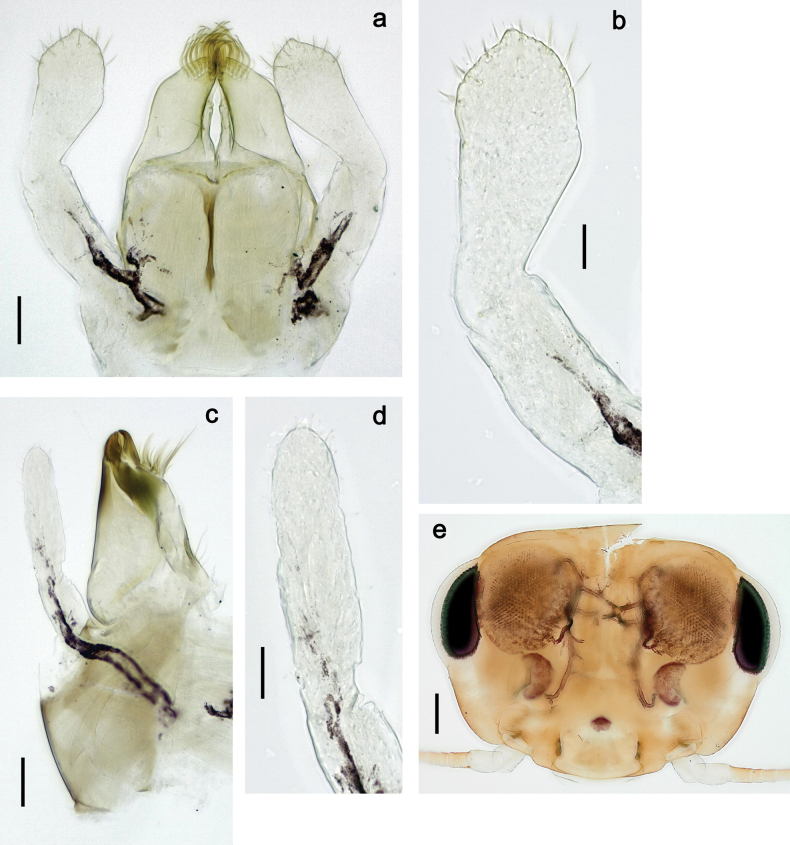
Papuanatula (Papuanatula) webbi sp. nov., larva: **a** labium **b** labial palp **c** maxilla **d** maxillary palp **e** male head, mature. Scale bars: 20 µm (**a, c**); 10 µm (**b, d**); 100 µm (**e**).

**Figure 124.**
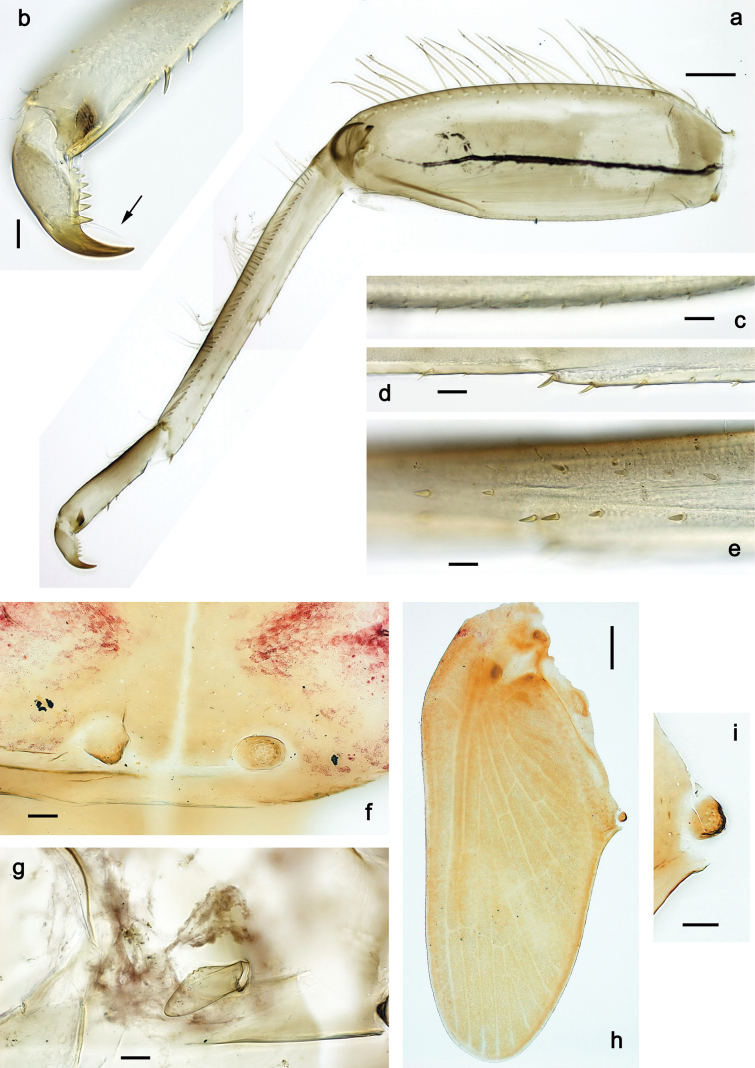
Papuanatula (Papuanatula) webbi sp. nov., larva: **a** hind leg **b** hind tarsus and claw (arrow: posterior seta) **c** hind femur, ventral margin **d** hind tibia, ventral margin **e** hind tibia, posterior surface **f** pronotum **g** metanotum with rudimentary hind protopteron **h** fore protopteron **i** fore protopteron protuberance. Scale bars: 50 µm (**a, h**); 10 µm (**b–g, i**).

**Figure 125.**
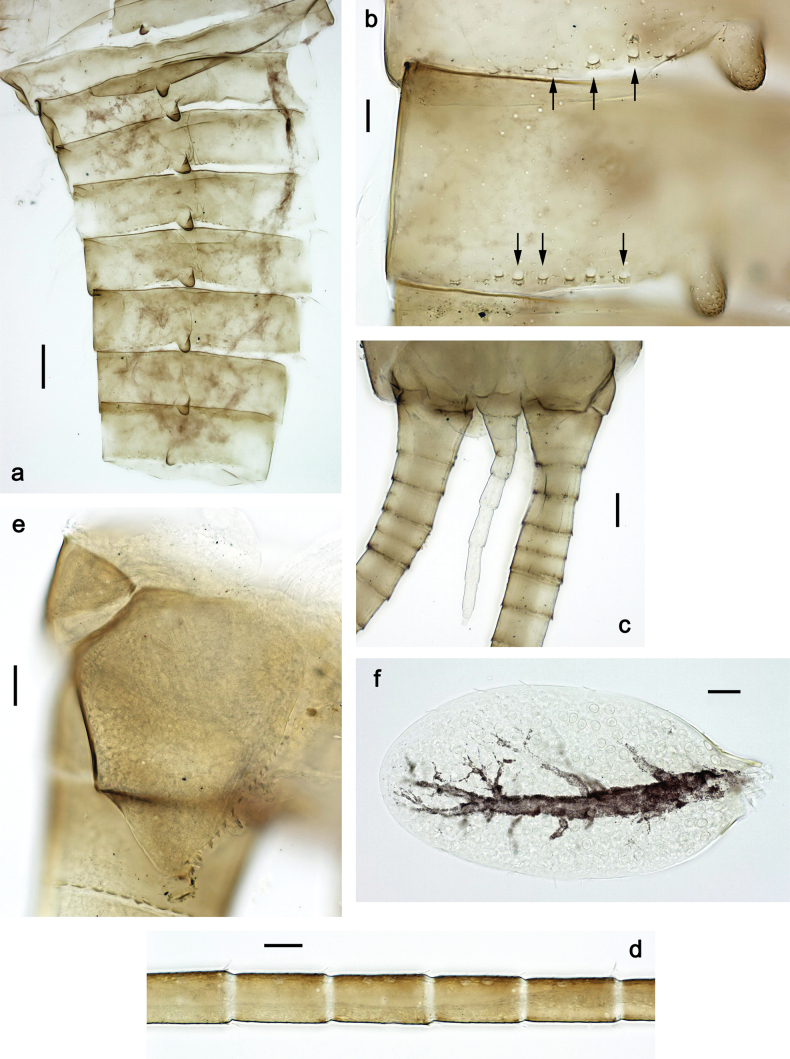
Papuanatula (Papuanatula) webbi sp. nov., larva: **a** abdominal terga I–IX **b** abdominal terga VI–VII (arrows: minute, acute spines) **c** paracercus **d** cercus **e** paraproct **f** tergalius III. Scale bars: 50 µm (**a**); 10 µm (**b, d–f**); 20 µm (**c**).

**Figure 126.**
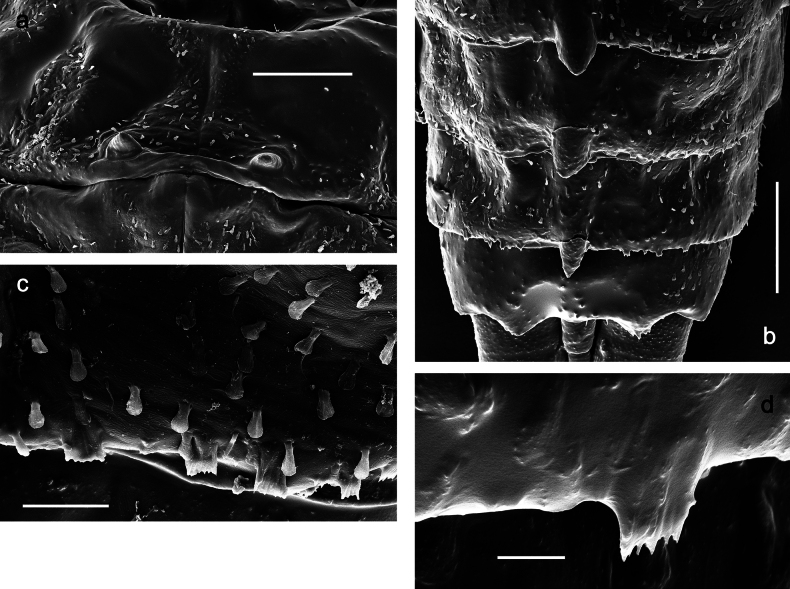
Papuanatula (Papuanatula) webbi sp. nov., larva (SEM): **a** pronotum **b** abdominal terga VII–X **c** abdominal tergum VII, posterior margin **d** spine on posterior margin of abdominal tergum IX. Scale bars: 100 µm (**a, b**); 20 µm (**c**); 5 µm (**d**).

Figs 121 , 122 , 123 , 124 , 125 , 126

#### Etymology.

The species is dedicated to Jeff Michael Webb (Rhithron Associates, USA), who was contributing to this study in an early phase.

#### Material examined.

***Holotype*.** Indonesia • larva; Papua Barat, Kebar to Aibogar, slow forest stream; 00°51'45"S, 132°49'48"E; 503 m; 04.xi.2013; leg. M. Balke: (BH025); on slide; GBIFCH01221788; MZB. ***Paratypes*.** 16 larvae; same data as holotype; 2 on slide; GBIFCH01221798, GBIFCH01221790, GBIFCH01221804; MZL; 14 in alcohol; GBIFCH00975827, GBIFCH00975834, GBIFCH00975835, GBIFCH00975837, GBIFCH00975838, GBIFCH00975840, GBIFCH00975844, GBIFCH00975901; MZL.

#### Diagnosis.

**Larva**. The following combination of characters distinguishes *P.webbi* sp. nov. from other species of *Papuanatula* s. str.: body dorsally without row of long, fine, simple setae along midline; pronotum with pair of small, triangular, apically rounded protuberances near medioposterior margin; fore protoptera with pair of minute protuberances at medioposterior margin; metanotum and abdominal terga I–IX posteromedially with small to medium protuberance, oriented dorsoposteriad; femur with subquadrangular blank in basal area and long, narrow blank along dorsal margin; paracercus with eight segments.

#### Description.

**Larva** (Figs 121–126). Body length 2.4–3.2 mm, cerci much longer than body length (~ 1.5×).

***Cuticular coloration*** (Fig. 121a–c). Head, thorax and abdomen dorsally brown. Femur grey-brown, with subquadrangular blank in basal area, long, narrow blank along dorsal margin and rounded blank in distal area; tibia and tarsus ecru, in medial area grey-brown. Head, thorax and abdomen ventrally pale brown, protuberances of thoracic sterna brown, abdominal segments II–VIII slightly darker than thorax. Cerci yellow-brown.

***Hypodermal coloration*** (Fig. 121a, b). Abdominal terga I–IX with narrow, dark brown transverse band close to posterior margin.

***Head*. *Antenna*** (Fig. 121a–c). Length 1.5× head length. As typical for subgenus. ***Developing turbinate eyes in last instar male larva*** (Fig. 123e) rather large, but with distance to each other. ***Labrum*** (Fig. 122a). Relatively small, length 0.5× maximum width, laterally slightly convex. Dorsal, sub-marginal arc with ~ 10 feathered setae. ***Right mandible*** (Fig. 122c, d). Margin between prostheca and mola with row of minute denticles. Otherwise, as typical for subgenus. ***Left mandible*** (Fig. 122e, f). Margin between prostheca and mola with few minute denticles. Otherwise, as typical for subgenus. ***Hypopharynx*** (Fig. 122b). As typical for genus. ***Maxilla*** (Fig. 123c, d). Maxillary palp subequal in length to galea-lacinia; palp segment II ~ 1.7× length of segment I. Otherwise, as typical for genus. ***Labium*** (Fig. 123a, b). As typical for the genus. Paraglossa dorsally with two spine-like setae near inner, distolateral margin. Labial palp with segment I ~ 1.2× length of segments II and III combined. Segment II without distomedial protuberance, dorsally with row of four spine-like setae near outer, distolateral margin. Segment III conical, pointed, 0.7× length of segment II.

***Thorax*. *Sterna*.** With small protuberances on sides of prosternum and close to openings of mesothoracic and metathoracic sternal apodemes (as in Fig. 108a). ***Terga*** (Figs 124f–I, 126a). Pronotum with pair of small, triangular, apically rounded protuberances near posteromedial margin; metanotum posteromedially with small, apically rounded protuberance. Fore protoptera with pair of minute protuberances at medioposterior margin. ***Hind protoptera*** (Fig. 124g). Absent, but sometimes with vestiges. ***Legs*** (Fig. 124a–e). Ratio of leg segments: fore leg 0.9:1.0:0.3:0.1, middle leg 0.9:1.0:0.3:0.1 and hind leg 1.0:1.0:0.4:0.2. ***Femur*.** Length ~ 3× maximum width. ***Claw*** with one row of six denticles; one posterior seta.

***Abdomen*. *Terga*** (Figs 125a, b, 126b–d). Terga I–IX posteromedially with small to medium, apically rounded protuberance, oriented dorsoposteriad. Posterior margin of terga: I–III smooth, without denticles; IV–IX with short, rounded denticles, apically carrying minute, fine, acute spines. Surface with scattered small, spoon-shaped, striated scales. ***Tergalii*** (Fig. 125f). Narrow oblong, tracheation with strongly developed trunk, other tracheation poorly developed; margins smooth, with few short, fine, simple setae. ***Paraproct*** (Fig. 125e). Posterior margin with prolongation and row of minute denticles. ***Caudalii*** (Fig. 125c, d). Cerci without swimming setae, in middle part one or two insertions per segment still visible, and sometimes with a short, rudimentary swimming seta. Paracercus with eight segments.

***Pose of subimaginal gonostyli under larval cuticle*.** Unknown.

**Subimago.** Unknown.

**Imago.** Unknown.

**Egg.** Unknown.

#### Distribution.

New Guinea (Fig. 148).

### ﻿﻿ Papuanatula (Papuanatula) zebrata sp. nov.

Taxon classificationAnimaliaEphemeropteraBaetidae

94C0A045-2CA0-519E-979C-BDDEE814C6DF

https://zoobank.org/DD6997FD-48B1-4377-8ED9-7F0E24F5F273

**Figure 127.**
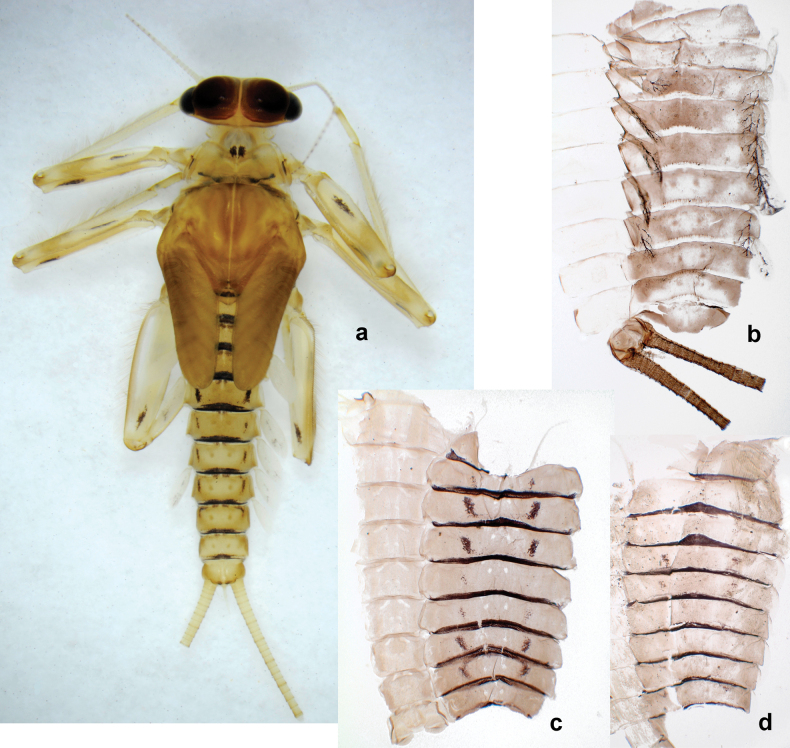
Papuanatula (Papuanatula) zebrata sp. nov.: **a** male larva **b** cuticle of larval abdomen **c** abdomen of male subimago extracted from larva **d** larval abdomen with hypodermal coloration (**b, c** holotype).

**Figure 128.**
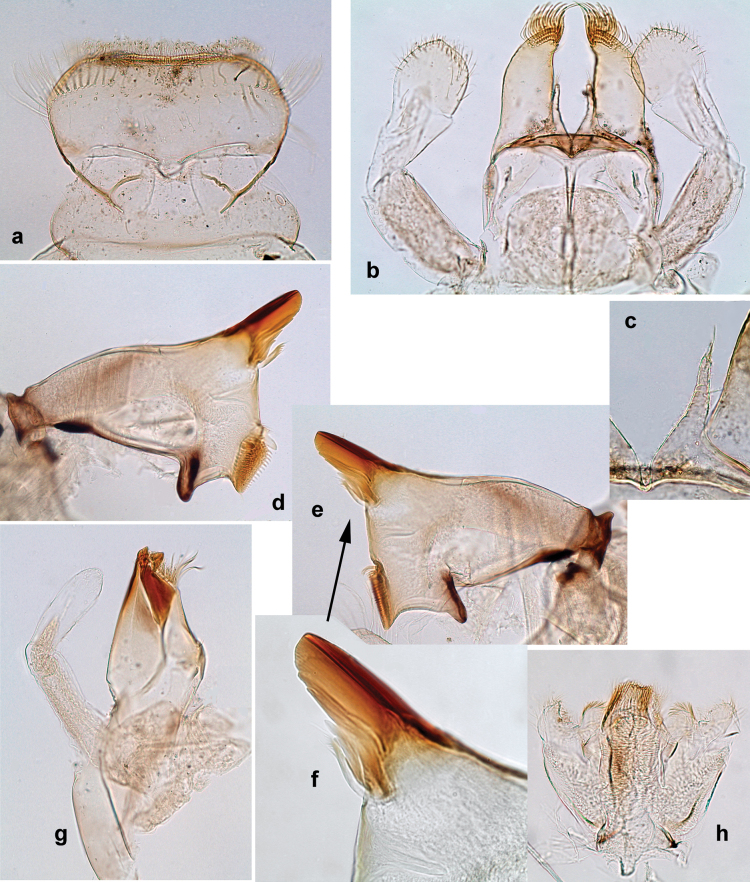
Papuanatula (Papuanatula) zebrata sp. nov., larva: **a** labrum **b, c** labium **d–f** left and right mandibles **g** maxilla **h** hypopharynx with superlinguae (**a, b** holotype).

**Figure 129.**
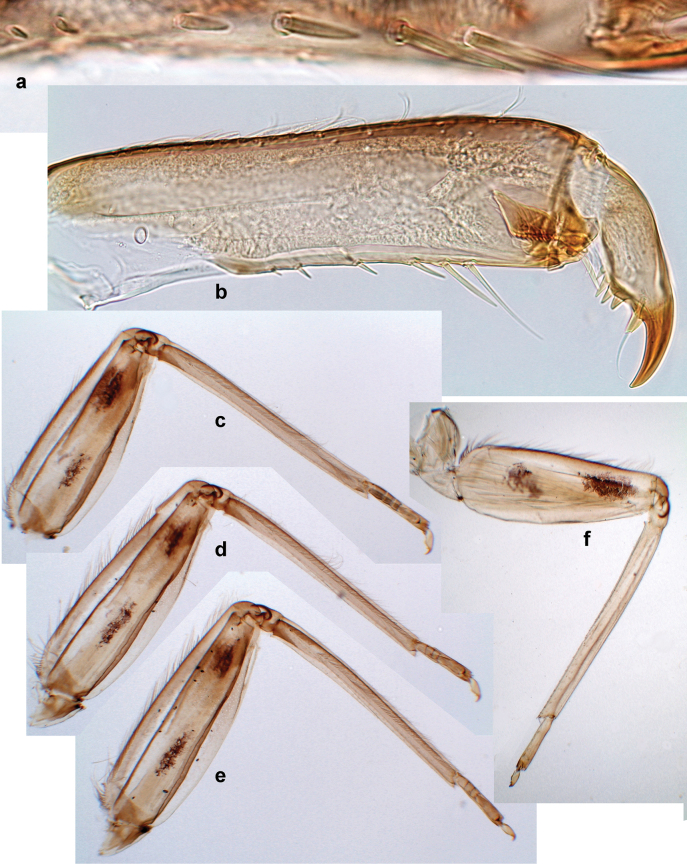
Papuanatula (Papuanatula) zebrata sp. nov., larva: **a** setae on inner side of middle tarsus **b** tarsus and claw **c–e** fore, middle, and hind legs with developing subimaginal legs inside **f** fore leg (**a, c–e** holotype).

**Figure 130.**
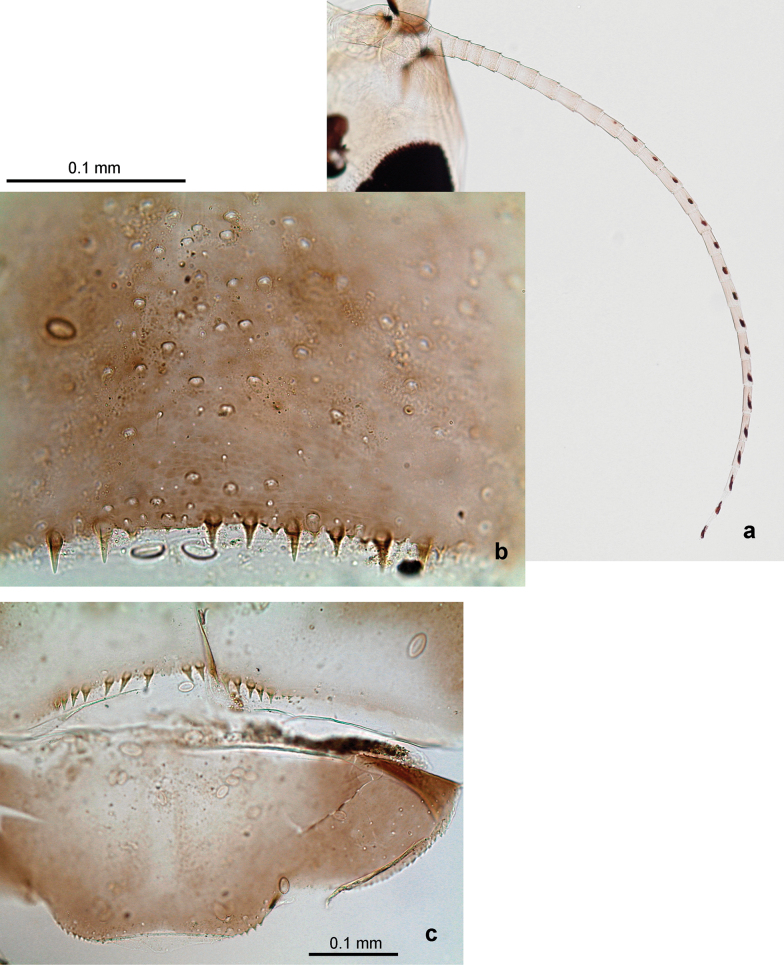
Papuanatula (Papuanatula) zebrata sp. nov., larva: **a** antenna **b** abdominal tergum V **c** abdominal terga IX–X (**b, c** holotype).

**Figure 131.**
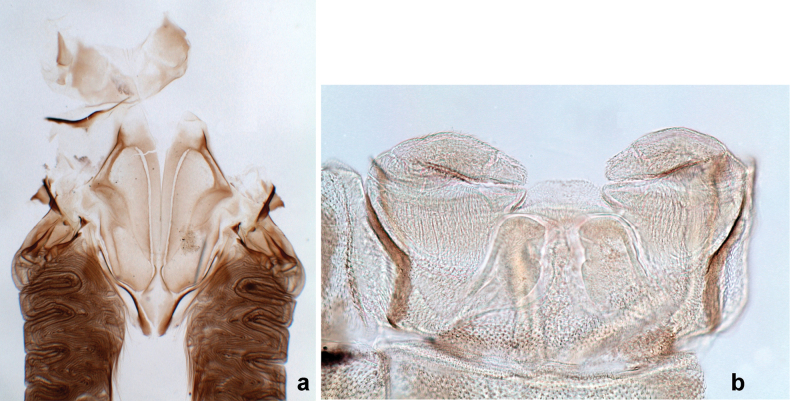
Papuanatula (Papuanatula) zebrata sp. nov., subimagines: **a** cuticle of female subimaginal mesonotum **b** male subimaginal genitalia developing under larval exuviae (**b** holotype).

Figs 127 , 128 , 129 , 130 , 131

#### Etymology.

The species name *zebrata* refers to the hypodermal coloration of larval abdomen (and probably that of winged stages) which includes contrasting, dark brown transverse bands on posterior margins of terga (Fig. 127a, d).

#### Material examined.

***Holotype*.** Male larva ready to molt to subimago; INDONESIA • Papua, Baliem valley, Wamena, river Elagaima; 15–19.viii.2012; coll. N. Kluge & L. Sheyko; SPbU. ***Paratypes*.** same data as holotype, 25 larvae; SPbU.

#### Diagnosis.

**Larva.** The following combination of characters distinguishes *P.zebrata* sp. nov. from other species of *Papuanatula* s. str.: body without row of long, fine setae along midline; abdominal terga without protuberances; femur with brown, hypodermal streak in basal 1/2 and brown spot in distal area; tergalii colorless; posterior margin of abdominal terga with heterogenous, sharply pointed denticles.

#### Description.

**Larva** (Figs 127–130). ***Cuticular coloration*.** Head, pronotum, mesonotum and metanotum ochre with brownish areas; fore protoptera nearly uniformly ochre (Fig. 127a). Thoracic pleura brownish, sterna mostly colorless. Cuticle of femur with brownish margins and large blank occupying most part of proximal 1/2 (Fig. 129c–f). Tibia and tarsus mostly ochre (Fig. 129c–f). Abdominal terga brownish with paler blanks; each tergum VI–IX with median blank and pair of brown sigilla inside it. Sterna mostly colorless (Fig. 127b). Cerci uniformly pale brownish.

***Hypodermal coloration*.** Anterior side of each femur with roundish or longitudinal, dark brown macula on proximal 1/2; posterior side of each femur with or without brown macula on distal 1/2 (Fig. 129c–f). Each abdominal tergum I–IX with contrasting, dark brown band on posterior margin; some terga also with pair of brown spots (Fig. 127d). Tissues surrounding tracheae of tergalii either with brown pigmentation (Fig. 127b), or without pigmentation, so that tracheae poorly visible (Fig. 127a).

***Head*. *Antenna*** (Fig. 130a). Length ~ 2× head length. As typical for subgenus. ***Developing turbinate eyes in last instar male larva*** with facets equally developed on middle and periphery areas (as in Fig. 32d). ***Labrum*** (Fig. 128a) slightly widened distally; long setae on dorsal surface forming regular transverse row; each seta pointed, with moderately long processes on both sides. ***Right mandible*** (Fig. 128e, f). Incisor with indistinct denticles near base; kinetodontium slightly separated from incisor and terminated with three denticles, with distal denticle longest. ***Left mandible*** (Fig. 128d). Incisor and kinetodontium non-distinguishable, together with three small denticles proximad of stretched apex of incisor. ***Hypopharynx*** (Fig. 128h) apically with pair of fields of stout, short, setae-like spines on apex. ***Maxilla*** (Fig. 128g). Maxillary palp as long as galea-lacinia. Otherwise, as typical for genus. ***Labium*** (Fig. 128b, c). Paraglossae widest at base and narrowing toward apex; three apical setal rows bent at apex of paraglossa. Glossa shorter than half of paraglossa, with finger-like (distal) portion as long as triangular (proximal) portion. Glossa with several long setae in distal 1/2 and one long seta near middle of ventral side. Labial palp without distomedian projection on segment II; segment III with median margin as long as lateral margin.

***Thorax*. *Sterna*** without protuberances. ***Terga*** without protuberances. Metanotum without hind protoptera or their vestiges. ***Legs*** (Fig. 129a–f). Fore femur widened in proximal part; hind tibia shorter than others. ***Femur*.** Outer side of each femur with single regular row of long, hair-like setae bearing numerous fine, short branches on all sides (Fig. 129c–f, as in Figs 41g, 68b). ***Tibia*.** Patella-tibial suture present on all legs, terminated near middle of inner margin of tibia (Fig. 129c–f). Tibia-tarsal condylus turned to anterior side. Anterior side of each tibia with regular row of hair-like setae similar to setae on femur, but smaller (Fig. 129c–f). ***Tarsus*.** Anterior side of each tarsus with regular row of similar, but smaller setae (Fig. 129b). Posterior side of each tarsus with regular row of stout setae; two or three most distal of them elongated and pointed; most distal seta longer than others (but with shape and thickness similar to previous one) (Fig. 129a). ***Claw*** with row of 4–6 denticles and one somewhat larger denticle distad of them; long, arched, posterior seta (Fig. 129b).

***Abdomen*. *Terga*** (Figs 127b, 130b, c) without long setae on midline. Abdominal terga without dorsal unpaired or paired protuberances, only with slightly expressed, unpaired, median elevations. Abdominal terga I–III without denticles or with few denticles on posterior margins; posterior margins of abdominal terga IV–IX with conical, sharply pointed denticles irregularly alternated with smaller pointed denticles. Posterior margin of tergum X mostly smooth, with few pointed denticles on sides. ***Tergalii*** (Fig. 127a, b) of abdominal segment I absent; tergalii II–VII subequal, oval. Each tergalius with costal and anal ribs narrow, smooth, present on proximal 1/2 of tergalius only (as in Fig. 66i). ***Paraproct*** without posterior prolongation; margins membranous, smooth, lacking denticles. ***Caudalii*** (Fig. 127a, b) without swimming setae or their vestiges. Paracercus short, consisting of ~ 8–10 segments.

***Pose of subimaginal gonostyli under larval cuticle*** (Fig. 131b). In mature larva ready to molt to subimago, subimaginal gonostyli packed under larval cuticle in “*Labiobaetis*-type” pose: 2^nd^ segments directed medially and bent proximally; 3^rd^ segment directed medially (as continuation of 2^nd^ segment) and narrowed apically, being deformed corresponding to space between subimaginal styliger and larval cuticle.

**Subimago. *Cuticular coloration*.** Pronotum and mesonotum on Fig. 131a.

***Texture*.** On all legs of both sexes, each tarsomere covered mostly with blunt microlepides, with pointed microlepides near apex (as in Fig. 70i).

**Imago**. Unknown. Judging from larval hypodermal coloration and identical hypodermal coloration of male and female larvae ready to molt to subimago, imagines of both sexes have following features: Femur of each leg pair with two dark brown maculae, one on proximal 1/2 and another on distal 1/2. Each abdominal tergum I–IX with contrasting, dark brown band on posterior margin; some terga also with pair of brown spots (as in Fig. 127d). Judging from fully developed facetted surface of turbinate eyes in larva, male imaginal turbinate eyes are widened distally and have wide facetted surfaces.

**Egg.** Unknown.

#### Dimension.

Body length 4–5 mm.

#### Comparison.

Larva of the new species P. (Papuanatula) zebrata sp. nov. has similarities with *P.lenos* in absence of long hairs and denticles on abdominal terga and presence of brown hypodermal maculae on femora; *P.zebrata* sp. nov. differs from *P.lenos* by absence of special brown markings on abdominal terga IV, VII and VIII, sharper and heterogeneous denticles on posterior margins of abdominal terga and colorless tracheae of tergalii.

#### Distribution.

New Guinea (Fig. 148).

### ﻿﻿Subgenus Papuafiliola subgen. nov.

Taxon classificationAnimaliaEphemeropteraBaetidae

49A29F68-9938-5331-BB36-E82080C2252B

https://zoobank.org/807580D4-6B00-498E-BDC5-DAD5C1B9A50C

#### Type species.

Papuanatula (Papuafiliola) stenophylla sp. nov.

#### Etymology.

The new genus-group name *Papuafiliola* is formed from the nouns Papua and *filiola* (most known Latin word for “little daughter”). Gender is feminine.

#### Diagnosis (larval characters).

***Antenna*** with each flagellomere symmetric, cylindrical, without brown hypodermal spot (Fig. 133b) (in contrast to *Papuanatula* s. str.).

***Labrum*** not enlarged and widest at base, dorsally with few submarginal, simple setae (in contrast to feathered setae in *Papuanatula* s. str.)

***Mandibles*** with incisor shortened (Fig. 134b–e) (in contrast to elongated in *Papuanatula* s. str.).

***Labium***: Glossae longer than half of paraglossae, with finger-like (distal) portion much longer than triangular (proximal) portion. Labial palp with distomedian projection on 2^nd^ segment (Fig. 134h–j) (in contrast to *Papuanatula* s. str.).

***Legs***: outer side of femur with regular row of long, slender, flattened, parallel-sided setae with blunt apices. Tibia and tarsus with regular row of similar setae. Inner margin of tarsus with distalmost seta not longer or only slightly longer than others.

***Paraproct*** without posterior prolongation.

***Pose of subimaginal gonostyli under larval cuticle*** in “*Crassolus*-type” (Kluge et al. 2020; Kluge 2021): gonostylus shortened and bent in articulation of 2^nd^ and 3^rd^ segments; 2^nd^ segment directed caudally-medially and not bent; 3^rd^ segment directed medially-cranially and terminated mediad of 2^nd^ segment. Besides these characters, both known species of Papuafiliola subgen. nov. have common peculiar characters: body, legs, and marginal ribs of tergalii are covered with minute denticles (Figs 135a, b, d, 136k), which form fields of peculiar shape on femora.

**Eggs** of both species with net-like relief (Figs 138a, b, 145a–e).

##### ﻿﻿Species included in Papuafiliola subgen. nov.

Papuanatula (Papuafiliola) stenophylla sp. nov.

Papuanatula (Papuafiliola) tuberculata sp. nov.

### ﻿﻿ Papuanatula (Papuafiliola) stenophylla sp. nov.

Taxon classificationAnimaliaEphemeropteraBaetidae

2D0E5D31-1DEE-532F-87CE-60ED436663FC

https://zoobank.org/45221212-60FC-4F96-AA3F-24011D1D55D2

**Figure 132.**
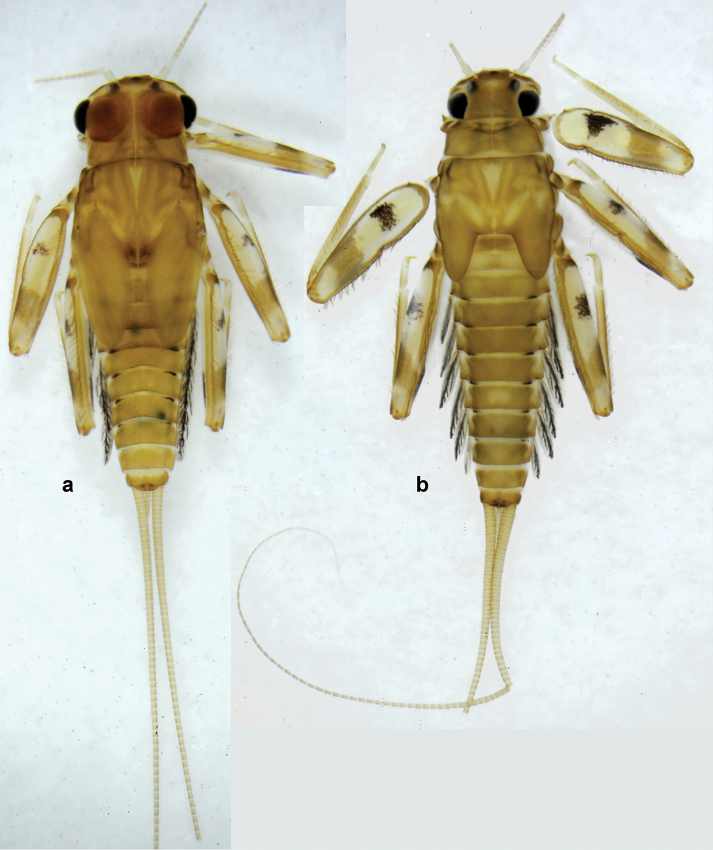
Papuanatula (Papuafiliola) stenophylla sp. nov., larvae.

**Figure 133.**
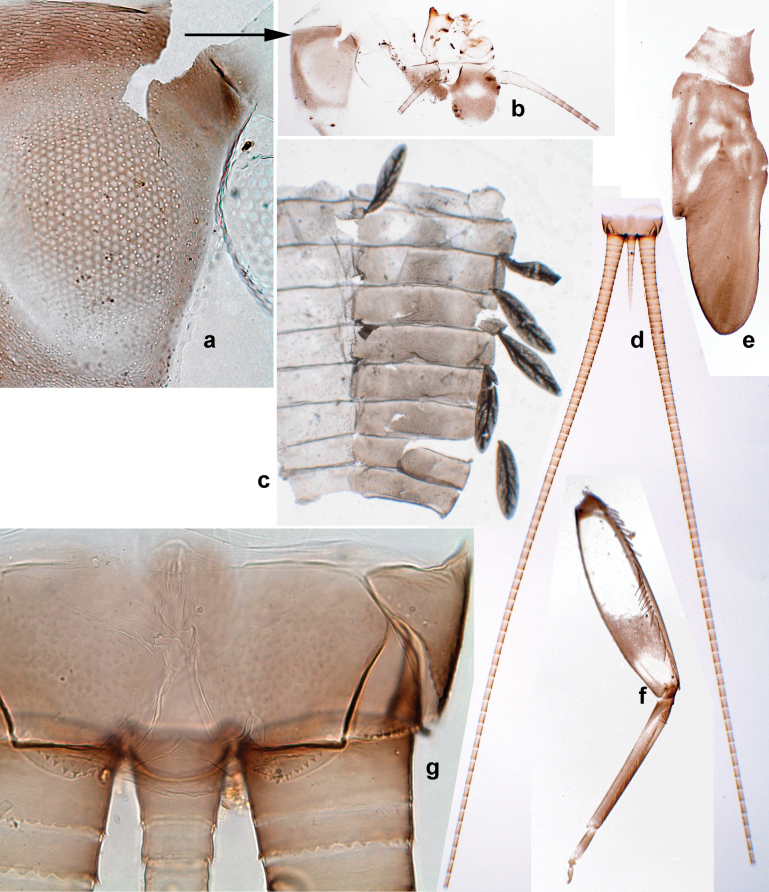
Papuanatula (Papuafiliola) stenophylla sp. nov., larval exuviae (holotype; b–f in one magnification): **a** enlarged dorsal eye of male **b** parts of head **c** abdominal terga and sterna **d** caudalii **e** half of pronotum and mesonotum **f** leg **g** abdominal segment X (ventral view with paraprocts).

**Figure 134.**
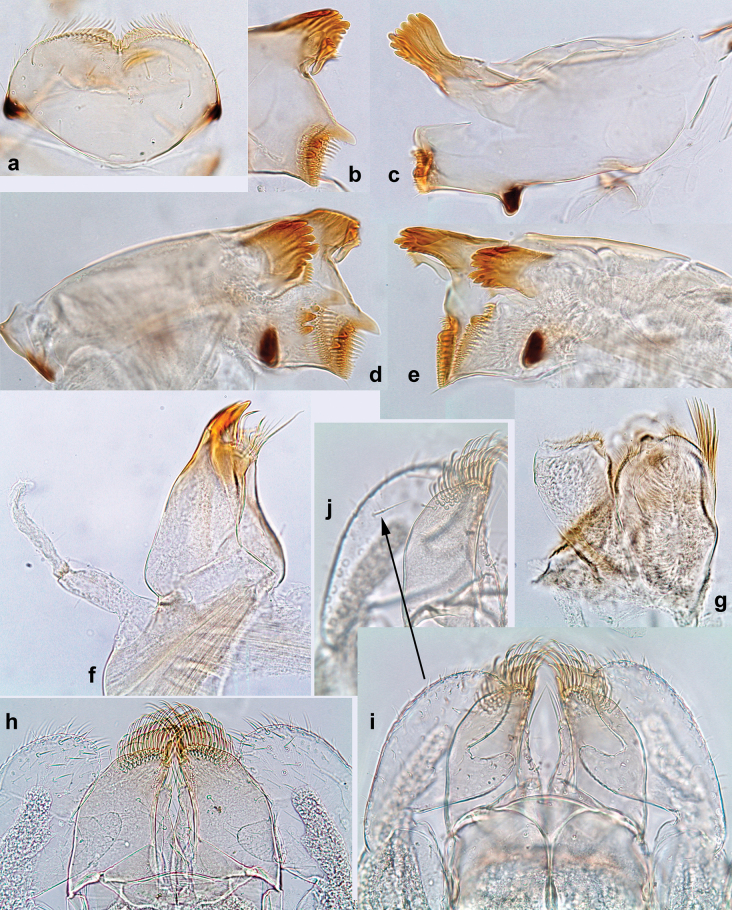
Papuanatula (Papuafiliola) stenophylla sp. nov., larva (**a–c** holotype): **a** labrum **b, c** left and right mandibles **d, e** left and right mandibles with developing mandibles of next instar **f** maxilla **g** hypopharynx (lateral view) **h, i** labium **j** labial palp (dorsal view).

**Figure 135.**
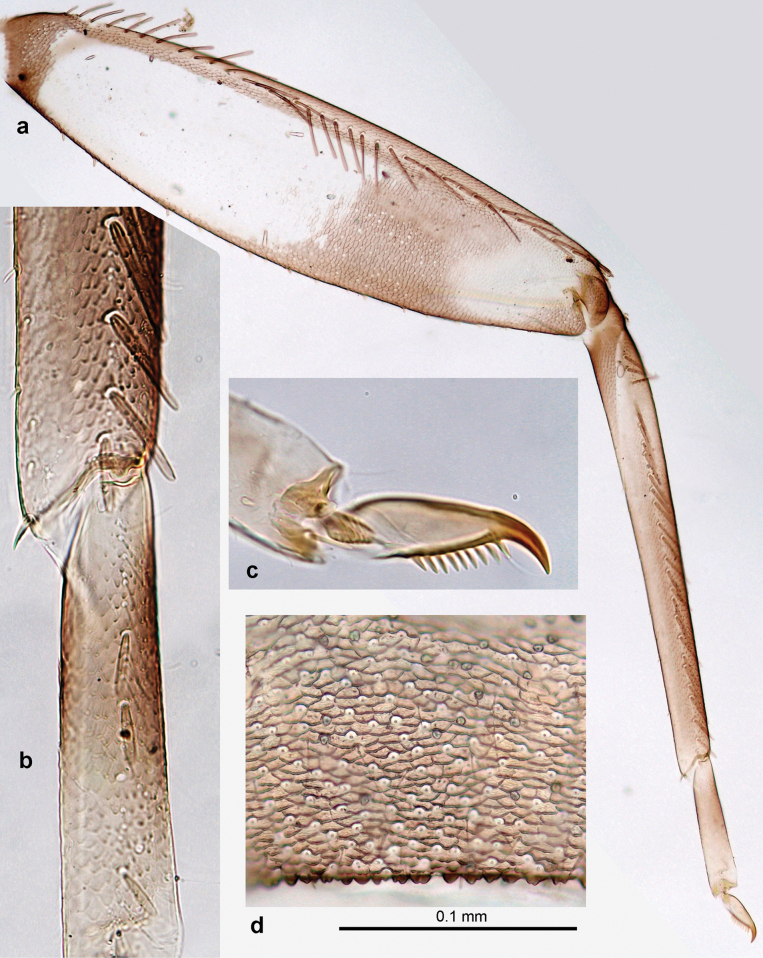
Papuanatula (Papuafiliola) stenophylla sp. nov., larval exuviae (holotype): **a** leg **b** apex of tibia and base of tarsus (dorsal view) **c** claw **d** abdominal tergum VII (dry).

**Figure 136.**
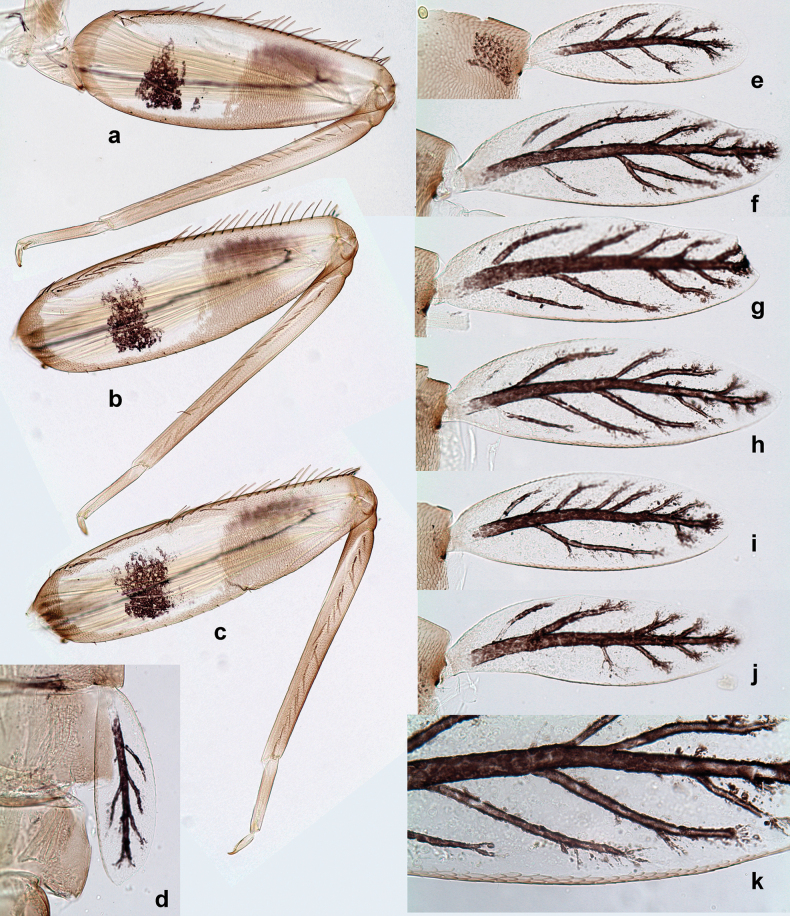
Papuanatula (Papuafiliola) stenophylla sp. nov., larva: **a–c** fore, middle, and hind legs **d** tergalius VII in natural position **e–j** tergalii of II–VII **k** anal rib of tergalius VII.

**Figure 137.**
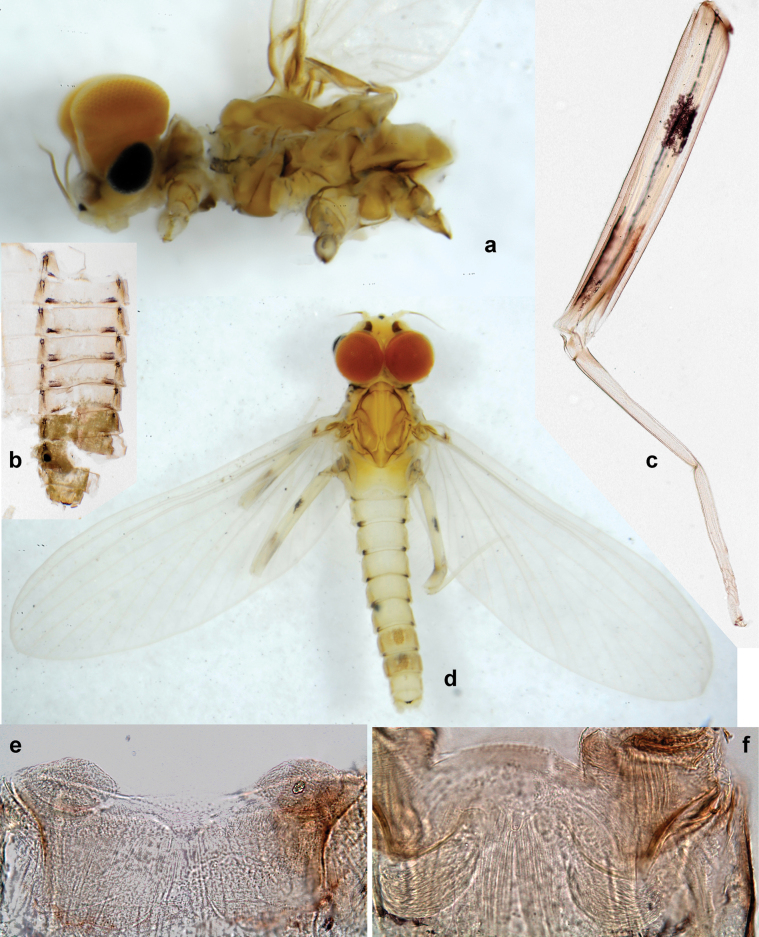
Papuanatula (Papuafiliola) stenophylla sp. nov., male imago (**a–c, f** holotype): **a** head and thorax **b** abdomen **c** hind leg **d** male subimago **e** subimaginal gonostyli developing under larval cuticle **f** styliger and gonovectes of male imago.

Figs 132 , 133 , 134 , 135 , 136 , 137

#### Etymology.

The species name *stenophylla* (from *στενος*—slender, and *φυλλον*—leaf) refers to theslender tergalii.

#### Material examined.

***Holotype*.** L-S/I♂ {specimen number [XX](5)C2012}; Indonesia • Papua, Depapre; 28.viii.2012; coll. N. Kluge & L. Sheyko; SPbU. ***Paratypes*.** Same locality and collectors; 25–28.viii.2012: 2 S♂, 11 larvae; SPbU.

#### Diagnosis.

**Larva.** The following combination of characters distinguishes *P.stenophylla* sp. nov. from the other species of Papuanatula (Papuafiliola): femora and abdomen with brown hypodermal maculae; without median protuberances on abdominal terga; tergalii narrow.

#### Description.

**Larva** (Figs 132–136). ***Cuticular coloration*.** Head, pronotum and mesonotum mostly pale brownish, with some paler areas. Fore protopteron nearly uniformly pale brownish (Fig. 133b, e). Metanotum darker brownish medially, colorless laterally. Thoracic pleura pale brownish, sterna mostly colorless. Cuticle of legs mostly colorless, with brownish outer margin and two brownish transverse bands on anterior surface: one close to base and another in apical part; cuticle of these brownish areas serrate (Fig. 135a, b). Abdominal terga mostly pale brownish; lateral areas of anterior terga paler or colorless; sterna colorless (Fig. 133c). Cerci uniformly pale brownish.

***Hypodermal coloration*.** Anterior side of each femur with wide, contrasting, dark brown macula in middle of area with non-pigmented cuticle (Fig. 136a–c). Abdomen with sublateral, transverse, contrasting, dark brown maculae between terga (Fig. 132a, b). Tissues surrounding tracheae of tergalii (main trachea and its branches) with extensive dark brown pigmentation (Fig. 136d–k).

***Head*. *Antenna*** (Fig. 133b). As typical for subgenus, with each flagellomere symmetric, cylindrical. ***Developing turbinate eyes in last instar male larva*** (Fig. 133a) with facets equally developed on middle and periphery areas. ***Labrum*** (Fig. 134a) short and widest at base; long setae on dorsal surface simple (not branched), few (2–4 on each side) and irregularly situated. ***Right mandible*** (Fig. 134c, e) short, with incisor not elongated; incisor terminating with 4 denticles, most distal denticle shorter than others; other three denticles subequal; mola with seta on proximal corner. ***Left mandible*** (Fig. 134b, d) short, with incisor not elongated; incisor terminating with four denticles, most distal denticle shorter than others; other three denticles subequal; mola with seta on proximal corner. ***Hypopharynx*** (Fig. 134g) with bunch of long, straight, stout, setae-like spines situated on common projection. ***Maxilla*** (Fig. 134f) short and wide; maxillary palp 2-segmented, as long as galea-lacinia. ***Labium*** (Fig. 134h–j) Paraglossae widest at base, lateral side without concavity; three apical setal rows straight (not bent at apex of paraglossa). Glossa longer than half of paraglossa, with finger-like (distal) portion much longer than triangular (proximal) portion. Ventral side of glossa with several irregularly arranged setae both on triangular and finger-like portions. Labial palp with long, narrow, arched distomedian projection on segment II; segment III with median margin shorter than lateral margin.

***Thorax*. *Sterna*** without protuberances. ***Terga*.** Without long setae on midline. Metanotum without hind protoptera or their vestiges. ***Legs*** (Figs 135a–c, 136a–c). Fore femur widened in proximal part; hind tibia shorter than others. ***Femur*.** Outer side of each femur with single regular row of long, slender, flattened, parallel-sided setae with blunt apices. Cuticle of anterior surface of each femur with serrate areas corresponding with brownish pigmentation (see above). ***Tibia*.** Patella-tibial suture present on all legs, terminated proximad of inner margin of tibia. Tibia-tarsal condylus turned to anterior side. Anterior side of each tibia with regular row of long, slender, flattened, parallel-sided setae with blunt apices, similar to that on femur, but shorter. ***Tarsus*.** Anterior side of each tarsus with regular row of similar, but shorter setae. Posterior side of each tarsus with regular row of few small, stout, pointed setae, most distal of which not longer or slightly longer than others. ***Claw*** with row of 9–11 subequal denticles and with long, arched seta on posterior side.

***Abdomen*. *Terga*** (Figs 133c, 135d). Abdominal terga without long setae on midline. All abdominal terga smooth, without median or submedian elevations or protuberances. Surface of abdominal terga rough, with numerous short sensilla, without long setae; posterior margins of abdominal terga II–X with very small, blunt, dark brown denticles. Posterior margins of abdominal sterna without denticles. ***Tergalii*** (Fig. 136d–k) of abdominal segment I absent; tergalii II–VII subequal, long and narrow. Each tergalius with anal rib longer than costal rib; ribs with very small, irregularly situated denticles on dorsal side. ***Paraproct*** (Fig. 133g) with small, equal denticles on posterior margin, without denticles on median margin, without posterior prolongation. ***Caudalii*** (Fig. 133d). Cerci without swimming setae. Paracercus short, consisting of ~ 15 segments.

***Pose of subimaginal gonostyli under larval cuticle*** (Fig. 137e). In mature larva ready to molt to subimago, subimaginal gonostyli packed under larval cuticle in “*Crassolus*-type” pose (Kluge et al. 2020; Kluge 2021): gonostylus shortened and bent in articulation of 2^nd^ and 3^rd^ segments; 2^nd^ segment directed caudally-medially and not bent; 3^rd^ segment directed medially-cranially and terminated mediad of 2^nd^ segment.

**Subimago. *Cuticular coloration*** (Fig. 137d). Mesonotum very pale brownish with medioparapsidal sutures colorless, some sutures darker brownish. Thoracic pleura with brown and nearly colorless areas. Sterna nearly colorless. Wing membrane colorless, microtrichia brown. Legs nearly colorless; outer side of femur and base of tibia tinged with pale brownish. Abdomen very pale brownish, cerci colorless.

***Hypodermal coloration*.** As in imago.

***Texture*.** On all legs of female, terminal tarsomere covered with pointed microlepides only; other tarsomeres covered partly with pointed, partly with blunt microlepides (as in Fig. 143c, d).

**Imago. *Imago, male*** (partly molted from subimago). Head ochre. Antennae ochre. Turbinate eyes dull-red, wide, with faceted surfaces round, contiguous. Thorax ochre, equally pale dorsally, laterally, and ventrally. Fore wing with membrane colorless, veins pale ochre, base of RA and costal brace colored with brownish. Pterostigma with 2–3 oblique, incomplete cross veins (Fig. 137a). Legs ochre; femur of each leg with wide brownish band in distal part and with compact, oval, brown macula in proximal 1/2 (Fig. 137c). On middle and hind legs, tarsus with 2 apical spines, on 1^st^+2^nd^ and 3^rd^ tarsomeres. Abdomen mostly whitish, terga VII–VIII pale ochre; each tergum I–VIII with pair of small, dark brown sublateral maculae on posterior margin (Fig. 137b). Cerci pale ochre.

Shape of gonostyli unknown.

***Imago, female*.** Unknown.

**Figure 138.**
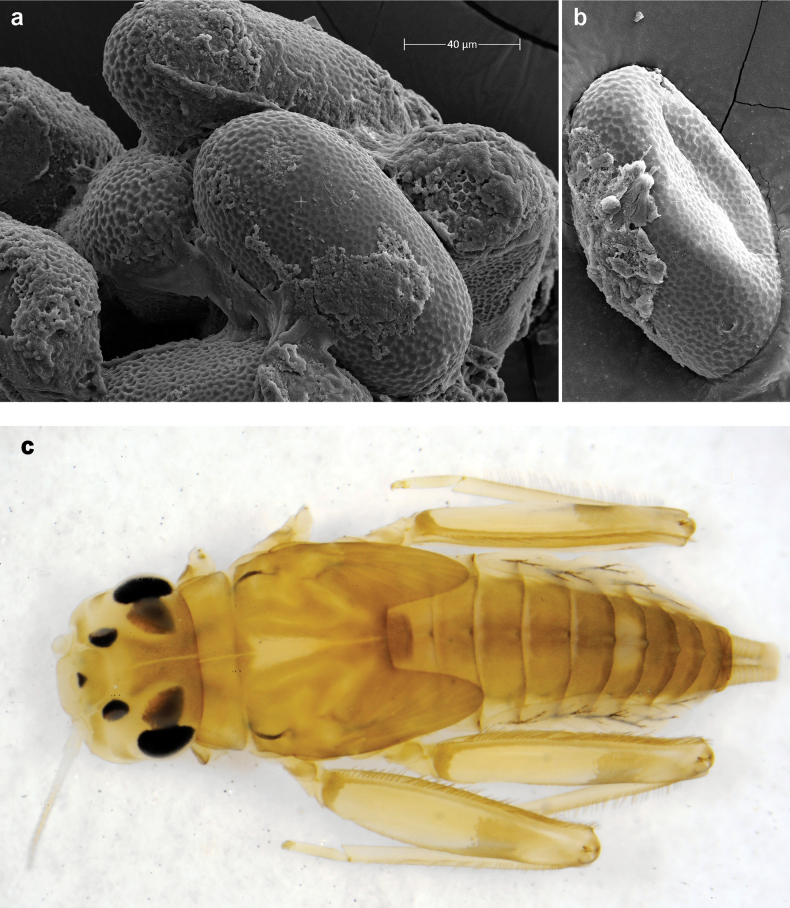
**a, b**Papuanatula (Papuafiliola) stenophylla sp. nov., eggs (extracted from mature larva) **c**Papuanatula (Papuafiliola) tuberculata sp. nov., larva.

**Egg** (Fig. 138a, b). Elongate oval. Chorion entirely and evenly covered with ridges forming a net-like relief.

#### Dimension.

Fore wing length of male (and approximate body length) 3.5 mm.

#### Comparison.

Larva of Papuanatula (Papuafiliola) stenophylla sp. nov. differs from P. (Papuafiliola) tuberculata sp. nov. by presence of brown hypodermal maculae on femora and abdomen, absence of median protuberances on abdominal terga and narrower tergalii. Imago of P. (Papuafiliola) stenophylla sp. nov. differs from P. (Papuafiliola) tuberculata sp. nov. by presence of brown hypodermal maculae on proximal part of middle and hind femora.

#### Distribution.

New Guinea (Fig. 148).

### ﻿﻿ Papuanatula (Papuafiliola) tuberculata sp. nov.

Taxon classificationAnimaliaEphemeropteraBaetidae

EA88C842-6274-5D86-8225-9B48D59E7A64

https://zoobank.org/2315FD83-BEAF-41B3-9164-42A700945CC8

Figs 138 , 139 , 140 , 141 , 142 , 143 , 144 , 145

#### Etymology.

The species name *tuberculata* refers to the unpaired tubercles on abdominal terga of the larva (Fig. 138c).

#### Material examined.

***Holotype*.** L-S-I♀ {specimen number [V] (2) 2012}; Indonesia • Papua, Baliem valley, Wamena, river Elagaima; 16.viii.2012; coll. N. Kluge & L. Sheyko; SPbU. ***Paratypes*.** Same locality and collectors; 15–19.viii.2012: 2 larvae; SPbU.

#### Diagnosis.

**Larva.** The following combination of characters distinguishes *P.tuberculata* sp. nov. from the other species of Papuafiliola subgen. nov.: absence of brown hypodermal maculae on femora and abdomen; presence of median protuberances on abdominal terga; tergalii rather wide.

#### Description.

**Figure 139.**
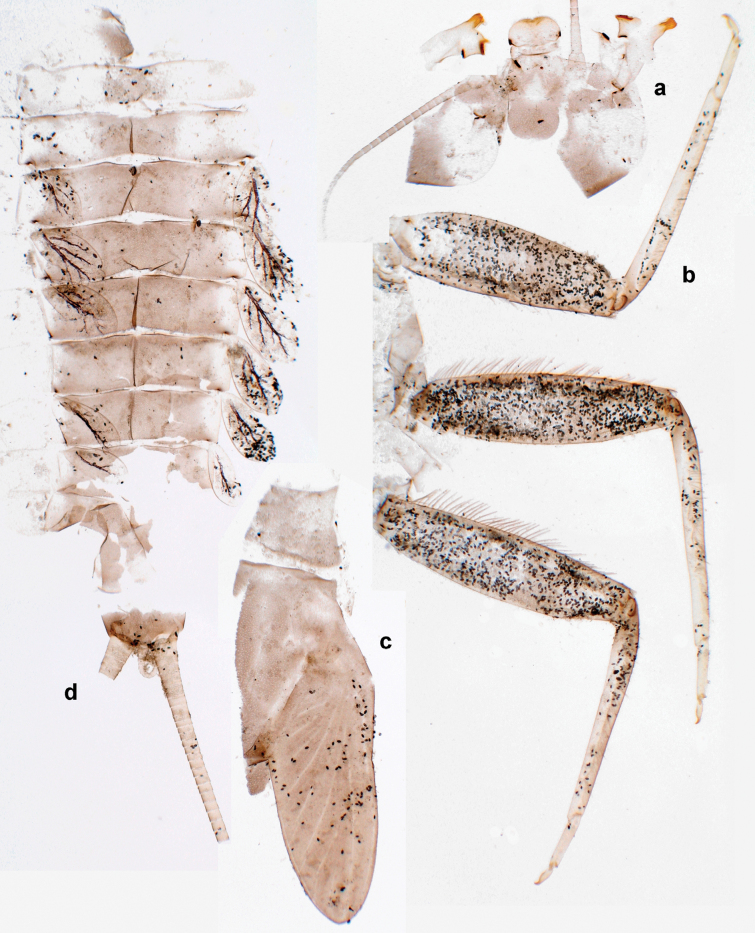
Papuanatula (Papuafiliola) tuberculata sp. nov., larval exuviae (holotype, in one magnification): **a** head **b** fore, middle, and hind legs (densely covered with diatom algae) **c** half of pronotum and mesonotum **d** abdomen.

**Figure 140.**
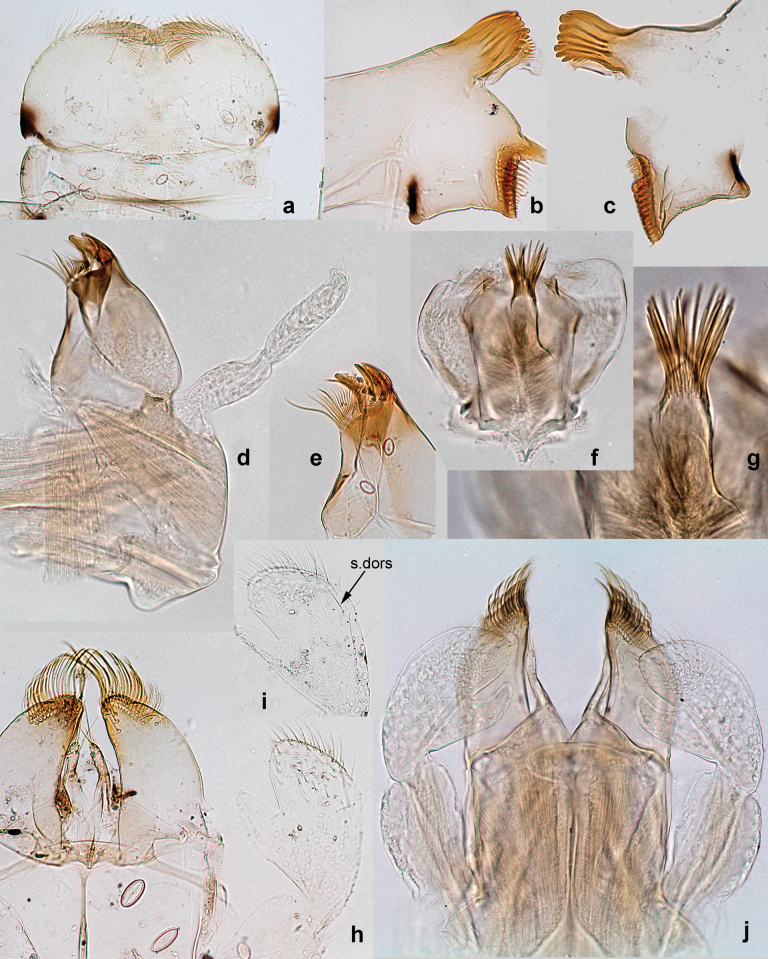
Papuanatula (Papuafiliola) tuberculata sp. nov., larva (**a–c, e, h, i** holotype): **a** labrum **b**, **c** left and right mandibles **d, e** maxillae **f, g** hypopharynx **h–j** labium (**i** focus on dorsal side).

**Figure 141.**
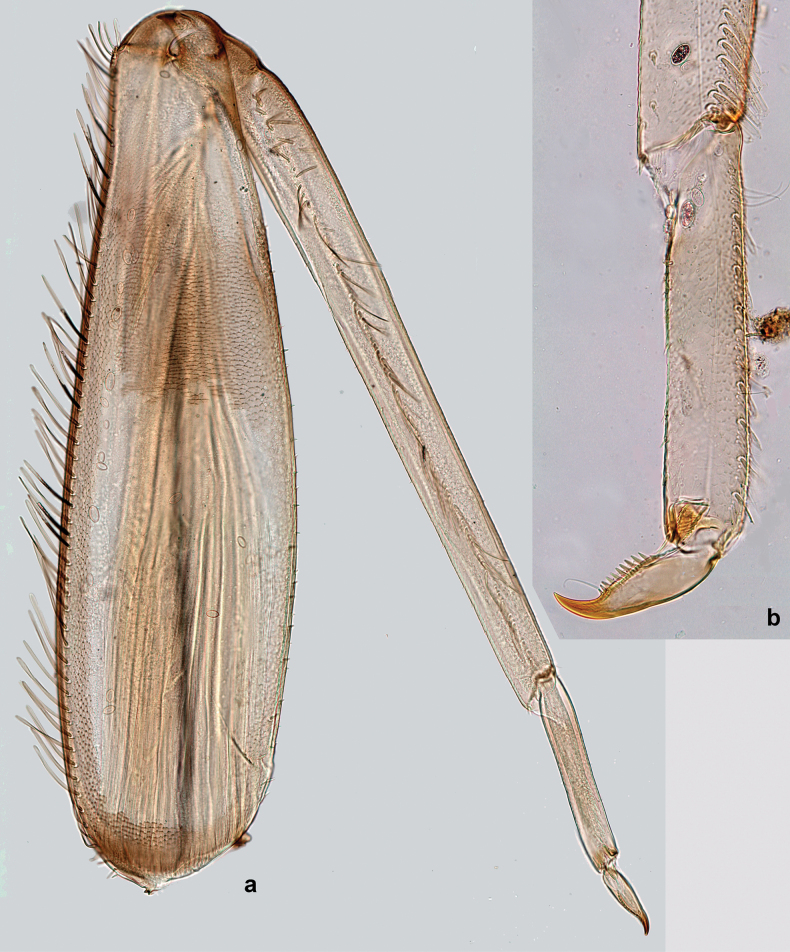
Papuanatula (Papuafiliola) tuberculata sp. nov., larva: **a** leg **b** tarsus of fore leg (holotype).

**Figure 142.**
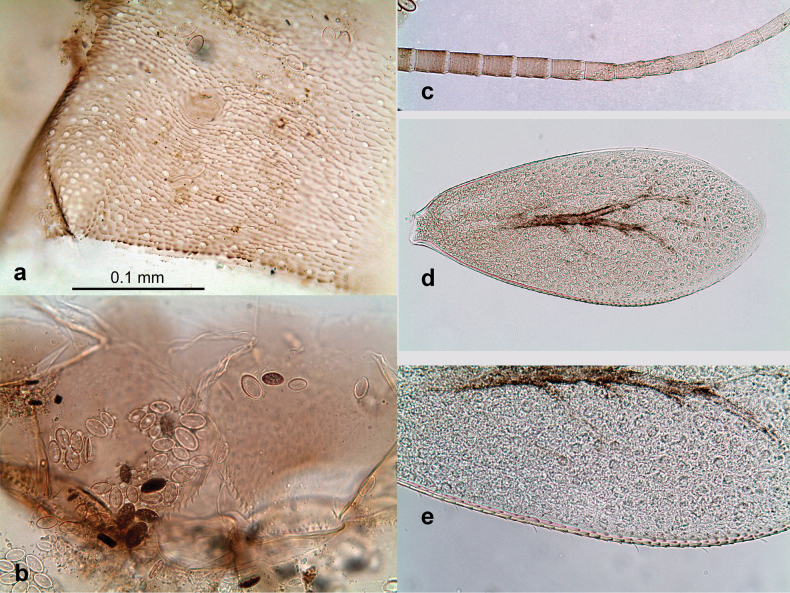
Papuanatula (Papuafiliola) tuberculata sp. nov., larva: **a** tergum III **b** paraprocts **c** antenna **d** tergalius IV **e** its enlarged anal rib (**a–c** holotype).

**Larva** (Figs 138–142). ***Cuticular coloration*.** Head, pronotum and mesonotum mostly pale brownish, with some paler areas. Fore protopteron pale brownish, with narrow paler lines along concave veins (Fig. 139a, c). Metanotum darker brownish medially, colorless laterally. Thoracic pleura pale brownish, sterna colorless. Cuticle of legs mostly colorless, with brownish outer margin and two brownish transverse bands on anterior surface: one close to base and another in apical part; cuticle of these brownish areas serrate (Figs 139b, 141a, b). Abdominal terga mostly pale brownish, with lateral areas paler or colorless; tergum VI more or less paler than others; sterna colorless (Figs 138, 139d). Cerci uniformly pale brownish.

***Hypodermal coloration*.** Not expressed (at least in penultimate larval instar). Tissues surrounding tracheae of tergalii (main trachea and its branches) with brown pigmentation (Fig. 142d, e).

***Head*. *Antenna*** (Fig. 142c) with each flagellomere symmetric, cylindrical. ***Developing turbinate eyes in last instar male larva*.** Unknown. ***Labrum*** (Fig. 140a) short and widest at base; long setae on dorsal surface simple (not branched), constitute two pairs: submedian pair and one more pair laterad of them. ***Right mandible*** (Fig. 140c) short, with incisor not elongated; incisor terminating with four denticles, among which most distal denticle shorter than others; other three denticles subequal; mola with seta on proximal corner. ***Left mandible*** (Fig. 140b) short, with incisor not elongated; incisor terminating with four denticles, among which most distal denticle shorter than others; other three denticles subequal; mola with seta on proximal corner. ***Hypopharynx*** (Fig. 140f, g) with bunch of long, straight, stout, setae-like spines situated on common projection. ***Maxilla*** (Fig. 140d, e) short and wide; maxillary palp 2-segmented, as long as galea-lacinia. ***Labium*** (Fig. 140h–j). Paraglossae widest at base, lateral side with slight concavity near base; three apical setal rows parallel to apical-lateral margin only (not bent parallel to median margin). Glossa longer than half of paraglossa, with finger-like (distal) portion much longer than triangular (proximal) portion. Ventral side of glossa with several irregularly arranged setae both on triangular and finger-like portions. Labial palp with long, narrow, arched distomedian projection on segment II; segment III with median margin shorter than lateral margin.

***Thorax*. *Sterna*** without protuberances. ***Terga*.** Without long setae on midline; without protuberances. Metanotum without hind protoptera or their vestiges. ***Legs*** (Figs 139b, 141a, b). Fore femur widened in proximal part; hind tibia shorter than others. ***Femur*.** Outer side of each femur with single regular row of long, slender, flattened, parallel-sided setae with blunt apices. Cuticle of anterior surface of each femur with serrate areas corresponding with brownish pigmentation (see above). ***Tibia*.** Patella-tibial suture present on all legs, terminated proximad of inner margin of tibia. Tibia-tarsal condylus turned to anterior side. Anterior side of each tibia with regular row of long, slender, flattened, parallel-sided setae with blunt apices, similar to that on femur, but shorter. ***Tarsus*.** Anterior side of each tarsus with regular row of similar, but shorter setae. Posterior side of each tarsus with regular row of few small, stout, pointed setae, most distal of which not longer or slightly longer than others. ***Claw*** with row of 8–10 subequal denticles and with long, arched seta on posterior side.

***Abdomen*. *Terga*** (Figs 138, 139d, 142a) without long setae on midline (e.g., in contrast to *P.obscura* sp. nov.). Each abdominal tergum I–IX with slightly expressed median ridge elevating toward posterior margin. Surface of abdominal terga serrate, with numerous short sensilla; posterior margins of abdominal terga II–X with very small, blunt, dark brown denticles. Abdominal sterna without serrations and denticles. ***Tergalii*** (Fig. 142d, e) of abdominal segment I absent; tergalii II–VII subequal, oval. Each tergalius with anal rib longer than costal rib; ribs with very small, irregularly situated denticles on dorsal side. ***Paraproct*** (Fig. 142b) with many small, equal denticles on median and posterior margins, without posterior prolongation. ***Caudalii*** (Fig. 139d). Cerci without swimming setae. Paracercus short, consisting of ~12 segments.

***Pose of subimaginal gonostyli under larval cuticle*.** Unknown.

**Figure 143.**
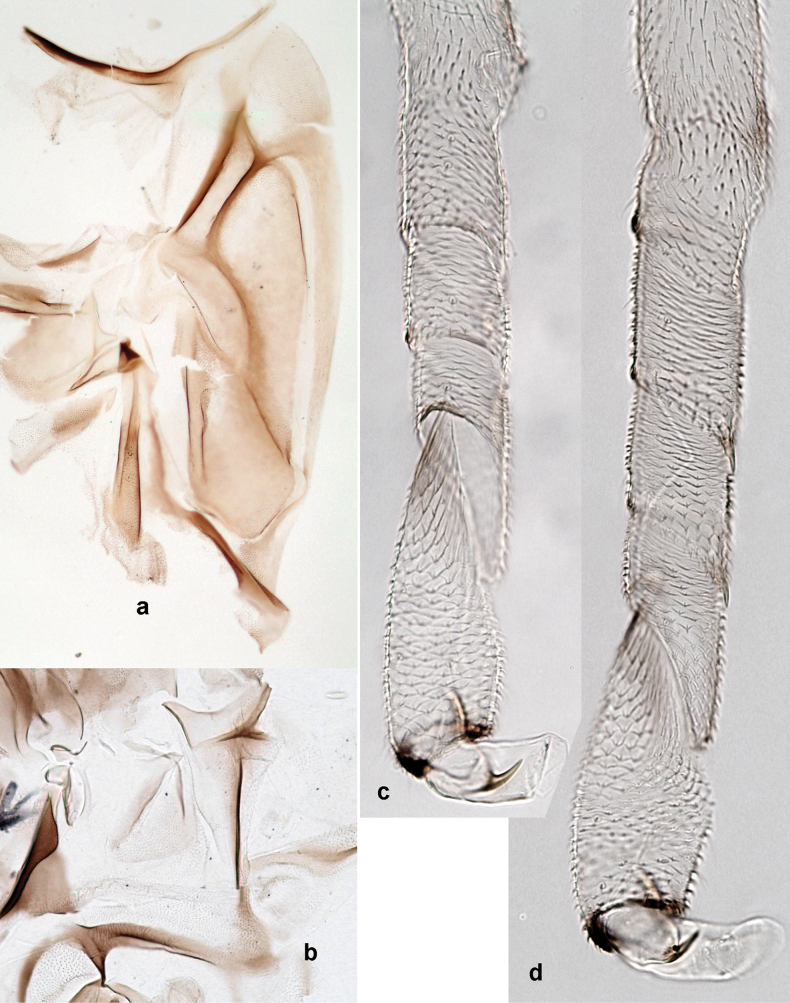
Papuanatula (Papuafiliola) tuberculata sp. nov., subimaginal exuviae (holotype): **a** half of mesonotum **b** mesopleuron **c** hind tarsus **d** fore tarsus.

**Subimago. *Cuticular coloration*.** Pronotum partly brownish. Mesonotum pale brown with medioparapsidal suture colorless, other sutures darker brown (Fig. 143a). Meso- and metathoracic pleura and sterna with colorless, pale brownish and dark brown areas (Fig. 143b). Cuticle of wings colorless, with microtrichiae brownish. Legs nearly colorless, with pale brown bordering on femur and base of tibia (as in Fig. 36d). Abdomen very pale brownish. Cerci colorless with setae brownish.

***Hypodermal coloration*.** As in imago.

***Texture*.** On all legs of female, terminal tarsomere covered with pointed microlepides only; other tarsomeres covered partly with pointed, partly with blunt microlepides (Fig. 143c, d).

**Imago. *Imago, male*.** Unknown.

**Figure 144.**
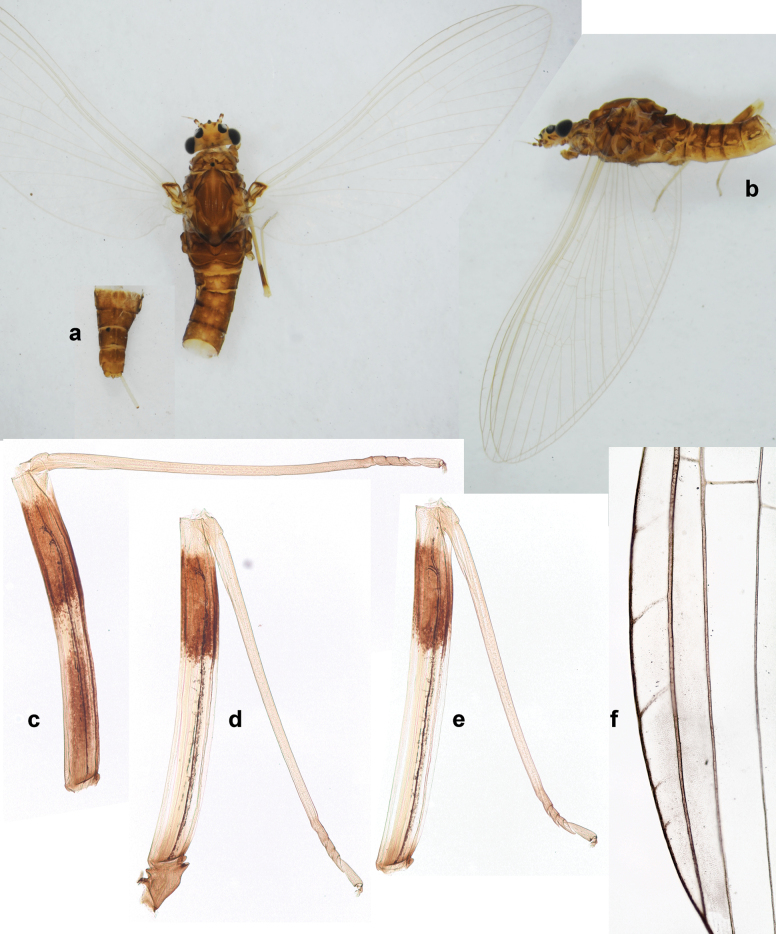
Papuanatula (Papuafiliola) tuberculata sp. nov., female imago (holotype): **a** dorsal view **b** lateral view **c–e** fore, middle and hind legs **f** pterostigma.

***Imago, female*** (Fig. 144a–f). Head ochre with brown. Antennae ochre, distal parts of scapus and pedicellum brown. Thorax brown with ochre, equally dark dorsally, laterally, and ventrally. Fore wing with membrane mostly colorless, veins pale ochre; base of wing, including costal brace contrastingly pigmented with brown and ochre. Pterostigma with three or four oblique, incomplete cross veins (Fig. 144f). Legs ochre; femur of each leg with wide reddish-brown band in distal part; femur of fore leg, besides this, with longitudinal reddish-brown macula occupying most part of proximal 1/2 (Fig. 144c–e). On each leg, tarsus with two apical spines: on 2^nd^ and 3^rd^ tarsomeres on fore leg, on 1^st^+2^nd^ and 3^rd^ tarsomeres on middle and hind legs. Each abdominal tergum I–IX brown with paler ochre areas; tergum X brownish ochre; sterna ochre (Fig. 144a, b). Cerci pale ochre.

**Figure 145.**
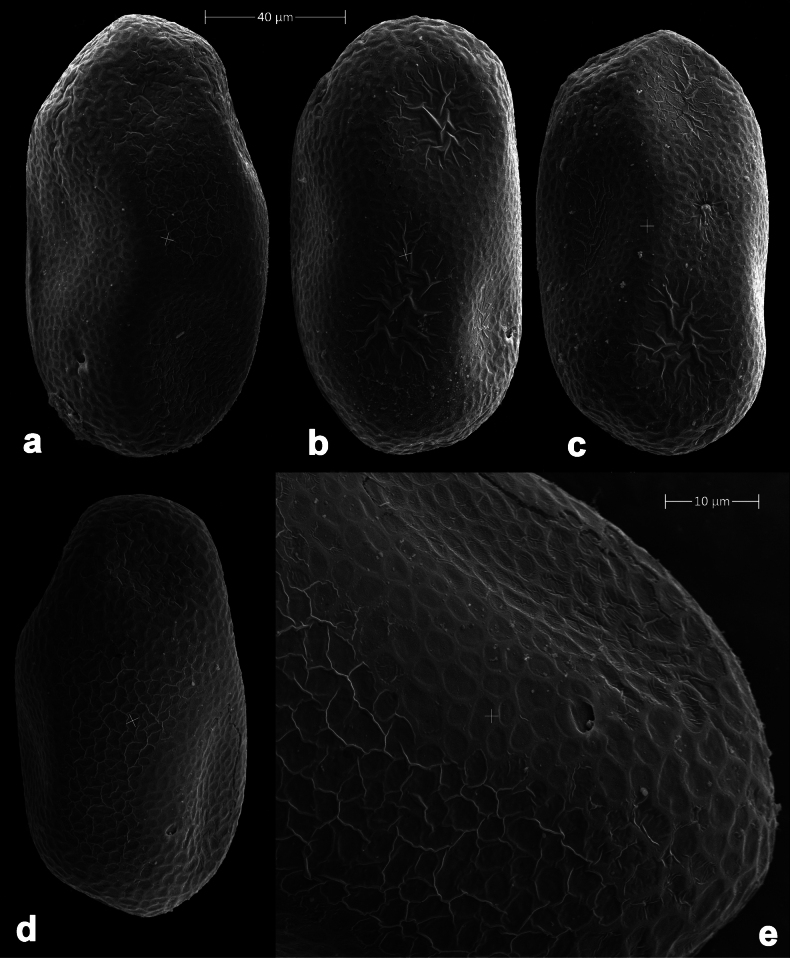
**a–e**Papuanatula (Papuafiliola) tuberculata sp. nov., eggs (extracted from imago).

**Egg** (Fig. 145a–e). Elongate oval. Chorion entirely and evenly covered with ridges forming a net-like relief.

**Figure 146.**
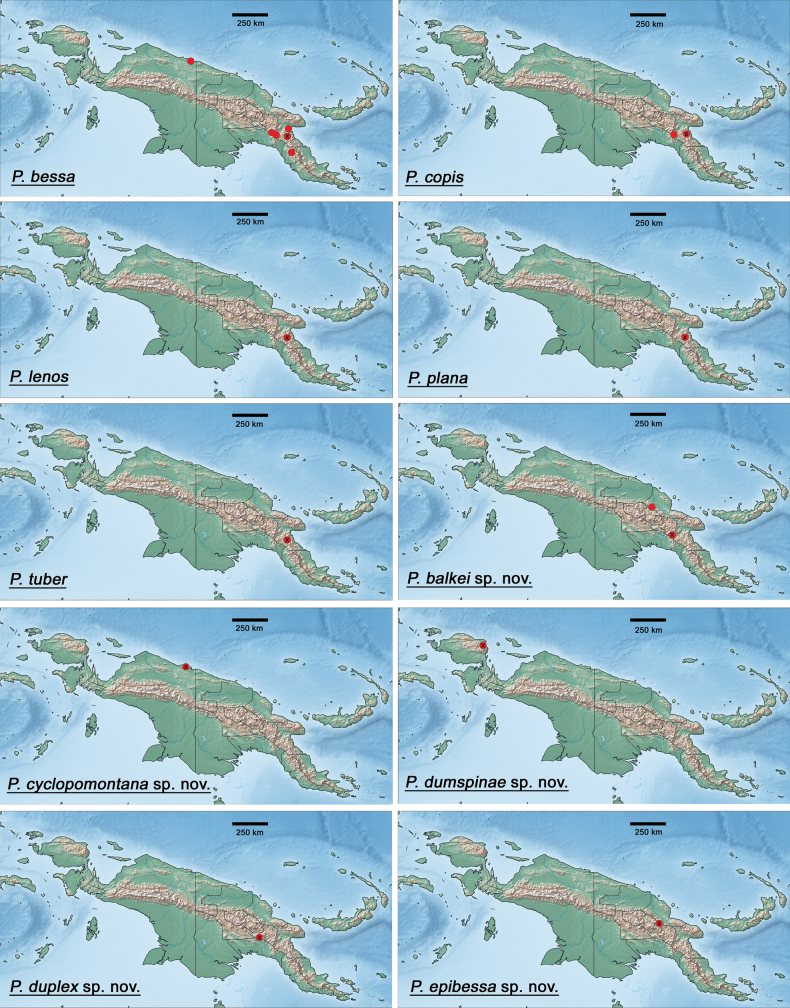
Distribution of *Papuanatula* species (x: type locality).

**Figure 147.**
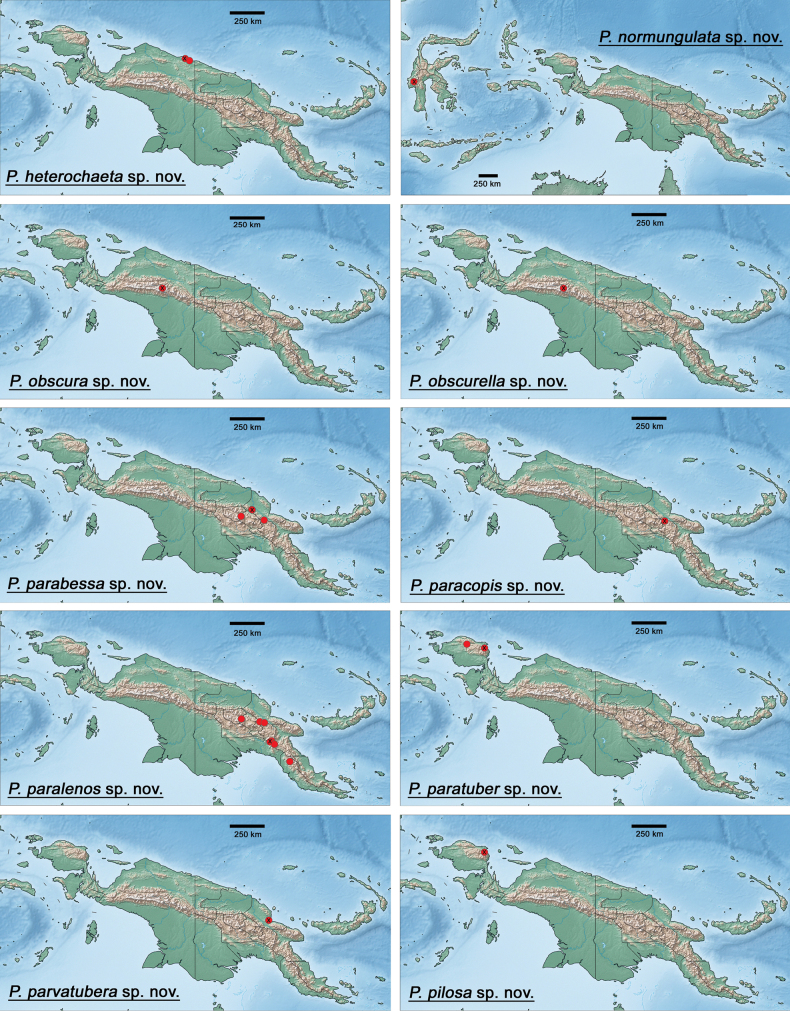
Distribution of *Papuanatula* species (x: type locality).

#### Dimension.

Fore wing length of female (and approximate body length) 50 mm.

#### Comparison.

Larva of Papuanatula (Papuafiliola) tuberculata sp. nov. differs from P. (Papuafiliola) stenophylla sp. nov. by absence of brown hypodermal maculae on femora and abdomen, presence of median protuberances on abdominal terga and wider tergalii. Imago of P. (Papuafiliola) tuberculata sp. nov. differs from P. (Papuafiliola) stenophylla sp. nov. by absence of brown hypodermal maculae on proximal part of middle and hind femora.

#### Distribution.

**Figure 148.**
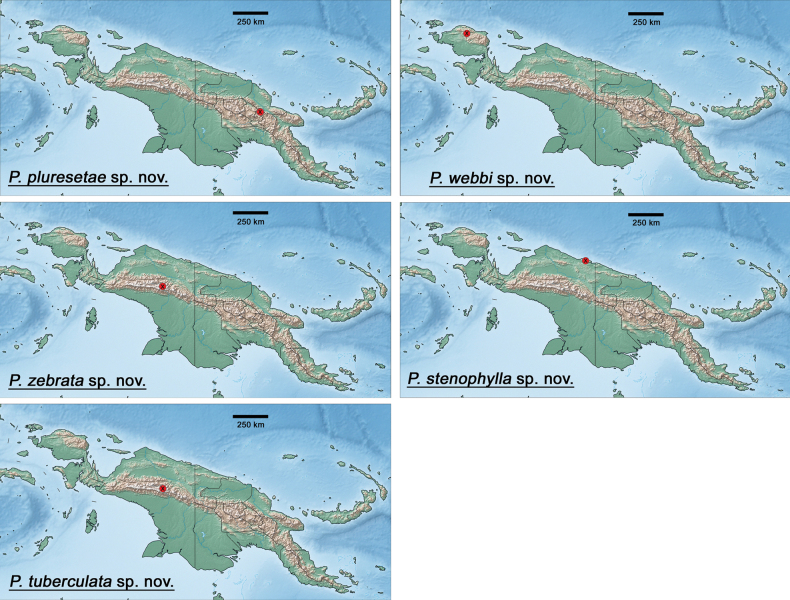
Distribution of *Papuanatula* species (x: type locality).

New Guinea (Fig. 148).

### ﻿﻿Key to the species of *Papuanatula* (larvae)

(for definition of species groups see discussion)

**Table:** 

1	Antennal flagellum distally without brown dots; labial palp segment II with distinct distomedial projection; incisors of mandibles not blade-like; labrum dorsally with few simple setae (Papuafiliola subgen. nov.)	**2**
–	Antennal flagellum distally with brown dots; labial palp segment II without distomedial projection; incisors of mandibles blade-like; labrum dorsally with submarginal arc of feathered setae (*Papuanatula* s. str.)	**3**
2(1)	Abdominal terga without protuberances; femur and abdomen with brown hypodermal maculae	***P.stenophylla* sp. nov.**
–	Abdominal terga with median protuberances; femur and abdomen without brown hypodermal maculae	***P.tuberculata* sp. nov.**
3(1)	Head, thorax, and abdomen dorsally with irregular row of long, fine, simple setae on midline (group *P.bessa*)	**4**
–	Head, thorax, and abdomen dorsally without row of setae on midline	**11**
4(3)	Metanotum and abdominal terga I–III or I–V with medioposterior broad, paired humps (Fig. 5a–f), may be poorly developed (Fig. 53a)	**5**
–	Metanotum and abdominal terga without paired humps	**7**
5(4)	Metanotum and abdominal terga I–V with medioposterior, paired humps; femur with wedge-shaped blank in proximal area; claw with one posterior seta	**6**
–	Metanotum and abdominal terga I–III with medioposterior, paired humps poorly developed; femur without wedge shaped blank; claw with 1–3 posterior setae	***P.epibessa* sp. nov.**
6(5)	Abdominal terga II–IV dark brown with brighter, oblong marking	***P.bessa***
–	Abdominal terga II–VI with paired, semicircular, dark brown markings	***P.parabessa* sp. nov.**
7(4)	Paracercus vestigial (max. 2 segments); claw with 3 or 4 posterior setae	***P.pluresetae* sp. nov.**
–	Paracercus with 7–9 segments; claw with 1 posterior seta	**8**
8(7)	Abdominal terga II–IX with long, triangular, pointed denticles on posterior margins	**9**
–	Abdominal terga II–IX with triangular, apically rounded denticles on posterior margins	**10**
9(8)	Abdominal terga V, VI, and X much brighter than other terga; apical setal rows of paraglossae curved; triangular, pointed denticles on posterior margins of abdominal terga long and narrow	***P.dumspinae* sp. nov.**
–	Abdomen with rather uniform color; apical setal rows of paraglossae straight; triangular, pointed denticles on posterior margins of abdominal terga of different length	***P.cyclopomontana* sp. nov.**
10(8)	Femur with clearly outlined wedge-shaped blank; abdomen dorsally brownish, terga I, V, VI, and X brighter; tergalii with poorly visible tracheation; small scales on abdominal terga roundish with radial striation	***P.obscura* sp. nov.**
–	Femur with wedge-shaped blank, overlaid with scattered brownish color; abdomen dorsally dark brown, terga V, VI, and X brighter; tergalii with clearly visible, pigmented tracheation; small scales on abdominal terga elongate, slightly trapezoid, striated	***P.balkei* sp. nov.**
11(3)	Abdominal terga at least partly with posteromedial protuberance	**12**
–	Abdominal terga without protuberance	**20**
12(11)	Single, posteromedial protuberance at least on some (IV–VIII) abdominal terga (group *P.copis*)	**13**
–	Paired, posteromedial protuberances on abdominal terga I–IX	***P.duplex* sp. nov.**
13(12)	Sulawesi; patella-tibial suture reduced; posterior setae on claw absent; outer margin of femur and tibia with stripe of dense setae	***P.normungulata* sp. nov.**
–	New Guinea; patella-tibial suture present; posterior setae on claw present; femur and tibia with regular row of long setae	**14**
14(13)	Paracercus with 7 or 8 segments	**15**
–	Paracercus vestigial	**16**
15(14)	Thoracic terga without protuberance; abdominal terga IV–VIII with small, triangular, posteromedial protuberance, oriented posteriorly (may be vestigial on II, III, and IX)	***P.parvatubera* sp. nov.**
–	Pronotum with small, paired, triangular protuberances; fore protoptera with pair of minute protuberances at posteromedial margin; metanotum with small, rounded protuberance; abdominal terga I–IX with apically rounded protuberance, oriented dorsoposteriorly	***P.webbi* sp. nov.**
16(14)	Pronotum posteromedially with pair of protuberances	**17**
–	Pronotum without protuberance (immature larva may have minute, single, pointed protuberance at posteromedial margin)	**18**
17(16)	Abdominal terga I–VIII posteromedially with short, stout protuberances, oriented dorsally; length of mature larva 2.7–3.4 mm; tergalii narrow elongate, not tracheated or poorly tracheated, margins smooth without setae; paraproct without extension, marginally without spines	***P.tuber***
–	Abdominal terga I–IX posteromedially with medium, pointed protuberances, oriented dorsoposteriorly; length of mature larva ~ 4.5 mm; tergalii skewed ovoid, tracheation well developed, margins smooth with short, simple setae; paraproct with extension and with marginal spines	***P.pilosa* sp. nov.**
18(16)	Abdominal terga I–VIII posteromedially with medium, pointed protuberances; pro-, meso-, and metanotum without protuberance	***P.paratuber* sp. nov.**
–	Abdominal terga I–VIII posteromedially with long, pointed protuberances; metanotum with conspicuous posteromedial protuberance; at least immature larva with small, acute, posteromedial protuberance on pro- and mesonotum	**19**
19(18)	Metanotum and abdominal terga I–VIII with posteromedial, long, finely pointed protuberances, oriented dorsoposteriorly; subtriangular process usually undeveloped; labial palp segment III globular	***P.copis***
–	Metanotum and abdominal terga I–VIII with posteromedial, long, pointed protuberances, hook-like, bent posteriorly; subtriangular process developed; labial palp segment III oblong	***P.paracopis* sp. nov.**
20(11)	Femur anteriorly without marking in proximal 1/2 (group *P.plana*)	**21**
–	Femur anteriorly with conspicuous brown to blackish, hypodermal marking in proximal 1/2 (group *P.lenos*)	**22**
21(20)	Abdomen with hypodermal, brown band on posterior margin of terga I–IX	***P.obscurella* sp. nov.**
–	Only abdominal tergum IV with dark brown, medioposterior mark	***P.plana***
22(20)	New Britain; femur anteriorly with large, brown, triangular macula	***P.vaisisi***
–	New Guinea; femur anteriorly with macula other than triangular	**23**
23(22)	Femur anteriorly with dark brown, shoe-shaped macula inside large, proximal blank; hypopharynx apically with pair of bunches of seta-like spines	***P.heterochaeta* sp. nov.**
–	Femur anteriorly with macula other than shoe-shaped in proximal 1/2; hypopharynx with usual, unpaired bunch of seta-like spines	**24**
24(23)	Femur anteriorly with brown, hypodermal streak in proximal 1/2; posterior margin of abdominal terga with heterogenous, sharply pointed denticles	***P.zebrata* sp. nov.**
–	Femur anteriorly with red-brown to dark brown or blackish, oblong to drop-shaped hypodermal marking in mediodistal area; posterior margin of abdominal terga with regular, triangular denticles	**25**
25(24)	Thorax dorsally without distinct markings; femur with row of short, spine-like setae on inner margin; tergalii margin smooth, with short, fine, simple setae	***P.lenos***
–	Pronotum with large, dark brown marking medially on anterior margin, narrow dark brown band along posterior margin of pronotum and anterior margin of mesonotum; femur with many medium, spine-like setae along inner margin; tergalii margin with minute serration and short, fine, simple setae	***P.paralenos* sp. nov.**

## ﻿﻿Discussion

### ﻿﻿Relationships and affinities of *Papuanatula*

Lugo-Ortiz and McCafferty (1999) discussed possible phylogenetic relationships of *Papuanatula* without providing any final determination. They highlighted similarities to genera like *Jubabaetis* Müller-Liebenau, *Liebebiella* Waltz & McCafferty, *Platybaetis* Müller-Liebenau, and others that may be adaptations to fast-flowing streams. Today, these genera are part of Acentrellini (Kluge and Novikova 2011). Lugo-Ortiz and McCafferty (1999) also mentioned the femoral patch, present in these genera, but absent in *Papuanatula*. A possible relationship to *Labiobaetis* was also discussed due to the sclerotized penial bridge between gonostyli of the male genitalia, but equally refuted mainly because of other morphological differences in the gonostyli (Lugo-Ortiz and McCafferty 1999). Indeed, the larvae of *Papuanatula* show many similarities to larvae of the tribe Acentrellini Kluge & Novikova, 2011: femur with row of long, dense setae on outer margin; presence of a regular row of long, dense setae on tibia and tarsus; claws twisted, directed perpendicular to general flatness of the leg; cerci long, primary swimming setae strongly reduced or vestigial; paracercus strongly reduced or vestigial. On the other hand, *Papuanatula* is distinguished from Acentrellini by other important larval characters: glossae of labium much shorter than paraglossae; labrum dorsally with submarginal arc of long, feathered setae (only in *Papuanatula* s. str., few simple setae in Papuafiliola subgen. nov.); femoral patch absent on all legs; paraproct usually with proximal expansion; sterno-styliger muscle absent (present in all species of Acentrellini). Furthermore, in imagos, the shape of the anteronotal protuberance of mesonotum typical for Acentrellini is not found in *Papuanatula* (Kluge and Novikova 2011). Most of the similarities between *Papuanatula* and Acentrellini larvae are probably convergences and adaptations to fast-flowing water. Among them, the presence of protuberances on the thoracic sterna close to openings of the sternal apodemes; besides being present in many species of *Papuanatula*, such protuberances are present in other rheophilic taxa, e.g., some species of *Acentrella* (Kluge and Novikova 2011: table 2), *Baetiella* (Novikova and Kluge 1987: fig. 3.4–6) and Baetisgr.lutheri (Müller-Liebenau 1974: fig. 4; Eiseler 2005: figs 76, 77; Yanai et al. 2018: fig. 9). Possibly, these protuberances could help to avoid accidental drifting in stronger currents, similar to other structures of *Papuanatula* larvae like the protuberances on tergites or the long rows of setae on the legs.

The genus *Papuanatula* is placed in the tribe Labiobaetini Kluge & Novikova, 2016, which is characterized by the following combination of characters: in male genitalia, the sterno-styliger muscle is completely absent; in mature male larva, subimaginal gonostyli developing under larval cuticle are folded in the “*Labiobaetis*-type” pose; in male imaginal gonostyli, segment II has no significant widenings, segment III (terminal segment) is short and not much narrower than segment II; in larval labium, paraglossae are more or less strengthened and widened (Kluge and Novikova 2016). Each of these characters is also found in other taxa of Baetidae, but not in this combination (Kluge and Novikova 2014). However, in the subgenus Papuafiliola subgen. nov., the pose of the subimaginal gonostyli developing under cuticle of mature male larvae is of the “*Crassolus*-type” and not “*Labiobaetis*-type” (contrary to *Papuanatula* s. str.).

The genus *Philibaetis* Kaltenbach, Garces & Gattolliat, 2021 has a similar arrangement of long, robust setae mediolaterally on both mandibles as in *Papuanatula*. It further shares the blade-like elongated incisors of both mandibles and the pose of the subimaginal gonostyli developing under larval cuticle of mature male larvae with *Papuanatula* s. str., and the posterior setae on the claws of *Papuanatula* (Kaltenbach et al. 2021b). *Philibaetis* was preliminarily assigned to Labiobaetini, awaiting the description of an imago, which is still unknown.

### ﻿﻿Morphological species groups of *Papuanatula* s. str.

The morphological comparison of the larvae of all species of *Papuanatula* s. str. reveals several well-defined morphological species groups. Some of them could be natural clades, and their characters are listed below.

#### ﻿﻿*Papuanatulabessa* species group

Body dorsally with irregular row of long, fine, soft setae on midline; abdominal terga partially with paired, medioposterior humps or elevations. The following species are part of this group: *P.bessa*, *P.balkei* sp. nov., *P.cyclopomontana* sp. nov., *P.dumspinae* sp. nov., *P.epibessa* sp. nov., *P.obscura* sp. nov., *P.parabessa* sp. nov., and *P.pluresetae* sp. nov. Especially the first character (body dorsally with irregular row of setae on midline) should be regarded as an apomorphy, postulating that the *bessa* group is a clade.

#### ﻿﻿*Papuanatulalenos* species group

Body dorsally without row of setae on midline; body dorsally without protuberances; femur with hypodermal macula; thoracic sterna without protuberances; paraproct without posterior prolongation. The following species are part of this group: *P.lenos*, *P.heterochaeta* sp. nov., *P.paralenos* sp. nov., *P.zebrata* sp. nov., and probably *P.vaisisi*. The group is clearly defined; however, it remains unclear if it is a natural clade.

#### ﻿﻿*Papuanatulacopis* species group

Body dorsally without row of setae on midline; abdomen and sometimes also thorax dorsally with unpaired, conspicuous protuberances. The following species belong to this group: *P.copis*, *P.tuber*, *P.normungulata* sp. nov. (Sulawesi); *P.paracopis* sp. nov., *P.paratuber* sp. nov., *P.parvatubera* sp. nov., *P.pilosa* sp. nov., and *P.webbi* sp. nov. Probably, the *copis* group is not a natural clade, but is retained here for practical reasons.

#### ﻿﻿*Papuanatulaplana* species group

Body dorsally without row of setae on midline; body dorsally without protuberances; legs without hypodermal pigmentation; posterior margin of abdominal terga with pointed denticles. The following species belong to this group: *P.plana* and *P.obscurella* sp. nov. Based on morphology, both species are closely related.

#### ﻿﻿*Papuanatuladuplex* sp. nov.

So far, *P.duplex* sp. nov. is unique, based on the conspicuous, paired protuberances on metanotum and abdominal terga. The tips of the cylindrical, abdominal protuberances are covered with long, narrow scales.

### ﻿﻿Genetics

COI barcode sequences were obtained from nine species of *Papuanatula* s. str. (see Table 3). The interspecific distance is mostly rather high (19%–25%, K2P), but in one case 10%–12% (distance between *P.bessa* and *P.parabessa* sp. nov.). In this latter case, there is also a close morphological similarity, which points into the direction that they are closely related and their separation happened not too long ago. However, these distances are in line with genetic distances found in *Labiobaetis* Novikova & Kluge, 1987 in New Guinea (13%–31%; Kaltenbach and Gattolliat 2018). Ball et al. (2005) reported a mean interspecific, congeneric distance of 18% for mayflies from the United States and Canada. The intraspecific distances were mostly between 0% and 1%. Exceptions are a population of *P.bessa* and a population of *P.balkei* sp. nov. with up to 6% genetic distance between them at different locations. Here, the larger genetic distance may be explained by a possible isolation of some locations, while others may still be connected. Intraspecific distances of 4%–6% were also reported in some cases for *Labiobaetis* species in New Guinea, Indonesia, and Borneo (Kaltenbach and Gattolliat 2018, 2019, 2020), as well as in aquatic beetles in the Philippines (Komarek and Freitag 2020). Ball et al. (2005) also reported a case with 6% intraspecific distance in a mayfly in North America and intraspecific K2P distances of more than 3.5% are not uncommon within Plecoptera either (Gill et al. 2015; Gattolliat et al. 2016).

**Table 3.** Intraspecific (bold) and interspecific genetic distances between some species of *Papuanatula* s. str. (COI; Kimura 2-parameter); for location codes see under Material examined of each species.

	Species	Location	1	2	3	4	5	6	7	8	9	10	11	12	13	14	15	16	17	18	19	20	21
**1**	*P.bessa*	PNG161																					
**2**	*P.bessa*	PNG90	**0.01**																				
**3**	*P.bessa*	PNG90	**0.01**	**0.00**																			
**4**	*P.bessa*	PNG96	**0.05**	**0.06**	**0.06**																		
**5**	*P.balkei* sp. nov.	PNG87	0.23	0.24	0.24	0.23																	
**6**	*P.balkei* sp. nov.	PNG87	0.23	0.24	0.24	0.23	**0.00**																
**7**	*P.balkei* sp. nov.	PNG133	0.22	0.23	0.23	0.22	**0.05**	**0.05**															
**8**	*P.balkei* sp. nov.	PNG133	0.22	0.23	0.23	0.22	**0.06**	**0.06**	**0.00**														
**9**	*P.dumspinae* sp. nov.	BH68	0.21	0.22	0.22	0.22	0.22	0.22	0.22	0.23													
**10**	*P.dumspinae* sp. nov.	BH68	0.21	0.22	0.22	0.22	0.22	0.22	0.22	0.23	**0.00**												
**11**	*P.parabessa* sp. nov.	PNG110	0.10	0.11	0.11	0.11	0.20	0.20	0.21	0.21	0.22	0.22											
**12**	*P.parabessa* sp. nov.	PNG128	0.10	0.11	0.11	0.12	0.20	0.20	0.21	0.21	0.22	0.22	**0.01**										
**13**	*P.parabessa* sp. nov.	PNG152	0.10	0.11	0.11	0.12	0.20	0.20	0.21	0.21	0.22	0.22	**0.00**	**0.01**									
**14**	*P.parabessa* sp. nov.	PNG151	0.10	0.11	0.11	0.12	0.19	0.19	0.21	0.21	0.22	0.22	**0.01**	**0.01**	**0.01**								
**15**	*P.parabessa* sp. nov.	PNG151	0.10	0.11	0.11	0.12	0.19	0.19	0.21	0.21	0.22	0.22	**0.01**	**0.01**	**0.01**	**0.00**							
**16**	*P.parabessa* sp. nov.	PNG151	0.10	0.11	0.11	0.12	0.19	0.19	0.21	0.21	0.22	0.22	**0.01**	**0.01**	**0.01**	**0.00**	**0.00**						
**17**	*P.paracopis* sp. nov.	PNG106	0.23	0.24	0.24	0.22	0.23	0.23	0.24	0.24	0.21	0.21	0.22	0.22	0.22	0.22	0.22	0.22					
**18**	*P.paralenos* sp. nov.	PNG87	0.19	0.20	0.20	0.19	0.24	0.24	0.22	0.22	0.22	0.22	0.23	0.23	0.22	0.23	0.23	0.22	0.21				
**19**	*P.paratuber* sp. nov.	BH68	0.22	0.22	0.22	0.22	0.20	0.20	0.21	0.22	0.21	0.21	0.23	0.23	0.23	0.23	0.23	0.22	0.23	0.23			
**20**	*P.parvatubera* sp. nov.	PNG117	0.20	0.21	0.21	0.20	0.25	0.25	0.25	0.25	0.22	0.22	0.23	0.23	0.23	0.22	0.22	0.22	0.21	0.23	0.22		
**21**	*P.webbi* sp. nov.	BH25	0.23	0.23	0.23	0.24	0.23	0.23	0.24	0.25	0.25	0.25	0.25	0.24	0.25	0.24	0.24	0.24	0.25	0.24	0.23	0.20	
**22**	*P.webbi* sp. nov.	BH25	0.22	0.22	0.22	0.23	0.23	0.23	0.24	0.24	0.24	0.24	0.24	0.24	0.24	0.24	0.24	0.24	0.25	0.24	0.23	0.20	**0.00**

### ﻿﻿Diversity and distribution of *Papuanatula*

In general terms, island arc collisions and orogeny are key factors explaining the high biodiversity in New Guinea, as they create opportunities with newly formed, competition-free niches (Gressitt 1982; Toussaint et al. 2014 and citations therein).

The distribution data of *Papuanatula* species in this study show high levels of micro-endemism restricted to smaller areas in New Guinea. This indicates that allopatry could be a major driver of diversity in the genus. Remarkably, based on present data, the genus has a disjunct distribution in New Guinea (incl. New Britain) and the island of Sulawesi. Despite many collection activities and studies on Baetidae in continental Southeast Asia, Indonesia and the Philippines in recent years, no species of *Papuanatula* was found, apart from Sulawesi. However, the mayfly fauna of islands between Sulawesi and New Guinea (e.g., Ambon, Halmahera) is very poorly studied.

The megadiverse genus *Exocelina* Balke, 1998 (Coleoptera, Dytiscidae) was intensively studied in New Guinea, where 152 out of its 209 species worldwide have evolved (Shaverdo and Balke 2022). These studies included dense sampling, molecular phylogenetics and diversification analysis, and demonstrated allopatry to be the main mechanism of diversification in New Guinea (Toussaint et al. 2014). The authors found strong evidence that recent environmental change in the extremely structured central highlands of New Guinea with its ongoing formation of rich aquatic resources and remote valleys and mountain blocks has been the primary driver of diversification in that area, providing the setting for random colonization of new areas followed by isolation and speciation (Toussaint et al. 2013, 2014). There is also evidence that species in running waters are weaker dispersers then species living in standing water, which has been suggested to promote allopatric speciation and micro-endemism in the first group and dispersal in the second group (Ribera et al. 2001; Monaghan et al. 2005). Larvae of *Papuanatula* are rheophilic or even partly torrenticulous. Accordingly, they demonstrate many characters that are usually interpreted as adaptations to fast currents: protuberances on tergites and thoracic sternites; the presence of regular rows of long, dense setae on the legs; posterior setae on the claws; reduced swimming setae on cerci; and a reduced paracercus (Lugo-Ortiz and McCafferty 1999).

## Supplementary Material

XML Treatment for Papuanatula

XML Treatment for Papuanatula

XML Treatment for Papuanatula (Papuanatula) bessa

XML Treatment for Papuanatula (Papuanatula) copis

XML Treatment for Papuanatula (Papuanatula) lenos

XML Treatment for Papuanatula (Papuanatula) plana

XML Treatment for Papuanatula (Papuanatula) tuber

XML Treatment for Papuanatula (Papuanatula) vaisisi

XML Treatment for Papuanatula (Papuanatula) balkei

XML Treatment for Papuanatula (Papuanatula) cyclopomontana

XML Treatment for Papuanatula (Papuanatula) dumspinae

XML Treatment for Papuanatula (Papuanatula) duplex

XML Treatment for Papuanatula (Papuanatula) epibessa

XML Treatment for Papuanatula (Papuanatula) heterochaeta

XML Treatment for Papuanatula (Papuanatula) normungulata

XML Treatment for Papuanatula (Papuanatula) obscura

XML Treatment for Papuanatula (Papuanatula) obscurella

XML Treatment for Papuanatula (Papuanatula) parabessa

XML Treatment for Papuanatula (Papuanatula) paracopis

XML Treatment for Papuanatula (Papuanatula) paralenos

XML Treatment for Papuanatula (Papuanatula) paratuber

XML Treatment for Papuanatula (Papuanatula) parvatubera

XML Treatment for Papuanatula (Papuanatula) pilosa

XML Treatment for Papuanatula (Papuanatula) pluresetae

XML Treatment for Papuanatula (Papuanatula) webbi

XML Treatment for Papuanatula (Papuanatula) zebrata

XML Treatment for Papuafiliola

XML Treatment for Papuanatula (Papuafiliola) stenophylla

XML Treatment for Papuanatula (Papuafiliola) tuberculata

## ﻿﻿Additional information

### Author ORCIDs

Thomas Kaltenbach https://orcid.org/0000-0001-8052-0388

Nikita J. Kluge https://orcid.org/0000-0001-9741-7790

Jean-Luc Gattolliat https://orcid.org/0000-0001-5873-5083

### Data availability

All of the data that support the findings of this study are available in the main text.
